# The association of sarcopenia, possible sarcopenia and cognitive impairment: A systematic review and meta-analysis

**DOI:** 10.1371/journal.pone.0324258

**Published:** 2025-05-28

**Authors:** Jiahui Huang, Min Li, Qiangqiang Luo, Jing Li

**Affiliations:** 1 School of Nursing, Chinese Academy of Medical Sciences & Peking Union Medical College, Beijing, China; 2 Department of Neurosurgery, Peking Union Medical College Hospital, Beijing, China; Instituto Nacional de Geriatria, MEXICO

## Abstract

**Objectives:**

The study aimed to investigate the relationship between sarcopenia, possible sarcopenia, and cognitive impairment, and to analyze the impact of potential moderating factors.

**Methods:**

A comprehensive search was conducted on PubMed, EmBase, Web of Science, Cochrane Library, CNKI, Wanfang Databases, VIP and SinoMed from inception until March 2025. The quality of cross-sectional studies was assessed using the Agency for Healthcare Research and Quality Scale, while the Newcastle-Ottawa scale was used to assess the quality of included case-control and cohort studies. Subgroup analyses and meta-regression were employed to explore potential moderating variables and heterogeneity.

**Results:**

A total of 31 studies were included in this systematic review, of which 27 studies were quantitatively analyzed. The meta-analysis revealed that both sarcopenia and possible sarcopenia significantly associated with cognitive impairment (OR=1.88, 95%CI = 1.71–2.08), (OR=1.96, 95%CI = 1.50–2.58). Subgroup analyses revealed a stronger association between sarcopenia and cognitive impairment in specific demographics: among females with sarcopenia (OR=3.22, 95%CI = 1.23–8.40), in Asian populations (OR=1.96, 95%CI = 1.76–2.18), and within hospital settings (OR=3.12, 95%CI = 2.18–4.48). These findings underscore the influence of gender, ethnicity, and healthcare environment on the relationship between sarcopenia and cognitive impairment. An assessment of publication bias within studies providing adjusted odds ratios indicated potential bias. However, sensitivity analyses and trim-and-filling analyses confirmed the robustness of our findings, suggesting that the observed associations remain reliable despite the presence of publication bias.

**Conclusions:**

Individuals with sarcopenia or possible sarcopenia have approximately twice the odds of developing cognitive impairment compared to those without sarcopenia. Implementing systematic screening and targeted interventions for possible sarcopenia patients is essential to prevent from cognitive decline. Specifically, healthcare professionals should focus on women and inpatients with sarcopenia, employing proactive measures to avert cognitive impairment.

## Introduction

Sarcopenia, characterized by progressive and generalized skeletal muscle disorder leading to accelerated muscle mass and function loss [1], has been officially recognized as an older age disease with an ICD-10-CM diagnostic code [2]. The prevalence of sarcopenia stands at 8%-36% in individuals under 60 years old and 10%-27% in those over 60 years old, exhibiting an escalating trend annually [3]. It is estimated that the number of patients in the world will reach 200 million by 2050 [4]. Possible sarcopenia, a high-risk stage for developing sarcopenia, identifies a population with normal muscle mass but compromised muscle strength and/or physical function, prevalent at 30.0% among middle-aged and older adults in China [5]. Sarcopenia correlates with adverse outcomes, including diminished mobility [6], reduced quality of life [7], falls [8], and heightened mortality risks [9]. Notably, sarcopenia imposes a substantial health economic burden, encompassing both in direct [10] and indirect costs [11,12].

Cognitive impairment denotes the decline or disruption of mental and/or intellectual functioning [13]. It encompasses mild cognitive impairment and dementia, with Alzheimer’s disease emerging as the predominant form of dementia. The 2021 World Alzheimer’s Report [14] highlights dementia as one of the top 10 global causes of death. Presently, there are over 50 million individuals worldwide living with dementia, and this figure is anticipated to escalate to 152 million by 2050 [10]. The economic burden of dementia was roughly estimated at US$ 957.56 billion in 2015, projected to soar to US$ 9.12 trillion by 2050 [11]. Hence, it is crucial to identify and intervene in factors associated with cognitive impairment.

Previous meta-analyses [15–18] have established a significant association between sarcopenia and cognitive impairment. However, these studies primarily employed cross-sectional designs, lacking the support of long-term cohort studies, which limits the understanding of causal relationships. In recent years, there has been a substantial increase in research on this topic, particularly the addition of prospective studies [19–21], which warrants an updated systematic review and meta-analysis. Importantly, sarcopenia is a dynamic condition that may deteriorate or reverse, while possible sarcopenia represents its most active stage [22]. As a transitional phase between normal and sarcopenia, possible sarcopenia carries a 10.3% likelihood of progressing to sarcopenia and a 10.7% chance of reverting to a normal state in patients diagnosed with possible sarcopenia at baseline [22]. Although some research has explored the relationship between sarcopenia and cognitive impairment, the specific connection between possible sarcopenia and cognitive impairment remains under-investigated. Thus, conducting a detailed analysis and intervention for individuals at high risk of cognitive impairment due to sarcopenia is essential. Moreover, factors such as age and gender may significantly influence the relationship between sarcopenia and cognitive impairment, yet relevant research is still insufficient. To address these gaps, this study will systematically review the literature to investigate the relationship between sarcopenia, possible sarcopenia, and cognitive impairment. Additionally, we will analyze the impact of potential moderating factors, including baseline age, gender, publication year, study quality, number of covariates used in the model, and sample size. This research aims to provide a comprehensive understanding of the complex relationship between sarcopenia and cognitive impairment, thereby laying the groundwork for future intervention strategies.

## Materials and methods

This meta-analysis was reported based on the Preferred Reporting Items for Systematic Reviews and Meta-Analysis (PRISMA)2020 guidelines [23] (see S1 Table in S1 Data) and registered in the International Prospective Register of Systematic Reviews (PROSPERO) (CRD42023391557).

### Study selection

A literature search was conducted across multiple databases, including PubMed, EmBase, Web of Science, Cochrane Library, CNKI, Wanfang Data, VIP, SinoMed and Clinical Trials Registry, in both English and Chinese languages, from inception to March 14, 2025. The search strategy utilized a combination of the following terms: “Sarcopenia,” “Cognitive Impairment,” “Mild Cognitive Impairment,” “Cognitive Decline,” and “Dementia.” The particular search strategies for all included databases can be viewed in S1 Table in the S2 Data. Additionally, the reference lists of all retrieved articles were screened to identify other relevant research.

All included studies met the following criteria: (1) Observational studies, including cross-sectional, case-control, or cohort study designs; (2) The study population consisted of both exposure and control groups. The exposure group comprised patients with a definitive diagnosis of sarcopenia or possible sarcopenia. Sarcopenia characterized by low muscle mass (LMM), low muscle strength (LMS), and/or low physical performance (LPP), while possible sarcopenia characterized by LMS and/or LPP. The control group consisted of a healthy population that was matched to the exposure group. The study institutions, regions, and ethnicities were not limited; (3) The study reported an adjusted or crude odds ratio, hazard ratio, or relative risk and 95% confidence intervals. In instances when data were not available, we contacted corresponding authors to request the data to enable inclusion in our meta-analysis.

We excluded studies without primary data (animal experiments, review articles or case reports); conference presentations without information about the methods or the outcomes; studies in languages other than English or Chinese; and studies that evaluated cognitive impairment as a continuous measure.

Studies of the same research project were included only when they reported the results in different metrics (odds ratio or relative risk or hazard ratio). In addition, when two or more studies reported data from the same research project, we selected the most recently published.

### Outcomes

The primary outcome measure was the adjusted odds ratio (and 95% confidence interval) for cognitive decline/impairment in individuals with sarcopenia or possible sarcopenia, compared to those without sarcopenia.

### Data extraction and quality assessment

Data were systematically extracted using a standardized excel template that included basic literature details (first author, publication year, study area, institution), study design (cross-sectional, case-control, or cohort), sample size, participant demographics (age, gender), diagnostic criteria for sarcopenia or possible sarcopenia and cognitive impairment, odds ratio, relative risk, hazard ratio, and 95% confidence intervals, along with the number of covariates. For the adjusted meta-analysis, the data from the most comprehensively adjusted model in each report were utilized.

Quality assessment for cross-sectional studies was conducted using the Agency for Healthcare Research and Quality Scale (AHRQ) [24], comprising 11 items scored 1 (“yes”) or 0 (“no”/”unclear”), totaling up to 11 points. Studies scored 1–4, 5–7, and 8–11 were classified as low, moderate, and high quality, respectively. The Newcastle-Ottawa scale (NOS) [25] was applied to evaluate case-control and cohort studies, with a maximum score of 9: 1–3 indicating low quality, 4–6 moderate, and 7–9 high quality.

The literature screening, data extraction, and risk of bias assessment were independently performed by two researchers, who also conducted an independent and standardized evaluation of data extraction and quality assessment for an initial subset of five studies, following a predefined protocol. Discrepancies in any phase of the process were resolved through consensus or, if required, by consulting a third researcher to ensure methodological rigor and consistency.

### Data synthesis and analysis

In our meta-analysis, we employed a random-effects model when significant statistical heterogeneity was identified (*I*^2^ > 50%, *P* < 0.05). Conversely, a fixed-effects model was utilized in the absence of significant heterogeneity. Inverse variance method was selected to calculate the combined effects [26]. Analysis for adjusted odds ratio, crude odds ratio, adjusted relative risk/hazard ratio, and crude relative risk/hazard ratio were conducted separately. Meta-analyses were performed when more than two studies could be pooled. For the adjusted odds ratio and adjusted relative risk/hazard ratio, we pooled the estimates using the model with the greatest number of covariates presented by the authors. Subgroup analyses were conducted to investigate the relationships among various factors, including: 1) gender, 2) diagnostic criteria for sarcopenia, 3) tools of assessing cognitive impairment, 4) tools used for measuring muscle mass, 5) different geographical regions (different continents), 6) study settings, 7) study designs, 8) types of cognitive impairment, and 9) the adjustment for potential confounders (age and gender). Furthermore, we performed a meta-regression analysis on studies that reported adjusted odds ratios, relative risks, and hazard ratios individually. The aim was to identify potential moderators influencing the relationship between sarcopenia and cognitive impairment. The moderators assessed encompassed publication year, total sample size, average participant age at baseline, percentage of females, the number of covariates in the model, and study quality. To systematically evaluate study quality, we utilized a structured scoring system, categorizing studies into three tiers of quality: low (0), moderate (1), and high (2). Publication bias was assessed using funnel plots and Egger’s test for meta-analyses comprising 10 or more studies, with a trim-and-filling analysis conducted to adjust for detected asymmetry. To enhance statistical power, our primary analysis included studies providing effect size data on the relationship between pure sarcopenia or possible sarcopenia and cognitive impairment, even if these studies primarily focused on additional comorbidities such as sarcopenic obesity or sarcopenia combined with osteoporosis. To ensure the robustness of our findings and prevent potential underestimation of effect size due to studies where sarcopenia was compounded with other comorbidities, we conducted a sensitivity analysis excluding these studies. Sensitivity analyses were also performed to assess the research’s quality and stability. The data analysis employed Review Manager 5.3 (RevMan, The Nordic Cochrane Centre, The Cochrane Collaboration, 2014) and Stata 14 (Stata Corp, College Station, TX, USA). All tests were two-tailed and *p* < 0.05 was set as the level of significance.

## Results

### Search and study selection

**Fig 1:**
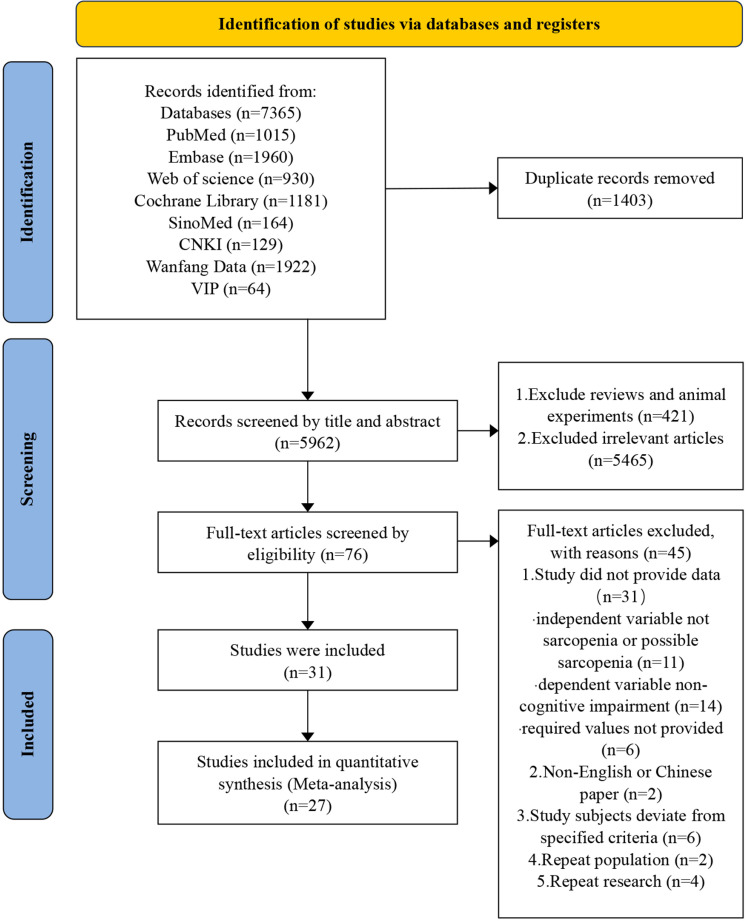
Flow chart of literature searching and screening.

The detailed process of literature searching and study selection was presented in the flow chart (Fig 1). A total of 7365 references were obtained in the initial search, 5962 references were obtained after the removal of duplicate references. A total of 76 studies were obtained by reading the titles and abstracts and excluding the reviews, meta-analysis, animal experiments and literatures with inconsistent research contents. After reading the full text and conducting secondary screening according to the exclusion criteria, a total of 31 studies [19–21,27–54] were finally included. S1 and S2 Tables in S3 Data presented a numbered list of the 5962 studies identified in the literature search after deduplication, along with the reasons for exclusion for each excluded study. To ensure a comprehensive literature review, we also searched for citing documents of the initially included studies and examined their reference lists. However, this additional search did not yield any new studies that met our inclusion criteria.

### Characteristics of included studies

A comprehensive summary of the included studies is presented in Table 1, with further detailed data provided in S2 Table of the S2 Data. The whole data supporting the findings is provided in S4 Data. Our analysis encompassed 31 studies, involving a total of 543258 participants ranging in average age from 58.0 to 86.4 years. The majority of these studies, 27 in total [19,20,27–41,43–49,51–53], reported crude or adjusted odds ratios, while two [21,42] provided relative risk and two reported [50,54] hazard ratio measures. A significant portion of the articles, 24 in number [20,21,27–32,35–38,40–43,45–51,53], delved into the influence of sarcopenia on cognitive impairment, whereas four articles [33,44,52,54] explored the relationship between possible sarcopenia and cognitive impairment, and an additional three studies [19,34,39] investigated the concurrent association between sarcopenia, possible sarcopenia, and cognitive impairment. One study [42] was exclusively male, two studies [39,46] exclusively female, and the remaining 28 had 0.90% to 89.90% female participants. The studies, published between 2013 and 2024, comprised 25 cross-sectional studies [27–39,41–49,51–53] and 6 cohort studies [19–21,40,50,54]. Most of the included studies were conducted in communities and Asia, 21 studies [19,21,27–32,35,37–41,43,44,47–49,51,53] were from Asia, 5 studies [34,42,45,46,54] were from Europe, and 4 studies [20,33,50,52] were from America. One study [36] conducted in six countries including China, Ghana, India, Mexico, Russia, and South Africa. There were 27 studies [19–21,27,29–33,35–37,39–44,46–54] conducted in community-dwelling setting and 4 studies [28,34,38,45] conducted in hospital settings. Regarding the selection of diagnostic criteria for sarcopenia, 9 studies [30,32,35,37,39,40,43,48,49] adhered to the Asian Working Group for Sarcopenia (AWGS 2014) criteria, while 11 studies [19,21,27–29,31,38,41,44,51,53] favored the modified AWGS (AWGS 2019) criteria. One study [46] adopted the European Working Group on Sarcopenia in Older People (EWGSOP) criteria, and 7 studies [20,33,34,36,45,52,54] followed the modified EWGSOP (EWGSOP 2) criteria. Ishii’s screening test (Ishii) and the Foundation for the National Institutes of Health (FNIH) were chosen in 3 studies [42,47,50], respectively. To assess subjects’ cognitive function, 15 studies [21,28–30,32,33,35,37,39–41,45,47,49,52] utilized the Mini-Mental State Examination (MMSE), 3 studies [27,43,48] employed the Montreal Cognitive Assessment (MoCA), 1 study [44] employed MMSE and MoCA to evaluate different type of cognitive impairment, 4 studies [20,34,36,53] utilized the National Institute on Aging—Alzheimer’s Association (NIA/AA), and 7 studies [19,31,38,42,46,50,51] employed other cognition assessment tools. A large-scale prospective study [54] using the UK Biobank database assessed dementia occurrence based on medical records and diagnostic codes, without directly utilizing standardized cognitive assessment tools. Seventeen studies [19,20,27,29,32,34,36,38–40,42,44,45,51–54] investigated the association of sarcopenia/possible sarcopenia and specific type of cognitive impairment. However, the remaining studies [21,28,30,31,33,35,37,41,43,46–50] did not specify the type of cognitive impairment. The overall quality of the included studies was considerable, with 14 medium-quality studies [28,34,38,41–48,51–53] and 17 high-quality studies [19–21,27,29–33,35–37,39,40,49,50,54] (see S3 and S4 Tables in S2 Data). Seven studies [28,29,44,50–53], although primarily focused on other comorbidities such as sarcopenic obesity or sarcopenia combined with osteoporosis, provided relevant effect size data.

**Table 1:** Characteristics of included studies.

Study ID		Population						Exposure			Outcomes	
Author (year)	Study design	Country	Sample size	Source of participants	Age, mean	Female %	Follow-up (years)	Sarcopenia Status	Diagnosis criteria	Muscle mass assessment tools	Type of cognitive impairment	Cognition assessment tools
**The studies provided for odds ratio.**												
Bian 2023 [31]	Cross-sectional	China	720	Community	70.92 ± 4.73	59.44	–	Sarcopenia	AWGS2019	BIA	CI	Memory guard
Ohta 2023 [41]	Cross-sectional	Japan	6426	Community	74.00 ± 9.00	65.45	–	Sarcopenia	AWGS2019	BIA	CI	MMSE
Deng 2022 [28]	Cross-sectional	China	836	Hospital	77.19 ± 8.38	58.61	–	Sarcopenia	AWGS2019	BIA	CI	MMSE
Wu 2021 [47]	Cross-sectional	China	2525	Community	84.50 ± 11.30	0.90	–	Sarcopenia	Ishii	CC	CI	MMSE
Zhu 2021 [49]	Cross-sectional	China	923	Community	72.37 ± 5.23	58.61	–	Sarcopenia	AWGS2014	BIA	CI	MMSE
Cipolli 2021 [33]	Cross-sectional	Brazil	529	Community	80.80 ± 4.90	70.10	–	Possible Sarcopenia	EWGSOP2	/	CI	MMSE
Xu 2020 [48]	Cross-sectional	China	582	Community	86.40 ± 3.50	57.70	–	Sarcopenia	AWGS2014	BIA	CI	MoCA
Kim 2019 [37]	Cross-sectional	Korea	1887	Community	75.80 ± 3.90	48.30	–	Sarcopenia	AWGS2014	DXA	CI	MMSE
Peng 2019 [43]	Cross-sectional	China	417	Community	76.00	54.44	–	Sarcopenia	AWGS2014	/	CI	MoCA
Wang 2018 [30]	Cross-sectional	China	915	Community	68.27 ± 6.24	50.71	–	Sarcopenia	AWGS2014	BIA	CI	MMSE
Huang 2015 [35]	Cross-sectional	China	731	Community	73.40 ± 5.40	46.20	–	Sarcopenia	AWGS2014	DXA	CI	MMSE
Van Kan 2013 [46]	Cross-sectional	France	2691	Community	≥75.00	100.00	–	Sarcopenia	EWGSOP	DXA	CI	SPMSQ
Fu 2023 [51]	Cross-sectional	China	2451	Community	62.10 ± 6.10	89.90	–	Sarcopenia	AWGS2019	BIA	MCI	AVLT or DSST or VFT or TMT-B
Sun 2023 [29]	Cross-sectional	China	1268	Community	71.80 ± 5.80	69.60	–	Sarcopenia	AWGS2019	BIA	MCI	MMSE
Lee 2023 [38]	Cross-sectional	Korea	286	Hospital	74.03	40.21	–	Sarcopenia	AWGS2019	BIA	MCI	SNSB-C
O’Donovan 2022 [52]	Cross-sectional	Colombia	5760	Community	71.00 ± 8.00	59.64	–	Possible Sarcopenia	EWGSOP2	/	MCI	Shorter version of MMSE
Jacob 2021 [36]	Cross-sectional	China、 Ghana、 India、 Mexico、 Russia、 South Africa	12912	Community	72.20 ± 10.80	54.80	–	Sarcopenia	EWGSOP2	Formular^a^	MCI	NIA/AA
Bai 2021 [27]	Cross-sectional	China	428	Community	86.34 ± 3.57	60.32	–	Sarcopenia	AWGS2019	BIA	MCI	MoCA
Chen 2021 [32]	Cross-sectional	China	1394	Community	/	58.82	–	Sarcopenia	AWGS2014	BIA	MCI	MMSE
Lee 2018 [39]	Cross-sectional	Korea	201	Community	74.30 ± 6.60	100.00	–	Sarcopenia、 Possible sarcopenia	AWGS2014	DXA	MCI	MMSE
Salinas 2021 [20]	Cohort	Mexico	496	Community	65.50 ± 7.30	65.10	8	Sarcopenia	EWGSOP2	Formular^a^	MCI	NIA/AA
Hu 2022 [19]	Cohort	China	2982	Community	67.3 ± 6.0	43.80	3	Sarcopenia、 Possible sarcopenia	AWGS2019	Formular^b^	MCI	HRS
Someya 2022 [44]	Cross-sectional	Japan	1615	Community	73.10 ± 5.40	57.60	–	Possible Sarcopenia	AWGS2019	DXA	MCI、AD	MoCA、 MMSE
Weng 2023 [53]	Cross-sectional	China	176	Community	71.40 ± 4.80	63.07	–	Sarcopenia	AWGS2019	BIA	AD	NIA/AA
Dost 2022 [34]	Cross-sectional	Turkey	662	Hospital	73.60 ± 7.50	67.40	–	Sarcopenia、 Possible sarcopenia	EWGSOP2	BIA	AD	NIA/AA
Suzan 2022 [45]	Cross-sectional	Turkey	339	Hospital	76.90 ± 7.10	71.68	–	Sarcopenia	EWGSOP2	DXA	AD	MMSE
Nishiguchi 2016 [40]	Cohort	Japan	131	Community	74.20 ± 5.30	62.60	1	Sarcopenia	AWGS2014	BIA	CD	MMSE
**The studies provided for relative risk.**												
Ramoo 2022 [21]	Cohort	Malaysia	1946	Community	60.00-79.00	62.40	1	Sarcopenia	AWGS2019	BIA	CI	MMSE
Papachristou 2015 [42]	Cross-sectional	Britain	1570	Community	78.00	0.00	–	Sarcopenia	FNIH	BMI	MCI	TYM
**The study provided for hazard ratio.**												
Batsis 2021 [50]	Cohort	America	5822	Community	75.00-80.00	55.70	8	Sarcopenia	FNIH	/	CI	AD-8 or 10-word recall task or orientation or clock-drawing test
Ling 2024 [54]	Cohort	United Kingdom	483637	Community	58.00	54.20	13.6	Possible sarcopenia	EWGSOP2	BIA	AD	/

Abbreviations: AWGS, Asian Working Group for Sarcopenia; EWGSOP, European Working Group on Sarcopenia in Older People; Ishii, a simple screening test for sarcopenia in older adults created by Ishii; FNIH, Foundation for the National Institutes of Health; BIA, Bioelectrical Impedance Analysis; DXA, Dual-Energy X-ray Absorptiometry; CC, Calf Circumference; ^a^Fomular, Appendicular Skeletal Muscle Mass(ASM)=0.244*weight+7.8*height+6.6*gender−0.098*age+race−3.3 (where female = 0 and male = 1; race = 0 [White and Hispanic], race = 1.9 [Black] and race = 1.6 [Asian]). ASM was further divided by BMI based on measured weight and height to calculate the appendicular muscle mass index; ^b^Fomular, ASM = 0.193*weight+0.107*height -4.157* gender (male = 1, female = 0)-0.037*age-2.631. CI, cognitive impairment; MCI, mild cognitive impairment; AD, Alzheimer’s disease; CD, cognitive decline; MMSE, Mini Mental State Examination; MoCA, Montreal Cognitive Assessment; SPMSQ, Short Portable Mental Status Questionnaire; AVLT, Auditory Verbal Learning Test; DSST, Digit Symbol Substitution Test; VFT, Verbal Fluency Test; TMT-B, Trail-making Test B; SNSB-C, Seoul Neuropsychological Screening Battery Core; NIA/AA, National Institute on Aging—Alzheimer’s Association; HRS, a cognitive function based on the method used in the American Health and Retirement Study (HRS); TYM, Test Your Memory.

### Sarcopenia and cognitive impairment

#### Sarcopenia versus non-sarcopenia.

**Fig 2:**
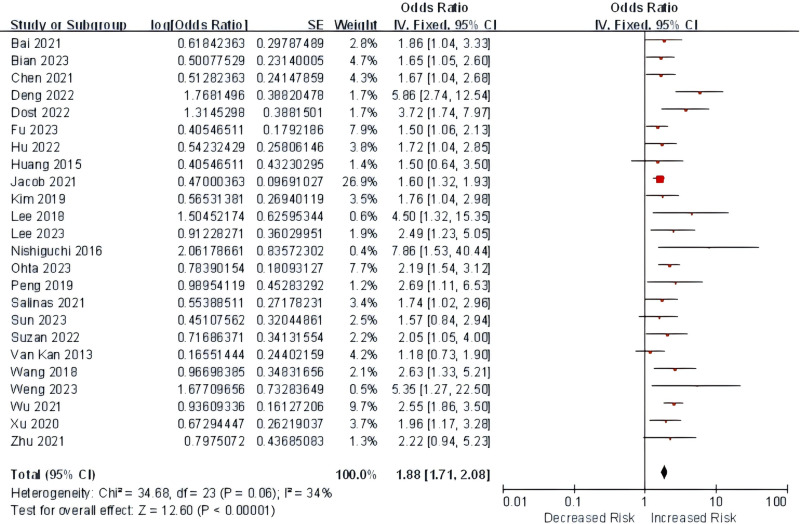
Forest plot of the adjusted odds ratios for the association between sarcopenia and cognitive impairment.

People with sarcopenia were at increased odds of cognitive impairment when compared with people without sarcopenia in adjusted(adjusted odds ratio = 1.88, 95%*CI* = 1.71, 2.08, *p* < 0.001; *I*^2^ = 34%, *Q* = 34.68, *p* = 0.06; fixed-effects model) (Fig 2) and crude odds ratio(odds ratio = 2.75, 95%*CI* = 2.26, 3.34, *p* < 0.001; *I*^2^ = 66%, *Q* = 56.54, *p* < 0.001;random-effects model) (S1 Fig in S2 Data) analysis. Given the restricted number of studies available, we were unable to perform a meta-analysis combining the adjusted and crude relative risks and hazard ratios. The funnel plots of adjusted odds ratio and crude odds ratio were presented in S2 and S3 Figs. Publication bias was evidenced for adjusted odds ratio (Egger’s intercept = 1.41, *p* = 0.006). The trim-and-filling technique adjusted the effects to an adjusted odds ratio of 1.812 (95%*CI* = 1.553, 2.115, p < 0.001;*Q* = 57.19, *p* = 0.001; random-effects model) (see S4 Fig). No publication bias was found by crude odds ratio (Egger’s intercept = 0.13, *p* = 0.914).

#### Subgroup and sensitivity analysis.

Significant associations between sarcopenia and cognitive impairment were observed across genders in studies that provided data for both adjusted and crude odds ratio analyses. This correlation appears to be a global phenomenon, with consistent evidence from Asia, Europe, and the Americas supporting a significant association between sarcopenia and cognitive impairment. Notably, this association was absent in African studies, suggesting regional differences that warrant further investigation. The significance of the relationship held true in various methodological contexts, including diverse criteria for sarcopenia assessment and multiple tools for evaluating cognitive impairment. An exception was noted in a single study employing both the EWGSOP criteria and the SPMSQ tool, which failed to demonstrate a significant link, thereby underscoring the necessity for diverse assessment methodologies in research. The consistency of these findings across different study designs, populations, and muscle mass measurement tools underscores the robustness of the observed relationship. The details of the subgroup analyses are summarized in Table 2.

**Table 2:** The results of subgroup analysis for odds ratio of sarcopenia/possible sarcopenia and cognitive impairment.

Subgroup	Outcomes	Numbers of studies	Heterogeneity		Effect model	Meta-analysis results	Effect size	
			*I*^2^(%)	*p*			*Z*	*p*
Studies with adjusted odds ratio of sarcopenia and cognitive impairment								
**Total**		24 [19,20,27–32,34–41,43,45–49,51,53]	33.7	0.056	fixed	1.883(1.707-2.078)	12.60	<0.001
**Gender**								
Male		8 [27,28,30,36–38,41,43]	0.0	0.800	random	1.756(1.447-2.131)	5.71	<0.001
Female		8 [27,28,30,36,38,39,41,46]	97.4	<0.001	random	3.219(1.234-8.396)	2.39	0.017
**Criteria for sarcopenia**								
AWGS 2014		9 [30,32,35,37,39,40,43,48,49]	0.0	0.573	fixed	2.044(1.625-2.570)	6.12	<0.001
AWGS 2019		9 [19,27–29,31,38,41,51,53]	43.6	0.077	fixed	1.932(1.630-2.290)	7.58	<0.001
EWGSOP		1 [46]	/	/	/	1.180(0.731-1.904)	0.68	0.498
EWGSOP 2		4 [20,34,36,45]	37.1	0.189	fixed	1.708(1.443-2.022)	6.23	<0.001
Ishii		1 [47]	/	/	/	2.550(1.859 − 3.498)	5.80	<0.001
**Cognition assessment tool**								
MMSE		12 [28–30,32,35,37,39–41,45,47,49]	26.4	0.185	fixed	2.252(1.917-2.646)	9.87	<0.001
MoCA		3 [27,43,48]	0.0	0.781	fixed	2.020(1.418-2.878)	3.90	<0.001
NIA/AA		4 [20,34,36,53]	56.6	0.075	fixed	1.716(1.443-2.040)	6.12	<0.001
Memory guard		1 [31]	/	/	/	1.650(1.048-2.597)	2.16	0.030
HRS		1 [19]	/	/	/	1.720(1.037-2.852)	2.10	0.036
SNSB-C		1 [38]	/	/	/	2.490(1.229-5.045)	2.53	0.011
AVLT or DSST or VFT or TMT-B		1 [51]	/	/	/	1.500(1.056-2.131)	2.26	0.024
SPMSQ		1 [46]	/	/	/	1.180(0.731-1.904)	0.68	0.498
**Muscle mass assessment tool**								
BIA		14 [27–32,34,38,40,41,48,49,51,53]	36.3	0.085	fixed	2.038(1.754-2.367)	9.30	<0.001
DXA		5 [35,37,39,45,46]	19.2	0.292	fixed	1.621(1.218-2.157)	3.31	0.001
Formular		3 [19,20,36]	0.0	0.934	fixed	1.627(1.374 − 1.925)	5.65	<0.001
CC		1 [47]	/	/	/	2.550(1.859-3.498)	5.80	<0.001
**Study region**								
Asia		21 [19,27–32,35–41,43,47–49,51,53]	22.5	0.173	fixed	1.955(1.755-2.177)	12.21	<0.001
Europe		4 [34,36,45,46]	55.4	0.081	fixed	1.708(1.223-2.386)	3.14	0.002
Africa		2 [36]	0.0	0.564	fixed	1.146(0.625-2.099)	0.44	0.659
America		2 [20,36]	0.0	0.507	fixed	1.569(1.014-2.428)	2.02	0.043
**Study sitting**								
Community-dwelling		20 [19,20,27,29–32,35–37,39–41,43,46–49,51,53]	12.7	0.296	fixed	1.808(1.632-2.003)	11.34	<0.001
Hospital		4 [28,34,38,45]	36.9	0.191	fixed	3.120(2.176-4.475)	6.19	<0.001
**Study design**								
Cross-sectional		21 [27–32,34–39,41,43,45–49,51,53]	36.6	0.048	fixed	1.885(1.702-2.089)	12.13	<0.001
Cohort		3 [19,20,40]	36.0	0.209	fixed	1.859(1.300-2.659)	3.40	0.001
**Type of cognitive impairment** **Sarcopenia**								
MCI		9 [19,20,27,29,32,36,38,39,51]	0.0	0.810	fixed	1.665(1.456-1.903)	7.46	<0.001
AD		3 [34,45,53]	6.9	0.341	fixed	2.868(1.785-4.609)	4.36	<0.001
CD		1 [40]	/	/	/	7.860(1.528-40.438)	2.47	0.014
**Possible sarcopenia**								
MCI		4 [19,34,39,44]	0.0	0.486	fixed	1.525(1.257-1.852)	4.27	<0.001
AD		2 [34,44]	0.0	0.779	fixed	3.110(2.047-4.724)	5.32	<0.001
**Adjustments**								
Age and gender		4 [35,48,51,53]	23.3	0.271	fixed	1.877(1.462-2.409)	4.94	<0.001
Studies with crude odds ratio of sarcopenia and cognitive impairment								
**Total**		20 [19,20,27–32,34,35,38,39,41,43,45–49,53]	66.4	<0.001	random	2.748(2.261-3.340)	10.16	<0.001
**Gender**								
Male		5 [27,28,30,38,41]	53.5	0.072	fixed	2.463(1.819-3.335)	5.83	<0.001
Female		6 [27,28,30,38,41,46]	73.8	0.002	random	3.267(1.964-5.437)	4.56	<0.001
**Criteria for sarcopenia**								
AWGS 2014		7 [30,32,35,39,43,48,49]	0.0	0.492	random	2.556(2.073-3.152)	8.78	<0.001
AWGS 2019		8 [19,27–29,31,38,41,53]	63.3	0.008	random	2.723(2.028-3.655)	6.66	<0.001
EWGSOP		1 [46]	/	/	/	1.390(0.906-2.133)	1.51	0.132
EWGSOP 2		3 [20,34,45]	71.2	0.031	random	2.922(1.629-5.244)	3.59	<0.001
Ishii		1 [47]	/	/	/	4.810(3.618 − 6.396)	10.81	<0.001
**Cognition assessment tool**								
MMSE		10 [28–30,32,35,39,41,45,47,49]	64.4	0.003	random	3.161(2.431-4.109)	8.60	<0.001
MoCA		3 [27,43,48]	2.6	0.358	random	2.206(1.627-2.993)	5.09	<0.001
NIA/AA		3 [20,34,36]	74.1	0.021	random	3.401(1.580-7.323)	3.13	0.002
Memory guard		1 [31]	/	/	/	1.767(1.142-2.734)	2.56	0.011
HRS		1 [19]	/	/	/	2.850(1.942-4.183)	5.35	<0.001
SNSB-C		1 [38]	/	/	/	2.590(1.333-5.032)	2.81	0.005
SPMSQ		1 [46]	/	/	/	1.390(0.906-2.133)	1.51	0.132
**Muscle mass assessment tool**								
BIA		12 [27–32,34,38,41,48,49,53]	59.2	0.005	random	2.708(2.147-3.415)	8.42	<0.001
DXA		4 [35,39,45,46]	61.7	0.050	random	2.412(1.434-4.058)	3.32	0.001
Formular		2 [19,20]	44.3	0.180	random	2.370(1.588 − 3.537)	4.22	<0.001
CC		1 [47]	/	/	/	4.810(3.618-6.396)	10.81	<0.001
**Study region**								
Asia		16 [19,27–32,35,38,39,41,43,47–49,53]	61.4	0.001	random	2.855(2.327-3.502)	10.06	<0.001
Europe		3 [34,45,46]	84.3	0.002	random	2.646(1.221-5.731)	2.47	0.014
America		1 [20]	/	/	/	1.890(1.190-3.001)	2.70	0.007
**Study setting**								
Community-dwelling		16 [19,20,27,29–32,35,39,41,43,46–49,53]	64.8	<0.001	random	2.525(2.060-3.094)	8.93	<0.001
Hospital		4 [28,34,38,45]	64.0	0.040	random	4.100(2.411-6.972)	5.21	<0.001
**Study design**								
Cross-sectional		18 [27–32,34,35,38,39,41,43,45–49,53]	68.4	<0.001	random	2.815(2.266-3.497)	9.35	<0.001
Cohort		2 [19,20]	44.3	0.180	random	2.370(1.588-3.537)	4.22	<0.001
**Type of cognitive impairment** **Sarcopenia**								
MCI		7 [19,20,27,29,32,38,39]	0.0	0.448	random	2.304(1.916-2.771)	8.87	<0.001
AD		3 [34,45,53]	27.7	0.251	random	3.835(2.393-6.147)	5.58	<0.001
**Possible sarcopenia**								
MCI		3 [19,39,52]	0.0	0.691	random	1.804(1.485, 2.192)	5.94	<0.001
AD		1 [34]	/	/	/	4.286(2.851, 6.443)	7.00	<0.001

Abbreviations: AWGS, Asian Working Group for Sarcopenia; EWGSOP, European Working Group on Sarcopenia in Older People; Ishii, a simple screening test for sarcopenia in older adults created by Ishii; MMSE, Mini Mental State Examination; MoCA, Montreal Cognitive Assessment; NIA/AA, National Institute on Aging—Alzheimer’s Association; HRS, the cognitive function based on the method used in the American Health and Retirement Study; SNSB-C, Seoul Neuropsychological Screening Battery Core; AVLT, Auditory Verbal Learning Test; DSST, Digit Symbol Substitution Test; VFT, Verbal Fluency Test; TMT-B, Trail-making Test B; SPMSQ, Short Portable Mental Status Questionnaire; BIA, Bioelectrical Impedance Analysis; DXA, Dual-Energy X-ray Absorptiometry; ^a^Fomular, Appendicular Skeletal Muscle Mass(ASM)=0.244*weight+7.8*height+6.6*gender−0.098*age+race−3.3 (where female = 0 and male = 1; race = 0 [White and Hispanic], race = 1.9 [Black] and race = 1.6 [Asian]). ASM was further divided by BMI based on measured weight and height to calculate the appendicular muscle mass index; ^b^Fomular, ASM = 0.193*weight+0.107*height -4.157*gender (male = 1, female = 0)-0.037*age-2.631; CC, Calf Circumference; MCI, mild cognitive impairment; AD, Alzheimer’s disease; CD, cognitive decline.

We removed the studies that investigating the relationship between sarcopenia combined with obesity or osteoporosis and cognitive impairment in adjusted and crude odds ratio analysis. The results remained significant for adjusted and crude odds ratio analysis (see S5 and S6 Figs in the data S2 Data). In addition, our sensitivity analysis revealed that all the results remained significant and were not significantly impacted by any individual study (see S7 and S8 Figs).

#### Meta-regressions.

Publication year, total number of participants, average age of participants at baseline, percentage of females, the number of covariates used in the model and study quality were investigated as potential moderators through meta-regression analysis. None of the investigated moderators significantly explained the variance of the relationship of sarcopenia and cognitive impairment in adjusted odds ratio analysis. The detailed results of the meta-regressions are summarized in Table 3.

**Table 3:** Meta-Regression analysis of moderators influencing the relationship between sarcopenia and cognitive impairment in observational studies.

Moderator	Number of studies	*β*	95%*CI*	*p*	*R* ^2^
Publication year	22	0.99	0.90,1.09	0.851	100.00
Sample size	22	1.00	1.00, 1.00	0.071	
Age	22	1.01	0.98,1.03	0.549	
Percent females	22	1.00	0.99,1.01	0.561	
Study quality	22	0.80	0.60,1.08	0.141	
Number of covariates	22	0.96	0.92,1.01	0.112	

### Possible sarcopenia and cognitive impairment

#### Possible sarcopenia versus non-possible sarcopenia.

People with possible sarcopenia were at increased odds of cognitive impairment when compared with people without possible sarcopenia in adjusted(adjusted odds ratio = 1.96, 95%*CI* = 1.50, 2.58, *p* < 0.001; *I*^2^ = 54.6%, *Q* = 13.21, *p* = 0.04; random-effects model) (S9 Fig) and crude odds ratio(odds ratio = 2.40, 95%*CI* = 1.66, 3.46, *p* < 0.001; *I*^2^ = 73.7%, *Q* = 15.22, *p* = 0.004;random-effects model) (S10 Fig) analysis.

#### Subgroup and sensitivity analysis.

The subgroup results indicated that regardless of whether the studies were adjusted or not, possible sarcopenia is significantly associated with cognitive impairment. However, it is noteworthy that the effect size in the unadjusted studies for AD is larger, which may reflect the impact of confounding factors. Sensitivity analyses detected that the adjusted and unadjusted odds ratio of possible sarcopenia and cognitive impairment were not significantly affected by any individual study (see S11 and S12 Figs).

## Discussion

The present meta-analysis unveils a compelling association between sarcopenia and cognitive impairment. Our findings indicate that individuals with sarcopenia have much higher odds of cognitive impairment compared to those without the condition, with an adjusted odds ratio of 1.88 and a crude odds ratio of 2.75. This relationship persists across genders and regions, suggesting a global relevance that transcends geographical and demographic boundaries. Additionally, this study introduces a new perspective by delving into the relationship between possible sarcopenia and cognitive impairment. By exploring this less-researched area, we aim to broaden our understanding of the early signs and risk factors associated with cognitive decline.

The interplay between sarcopenia and cognitive decline is multifaceted, with aging being a key factor leading to the deterioration of both muscle and cognitive functions. Studies indicated that sarcopenia shares common pathophysiological links with cognitive impairments, characterized by elevated levels of pro-inflammatory cytokines, reduced levels of anti-inflammatory cytokines, mitochondrial dysfunction, oxidative stress, and disruptions in the gut microbiome [55–59]. Moreover, muscles, considered endocrine organs, express and secrete a variety of myokines, including cytokines (e.g., IL6), peptides (e.g., FNDC5/Irisin), and growth factors (e.g., BDNF). The contraction of skeletal muscles plays a significant role in regulating cognitive functions, which also explains the mechanism by which exercise improves cognitive abilities [60–63]. Imbalances in myokine secretion in sarcopenia are associated with the disruption of the muscle-brain axis, adversely impacting higher cognitive functions such as learning, memory, and motor coordination [64], thereby connecting muscle health with neurological integrity. Changes in testosterone [65], insulin [66], and growth hormone-releasing hormone [67,68] are also related to the regulation of muscle and brain functions. The convergence of genetic susceptibility and environmental exposures, such as the APOE gene [69] and the Amyloid Precursor Protein (APP) [70], along with lifestyle and nutritional status, further complicates the relationship between sarcopenia and cognitive disorders. APP, a transmembrane protein abundantly expressed at CNS synapses and the Neuromuscular Junction [71], is crucial for muscle contraction and neurotransmission. Variants in APP are associated with a decline in muscle mass and strength, and its abnormal processing leads to the production of Amyloid Beta (Aβ), a pathological hallmark of Alzheimer’s disease (AD) [72]. Although these potential mechanisms are still under investigation, our research suggests a stronger correlation between sarcopenia and AD compared to mild cognitive impairment (MCI), indirectly supporting the potential roles of these pathways.

The association of sarcopenia and cognitive impairment have been extensively studied and quantified in numerous systematic reviews and meta-analyses [15,16,18,73]. Consistent with previous research, our study also confirmed that sarcopenia is significantly associated with cognitive impairment. This relationship remained robust across various factors, including geographical region, participant demographics, gender, publication year, study quality, and the number of confounding factors used in the studies. Divergent from prior studies, our investigation introduces a detailed subgroup analysis and meta-regression to explore whether different participant characteristics, types of cognitive impairment, and assessment tools affect the relationship between these two conditions. Additionally, we provide an extra layer of analysis by examining the often-overlooked transitional state of “possible sarcopenia” and its association with cognitive impairment.

The EWGSOP2 [74] identifies low muscle strength as a crucial indicator of sarcopenia, while the AWGS in 2019 defines “possible sarcopenia” based on the presence of low muscle strength and/or low physical function. Our study reveals a stronger correlation between possible sarcopenia and cognitive impairment compared to that observed with sarcopenia. Previous researches [32,35,75] have suggested that this association may be explained by the interrelationship between key components of sarcopenia—such as muscle strength and physical function—and cognitive function. Imaging studies have indicated that brain atrophy, which is associated with decreased muscle mass, may contribute to cognitive decline in individuals with sarcopenia [76]. However, evidence linking muscle quality, cognitive impairment, and dementia remains limited. For instance, an interventional study involving patients with Alzheimer’s disease demonstrated improvements in muscle quality following nutritional supplementation, yet no significant changes in cognitive function were observed [77]. This suggests that the connection between muscle strength, physical function, and cognitive performance may be more pronounced [78]. Our study underscores the importance of assessing muscle strength and physical function in older adults, particularly in regions with limited medical resources. Early identification of patients with possible sarcopenia could be beneficial in preventing or mitigating adverse cognitive outcomes.

Subgroup analyses, regardless of the method used (BIA, DXA, or simple muscle mass calculation formulas), consistently indicate that sarcopenia is an influencing factor for cognitive impairment. Studies utilizing Bioelectrical Impedance Analysis (BIA) demonstrated the strongest correlation. BIA has notable advantages including being radiation-free, affordable, and portable [74], rendering it suitable for primary healthcare settings and large-scale epidemiological screenings [79]. However, compared to Dual-Energy X-ray Absorptiometry (DXA), a tool preferred for clinical muscle mass evaluation [74], BIA tends to overestimate muscle mass [80,81], which may raise the diagnostic threshold for sarcopenia and strengthen the association between sarcopenia and cognitive impairment. Kawakami et al. [82] discovered a positive correlation between calf circumference and muscle mass, prompting Wu et al. [47] to quantify calf circumference as a muscle mass indicator. Despite possible measurement errors in older adults due to skin elasticity loss and fat distribution changes, calf circumference remains an ideal, simple, and non-invasive tool for muscle mass assessment and sarcopenia screening in primary healthcare settings [83]. Additionally, a single study [42] utilizing adjusted BMI as a muscle mass assessment tool reported no correlation between the two conditions. However, reservations are expressed as BMI is not a mainstream muscle mass assessment tool, and limited research is available on this topic. Future studies should validate results using gold-standard methods like CT/MRI while considering clinical feasibility. Notably, the heterogeneity of sarcopenia diagnostic criteria—such as differing tools and cutoff values for muscle strength (e.g., varying grip dynamometers) and physical function (e.g., gait speed, chair-rise test)—introduces a risk of misclassification [84]. These discrepancies underscore the need to standardize sarcopenia definitions and measurement protocols to ensure consistency in research and clinical practice.

With the exception of the SPMSQ test, aggregated data from various cognitive assessment tools consistently demonstrated a significant relationship between the two diseases. A study [85] reported that the SPMSQ scale identified cognitive impairment in a mere 5.62% of cases, potentially due to its low level of difficulty. Huang et al. [35] suggested a specific correlation between sarcopenia and verbal fluency, while Kim et al. [37] identified its impact on processing speed and executive function in male patients. Due to the diverse dimensions assessed, the SPMSQ scale does not evaluate these specific aspects, potentially accounting for the absence of a significant correlation. However, since only one study utilized the SPMSQ for assessing cognitive function, additional research is essential to establish their efficacy.

Research findings indicate that women diagnosed with sarcopenia face a stronger association with cognitive impairment compared to men. The gender disparity in the sarcopenia-cognition association may stem from sex-specific hormonal and myokine regulation. Postmenopausal estrogen depletion disrupts its protective roles in musculoskeletal and neurological health [86,87]. This loss likely exacerbates both sarcopenia and cognitive decline. Additionally, postmenopausal women also experience a steeper decline in brain-derived neurotrophic factor (BDNF), essential for hippocampal neurogenesis and memory consolidation [88]. Estrogen-dependent BDNF depletion may further strengthen the sarcopenia-cognition association. Despite the ongoing debate regarding gender’s impact on sarcopenia and cognitive impairment, this study implies that sarcopenia poses a risk factor for both genders. Gender may lead to differential effects of sarcopenia on cognitive function. The association between sarcopenia and cognitive impairment appears stronger in Asia than in Europe and the Americas, while African studies display no significant correlation. Several factors contribute to this regional disparity. Firstly, Asia’s large older adult population [89] naturally increases the prevalence of age-related conditions like sarcopenia and cognitive decline. In our study, Asian participants had the highest average age, which may explain the stronger link between these conditions. Secondly, protein intake is crucial. While the recommended daily protein intake for healthy older adults is 1.0–1.2 g/kg, and 1.2–1.5 g/kg for those with acute or chronic diseases [90], Asian populations typically consume less [91]. This deficiency may contribute to higher rates of both sarcopenia and cognitive impairment in Asia. In contrast, the Mediterranean diet in Southern Europe, rich in anti-inflammatory foods, offers protection for both muscle function [92] and neurocognitive health [93]. Geographic and socioeconomic factors also play a role. Cognitive impairment is most prevalent in Asia and the Pacific, especially in middle- and high-income areas [94]. The lack of a significant link in Africa may stem from limited original research there. Studies highlight the challenges of accessing medical care in Africa and other low- and middle-income regions [95], where economic constraints hinder sarcopenia screening [33,52,96], leading to underreporting of its incidence and severity. Individuals with sarcopenia admitted to hospitals exhibited a stronger association with cognitive impairment in contrast to those residing in community settings. This may be related to the age of the study population, comorbidities, and different measurement tools. These findings underscore the necessity for hospitals to prioritize concerns related to the older adults and institute preventive strategies targeting sarcopenia among high-risk patients. Furthermore, it is recommended to conduct cognitive function assessments and interventions for individuals diagnosed with sarcopenia. It is imperative to educate and survey community residents promptly, particularly encouraging and monitoring middle-aged and older adults to undertake exercise and dietary interventions [97] for preventing and treating sarcopenia and cognitive impairment.

Our study, while robust, has several limitations that warrant discussion. Firstly, the geographical origins of the included studies are predominantly Asian, which limits the generalizability of our findings to other populations. Efforts should be made to include more diverse cohorts in future studies to better represent global demographics. Secondly, although our meta-analysis trim-and-filling method indicated that the results are robust despite publication bias, this bias still raises concerns about the comprehensiveness of the evidence base. We have taken steps to mitigate this by including studies that, while primarily investigating the relationship between sarcopenic obesity and osteoporosis with cognitive impairment, provided separate statistical values for sarcopenia and cognitive impairment. The consistency of our sensitivity analyses after excluding these studies further attests to the stability of our findings. However, the predominance of cross-sectional studies in our synthesis restricts our ability to infer causality. This limitation highlights an urgent need for additional longitudinal and interventional studies to clarify the temporal sequence and nature of the relationship between sarcopenia and cognitive decline. Our study focused solely on effect sizes where cognitive impairment served as the outcome, omitting analyses of studies using changes in cognitive scores as indicators. This choice may limit our understanding of the dynamic nature of cognitive function changes. Future research should prioritize studies using cognitive scores as primary outcomes to comprehensively assess the relationship between sarcopenia and cognitive decline. Furthermore, data limitations in primary studies prevented a detailed exploration of how socioeconomic and lifestyle factors influence the association between these two conditions. While our meta-analysis incorporated adjusted ORs that accounted for these factors, their role as potential confounders or effect modifiers could not be fully elucidated. Future research should systematically analyze these variables to better understand their role in this relationship. Finally, the heterogeneity in study populations, sarcopenia assessment tools, and cognitive impairment evaluations introduced variability, complicating the interpretation of a unified relationship. Despite observed associations, the diversity in methodologies hinders a definitive conclusion regarding the temporality and nature of the relationship between sarcopenia and cognitive impairment. It is imperative to conduct more harmonized and standardized studies to provide a robust evidence base for developing preventive and therapeutic strategies.

Future research must prioritize additional longitudinal and neuroimaging studies to delve into the underlying mechanisms of sarcopenia and cognitive impairment. Such investigations are critical for devising more precise and efficacious strategies for prevention and treatment. Integrating interventions for possible sarcopenia with those for cognitive impairment could enhance the efficiency of medical resource utilization among the older adults. Our findings suggest that individuals with possible sarcopenia should be vigilant. However, there is a dearth of intervention studies focusing on such patients [98]. Currently, there is no established cure for sarcopenia, but exercise intervention is recognized as a vital method to prevent the deterioration of physical function. Resistance exercise, a key component of exercise intervention, has been shown to improve muscle mass and function in patients with sarcopenia [99] and is effective in enhancing cognitive function in older adults [100] and those with dementia [101]. Yet, research on the impact of exercise interventions on cognitive function in individuals with sarcopenia is scarce. Future research should explore the synergistic benefits of aerobic, resistance, and cognitive training to formulate effective interventions targeting cognitive function in individuals with sarcopenia. Concurrently, intervention studies are needed to determine whether improvements in sarcopenia can effectively reduce the incidence of cognitive impairment.

## Conclusions

In conclusion, our findings highlight that individuals with possible sarcopenia or sarcopenia exhibit a significantly higher likelihood of cognitive impairment compared to the general population. Healthcare professionals should prioritize early cognitive screening and intervention for individuals with sarcopenia, with special consideration for older adults, females, and hospitalized patients. Additional brain imaging and longitudinal studies are imperative to clarify the relationship between sarcopenia and cognitive impairment. Furthermore, interventional studies are necessary to investigate whether enhancements in sarcopenia can prevent cognitive impairment.

## Supporting information

S1 Data PRISMA checklist. (DOCX)

S2 Data Studies characteristics and analysis. (DOCX)

S3 Data Studies included and excluded. (DOCX)

S4 Data The data supporting the findings. (XLSX)
