# Supplementary material for: The association of sarcopenia, possible sarcopenia and cognitive impairment: A systematic review and meta-analysis
Source: PLoS One. 2025 May 28;20(5):e0324258. doi: 10.1371/journal.pone.0324258 (PMC12118908; doi:10.1371/journal.pone.0324258)
Supplement: S2 Data — (DOCX) [file pone.0324258.s002.docx]

## The association of sarcopenia, possible sarcopenia and cognitive impairment: a systematic review and meta-analysis

**Index**

**Tables**

- S1 Table: Search strategy
- S2 Table: Detailed characteristics of included studies
- S3 Table: Risk of bias assessment results for included cross-sectional studies
- S4 Table: Risk of bias assessment results for included cohort studies

**Figures**

- S1 Fig: Forest plot of the association between sarcopenia and cognitive impairment (Crude OR)
- S2 Fig: Funnel plot of the association between sarcopenia and cognitive impairment (Adjusted OR)
- S3 Fig: Funnel plot of the association between sarcopenia and cognitive impairment (Crude OR)
- S4 Fig: Trim and fill analysis of the association between sarcopenia and cognitive impairment (Adjusted OR)
- S5 Fig: Sensitivity analysis of adjusted OR studies excluding comorbidities
- S6 Fig: Sensitivity analysis of crude OR studies excluding comorbidities
- S7 Fig: Sensitivity analysis of studies providing adjusted OR
- S8 Fig: Sensitivity analysis of studies providing crude OR
- S9 Fig: Forest plot of the association between possible sarcopenia and cognitive impairment (Adjusted OR)
- S10 Fig: Forest plot of the association between possible sarcopenia and cognitive impairment (Crude OR)
- S11 Fig: Sensitivity analysis of the association between possible sarcopenia and cognitive impairment using adjusted OR
- S12 Fig: Sensitivity analysis of the association between possible sarcopenia and cognitive impairment using crude OR

**S1 Table. Search strategy.**

| **Database** | **Results** | **Search terms** |
| --- | --- | --- |
| **PubMed** | *Search Date:*  *December 2, 2023*  754 | Search: ((((((((((Sarcopenia[MeSH Terms]) OR (Sarcopenias[Title/Abstract])) OR (skeletal muscle[Title/Abstract])) OR (muscle mass[Title/Abstract])) OR (muscle strength[Title/Abstract])) OR (physical performance[Title/Abstract])) OR (gait speed[Title/Abstract])) OR (hyposthenia[Title/Abstract])) OR (grip strength[Title/Abstract]) AND (ffrft[Filter])) AND ((((((((((((((((((((((((((((((Cognitive Dysfunction[MeSH Terms]) OR (Cognitive Dysfunctions[Title/Abstract])) OR (Dysfunction, Cognitive[Title/Abstract])) OR (Dysfunctions, Cognitive[Title/Abstract])) OR (Cognitive Impairments[Title/Abstract])) OR (Cognitive Impairment[Title/Abstract])) OR (Impairment, Cognitive[Title/Abstract])) OR (Impairments, Cognitive[Title/Abstract])) OR (Cognitive Disorder[Title/Abstract])) OR (Cognitive Disorders[Title/Abstract])) OR (Disorder, Cognitive[Title/Abstract])) OR (Disorders, Cognitive[Title/Abstract])) OR (Mild Cognitive Impairment[Title/Abstract])) OR (Cognitive Impairment, Mild[Title/Abstract])) OR (Cognitive Impairments, Mild[Title/Abstract])) OR (Impairment, Mild Cognitive[Title/Abstract])) OR (Impairments, Mild Cognitive[Title/Abstract])) OR (Mild Cognitive Impairments[Title/Abstract])) OR (Cognitive Decline[Title/Abstract])) OR (Cognitive Declines[Title/Abstract])) OR (Decline, Cognitive[Title/Abstract])) OR (Declines, Cognitive[Title/Abstract])) OR (Mental Deterioration[Title/Abstract])) OR (Deterioration, Mental[Title/Abstract])) OR (Deteriorations, Mental[Title/Abstract])) OR (Mental Deteriorations[Title/Abstract])) OR (Cognition[Title/Abstract])) OR (Cognitive function[Title/Abstract]) AND (ffrft[Filter])) OR ((Dementia[MeSH Terms]) OR (Dementias[Title/Abstract]) AND (ffrft[Filter]))) OR (((((((((((((((((((((((((Alzheimer Disease[MeSH Terms]) OR (Alzheimer Dementia[Title/Abstract])) OR (Alzheimer Dementias[Title/Abstract])) OR (Dementia, Alzheimer[Title/Abstract])) OR (Alzheimer's Disease[Title/Abstract])) OR (Dementia, Senile[Title/Abstract])) OR (Senile Dementia[Title/Abstract])) OR (Dementia, Alzheimer Type[Title/Abstract])) OR (Alzheimer Type Dementia[Title/Abstract])) OR (Alzheimer Type Senile Dementia[Title/Abstract])) OR (Primary Senile Degenerative Dementia[Title/Abstract])) OR (Dementia, Primary Senile Degenerative[Title/Abstract])) OR (Alzheimer Sclerosis[Title/Abstract])) OR (Sclerosis, Alzheimer[Title/Abstract])) OR (Alzheimer Syndrome[Title/Abstract])) OR (Alzheimer's Diseases[Title/Abstract])) OR (Alzheimer Diseases[Title/Abstract])) OR (Alzheimers Diseases[Title/Abstract])) OR (Senile Dementia, Alzheimer Type[Title/Abstract])) OR (Dementia, Presenile[Title/Abstract])) OR (Presenile Dementia[Title/Abstract])) OR (Alzheimer Disease, Late Onset[Title/Abstract])) OR (Late Onset Alzheimer Disease[Title/Abstract])) OR (Alzheimer's Disease, Focal Onset[Title/Abstract])) OR (Focal Onset Alzheimer's Disease[Title/Abstract]) AND (ffrft[Filter])) AND (ffrft[Filter]))) AND ((((((((((((((((((((((Cohort Studies[MeSH Terms]) OR (Cohort Study[Title/Abstract])) OR (Studies, Cohort[Title/Abstract])) OR (Study, Cohort[Title/Abstract])) OR (Longitudinal Studies[Title/Abstract])) OR (Longitudinal Study[Title/Abstract])) OR (Studies, Longitudinal[Title/Abstract])) OR (Prospective Studies[Title/Abstract])) OR (Prospective Study[Title/Abstract])) OR (Studies, Prospective[Title/Abstract])) OR (Study, Prospective[Title/Abstract])) OR (Case-Control Studies[Title/Abstract])) OR (Cross-Sectional Studies[Title/Abstract])) OR (Prevalence Studies[Title/Abstract])) OR (Prevalence Study[Title/Abstract])) OR (Studies, Prevalence[Title/Abstract])) OR (Study, Prevalence[Title/Abstract])) OR (Cross Sectional Studies[Title/Abstract])) OR (Cross-Sectional Study[Title/Abstract])) OR (Studies, Cross-Sectional[Title/Abstract])) OR (Study, Cross-Sectional[Title/Abstract])) OR (Cross Sectional Analysis[Title/Abstract]) AND (ffrft[Filter])) Sort by: Most Recent |
|  | *Search Date: March 14, 2025*  261 | ((((((((((Sarcopenia[MeSH Terms]) OR (Sarcopenias[Title/Abstract])) OR (skeletal muscle[Title/Abstract])) OR (muscle mass[Title/Abstract])) OR (muscle strength[Title/Abstract])) OR (physical performance[Title/Abstract])) OR (gait speed[Title/Abstract])) OR (hyposthenia[Title/Abstract])) OR (grip strength[Title/Abstract])) AND ((((((((((((((((((((((((((((((Cognitive Dysfunction[MeSH Terms]) OR (Cognitive Dysfunctions[Title/Abstract])) OR (Dysfunction, Cognitive[Title/Abstract])) OR (Dysfunctions, Cognitive[Title/Abstract])) OR (Cognitive Impairments[Title/Abstract])) OR (Cognitive Impairment[Title/Abstract])) OR (Impairment, Cognitive[Title/Abstract])) OR (Impairments, Cognitive[Title/Abstract])) OR (Cognitive Disorder[Title/Abstract])) OR (Cognitive Disorders[Title/Abstract])) OR (Disorder, Cognitive[Title/Abstract])) OR (Disorders, Cognitive[Title/Abstract])) OR (Mild Cognitive Impairment[Title/Abstract])) OR (Cognitive Impairment, Mild[Title/Abstract])) OR (Cognitive Impairments, Mild[Title/Abstract])) OR (Impairment, Mild Cognitive[Title/Abstract])) OR (Impairments, Mild Cognitive[Title/Abstract])) OR (Mild Cognitive Impairments[Title/Abstract])) OR (Cognitive Decline[Title/Abstract])) OR (Cognitive Declines[Title/Abstract])) OR (Decline, Cognitive[Title/Abstract])) OR (Declines, Cognitive[Title/Abstract])) OR (Mental Deterioration[Title/Abstract])) OR (Deterioration, Mental[Title/Abstract])) OR (Deteriorations, Mental[Title/Abstract])) OR (Mental Deteriorations[Title/Abstract])) OR (Cognition[Title/Abstract])) OR (Cognitive function[Title/Abstract])) OR ((Dementia[MeSH Terms]) OR (Dementias[Title/Abstract]))) OR (((((((((((((((((((((((((Alzheimer Disease[MeSH Terms]) OR (Alzheimer Dementia[Title/Abstract])) OR (Alzheimer Dementias[Title/Abstract])) OR (Dementia, Alzheimer[Title/Abstract])) OR (Alzheimer's Disease[Title/Abstract])) OR (Dementia, Senile[Title/Abstract])) OR (Senile Dementia[Title/Abstract])) OR (Dementia, Alzheimer Type[Title/Abstract])) OR (Alzheimer Type Dementia[Title/Abstract])) OR (Alzheimer Type Senile Dementia[Title/Abstract])) OR (Primary Senile Degenerative Dementia[Title/Abstract])) OR (Dementia, Primary Senile Degenerative[Title/Abstract])) OR (Alzheimer Sclerosis[Title/Abstract])) OR (Sclerosis, Alzheimer[Title/Abstract])) OR (Alzheimer Syndrome[Title/Abstract])) OR (Alzheimer's Diseases[Title/Abstract])) OR (Alzheimer Diseases[Title/Abstract])) OR (Alzheimers Diseases[Title/Abstract])) OR (Senile Dementia, Alzheimer Type[Title/Abstract])) OR (Dementia, Presenile[Title/Abstract])) OR (Presenile Dementia[Title/Abstract])) OR (Alzheimer Disease, Late Onset[Title/Abstract])) OR (Late Onset Alzheimer Disease[Title/Abstract])) OR (Alzheimer's Disease, Focal Onset[Title/Abstract])) OR (Focal Onset Alzheimer's Disease[Title/Abstract])))) AND ((((((((((((((((((((((Cohort Studies[MeSH Terms]) OR (Cohort Study[Title/Abstract])) OR (Studies, Cohort[Title/Abstract])) OR (Study, Cohort[Title/Abstract])) OR (Longitudinal Studies[Title/Abstract])) OR (Longitudinal Study[Title/Abstract])) OR (Studies, Longitudinal[Title/Abstract])) OR (Prospective Studies[Title/Abstract])) OR (Prospective Study[Title/Abstract])) OR (Studies, Prospective[Title/Abstract])) OR (Study, Prospective[Title/Abstract])) OR (Case-Control Studies[Title/Abstract])) OR (Cross-Sectional Studies[Title/Abstract])) OR (Prevalence Studies[Title/Abstract])) OR (Prevalence Study[Title/Abstract])) OR (Studies, Prevalence[Title/Abstract])) OR (Study, Prevalence[Title/Abstract])) OR (Cross Sectional Studies[Title/Abstract])) OR (Cross-Sectional Study[Title/Abstract])) OR (Studies, Cross-Sectional[Title/Abstract])) OR (Study, Cross-Sectional[Title/Abstract])) OR (Cross Sectional Analysis[Title/Abstract])) Filters: from 2023/12/2 - 2025/3/14 |
| **Cochrane** | *Search Date:*  *December 2, 2023*  1043 | #1 Sarcopenia  #2 (Sarcopenias):ab,ti,kw OR (skeletal muscle):ab,ti,kw OR (muscle mass):ab,ti,kw OR (muscle strength):ab,ti,kw OR (physical performance):ab,ti,kw OR (gait speed):ab,ti,kw OR (Hyposthenia):ab,ti,kw OR (grip strength):ab,ti,kw  #3 Cognitive Dysfunction  #4 (Cognitive Dysfunctions):ab,ti,kw OR (Dysfunction, Cognitive):ab,ti,kw OR (Dysfunctions, Cognitive):ab,ti,kw OR (Cognitive Impairments):ab,ti,kw OR (Cognitive Impairment):ab,ti,kw OR (Impairment, Cognitive):ab,ti,kw OR (Impairments, Cognitive):ab,ti,kw OR (Cognitive Disorder):ab,ti,kw OR (Cognitive Disorders):ab,ti,kw OR (Disorder, Cognitive):ab,ti,kw OR (Disorders, Cognitive):ab,ti,kw OR (Mild Cognitive Impairment):ab,ti,kw OR (Cognitive Impairment, Mild):ab,ti,kw OR (Cognitive Impairments, Mild):ab,ti,kw OR (Impairment, Mild Cognitive):ab,ti,kw OR (Impairments, Mild Cognitive):ab,ti,kw OR (Mild Cognitive Impairments):ab,ti,kw OR (Cognitive Decline):ab,ti,kw OR (Cognitive Declines):ab,ti,kw OR (Decline, Cognitive):ab,ti,kw OR (Declines, Cognitive):ab,ti,kw OR (Mental Deterioration):ab,ti,kw OR (Deterioration, Mental):ab,ti,kw OR (Deteriorations, Mental):ab,ti,kw OR (Mental Deteriorations):ab,ti,kw OR (Cognition):ab,ti,kw OR (Cognitive function):ab,ti,kw  #5 Dementia  #6 (Dementias):ab,ti,kw  #7 Alzheimer Disease  #8 (Alzheimer Dementia):ab,ti,kw OR (Alzheimer Dementias):ab,ti,kw OR (Dementia, Alzheimer):ab,ti,kw OR (Alzheimer's Disease):ab,ti,kw OR (Dementia, Senile):ab,ti,kw OR (Senile Dementia):ab,ti,kw OR (Dementia, Alzheimer Type):ab,ti,kw OR (Alzheimer Type Dementia):ab,ti,kw OR (Alzheimer Type Senile Dementia):ab,ti,kw OR (Primary Senile Degenerative Dementia):ab,ti,kw OR (Dementia, Primary Senile Degenerative):ab,ti,kw OR (Alzheimer Sclerosis):ab,ti,kw OR (Sclerosis, Alzheimer):ab,ti,kw OR (Alzheimer Syndrome):ab,ti,kw OR (Alzheimer's Diseases):ab,ti,kw OR (Alzheimer Diseases):ab,ti,kw OR (Alzheimers Diseases):ab,ti,kw OR (Senile Dementia, Alzheimer Type):ab,ti,kw OR (Dementia, Presenile):ab,ti,kw OR (Presenile Dementia):ab,ti,kw OR (Alzheimer Disease, Late Onset):ab,ti,kw OR (Late Onset Alzheimer Disease):ab,ti,kw OR (Alzheimer's Disease, Focal Onset):ab,ti,kw OR (Focal Onset Alzheimer's Disease):ab,ti,kw  #9 Cohort Studies  #10 (Cohort Study):ab,ti,kw OR (Studies, Cohort):ab,ti,kw OR (Study, Cohort):ab,ti,kw OR (Longitudinal Studies):ab,ti,kw OR (Longitudinal Study):ab,ti,kw OR (Studies, Longitudinal):ab,ti,kw OR (Prospective Studies):ab,ti,kw OR (Prospective Study):ab,ti,kw OR (Studies, Prospective):ab,ti,kw OR (Study, Prospective):ab,ti,kw OR (Case-Control Studies):ab,ti,kw OR (Cross-Sectional Studies):ab,ti,kw OR (Prevalence Studies):ab,ti,kw OR (Prevalence Study):ab,ti,kw OR (Studies, Prevalence):ab,ti,kw OR (Study, Prevalence):ab,ti,kw OR (Cross Sectional Studies):ab,ti,kw OR (Cross-Sectional Study):ab,ti,kw OR (Studies, Cross-Sectional):ab,ti,kw OR (Study, Cross-Sectional):ab,ti,kw OR (Cross Sectional Analysis):ab,ti,kw  #11 #1 OR #2  #12 #3 OR #4 OR #5 OR #6 OR #7 OR #8  #13 #9 OR #10  #14 #11 AND #12 AND #13 |
|  | *Search Date: March 14, 2025*  138 | Use the same search terms above  Filters: from 2023/12/2-2025/3/14 |
| **WOS** | *Search Date:*  *December 2, 2023*  772 | **#1 TS=(sarcopenia OR sarcopenias ) and Preprint Citation Index (Exclude–Database)**  **#2 TS=(Cognitive Dysfunction OR Cognitive Dysfunctions OR Dysfunction, Cognitive OR Dysfunctions, Cognitive OR Cognitive Impairments OR Cognitive Impairment OR Impairment, Cognitive OR Impairments, Cognitive OR Cognitive Disorder OR Cognitive Disorders OR Disorder, Cognitive OR Disorders, Cognitive OR Mild Cognitive Impairment OR Cognitive Impairment, Mild OR Cognitive Impairments, Mild OR Impairment, Mild Cognitive OR Impairments, Mild Cognitive OR Mild Cognitive Impairments OR Cognitive Decline OR Cognitive Declines OR Decline, Cognitive OR Declines, Cognitive OR Mental Deterioration OR Deterioration, Mental OR Deteriorations, Mental OR Mental Deteriorations OR Cognition OR Cognitive function OR Dementia OR Dementias OR Alzheimer Disease OR Alzheimer Dementia OR Alzheimer Dementias OR Dementia, Alzheimer OR Alzheimer's Disease OR Dementia, Senile OR Senile Dementia OR Dementia, Alzheimer Type OR Alzheimer Type Dementia OR Alzheimer Type Senile Dementia OR Primary Senile Degenerative Dementia OR Dementia, Primary Senile Degenerative OR Alzheimer Sclerosis OR Sclerosis, Alzheimer OR Alzheimer Syndrome OR Alzheimer's Diseases OR Alzheimer Diseases OR Alzheimers Diseases OR Senile Dementia, Alzheimer Type OR Dementia, Presenile OR Presenile Dementia OR Alzheimer Disease, Late Onset OR Late Onset Alzheimer Disease OR Alzheimer's Disease, Focal Onset OR Focal Onset Alzheimer's Disease) and Preprint Citation Index (Exclude – Database)**  **#3 TS=(Cohort Studies OR Cohort Study OR Studies, Cohort OR Study, Cohort OR Longitudinal Studies OR Longitudinal Study OR Studies, Longitudinal OR Prospective Studies OR Prospective Study OR Studies, Prospective OR Study, Prospective OR Case-Control Studies OR Cross-Sectional Studies OR Prevalence Studies OR Prevalence Study OR Studies, Prevalence OR Study, Prevalence OR Cross Sectional Studies OR Cross-Sectional Study OR Studies, Cross-Sectional OR Study, Cross-Sectional OR Cross Sectional Analysis) and Preprint Citation Index (Exclude – Database)**  **#4 #1 AND #2 AND #3** |
|  | *Search Date:*  *March 14, 2025*  158 | **#1 TS=(sarcopenia OR sarcopenias ) and Preprint Citation Index (Exclude–Database)**  **#2 TS=(Cognitive Dysfunction OR Cognitive Dysfunctions OR Dysfunction, Cognitive OR Dysfunctions, Cognitive OR Cognitive Impairments OR Cognitive Impairment OR Impairment, Cognitive OR Impairments, Cognitive OR Cognitive Disorder OR Cognitive Disorders OR Disorder, Cognitive OR Disorders, Cognitive OR Mild Cognitive Impairment OR Cognitive Impairment, Mild OR Cognitive Impairments, Mild OR Impairment, Mild Cognitive OR Impairments, Mild Cognitive OR Mild Cognitive Impairments OR Cognitive Decline OR Cognitive Declines OR Decline, Cognitive OR Declines, Cognitive OR Mental Deterioration OR Deterioration, Mental OR Deteriorations, Mental OR Mental Deteriorations OR Cognition OR Cognitive function OR Dementia OR Dementias OR Alzheimer Disease OR Alzheimer Dementia OR Alzheimer Dementias OR Dementia, Alzheimer OR Alzheimer's Disease OR Dementia, Senile OR Senile Dementia OR Dementia, Alzheimer Type OR Alzheimer Type Dementia OR Alzheimer Type Senile Dementia OR Primary Senile Degenerative Dementia OR Dementia, Primary Senile Degenerative OR Alzheimer Sclerosis OR Sclerosis, Alzheimer OR Alzheimer Syndrome OR Alzheimer's Diseases OR Alzheimer Diseases OR Alzheimers Diseases OR Senile Dementia, Alzheimer Type OR Dementia, Presenile OR Presenile Dementia OR Alzheimer Disease, Late Onset OR Late Onset Alzheimer Disease OR Alzheimer's Disease, Focal Onset OR Focal Onset Alzheimer's Disease) and Preprint Citation Index (Exclude – Database)**  **#3 TS=(Cohort Studies OR Cohort Study OR Studies, Cohort OR Study, Cohort OR Longitudinal Studies OR Longitudinal Study OR Studies, Longitudinal OR Prospective Studies OR Prospective Study OR Studies, Prospective OR Study, Prospective OR Case-Control Studies OR Cross-Sectional Studies OR Prevalence Studies OR Prevalence Study OR Studies, Prevalence OR Study, Prevalence OR Cross Sectional Studies OR Cross-Sectional Study OR Studies, Cross-Sectional OR Study, Cross-Sectional OR Cross Sectional Analysis) and Preprint Citation Index (Exclude – Database)**  **#4 #1 AND #2 AND #3 and Preprint Citation Index (Exclude – Database) and 2024 or 2025 (Publication Years) (n=138)**  **2.** Manual search using the same strategy in web of science 2023.12.2-2023.12.31 （n=20） |
| **EmBase** | *Search Date:*  *December 2, 2023*  1656 | #1. 'sarcopenia'/exp OR sarcopenia  #2. 'sarcopenias':ab,ti OR 'skeletal muscle':ab,ti OR 'muscle mass':ab,ti OR 'muscle strength':ab,ti OR 'physical performance':ab,ti OR 'gait speed':ab,ti OR 'hyposthenia':ab,ti OR 'grip strength':ab,ti  #3. #1 OR #2  #4. cognitive AND dysfunction  #5. 'cognitive dysfunctions':ab,ti OR 'dysfunction,cognitive':ab,ti OR 'dysfunctions,cognitive':ab,ti OR 'cognitive impairments':ab,ti OR 'cognitive impairment':ab,ti OR 'impairment, cognitive':ab,ti OR 'impairments, cognitive':ab,ti OR 'cognitive disorder':ab,ti OR 'cognitive disorders':ab,ti OR 'disorder, cognitive':ab,ti OR 'disorders, cognitive':ab,ti OR 'mild cognitive impairment':ab,ti OR 'cognitive impairment, mild':ab,ti OR 'cognitive impairments, mild':ab,ti OR 'impairment, mild cognitive':ab,ti OR 'impairments, mild cognitive':ab,ti OR 'mild cognitive impairments':ab,ti OR 'cognitive decline':ab,ti OR 'cognitive declines':ab,ti OR 'decline,cognitive':ab,ti OR 'declines, cognitive':ab,ti OR 'mental deterioration':ab,ti OR 'deterioration, mental':ab,ti OR 'deteriorations,mental':ab,ti OR 'mental deteriorations':ab,ti OR 'cognition':ab,ti OR 'cognitive function':ab,ti  #6. dementia  #7.'dementias':ab,ti  #8. alzheimer AND disease  #9. 'alzheimer dementia':ab,ti OR 'alzheimer dementias':ab,ti OR 'dementia, alzheimer':ab,ti OR 'alzheimers disease':ab,ti OR 'dementia, senile':ab,ti OR 'senile dementia':ab,ti OR 'dementia, alzheimer type':ab,ti OR 'alzheimer type dementia':ab,ti OR 'alzheimer type senile dementia':ab,ti OR 'primary senile degenerative dementia':ab,ti OR 'dementia, primary senile degenerative':ab,ti OR 'alzheimer sclerosis':ab,ti OR 'sclerosis, alzheimer':ab,ti OR 'alzheimer syndrome':ab,ti OR 'alzheimer diseases':ab,ti OR 'alzheimers diseases':ab,ti OR 'senile dementia, alzheimer type':ab,ti OR 'dementia, presenile':ab,ti OR 'presenile dementia':ab,ti OR 'alzheimer disease, late onset':ab,ti OR 'late onset alzheimer disease':ab,ti OR 'alzheimers disease, focal onset':ab,ti OR 'focal onset alzheimers disease':ab,ti  #10. #4 OR #5 OR #6 OR #7 OR #9  #11. cohort AND studies  #12. 'cohort study':ab,ti OR 'studies, cohort':ab,ti OR 'study, cohort':ab,ti OR 'longitudinal studies':ab,ti OR 'longitudinal study':ab,ti OR 'studies, longitudinal':ab,ti OR 'prospective studies':ab,ti OR 'prospective study':ab,ti OR 'studies, prospective':ab,ti OR 'study, prospective':ab,ti OR 'case-control studies':ab,ti OR 'cross-sectional studies':ab,ti OR 'prevalence studies':ab,ti OR 'prevalence study':ab,ti OR 'studies, prevalence':ab,ti OR 'study, prevalence':ab,ti OR 'cross sectional studies':ab,ti OR 'cross-sectional study':ab,ti OR 'studies, cross-sectional':ab,ti OR 'study, cross-sectional':ab,ti OR 'cross sectional analysis':ab,ti  #13 #11 OR #12  #14 #3 AND #10 AND #13 |
|  | *Search Date:*  *March 14, 2025*  304 | 1. #1. 'sarcopenia'/exp OR sarcopenia  #2. 'sarcopenias':ab,ti OR 'skeletal muscle':ab,ti OR 'muscle mass':ab,ti OR 'muscle strength':ab,ti OR 'physical performance':ab,ti OR 'gait speed':ab,ti OR 'hyposthenia':ab,ti OR 'grip strength':ab,ti  #3. #1 OR #2  #4. cognitive AND dysfunction  #5. 'cognitive dysfunctions':ab,ti OR 'dysfunction,cognitive':ab,ti OR 'dysfunctions,cognitive':ab,ti OR 'cognitive impairments':ab,ti OR 'cognitive impairment':ab,ti OR 'impairment, cognitive':ab,ti OR 'impairments, cognitive':ab,ti OR 'cognitive disorder':ab,ti OR 'cognitive disorders':ab,ti OR 'disorder, cognitive':ab,ti OR 'disorders, cognitive':ab,ti OR 'mild cognitive impairment':ab,ti OR 'cognitive impairment, mild':ab,ti OR 'cognitive impairments, mild':ab,ti OR 'impairment, mild cognitive':ab,ti OR 'impairments, mild cognitive':ab,ti OR 'mild cognitive impairments':ab,ti OR 'cognitive decline':ab,ti OR 'cognitive declines':ab,ti OR 'decline,cognitive':ab,ti OR 'declines, cognitive':ab,ti OR 'mental deterioration':ab,ti OR 'deterioration, mental':ab,ti OR 'deteriorations,mental':ab,ti OR 'mental deteriorations':ab,ti OR 'cognition':ab,ti OR 'cognitive function':ab,ti  #6. 'dementia'/exp OR dementia  #7.'dementias':ab,ti  #8. alzheimer AND disease  #9. 'alzheimer dementia':ab,ti OR 'alzheimer dementias':ab,ti OR 'dementia, alzheimer':ab,ti OR 'alzheimers disease':ab,ti OR 'dementia, senile':ab,ti OR 'senile dementia':ab,ti OR 'dementia, alzheimer type':ab,ti OR 'alzheimer type dementia':ab,ti OR 'alzheimer type senile dementia':ab,ti OR 'primary senile degenerative dementia':ab,ti OR 'dementia, primary senile degenerative':ab,ti OR 'alzheimer sclerosis':ab,ti OR 'sclerosis, alzheimer':ab,ti OR 'alzheimer syndrome':ab,ti OR 'alzheimer diseases':ab,ti OR 'alzheimers diseases':ab,ti OR 'senile dementia, alzheimer type':ab,ti OR 'dementia, presenile':ab,ti OR 'presenile dementia':ab,ti OR 'alzheimer disease, late onset':ab,ti OR 'late onset alzheimer disease':ab,ti OR 'alzheimers disease, focal onset':ab,ti OR 'focal onset alzheimers disease':ab,ti  #10. #4 OR #5 OR #6 OR #7 OR #9  #11. cohort AND studies  #12. 'cohort study':ab,ti OR 'studies, cohort':ab,ti OR 'study, cohort':ab,ti OR 'longitudinal studies':ab,ti OR 'longitudinal study':ab,ti OR 'studies, longitudinal':ab,ti OR 'prospective studies':ab,ti OR 'prospective study':ab,ti OR 'studies, prospective':ab,ti OR 'study, prospective':ab,ti OR 'case-control studies':ab,ti OR 'cross-sectional studies':ab,ti OR 'prevalence studies':ab,ti OR 'prevalence study':ab,ti OR 'studies, prevalence':ab,ti OR 'study, prevalence':ab,ti OR 'cross sectional studies':ab,ti OR 'cross-sectional study':ab,ti OR 'studies, cross-sectional':ab,ti OR 'study, cross-sectional':ab,ti OR 'cross sectional analysis':ab,ti  #13 #11 OR #12  #14 #3 AND #10 AND #13  #15 #14 AND (2024:py OR 2025:py) (n=304)  2. Manual search using the same strategy in Embase 2023.12.2-2023.12.31 （n=0） |
| **CNKI** | *Search Date:*  *December 2, 2023*  93 | （主题：肌减少症） OR （主题：肌少症） OR （主题：肌肉衰减综合征） OR （主题：肌肉衰减） OR （主题：肌肉减少症） OR （主题：骨骼肌减少症） OR （主题：少肌症） AND （主题：认知功能障碍） OR （主题：认知减退） OR （主题：认知损害） OR （主题：认知障碍） OR （主题：认知功能） OR （主题：阿尔茨海默病） OR （主题：阿尔茨海默症） OR （主题：痴呆） OR （主题：老年痴呆） |
|  | *Search Date:*  *March 14, 2025*  36 | Use the same search terms above  Time frame - Publication time：2023/12/2-2025/3/14 |
| **Wanfang Databases** | *Search Date:*  *December 2, 2023*  1787 | (主题:(肌减少症 OR 肌少症 OR 肌肉衰减综合征 OR 肌肉衰减 OR 肌肉减少症 OR 骨骼肌减少症 OR 少肌症) and 主题:(认知功能障碍 OR 认知减退 OR 认知损害 OR 认知障碍 OR 认知功能 OR 阿尔茨海默病 OR 阿尔茨海默症 OR 痴呆 OR 老年痴呆)) |
|  | *Search Date:*  *March 14, 2025*  135 | 1.(主题:(肌减少症 OR 肌少症 OR 肌肉衰减综合征 OR 肌肉衰减 OR 肌肉减少症 OR 骨骼肌减少症 OR 少肌症) and 主题:(认知功能障碍 OR 认知减退 OR 认知损害 OR 认知障碍 OR 认知功能 OR 阿尔茨海默病 OR 阿尔茨海默症 OR 痴呆 OR 老年痴呆)) and 发表时间:2024-* （n=130）  2. Manual search using the same strategy in Wanfang Databases 2023.12.2–2023.12.31 （n=5） |
| **VIP** | *Search Date:*  *December 2, 2023*  47 | ((((((题名或关键词=肌减少症 OR 题名或关键词=肌肉衰减综合征) OR 题名或关键词=肌肉衰减) OR 题名或关键词=肌少症) OR 题名或关键词=肌肉减少症) OR 题名或关键词=骨骼肌减少症) OR 题名或关键词=少肌症) AND ((((((((题名或关键词=认知功能障碍 OR 题名或关键词=认知减退) OR 题名或关键词=认知损害) OR 题名或关键词=认知障碍) OR 题名或关键词=认知功能) OR 题名或关键词=阿尔茨海默病) OR 题名或关键词=阿尔茨海默症) OR 题名或关键词=痴呆) OR 题名或关键词=老年痴呆)) |
|  | *Search Date:*  *March 14, 2025*  17 | 1.(((((((题名或关键词=肌减少症 OR 题名或关键词=肌肉衰减综合征) OR 题名或关键词=肌肉衰减) OR 题名或关键词=肌少症) OR 题名或关键词=肌肉减少症) OR 题名或关键词=骨骼肌减少症) OR 题名或关键词=少肌症) AND ((((((((题名或关键词=认知功能障碍 OR 题名或关键词=认知减退) OR 题名或关键词=认知损害) OR 题名或关键词=认知障碍) OR 题名或关键词=认知功能) OR 题名或关键词=阿尔茨海默病) OR 题名或关键词=阿尔茨海默症) OR 题名或关键词=痴呆) OR 题名或关键词=老年痴呆)) AND (years:[2024 TO 2025]) （n=15）  2. Manual search using the same strategy in VIP 2023.12.2–2023.12.31 （n=2） |
| **SinoMed** | *Search Date:*  *December 2, 2023*  118 | #1 "肌减少症"[不加权:扩展]  #2 ("肌少症"[常用字段:智能] OR "肌肉衰减综合征"[常用字段:智能] OR "肌肉衰减"[常用字段:智能] OR "肌肉减少症"[常用字段:智能] OR "骨骼肌减少症"[常用字段:智能] OR "少肌症"[常用字段:智能]) AND -2023[日期]  #3 "认知功能障碍"[不加权:扩展]  #4 ("认知减退"[常用字段:智能] OR "认知损害"[常用字段:智能] OR "认知障碍"[常用字段:智能] OR "认知功能"[常用字段:智能] OR "阿尔茨海默病"[常用字段:智能] OR "阿尔茨海默症"[常用字段:智能] OR "痴呆"[常用字段:智能] OR "老年痴呆"[常用字段:智能]) AND -2023[日期]  #5" (#2) OR (#1)  #6" (#4) OR (#3)  #7" (#6) AND (#5) |
|  | *Search Date:*  *March 14, 2025*  46 | 1.#1 "肌减少症"[不加权:扩展]  #2 ("肌少症"[常用字段:智能] OR "肌肉衰减综合征"[常用字段:智能] OR "肌肉衰减"[常用字段:智能] OR "肌肉减少症"[常用字段:智能] OR "骨骼肌减少症"[常用字段:智能] OR "少肌症"[常用字段:智能]) AND 2024-2025[日期]  #3 "认知功能障碍"[不加权:扩展]  #4 ("认知减退"[常用字段:智能] OR "认知损害"[常用字段:智能] OR "认知障碍"[常用字段:智能] OR "认知功能"[常用字段:智能] OR "阿尔茨海默病"[常用字段:智能] OR "阿尔茨海默症"[常用字段:智能] OR "痴呆"[常用字段:智能] OR "老年痴呆"[常用字段:智能]) AND 2024-2025[日期]  #5" (#2) OR (#1)  #6" (#4) OR (#3)  #7" (#6) AND (#5) AND 2024-2025[日期] （n=44）  2.Manual search using the same strategy in SinoMed 2023.12.2–2023.12.31 （n=2） |

**S2 Table. Detailed characteristics of included studies.**

| **Author**  **(year)** | **Sarcopenia Status** | **Type of cognitive impairment** | **Crude OR/RR/HR** | **Adjusted OR/RR/HR** | **Covariates** |
| --- | --- | --- | --- | --- | --- |
| **The studies provided for odds ratio.** | | | | | |
| **Bian 2023[1]** | Sarcopenia | CI | 1.767 (1.142, 2.734) | 1.650 (1.048, 2.596) | age, gender, education, annual household income, living alone, BMI, hypertension, diabetes, cancer, chronic heart disease, Morse, GDS-15, social support level |
| **Ohta 2023[2]** | Sarcopenia、  Severe sarcopenia | CI | 3.160 (2.370, 4.210)  male：2.920 (1.910, 4.460)  female：3.370 (2.280, 4.960)  Severe sarcopenia:  9.910 (7.110, 13.810) | 2.190 (1.540-3.130)  male：2.000 (1.140-3.510)  female：2.360 (1.480-3.760)  Severe sarcopenia:  3.430 (2.140, 5.490) | age, gender, educational attainment, smoking status, drinking status, cohort categories, living alone, history of non-communicable diseases (hypertension, diabetes, dyslipidemia, and stroke), geriatric depressive symptom (GDS ≥ 5), frequency of going outdoors, and exercise habits |
| **Deng 2022[3]** | Sarcopenia | CI | 8.280(4.020-17.080)  male：5.140(2.140-12.350)  female：19.240(4.520-81.820) | 5.860(2.740-12.550)  male：3.250(1.220-8.700)  female：15.170(47-66.390) | age, gender, smoking, alcohol, stroke, mental health, respiratory disease, diabetes, hypertension, chronic heart disease, osteoporosis |
| **Wu 2021[4]** | Sarcopenia | CI | 4.810 (3.620−6.400) | 2.550(1.860-3.500) | age, gender, national, education, marital status, smoking, alcohol, physical activity, BMI, hypertension, diabetes, heart  disease, respiratory disease |
| **Zhu 2021[5]** | Sarcopenia | CI | 2.040 (1.160–3.570) | 2.220 (0.940-5.210) | age, gender, smoking, alcohol, BMI, BFR, hypertension, diabetes, dyslipidemia, |
| **Cipolli 2021[6]** | Possible  Sarcopenia | CI | 2.530 (1.490–4.300) | 2.520 (1.420–4.470) | age, gender, education, physical activity, hypertension, diabetes, obesity, depression |
| **Xu 2020[7]** | Sarcopenia | CI | 2.070 (1.310–3.270) | 1.960(1.170-3.270)  2.090(1.320–3.330) **^a^** | age, gender, education, marital status, smoking, alcohol, physical activity, BMI, coronary heart disease, stroke, hypertension, diabetes, chronic obstructive pulmonary disease, chronic kidney disease, osteoarthritis, tumors of any type, depression |
| **Kim 2019[8]** | Sarcopenia | CI | N/A | male: 1.760(1.040-2.990) | age, education, smoking, alcohol, nutritional status, physical activity, BMI, number of comorbidities, self-reported health status, depression |
| **Peng 2019[9]** | Sarcopenia | CI | 3.620(1.710–7.650) | 2.690(1.110-6.550)  male：1.220(0.220-6.670) | age, gender, education, physical activity, APOE e4 status, depression |
| **Wang 2018[10]** | Sarcopenia | CI | 3.380(1.960-5.830)  male：2.300(0.830-6.410)  female：3.800(1.940-7.440) | 2.630(1.330-5.210)  male：1.830(0.580-5.760)  female：2.590(1.150-5.850) | age, gender, smoking, physical activity, BMI, medication, chronic disease, depression |
| **Huang 2015[11]** | Sarcopenia | CI | 2.720 (1.328-5.569) | 1.500(0.641-3.490)  2.280(1.069-4.857) **^a^** | age, gender, BMI, alcohol, physical activity, Charlson comorbidity index, Functional Autonomy Measurement System, depression |
| **Van Kan 2013[12]** | Sarcopenia | CI | female：1.390 (0.900-2.120) | female：1.180(0.730-1.900) | age, education, ADL disability, physical activity, recruitment center |
| **Fu 2023[13]** | Sarcopenia | MCI | N/A | 1.500 (1.060- 2.140)  1.570(1.120-2.200) **^a^** | age, gender, employment status, living alone, income, education level, marital status, smoking status, alcohol intake, physical activity, hypertension, diabetes, and cardiovascular disease |
| **Sun 2023[14]** | Sarcopenia | MCI | 1.800(1.040-3.100) | 1.570(0.840-2.950) | age, gender, marital status, living alone, smoking, alcohol, physical activity, nutrition status, BMI, hypertension, osteoarthritis |
| **Lee 2023[15]** | Sarcopenia | MCI | 2.590 (1.330-5.020)  male：1.890 (0.790- 4.450)  female：3.790 (1.330, 10.810) | 2.490 (1.230-5.050)  male：1.500 (0.570-3.960)  female：4.720 (1.390-15.970) | age, education, family income, marital status, smoking, alcohol, physical activity, disease history (hypertension, diabetes), APOE 4 genotype |
| **O'Donovan**  **2022[16]** | Possible  Sarcopenia | MCI | 1.820 (1.270, 2.600) | 1.840 (1.250, 2.710) | age, gender, height, education, income, civil status, smoking, and alcohol drinking |
| **Jacob 2021[17]** | Sarcopenia | MCI | N/A | 1.600(1.320-1.930)  male：1.740(1.350-2.230)  female：1.630(1.250-2.130)  South Africa：0.690(0.110-4.240)  Ghana：1.220(0.640-2.310)  Mexico：1.270(0.590-2.710)  Russia：1.380(0.390-4.870)China：1.640(1.290-2.080)  India：1.940(1.260-3.010) | education, family income, physical activity, smoking, alcohol, hypertension, diabetes |
| **Bai 2021[18]** | Sarcopenia | MCI | 1.935(1.211-3.093)  male：1.044(0.488-2.234)  female：3.004(1.639-5.508) | 1.856(1.035-3.327)  male：0.843(0.290-2.449)  female：3.256(1.474-7.192) | age, gender, education, physical activity, smoking, alcohol, ADL disability, hypertension, diabetes, chronic heart disease, obesity, depression |
| **Chen 2021[19]** | Sarcopenia | MCI | 2.340 (1.600-3.430) | 1.670 (1.040-2.680) | age, gender, marital status, living alone, education, sleeping duration, nutrition status, physical activity, BMI, number of medications, stroke, osteoarthritis |
| **Lee 2018[20]** | Sarcopenia、  Possible sarcopenia | MCI | 5.493 (1.854-16.270)  Possible sarcopenia：  2.632 (1.070-6.474) | female：4.502(1.317-15.319)  possible sarcopenia：  2.361 (0.914-6.009) | age, education, BMI, lean body mass |
| **Salinas 2021[21]** | Sarcopenia | MCI | 1.890(1.190-3.000) | 1.740(1.020-2.960) | age, gender, education, marital status, paid job, physical activity, smoking, alcohol, vegetable and fruit intake, frailty status, diabetes, stroke, depression, hypercholesterolaemia, hypertriglycaemia, hypertension, follow-up time duration, BMI, baseline MCI or cognitive function |
| **Hu 2022[22]** | Sarcopenia、  Possible sarcopenia | MCI | 2.850 (1.940, 4.180)  possible sarcopenia：  1.750 (1.380, 2.230) | 1.720 (1.040-2.860)  possible sarcopenia：  1.430 (1.060-1.910) | age, gender, residential area, education, marital status, average household income, smoking, alcohol, daily sleep time, BMI, comorbidities, depression |
| **Someya 2022[23]** | Possible  Sarcopenia | MCI、AD | N/A | MCI:1.33 (0.92-1.93)  male：1.48 (0.87-2.50)  female：1.17 (0.68-2.01)  AD:3.40 (1.61-7.20)  male：2.84 (0.83-9.67)  female：3.56 (1.36-9.33) | age, gender, education, physical activity, hypertension, diabetes, dyslipidemia, depression |
| **Weng 2023[24]** | Sarcopenia | AD | 4.580 (1.570–13.360) | 5.350 (1.270–22.460)  4.400 (1.490–13.010) **^a^** | age, gender, height, weight, smoking, drinking, hypertension, diabetes, education, low physical activity, mini-nutritional assessments, and Hamilton Depression Scale-17 |
| **Dost 2022[25]** | Sarcopenia、  Possible sarcopenia | AD | 5.346(2.876–9.938)  possible sarcopenia：  4.286(2.851–6.443) | 3.723(1.740-7.968)  possible sarcopenia：  2.987(1.805-4.944) | age, gender, education, Age-Adjusted Charlson Comorbidity Index, number of drugs, recurrent falls, vitamin B12, vitamin D, estimated glomerular filtration rate |
| **Suzan 2022[26]** | Sarcopenia | AD | 2.689 (1.542-4.688) | 2.048 (1.049-3.998) | Parkinson’s disease, Polypharmacy, Malnutrition, depression, Delirium, Insomnia, Urinary incontinence |
| **Nishiguchi 2016[27]** | Sarcopenia | CD | N/A | 7.860(1.530-40.500) | age, gender, education, family structure, BMI, the pre-MMSE score |
| **The studies provided for relative risk.** | | | | | |
| **Ramoo 2022[28]** | Sarcopenia、  Sever sarcopenia | CI | 2.130 (1.460–3.090)  Sever sarcopenia：  2.850 (1.950–4.150) | 1.800 (1.180–2.750)  Sever sarcopenia：  2.010 (1.240–3.270) | age, gender, physical activity, depression, chronic pain, visual and hearing impairment |
| **Papachristou 2015[29]** | Sarcopenia、Sever sarcopenia | MCI | N/A | male: 1.720 (0.670–4.400)  Sever sarcopenia：  1.400 (0.560–3.510) | age, alcohol, social class, physical activity, smoking, history of cardiovascular disease and diabetes |
| **The study provided for hazard ratio.** | | | | | |
| **Batsis**  **2021[30]** | Sarcopenia | CI | 2.490 (2.230–2.780) | 1.600 (1.420–1.800) | age category, gender, smoking, status, education，comorbidities (heart disease, hypertension, diabetes, lung disease, stroke, cancer, ever walk) |
| **Ling**  **2024[31]** | Possible sarcopenia | AD | N/A | 1.320 (1.210-1.430) | age, gender, Townsend deprivation index, education, ethnicity, smoking, sleep duration and healthy diet, history of diseases (diabetes, high cholesterol, stroke, hypertension and cardiovascular disease) |

Abbreviations: OR, Odds ratio; RR, Relative risk; HR, Hazard ratio; CI, cognitive impairment; MCI, mild cognitive impairment; AD, Alzheimer's disease; CD, cognitive decline; N/A, Not applicable.

^a^: adjusted for age and gender.

S3 Table. Risk of bias assessment results for included cross-sectional studies.

| **Author**  **Year** | **1** | **2** | **3** | **4** | **5** | **6** | **7** | **8** | **9** | **10** | **11** | **Total** |
| --- | --- | --- | --- | --- | --- | --- | --- | --- | --- | --- | --- | --- |
| **Bian 2023[1]** | Y | Y | Y | Y | N | Y | Y | Y | N | Y | N | 8 |
| **Ohta 2023[2]** | Y | N | Y | Y | N | Y | Y | Y | N | Y | N | 7 |
| **Deng 2022[3]** | Y | Y | Y | U | N | Y | Y | Y | N | Y | N | 7 |
| **Wu 2021[4]** | Y | U | Y | Y | N | Y | Y | Y | N | Y | N | 7 |
| **Zhu 2021[5]** | Y | Y | Y | Y | N | Y | Y | Y | N | Y | N | 8 |
| **Cipolli 2021[6]** | Y | Y | Y | Y | N | Y | Y | Y | N | Y | N | 8 |
| **Xu 2020[7]** | Y | Y | Y | Y | N | Y | N | Y | N | N | N | 6 |
| **Kim 2019[8]** | Y | Y | Y | Y | N | Y | Y | Y | N | Y | N | 8 |
| **Peng 2019[9]** | Y | Y | Y | U | N | Y | N | Y | N | Y | N | 6 |
| **Wang 2018[10]** | Y | Y | Y | Y | N | Y | Y | Y | N | Y | N | 8 |
| **Huang 2015[11]** | Y | Y | Y | Y | N | Y | Y | Y | Y | Y | N | 9 |
| **Van Kan 2013[12]** | Y | Y | Y | Y | N | Y | N | Y | N | Y | N | 7 |
| **Fu 2023[13]** | Y | Y | Y | Y | N | Y | Y | Y | N | N | N | 7 |
| **Sun 2023[14]** | Y | Y | Y | Y | N | Y | Y | Y | N | Y | N | 8 |
| **Lee 2023[15]** | Y | Y | Y | U | N | Y | Y | Y | N | Y | N | 7 |
| **O'Donovan 2022[16]** | Y | Y | Y | Y | N | Y | U | Y | N | Y | N | 7 |
| **Jacob 2021[17]** | Y | Y | Y | Y | N | Y | Y | Y | N | Y | N | 8 |
| **Bai 2021[18]** | Y | Y | Y | Y | N | Y | Y | Y | N | Y | N | 8 |
| **Chen 2021[19]** | Y | Y | Y | Y | N | Y | Y | Y | N | Y | N | 8 |
| **Lee 2018[20]** | Y | Y | Y | Y | N | Y | Y | Y | N | Y | N | 8 |
| **Someya 2022[23]** | Y | U | Y | Y | N | Y | Y | Y | N | Y | N | 7 |
| **Weng 2023[24]** | Y | Y | Y | U | N | Y | Y | Y | N | N | N | 6 |
| **Dost 2022[25]** | Y | Y | Y | U | N | Y | Y | Y | N | Y | N | 7 |
| **Suzan 2022[26]** | Y | Y | Y | U | N | Y | N | Y | N | Y | N | 6 |
| **Papachristou2015[29]** | Y | U | Y | Y | N | Y | Y | Y | N | Y | N | 7 |

Y: Yes; N: No; U: Unclear

1: Define the source of information (survey, record review). 2: List inclusion and exclusion criteria for exposed and unexposed subjects (cases and controls) or refer to previous publications. 3: Indicate time period used for identifying patients. 4: Indicate whether or not subjects were consecutive if not population-based. 5: lndicate if evaluators of subjective components of study were masked to other aspects of the status of the participants. 6: Describe any assessments undertaken for quality assurance purposes (e.g., test/retest of primary outcome measurements). 7. Explain any patient exclusions from analysis. 8: Describe how confounding was assessed and/or controlled. 9: If applicable, explain how missing data were handled in the analysis. 10: Summarize patient response rates and completeness of data collection. 11: Clarify what follow-up, if any, was expected and the percentage of patients for which incomplete data or follow-up was obtained

S4 Table. Risk of bias assessment results for included cohort studies.

| **Studies** | **Selection of cohorts** | | | | **Comparability of cohorts** | **Assessment of outcome** | | | **Total** |
| --- | --- | --- | --- | --- | --- | --- | --- | --- | --- |
|  | **Representative-ness of the exposed cohort** | **Selection of the non-exposed cohort** | **Ascertain-ment of exposure** | **Demonstration that outcome of interest was not present at start of study** | **Comparability of cohorts on the basis of the design or analysis** | **Assess-**  **ment of outcome** | **Was follow up long enough for outcomes to occur** | **Adequacy of follow up of cohorts** |  |
| **Salinas 2021[21]** | 1 | 1 | 1 | 1 | 2 | 1 | 1 | 0 | 8 |
| **Hu 2022[22]** | 1 | 1 | 1 | 1 | 2 | 1 | 1 | 0 | 8 |
| **Nishiguchi 2016[27]** | 1 | 1 | 1 | 1 | 2 | 1 | 1 | 0 | 8 |
| **Ramoo 2022[28]** | 1 | 1 | 1 | 1 | 1 | 1 | 1 | 0 | 7 |
| **Batsis 2021[30]** | 1 | 1 | 1 | 1 | 1 | 1 | 1 | 1 | 8 |
| **Ling 2024[31]** | 1 | 1 | 1 | 1 | 2 | 1 | 1 | 0 | 8 |

| S1 Fig. Forest plot of the association between sarcopenia and cognitive impairment (crude odds ratio). |
| --- |

|  |
| --- |
| S2 Fig. Funnel plots of the association between sarcopenia and cognitive impairment (adjusted odds ratio). |
|  |
| S3 Fig. Funnel plots of the association between sarcopenia and cognitive impairment (crude odds ratio). |
|  |
| S4 Fig. Trim-and-filling analysis of the association between sarcopenia and cognitive impairment (adjusted odds ratio). |
|  |
| S5 Fig. Sensitivity analysis of adjusted OR studies excluding comorbidities. |
|  |
| S6 Fig. Sensitivity analysis of crude OR studies excluding comorbidities. |
|  |
| S7 Fig. Sensitivity analysis of studies providing adjusted odds ratio. |
|  |
| S8 Fig. Sensitivity analysis of studies providing crude odds ratio. |

|  |
| --- |
| S9 Fig. Forest plot of the association between possible sarcopenia and cognitive impairment  (adjusted odds ratio). |
|  |
| S10 Fig. Forest plot of the association between possible sarcopenia and cognitive impairment  **(crude odds ratio).** |
|  |
| **S11 Fig. Sensitivity analysis of the relationship between possible sarcopenia and cognitive impairment using adjusted odds ratios.** |
|  |
| **S12 Fig. Sensitivity analysis of the relationship between possible sarcopenia and cognitive impairment using crude odds ratios.** |

**References**

**1.** Bian D, Li X, Xiao Y, Song K, Wang L, Shen J, et al. Relationship between social support, sarcopenia, and cognitive impairment in Chinese community-dwelling older adults. J Nutr Health Aging. 2023;27(9):726-33. doi: 10.1007/s12603-023-1973-2 PMID:37754212

**2.** Ohta T, Sasai H, Osuka Y, Kojima N, Abe T, Yamashita M, et al. Age- and sex-specific associations between sarcopenia severity and poor cognitive function among community-dwelling older adults in Japan: The IRIDE Cohort Study. Front Public Health. 2023;11:1148404. doi: 10.3389/fpubh.2023.1148404 PMID:37081953

**3.** Deng Z. The relationship between sarcopenic obesity and cognitive impairment in elderly inpatients. M.Sc. Thesis, Chongqing Medical University. 2022. Available from:<https://link.cnki.net/doi/10.27674/d.cnki.gcyku.2022.001208>

**4.** Wu B, Lyu YB, Cao ZJ, Wei Y, Shi WY, Gao X, et al. Associations of sarcopenia, handgrip strength and calf circumference with cognitive impairment among Chinese older adults. Biomed Environ Sci. 2021;34(11):859-70. doi: 10.3967/bes2021.119 PMID:34955146

**5.** Zhu H, Li HD, Feng BL, Zhang L, Zheng ZX, Zhang Y, et al. Association between sarcopenia and cognitive impairment in community-dwelling population. Chin Med J (Engl). 2020;134(6):725-7. doi: 10.1097/cm9.0000000000001310 PMID:33290284

**6.** Cipolli GC, Aprahamian I, Borim FSA, Falcão DVS, Cachioni M, Melo RC, et al. Probable sarcopenia is associated with cognitive impairment among community-dwelling older adults: results from the FIBRA study. Arq Neuropsiquiatr. 2021;79(5):376-83. doi: 10.1590/0004-282x-anp-2020-0186 PMID:34161525

**7.** Xu W, Chen T, Shan Q, Hu B, Zhao M, Deng X, et al. Sarcopenia is associated with cognitive decline and falls but not hospitalization in community-dwelling oldest old in China: A cross-sectional study. Med Sci Monit. 2020;26:e919894. doi: 10.12659/msm.919894 PMID:31980594

**8.** Kim M, Won CW. Sarcopenia is associated with cognitive impairment mainly due to slow gait speed: Results from the Korean Frailty and Aging Cohort Study (KFACS). Int J Environ Res Public Health. 2019;16(9). doi: 10.3390/ijerph16091491 PMID:31035553

**9.** Peng T, Chen Y, Chen T, Chiou J, Chen J. Association between sarcopenia and cognitive impairment in community-dwelling older adults. Alzheimer's Dementia. 2019;15:P822-P3. doi: 10.1016/j.jalz.2019.06.2940

**10.** Wang Y, Hao Q, Su L, Hai S, Wang H, Cao L, et al. The relationship between sarcopenia and cognitive impairment in community-dwelling elderly adults in China. J Sichuan Univ (Med Sci Edi). 2018;49(05):793-6. doi: 10.13464/j.scuxbyxb.2018.05.022

**11.** Huang C, Hwang A, Liu L, Lee W, Chen L, Peng L, et al. Association of dynapenia, sarcopenia, and cognitive impairment among community-dwelling older Taiwanese. Rejuvenation Res. 2016;19(1):71-8. doi: 10.1089/rej.2015.1710 PMID:26165544

**12.** Abellan van Kan G, Cesari M, Gillette-Guyonnet S, Dupuy C, Nourhashémi F, Schott AM, et al. Sarcopenia and cognitive impairment in elderly women: results from the EPIDOS cohort. Age Ageing. 2013;42(2):196-202. doi: 10.1093/ageing/afs173 PMID:23221099

**13.** Fu Y, Li X, Wang T, Yan S, Zhang X, Hu G, et al. The prevalence and agreement of sarcopenic obesity using different definitions and its association with mild cognitive impairment. J Alzheimers Dis. 2023;94(1):137-46. doi: 10.3233/jad-221232 PMID:37212103

**14.** Sun Y, Chen X, Guo Q. The combined effect of sarcopenia and osteoporosis on the prevalence of mild cognitive impairment in Chinese community-dwelling elderly adults. 2022 China Rehabilitation Medical Association Comprehensive Academic Annual Meeting and International Rehabilitation Medical Industry Expo; Fuzhou, Fujian, China2023. p. 9. doi: 10.26914/c.cnkihy.2023.001855

**15.** Lee HJ, Choi JY, Hong D, Kim D, Min JY, Min KB. Sex differences in the association between sarcopenia and mild cognitive impairment in the older Korean population. BMC Geriatr. 2023;23(1):332. doi: 10.1186/s12877-023-03911-4 PMID:37248457

**16.** O'Donovan G, Sarmiento OL, Hessel P, Muniz-Terrera G, Duran-Aniotz C, Ibáñez A. Associations of body mass index and sarcopenia with screen-detected mild cognitive impairment in older adults in Colombia. Front Nutr. 2022;9:1011967. doi: 10.3389/fnut.2022.1011967 PMID:36330135

**17.** Jacob L, Kostev K, Smith L, Oh H, López-Sánchez GF, Shin JI, et al. Sarcopenia and mild cognitive impairment in older adults from six low- and middle-income countries. J Alzheimers Dis. 2021;82(4):1745-54. doi: 10.3233/jad-210321 PMID:34219725

**18.** Bai A, Hu Y, Xu W, Liu J, Sun J, Zou L, et al. Prevalence of mild cognitive impairment and its correlation with sarcopenia in different genders among community-dwelling very old adults in Beijing. Chin J Clin Healthc. 2021;24(02):175-82. doi: 10.3969/J.issn.1672-6790.2021.02.006

**19.** Chen X, Han P, Yu X, Zhang Y, Song P, Liu Y, et al. Relationships between sarcopenia, depressive symptoms, and mild cognitive impairment in Chinese community-dwelling older adults. J Affect Disord. 2021;286:71-7. doi: 10.1016/j.jad.2021.02.067 PMID:33714172

**20.** Lee I, Cho J, Hong H, Jin Y, Kim D, Kang H. Sarcopenia is associated with cognitive impairment and depression in elderly Korean women. Iran J Public Health. 2018;47(3):327-34. doi: PMID:29845019

**21.** Salinas-Rodríguez A, Palazuelos-González R, Rivera-Almaraz A, Manrique-Espinoza B. Longitudinal association of sarcopenia and mild cognitive impairment among older Mexican adults. J Cachexia Sarcopenia Muscle. 2021;12(6):1848-59. doi: 10.1002/jcsm.12787 PMID:34535964

**22.** Hu Y, Peng W, Ren R, Wang Y, Wang G. Sarcopenia and mild cognitive impairment among elderly adults: The first longitudinal evidence from CHARLS. J Cachexia Sarcopenia Muscle. 2022;13(6):2944-52. doi: 10.1002/jcsm.13081 PMID:36058563

**23.** Someya Y, Tamura Y, Kaga H, Sugimoto D, Kadowaki S, Suzuki R, et al. Sarcopenic obesity is associated with cognitive impairment in community-dwelling older adults: the Bunkyo Health Study. 2022;41(5):1046-51. doi: 10.1016/j.clnu.2022.03.017

**24.** Weng Xf, Liu Sw, Li M, Zhang Y, Zhang Yc, Liu Cf, et al. Relationship between sarcopenic obesity and cognitive function in patients with mild to moderate Alzheimer's disease. 2023;23(6):944-53. doi: 10.1111/psyg.13015 PMID:37652079

**25.** Dost FS, Ates Bulut E, Dokuzlar O, Kaya D, Mutlay F, Yesil Gurel BH, et al. Sarcopenia is as common in older patients with dementia with Lewy bodies as it is in those with Alzheimer's disease. Geriatr Gerontol Int. 2022;22(5):418-24. doi: 10.1111/ggi.14383 PMID:35373438

**26.** Suzan V, Yavuzer H. Association of neurodegenerative diseases with sarcopenia and other geriatric syndromes. Turk J Geriatr. 2022;25(2). doi: 10.31086/tjgeri.2022.282

**27.** Nishiguchi S, Yamada M, Shirooka H, Nozaki Y, Fukutani N, Tashiro Y, et al. Sarcopenia as a risk factor for cognitive deterioration in community-dwelling older adults: A 1-year prospective study. J Am Med Dir Assoc. 2016;17(4):372.e5-8. doi: 10.1016/j.jamda.2015.12.096 PMID:26897591

**28.** Ramoo K, Hairi NN, Yahya A, Choo WY, Hairi FM, Peramalah D, et al. Longitudinal association between sarcopenia and cognitive impairment among older adults in rural Malaysia. Int J Environ Res Public Health. 2022;19(8). doi: 10.3390/ijerph19084723 PMID:35457592

**29.** Papachristou E, Ramsay SE, Lennon LT, Papacosta O, Iliffe S, Whincup PH, et al. The relationships between body composition characteristics and cognitive functioning in a population-based sample of older British men. BMC Geriatr. 2015;15:172. doi: 10.1186/s12877-015-0169-y PMID:26692280

**30.** Batsis JA, Haudenschild C, Roth RM, Gooding TL, Roderka MN, Masterson T, et al. Incident impaired cognitive function in sarcopenic obesity: Data from the National Health and Aging Trends Survey. J Am Med Dir Assoc. 2021;22(4):865-72.e5. doi: 10.1016/j.jamda.2020.09.008 PMID:34248422

**31.** Ling Y, Yuan S, Huang X, Tan S, Cheng H, Li L, et al. Association between probable sarcopenia and dementia risk: a prospective cohort study with mediation analysis. Transl Psychiatry. 2024;14(1). doi: 10.1038/s41398-024-03131-3 PMID:39353910
