# Supplementary material for: The association of sarcopenia, possible sarcopenia and cognitive impairment: A systematic review and meta-analysis
Source: PLoS One. 2025 May 28;20(5):e0324258. doi: 10.1371/journal.pone.0324258 (PMC12118908; doi:10.1371/journal.pone.0324258)
Supplement: S3 Data — (DOCX) [file pone.0324258.s003.docx]

**S1Table. List of included studies (n=31).**

| **Code** | **Included Studies** | **Extractors** |
| --- | --- | --- |
| **1** | Bai A, Hu Y, Xu W, Liu J, Sun J, Zou L, et al. Prevalence of mild cognitive impairment and its correlation with sarcopenia in different genders among community-dwelling very old adults in Beijing. Chin J Clin Healthc. 2021;24(02):175-82. doi: 10.1093/ageing/afac173 | December 2023  Huang J.H., Li M. |
| **2** | Deng Z. The relationship between sarcopenic obesity and cognitive impairment in elderly inpatients [Research Thesis]: Chongqing Medical University; 2022. https://d.wanfangdata.com.cn/thesis/ChhUaGVzaXNOZXdTMjAyNDA5MjAxNTE3MjUSCUQwMjc3MDgwNxoIMXRuZXNmc2I%3D | December 2023  Huang J.H., Li M. |
| **3** | Sun Y, Chen X, Guo Q. The combined effect of sarcopenia and osteoporosis on the prevalence of mild cognitive impairment in Chinese community-dwelling elderly adults. 2022 China Rehabilitation Medical Association Comprehensive Academic Annual Meeting and International Rehabilitation Medical Industry Expo; Fuzhou, Fujian, China2023. p. 9. doi: 10.26914/c.cnkihy.2023.001855 | December 2023  Huang J.H., Li M. |
| **4** | Wang Y, Hao Q, Su L, Hai S, Wang H, Cao L, et al. The relationship between sarcopenia and cognitive impairment in community-dwelling elderly adults in China.J Sichuan Univ(Med Sci Edi). 2018;49(05):793-6. doi:10.13464/j.scuxbyxb.2018.05.022. | December 2023  Huang J.H., Li M.  * Conflicts: Li J |
| **5** | Batsis JA, Haudenschild C, Roth RM, Gooding TL, Roderka MN, Masterson T, et al. Incident impaired cognitive function in sarcopenic obesity: Data from the National Health and Aging Trends Survey. J Am Med Dir Assoc. 2021;22(4):865-72.e5. doi: 10.1016/j.jamda.2020.09.008. | December 2023  Huang J.H., Li M. |
| **6** | Bian D, Li X, Xiao Y, Song K, Wang L, Shen J, et al. Relationship between social support, sarcopenia, and cognitive impairment in Chinese community-dwelling older adults. J Nutr Health Aging. 2023;27(9):726-33. doi: 10.1007/s12603-023-1973-2. | December 2023  Huang J.H., Li M. |
| **7** | Chen X, Han P, Yu X, Zhang Y, Song P, Liu Y, et al. Relationships between sarcopenia, depressive symptoms, and mild cognitive impairment in Chinese community-dwelling older adults. J Affect Disord. 2021;286:71-7. doi: 10.1016/j.jad.2021.02.067. | December 2023  Huang J.H., Li M. |
| **8** | Cipolli GC, Aprahamian I, Borim FSA, Falcao DVS, Cachioni M, Melo RCd, et al. Probable sarcopenia is associated with cognitive impairment among community-dwelling older adults: results from the FIBRA study. Arq Neuropsiquiatr. 2021;79(5):376-83. doi: 10.1590/0004-282x-anp-2020-0186. | December 2023  Huang J.H., Li M. |
| **9** | Dost FS, Ates Bulut E, Dokuzlar O, Kaya D, Mutlay F, Yesil Gurel BH, et al. Sarcopenia is as common in older patients with dementia with Lewy bodies as it is in those with Alzheimer's disease. Geriatr Gerontol Int. 2022;22(5):418-24. doi: 10.1111/ggi.14383. | December 2023  Huang J.H., Li M. |
| **10** | Fu Y, Li X, Wang T, Yan S, Zhang X, Hu G, et al. The prevalence and agreement of sarcopenic obesity using different definitions and its association with mild cognitive impairment. J Alzheimers Dis. 2023;94(1):137-46. doi: 10.3233/jad-221232. | December 2023  Huang J.H., Li M. |
| **11** | Hu Y, Peng W, Ren R, Wang Y, Wang G. Sarcopenia and mild cognitive impairment among elderly adults: The first longitudinal evidence from CHARLS. J Cachexia Sarcopenia Muscle. 2022;13(6):2944-52. doi: 10.1002/jcsm.13081. | December 2023  Huang J.H., Li M. |
| **12** | Huang CY, Hwang AC, Liu LK, Lee WJ, Chen LY, Peng LN, et al. Association of dynapenia, sarcopenia, and cognitive impairment among community-dwelling older Taiwanese. Rejuvenation Res. 2016;19(1):71-8. doi: 10.1089/rej.2015.1710. | December 2023  Huang J.H., Li M. |
| **13** | Jacob L, Kostev K, Smith L, Oh H, Lopez-Sanchez GF, Shin JI, et al. Sarcopenia and mild cognitive impairment in older adults from six low- and middle-income countries. J Alzheimers Dis. 2021;82(4):1745-54. doi: 10.3233/jad-210321. | December 2023  Huang J.H., Li M. |
| **14** | Kim M, Won CW. Sarcopenia is associated with cognitive impairment mainly due to slow gait speed: Results from the Korean Frailty and Aging Cohort Study (KFACS). Int J Environ Res Public Health. 2019;16(9). doi: 10.3390/ijerph16091491. | December 2023  Huang J.H., Li M. |
| **15** | Lee HJ, Choi JY, Hong D, Kim D, Min JY, Min KB. Sex differences in the association between sarcopenia and mild cognitive impairment in the older Korean population. BMC Geriatr. 2023;23(1):332. doi: 10.1186/s12877-023-03911-4. | December 2023  Huang J.H., Li M. |
| **16** | Lee I, Cho J, Hong H, Jin Y, Kim D, Kang H, et al. Sarcopenia is associated with cognitive impairment and depression in elderly Korean women. Iran J Public Health. 2018;47(3):327-34. Available at: http://ijph.tums.ac.ir | December 2023  Huang J.H., Li M. |
| **17** | Nishiguchi S, Yamada M, Shirooka H, Nozaki Y, Fukutani N, Tashiro Y, et al. Sarcopenia as a risk factor for cognitive deterioration in community-dwelling older adults: A 1-year prospective study. J Am Med Dir Assoc. 2016;17(4):372.e5-8. doi: 10.1016/j.jamda.2015.12.096. | December 2023  Huang J.H., Li M. |
| **18** | O'Donovan G, Sarmiento OL, Hessel P, Muniz-Terrera G, Duran-Aniotz C, Ibanez A. Associations of body mass index and sarcopenia with screen-detected mild cognitive impairment in older adults in Colombia. Front Nutr. 2022;9:1011967. doi: 10.3389/fnut.2022.1011967. | December 2023  Huang J.H., Li M. |
| **19** | Ohta T, Sasai H, Osuka Y, Kojima N, Abe T, Yamashita M, et al. Age- and sex-specific associations between sarcopenia severity and poor cognitive function among community-dwelling older adults in Japan: The IRIDE Cohort Study. Front Public Health. 2023;11:1148404. doi: 10.3389/fpubh.2023.1148404. | December 2023  Huang J.H., Li M. |
| **20** | Papachristou E, Ramsay SE, Lennon LT, Papacosta O, Iliffe S, Whincup PH, et al. The relationships between body composition characteristics and cognitive functioning in a population-based sample of older British men. BMC Geriatr. 2015;15:172. doi: 10.1186/s12877-015-0169-y. | December 2023  Huang J.H., Li M. |
| **21** | Peng T, Chen Y, Chen T, Chiou JM, Chen JH. Association between sarcopenia and cognitive impairment in community-dwelling older adults. Alzheimers Dement. 2019;15:P822-P3. doi：10.1016/j.jalz.2019.06.2940 | December 2023  Huang J.H., Li M.  * Conflicts: Li J |
| **22** | Ramoo K, Hairi NN, Yahya A, Choo WY, Hairi FM, Peramalah D, et al. Longitudinal association between sarcopenia and cognitive impairment among older adults in rural Malaysia. Int J Environ Res Public Health. 2022;19(8). doi: 10.3390/ijerph19084723. | December 2023  Huang J.H., Li M. |
| **23** | Salinas-Rodríguez A, Palazuelos-González R, Rivera-Almaraz A, Manrique-Espinoza B. Longitudinal association of sarcopenia and mild cognitive impairment among older Mexican adults. J Cachexia Sarcopenia Muscle. 2021;12(6):1848-59. doi: 10.1002/jcsm.12787. | December 2023  Huang J.H., Li M. |
| **24** | Someya Y, Tamura Y, Kaga H, Sugimoto D, Kadowaki S, Suzuki R, et al. Sarcopenic obesity is associated with cognitive impairment in community-dwelling older adults: the Bunkyo Health Study. Clin Nutr. 2022;41(5):1046-51. doi: 10.1016/j.clnu.2022.03.017 | December 2023  Huang J.H., Li M. |
| **25** | Suzan V, Yavuzer H. Association of neurodegenerative diseases with sarcopenia and other geriatric syndromes. Turk J Geriatr. 2022;25(2): 246-253. doi: 10.31086/tjgeri.2022.282 | December 2023  Huang J.H., Li M. |
| **26** | Abellan van Kan G, Cesari M, Gillette-Guyonnet S, Dupuy C, Nourhashemi, F, Schott AM, et al. Sarcopenia and cognitive impairment in elderly women: results from the EPIDOS cohort. Age Ageing. 2013;42(2):196-202. doi: 10.1093/ageing/afs173. | December 2023  Huang J.H., Li M. |
| **27** | Weng X, Liu S, Li M, Zhang Y, Zhang Y, Liu C, et al. Relationship between sarcopenic obesity and cognitive function in patients with mild to moderate Alzheimer's disease. Psychogeriatr. 2023;23(6):944-53. doi: 10.1111/psyg.13015 | December 2023  Huang J.H., Li M. |
| **28** | Wu B, Lyu YB, Cao ZJ, Wei Y, Shi WY, Gao X, et al. Associations of sarcopenia, handgrip strength and calf circumference with cognitive impairment among Chinese older adults. BES. 2021;34(11):859-70. doi: 10.3967/bes2021.119. | December 2023  Huang J.H., Li M. |
| **29** | Xu W, Chen T, Shan Q, Hu B, Zhao M, Deng X, et al. Sarcopenia is associated with cognitive decline and falls but not hospitalization in community-dwelling oldest old in China: A Cross-Sectional Study. Med Sci Monit. 2020;26:e919894. doi: 10.12659/msm.919894. | December 2023  Huang J.H., Li M. |
| **30** | Zhu H, Li HD, Feng BL, Zhang L, Zheng ZX, Zhang Y, et al. Association between sarcopenia and cognitive impairment in community-dwelling population. Chin Med J. 2020;134(6):725-7. doi: 10.1097/cm9.0000000000001310. | December 2023  Huang J.H., Li M. |
| **31** | Ling Y, Yuan S, Huang X, Tan S, Cheng H, Li L, et al. Association between probable sarcopenia and dementia risk: a prospective cohort study with mediation analysis. Transl Psychiatry. 2024;14(1). doi: 10.1038/s41398-024-03131-3. | March 2025  Huang J.H., Li M. |

**S2 Table. List of excluded studies with reasons for exclusion (n=5931).**

| **Exclusion Reason** | **Number of studies** | **Excluded Studies** |
| --- | --- | --- |
| **Required data not provided** | 31 | **Independent variable not sarcopenia or possible sarcopenia (n=11)**  1.Li B, Zhang Y, Liu P, Song Y, Li Y, Ma L. Correlation between muscle function and cognitive function in elderly.Practical Geriatrics. 2022;36(04):386-9. doi:10.3969/j.issn.1003-9198.2022.04.015.  2.Li C, Cai W, Wang G, Xu Y. Correlation between sarcopenia and cognitive dysfunction in the elderly in communities in Shanghai city.Geriatr Health Care. 2021;27(04):706-10. doi:10.3969/j.issn.1008-8296.2021.04.007.  3.Zhu Y, Wu Y, Yan Y, Zhang Z, Huang Z, Han C. Correlation study between pre-sarcopenia and cognitive impairment in the elderly in communitiy of Jindong District, Jinhua City, ZhejiangProvince.J Mod Med Health. 2023;39(20):3463-6+70. doi:10.3969/j.issn.1009-5519.2023.20.009.  4.Chen X, Hou L, Zhang Y, Dong B. Analysis of the prevalence of sarcopenia and its risk factors in the elderly in the Chengdu community. J Nutr Health Aging. 2021;25(5):600-605. doi: 10.1007/s12603-020-1559-1.  5.Dost FS, Erken N, Ontan MS, Bulut EA, Kaya D, Kocyigit SE, et al. Muscle strength seems to be related to the functional status and severity of dementia in older adults with Alzheimer's disease. Curr Aging Sci. 2023;16(1):75-83. doi: 10.2174/1573411018666220616114641.  6.Endo T, Akai K, Kijima T, Kitahara S, Abe T, Takeda M, et al. An association analysis between hypertension, dementia, and depression and the phases of pre-sarcopenia to sarcopenia: A cross-sectional analysis. PLoS One. 2021 Jul 22;16(7):e0252784. doi: 10.1371/journal.pone.0252784.   1. Espinel-Bermúdez MC, Sánchez-García S, García-Peña C, Trujillo X, Huerta-Viera M, Granados-García V, et al. Factores asociados a sarcopenia en adultos mayores mexicanos: Encuesta Nacional de Salud y Nutrición 2012 [Associated factors with sarcopenia among Mexican elderly: 2012 National Health and Nutrition Survey]. Rev Med Inst Mex Seguro Soc. 2018;56(Suppl 1):S46-S53. Spanish. https://pubmed.ncbi.nlm.nih.gov/29624960/ 2. Kim GR, Sun J, Han M, Nam CM, Park S. Evaluation of the directional relationship between handgrip strength and cognitive function: the Korean Longitudinal Study of Ageing. Age Ageing. 2019 May 1;48(3):426-432. doi: 10.1093/ageing/afz013. 3. Larijani B, Shafiee G, Ostovar A, Heshmat R, Sharifi F, Nabipour I. Association of osteosarcopenia and cognitive impairment in a community dwelling older population: The Bushehr Elderly Health (BEH) program. J Bone Miner Res. 2018;33:313. https://www.embase.com/records?subaction=viewrecord&id=L631815225 4. Larsson LE, Wang R, Cederholm T, Wiggenraad F, Rydén M, Hagman G, et al. Association of sarcopenia and its defining components with the degree of cognitive impairment in a memory clinic population. J Alzheimers Dis. 2023;96(2):777-788. doi: 10.3233/JAD-221186. 5. Moon JH, Moon JH, Kim KM, Choi SH, Lim S, Park KS, et al. Sarcopenia as a predictor of future cognitive impairment in older adults. J Nutr Health Aging. 2016;20(5):496-502. doi: 10.1007/s12603-015-0613-x. |
| **Dependent variable non-cognitive impairment (n=14)**  12.Du H, Yu M, Xue H, Lu X, Chang Y, Li Z. Association between sarcopenia and cognitive function in older Chinese adults: Evidence from the China health and retirement longitudinal study. Front Public Health. 2023 Jan 10;10:1078304. doi: 10.3389/fpubh.2022.1078304.  13.Henwood T, Hassan B, Swinton P, Senior H, Keogh J. Consequences of sarcopenia among nursing home residents at long-term follow-up. Geriatr Nurs. 2017 Sep-Oct;38(5):406-411. doi: 10.1016/j.gerinurse.2017.02.003.  14.Hsu YH, Liang CK, Chou MY, Liao MC, Lin YT, Chen LK, et al. Association of cognitive impairment, depressive symptoms and sarcopenia among healthy older men in the veterans retirement community in southern Taiwan: a cross-sectional study. Geriatr Gerontol Int. 2014 Feb;14 Suppl 1:102-8. doi: 10.1111/ggi.12221.  15.Li F, Bian D, Bai T, Jin H, Sun X, Lu J, et al. Cognitive impairment is associated with sarcopenia mainly related to attention and calculation in hospitalized Chinese elderly men. Asia Pac J Clin Nutr. 2022;31(3):534-542. doi: 10.6133/apjcn.202209_31(3).0021.  16.Lin A, Wang T, Li C, Pu F, Abdelrahman Z, Jin M, et al. Association of sarcopenia with cognitive function and dementia risk score: A national prospective cohort study. Metabolites. 2023 Feb 8;13(2):245. doi: 10.3390/metabo13020245.  17.Liu X, Hou L, Xia X, Liu Y, Zuo Z, Zhang Y, et al. Prevalence of sarcopenia in multi ethnics adults and the association with cognitive impairment: findings from West-China health and aging trend study. BMC Geriatr. 2020 Feb 17;20(1):63. doi: 10.1186/s12877-020-1468-5.  18.Liu X, Xia X, Hu F, Hou L, Jia S, Liu Y, et al. Nutrition status mediates the association between cognitive decline and sarcopenia. Aging (Albany NY). 2021 Mar 10;13(6):8599-8610. doi: 10.18632/aging.202672.  19.Maeda K, Akagi J. Cognitive decline is an independent comorbidity associated with sarcopenia in geriatric hospital patients. Clin Nutr. 2015;34:S137. doi: 10.1016/S0261-5614(15)30458-1  20.Maeda K, Akagi J. Cognitive impairment is independently associated with definitive and possible sarcopenia in hospitalized older adults: The prevalence and impact of comorbidities. Geriatr Gerontol Int. 2017;17(7):1048-56. doi: 10.1111/ggi.12825.  21.Maniscalco L, Veronese N, Ragusa FS, Vernuccio L, Dominguez LJ, Smith L, et al. Sarcopenia using muscle mass prediction model and cognitive impairment: A longitudinal analysis from the English longitudinal study on ageing. Arch Gerontol Geriatr. 2024 Feb;117:105160. doi: 10.1016/j.archger.2023.105160.  22.Tolea MI, Galvin JE. Sarcopenia and impairment in cognitive and physical performance. Clin Interv Aging. 2015 Mar 30;10:663-71. doi: 10.2147/CIA.S76275.  23.Yuenyongchaiwat K, Boonsinsukh R. Sarcopenia and its relationships with depression, cognition, and physical activity in Thai community-dwelling older adults. Curr Gerontol Geriatr Res. 2020 Dec 22;2020:8041489. doi: 10.1155/2020/8041489.  24.Liu G, Hong C, Xu S, Huang Y, Zheng F, Gao Y, et al. Association of sarcopenia with Parkinson's disease and related functional degeneration among older adults: A prospective cohort study in Europe. J Affect Disord. 2025 Apr 1;374:553-562. doi: 10.1016/j.jad.2025.01.084.  25.Kim J, Suh SI, Park YJ, Kang M, Chuang SJ, Lee ES, et al. Sarcopenia is a predictor for Alzheimer's continuum and related clinical outcomes. Sci Rep. 2024 Sep 10;14(1):21074. doi: 10.1038/s41598-024-62918-y. |
| **Required values not provided (n=6)**  26.Amini N, Dupont J, Lapauw L, Vercauteren L, Antonio L, O'Neill TW, et al. Sarcopenia-defining parameters, but not sarcopenia, are associated with cognitive domains in middle-aged and older European men. J Cachexia Sarcopenia Muscle. 2023 Jun;14(3):1520-1532. doi: 10.1002/jcsm.13229.  27.Baek JY, Lee E, Kim WJ, Jiang IY, Jung HW. A cumulative muscle index and its parameters for predicting future cognitive decline: Longitudinal outcomes of the ASPRA cohort. Int J Environ Res Public Health. 2021 Jul 9;18(14):7350. doi: 10.3390/ijerph18147350.  28.Hu F, Liu H, Liu X, Jia S, Zhao W, Zhou L, et al. Nutritional status mediates the relationship between sarcopenia and cognitive impairment: findings from the WCHAT study. Aging Clin Exp Res, 2021, 33(12): 3215-22. doi: 10.1007/s40520-021-01883-2  29.Jin Hee L, Tae Hui K, Hyun-Ghang J, Jae Young P, Hyeon J, Ki Woong K. Association of sarcopenia and cognitive impairment among older people in Korea. Eur Neuropsychopharmacol. 2015;25:S594.<https://www.embase.com/records?subaction=viewrecord&id=L72129926>  30.Peng TC, Chiou JM, Chen TF, Chen YC, Chen JH. Grip Strength and Sarcopenia predict 2-year cognitive impairment in community-dwelling older adults. J Am Med Dir Assoc. 2023 Mar;24(3):292-298.e1. doi: 10.1016/j.jamda.2022.10.015.  31.Yigit B, Oner C, Cetin H, Simsek EE. Association between sarcopenia and cognitive functions in older individuals: A cross-sectional study. Ann Geriatr Med Res. 2022 Jun;26(2):134-139. doi: 10.4235/agmr.22.0027. |
| **Non-English or Chinese paper** | 2 | 1. Sampaio RX, Barros RdS, Cera ML, Mendes FAdS, Garcia PA, et al. Associação dos parâmetros clínicos de sarcopenia e comprometimento cognitivo em pessoas idosas: estudo transversal impairment in older people: cross-sectional study. Revista Brasileira de Geriatria e Gerontologia. 2023;26:e220181-e. doi: 10.1590/1981-22562023026.220181.pt   Taniguchi Y, Seino S, Fujiwara Y, Nofuji Y, Nishi M, Murayama H, et al. [Cross-sectional and longitudinal associations of physical performance and skeletal muscle mass with cognition and cognitive decline among community-dwelling older Japanese]. Nihon Ronen Igakkai Zasshi. 2015;52(3):269-77. Japanese. doi: 10.3143/geriatrics.52.269. |
| **Study subjects deviate from specified criteria** | 6 | 1. [[1]](#footnote-1)Ida S, Nakai M, Ito S, Ishihara Y, Imataka K, Uchida A, et al. Association between sarcopenia and mild cognitive impairment using the Japanese version of the SARC-F in elderly patients with diabetes. J Am Med Dir Assoc. 2017 Sep 1;18(9):809.e9-809.e13. doi: 10.1016/j.jamda.2017.06.012. 2. [[2]](#footnote-2)Inoue T, Shimizu A, Satake S, Matsui Y, Ueshima, J, Murotani, K, et al. Association between osteosarcopenia and cognitive frailty in older outpatients visiting a frailty clinic. Arch Gerontol Geriatr. 2022 Jan-Feb;98:104530. doi: 10.1016/j.archger.2021.104530. 3. [[3]](#footnote-3)Wang H, Hai S, Liu YX, Cao L, Liu Y, Liu P, et al. Associations between sacopenic obesity and cognitive impairment in elderly Chinese community-dwelling individuals. J Nutr Health Aging. 2019;23(1):14-20. doi: 10.1007/s12603-018-1088-3. 4. [[4]](#footnote-4)Vallejo MS, Blümel JE, Chedraui P, Tserotas K, Salinas C, Rodrigues MA, et al. Risk of sarcopenia: A red flag for cognitive decline in postmenopause? Maturitas. 2025 Mar;194:108193. doi: 10.1016/j.maturitas.2025.108193. 5. [[5]](#footnote-5)Zhang C, Peng W, Liang W, Guo T, Hu K, Su W, et al. Sarcopenia and cognitive impairment in older adults: Long-term prognostic implications based on the National Health and Nutrition Examination Survey (2011-2014). Exp Gerontol. 2024 Oct 15;196:112561. doi: 10.1016/j.exger.2024.112561. 6. [[6]](#footnote-6)Lee JJ, Woo HD, Kim JH, Jung EJ, Lee K. Association of sarcopenia, ambient air pollution and cognitive function in a community-dwelling middle-aged and elderly Korean population: a community-based cohort study. BMJ Open. 2024 Dec 5;14(12):e092448. doi: 10.1136/bmjopen-2024-092448. |
| **Repeat population** | 2 | 1.Yang M, Yang Y, Wu T, Nan J, Li Y, Wang X, et al. A nested case-control study on the effect of sarcopenia on mild cognitive impairment using the CHARLS database. Geriatr Nurs. 2025 Jan-Feb;61:568-573. doi: 10.1016/j.gerinurse.2024.12.019.  2.Chen L, Li D, Tang K, Li Z, Huang X. Sleep duration and leisure activities are involved in regulating the association of depressive symptoms, muscle strength, physical function and mild cognitive impairment. Heliyon. 2024 Jun 27;10(13):e33832. doi: 10.1016/j.heliyon.2024.e33832. |
| **Repeat research** | 4 | 1. Du H, Yu M, Xue H, Lu X, Chang Y, Li Z. (2022). Association between sarcopenia and cognitive function in older Chinese adults: Evidence from the China health and retirement longitudinal study. Proceedings of the 7th Cross-Strait and Hong Kong and Macao Nutrition Science Conference, Qingdao, Shandong, China. doi:10.26914/c.cnkihy.2022.064908.  2.Bai A, Xu W, Sun J, Liu J, Deng X, Wu L, et al. Associations of sarcopenia and its defining components with cognitive function in community-dwelling oldest old. BMC Geriatr. 2021 May 6;21(1):292. doi: 10.1186/s12877-021-02190-1.  3.Chen LK, Chou MY, Liang CK. Association of cognitive impairment, depressive symptoms and sarcopenia among healthy older men in the veterans retirement community in southern Taiwan: A cross-sectional study. European Geriatric Medicine. 2014;5:S185. doi: 10.1016/s1878-7649(14)70492-9  4.Chen L. A study on mediators and indirect moderators of sarcopenia and mild cognitive impairment [Master]: Guangdong Medical University; 2024. https://d.wanfangdata.com.cn/thesis/ChhUaGVzaXNOZXdTMjAyNDA5MjAxNTE3MjUSCUQwMzQ4NTE5ORoIdG1iNTJtaDk%3D |
| **Animal experiment** | 247 | 1. Chen H, Wu W. Effects of Monosodium Glutamate and High-Sugar Diet on Metabolic and Memory Disorders in Rats with Type 2 Diabetes. Pharmaceutical Biotechnology. 2021;28(1):50-6. doi: 10.19526/j.cnki.1005-8915.20210111  2. Feng Y, Mou Y, Wang L, Hu M. Research on the Effect of Foshou Yangxin Decoction on Improving Cognitive Impairment in a Rat Model of Myocardial Mitochondrial Damage in Qi Deficiency and Blood Stasis Heart Failure Based on the Theory of "Heart and Brain Treatment". Asia-Pacific Traditional Medicine. 2024;20(7):17-23. doi: 10.11954/ytctyy.202407004  3. Fu H, Zhong Z, Gu X. Effect of Dexmedetomidine on Postoperative Cognitive Dysfunction Induced by Myocardial  Ischemia-reperfusion Based on SIRT1 Signaling. Chin J Clin Neurosci. 2024;32(1):13-21.  4. Hao H, Wang A, Wang L, Sun H. Protective Effect of Puerarin on Cognitive Dysfunction in Diabetic Rats Based on IRS-1/PI3 K/Akt Pathway. Frontiers in Pharmaceutical Sciences. 2019;22(7):1220-6. doi: 10.3969/j.issn.1008-049X.2019.07.008  5. Huang H, Du H, Li J, Kou J, Quan Q, Liu J, et al. Effect of Xanthohumol on Cognitive Impairment after Myocardial Ischemia-reperfusion in Rats with High-fat Diet. Progress in Modern Biomedicine. 2024;24(21):4020-7. doi: 10.13241/j.cnki.pmb.2024.21.004  6. Huang M, Ma R, Xiao Y, Huang M, Huang F, Huang F, et al. Study on rat model of chronic kidney disease complicated with cognitive impairment. Journal of Youjiang Medical University for Nationalities. 2021;43(5):579-83. doi: 10.3969/j.issn.1001-5817.2021.05.001  7. Jiang F, Sun J, Wang B, Zhang X, Zhu H, Chen H, et al. Relationship between hippocampal miR-3065-5p and IGF-1/PI3K/Akt signaling pathway in a mouse model of perioperative neurocognitive disorder. Chinese Journal of Anesthesiology. 2023;43(2):170-5. doi: 10.3760/cma.j.cn131073.20220625.00210  8. Li H, Lian Y, Liu R, Li S, Zhu Y. Neuroprotective effect of nobiletin on cognitive dysfunction rats after sevoflurane anesthesia. Chinese Journal of Behavioral Medicine and Brain Science. 2021;30(2):104-11. doi: 10.3760/cma.j.cn371468-20200617-01484  9. Li Y, Zhang Q, Yin C, Yu J, Hou Z, Wang Q. Effect of pre-infusion of young rat plasma on postoperative cognitive function in aged rats and role of PI3K/Akt signaling pathway. Chinese Journal of Anesthesiology. 2021;41(11):1338-42. doi: 10.3760/cma.j.cn131073.20210809.01114  10. Li Y, Song G, Xin S, Lin L, Cao Y. Identification of the immunogenicity of multivalence Aβ3-10 adenovirus vaccine and expression of Aβ in nasal mucosal in BALB/c mice. Journal of Dalian Medical University. 2016;38(4):320-5. doi: 10.11724/jdmu.2016.04.02  11. Liao Q, Yang H, Lu J, Liu Y, Ma L, Hou M, et al. Effect of amylin on learning and memory abilities and Akt signaling pathway in mice with Alzheimer’s disease. Journal of Medical Colleges of PLA. 2024;46(21):2467-74. doi: 10.16016/j.2097-0927.202404087  12. Lin Z, Cui X, He J, Shi W, Zeng L, Cheng C, et al. Effect of sodium phosphocreatine on learning,memory,and cognitive impairment in aged rats with VaD and its mechanism. Journal of Guizhou Medical University. 2023;48(7):745-52. doi: 10.19367/j.cnki.2096-8388.2023.07.001  13. Mao Y. Impact of Dexmedetomidine Combined with Mild Hypothermia on Cognitive Function in Rats with Sepsis-Related Encephalopathy. Jiangxi Medical Journal. 2018;53(10):1155-7,60. doi: 10.3969/j.issn.1006-2238.2018.10.042  14. Qu X, Piao C, Xiong C, Li P, Liu M, Zhou X. Improvement Effects of Shenrong Bunao Capsule on Learning and Memory Ability of Alzheimer's Disease Model Mice and Its Mechanism Study. China Pharmacy. 2019;30(23):3221-6. doi: 10.6039/j.issn.1001-0408.2019.23.10  15. Sheng R, Su R, Cao Y, Ke C, Tan Y, Pan J, et al. Effects of electroacupuncture on heart/pericardium meridian for memory ability and Aβ₁-₄₂ protein rats with Alzheimer’s disease. Shaanxi Journal of Traditional Chinese Medicine. 2024;45(11):1443-8. doi: 10.3969/j.issn.1000-7369.2024.11.001  16. Su L, Xu L, Guo T, Bian L, Zhang C, Wang S. Role of PI3K/Akt/mTOR signaling pathway in edaravone-induced reduction of postoperative cognitive dysfunction in aged rats. Chinese Journal of Anesthesiology. 2023;43(4):432-6. doi: 10.3760/cma.j.cn131073.20221025.00409  17. Tang X, Tan H, Zhao Q, Li Y, Wu D. Neuroprotective effect and mechanism of penehyclidine hydrochloride on model rats with cerebral infarction. Guangxi Medical Journal. 2021;43(23):2829-33,44. doi: 10.11675/j.issn.0253-4304.2021.23.13  18. Tian Y, Wu C, Zhang Y, Huang C. Role of interleukin-6 in brain tissues in cognitive impairment after myocardial infarction in mice. Chinese Journal of Anesthesiology. 2023;43(8):991-5. doi: 10.3760/cma.j.cn131073.20230418.00819  19. Wang B, Lin X, Han M, Liu Y, Tang C. Effects of Metformin on Cognitive Dysfunction and PI3K/Akt Pathway in Alzheimer's Disease Rats. Laboratory Animal and Comparative Medicine. 2021;41(4):313-20. doi: 10.12300/j.issn.1674-5817.2020.185  20. Wang L, Lai X, Liu S, Xu L, Shen Y, Hu H. Effects of Qingxin Kaiqiao Formula on Learning and Memory Ability of APP/PS1 Double Transgenic Mice Based on BDNF/TrkB Pathway. Chinese Archives of Traditional Chinese Medicine. 2024;42(3):172-7. doi: 10.13193/j.issn.1673-7717.2024.03.032  21. Wang R, Song J, Zhao H, Jia Y, Yuan Y, Ding R, et al. Effect of Huanglian Jiedu Decoction on TREM2/Akt/GSK3β pathway in cerebral cortex of APP/PS1 transgenic mice. China journal of Chinese materia medica. 2024;49(7):1924-31. doi: 10.19540/j.cnki.cjcmm.20240115.702  22. Wang Y, Xu Y, Zhang Z, Sun H, Zhang N, Yang B. Effect of Drynaria total flavonoids on the expression of NMDAR1, GluR2 and CaMK Ⅱ in the brain of hydrocortisone model mice. Journal of Hainan Medical University. 2022;28(24):1860-6. doi: 10.13210/j.cnki.jhmu.20220913.001  23. Xu B, Li J, Chai X, Yang J. Effects of sevoflurane and fentanyl on long term cognitive function in aged rats. Acta Universitatis Medicinalis Anhui. 2018;53(1):81-5. doi: 10.19405/j.cnki.issn1000-1492.2018.01.018  24. Xue J, Ma P, Ding Y, Wang C. Effects of transcutaneous auricular-vagus nerve stimulation on learning and memory ability and cognitive function in type 2 diabetic rats. Modern Journal of Integrated Traditional Chinese and Western Medicine. 2024;33(6):767-71. doi: 10.3969/j.issn.1008-8849.2024.06.007  25. Yang H, Li C, Du Q, Tang L, Zhu Q, Lin X, et al. Effects of Zuogui Jiangtang Jieyu Formula on glucose uptake and storage in hippocampus of rats with diabetes complicated with depression. China Journal of Traditional Chinese Medicine and Pharmacy. 2020;35(5):2332-7.  26. Yang R, Tian YM, Jin YJ, Zhai WJ, Zhang T, Zhao Z, et al. Granules Improve Learning and Memory Abilities and Cerebral Blood Flow in Rat Model of Vascular Cognitive Impairment via PI3K/Akt Signaling Pathway. Chinese Journal of Experimental Traditional Medical Formulae. 2024;30(22):52-60. doi: 10.13422/j.cnki.syfjx.20240836  27. Yang W, He X, Bai X, Yu L, Li Z, Zhang J, et al. Effects of carnosine on oxidative stress and NF-κB signaling pathway in rats with vascular cognitive impairment. Journal of Jilin University (Medicine Edition). 2020;46(2):329-34, Front insertion 3. doi: 10.13481/j.1671-587x.20200220  28. Yu J, Zhang Y, Mao L, Chen Q. Mechanism of sevoflurane anesthesia to impair cognitive function in gastric  cancer rats through Nrf2/xCT signaling pathway. Northwest Pharmaceutical Journal. 2025;40(1):1-8. doi: 10.3969/j.issn.1004-2407.2025.01.001  29. Yu Y, Hou J, Liu X, Chen L, Wan X, Xia Z. Role of hippocampal REV-ERBα in postoperative cognitive dysfunction in rats. Chinese Journal of Anesthesiology. 2022;42(9):1059-63. doi: 10.3760/cma.j.cn131073.20220507.00908  30. Zhang D, Fu Z, Chen L, Wu M, Li G, Li Q, et al. The beneficial effect of Irisin on lipopolysaccharide-induced inflammatory cognitive impairment of mice. Chinese Journal of Gerontology. 2023;43(11):2693-7. doi: 10.3969/j.issn.1005-9202.2023.11.039  31. Zhao D, Liu Y, Zhao Y, Lu M, Yang W, He X, et al. Effect of Carnosine on oxidative stress and NF-κB signaling pathway in hippocampus of diabetic rats. Chinese Journal of Clinical Anatomy. 2018;36(5):514-9. doi: 10.13418/j.issn.1001-165x.2018.05.007  32. Zhao X, Yang W, Han C, Chang X, Chen Y. Effects of sevoflurane postconditioning on cognition and hippocampal inflammation in myocardial ischemia-reperfusion rats. Journal of Shanxi Medical University. 2022;53(5):592-7. doi: 10.13753/j.issn.1007-6611.2022.05.013  33. Zhao Y, Dong R, Liu Y. Effects and Mechanismof QidiTangshen Granules on Cognitive Functionof Rats with-Diabetes. World Journal of Integrated Traditional and Western Medicine. 2022;17(4):725-9,47. doi: 10.13935/j.cnki.sjzx.220415  34. Zheng J, Mo X, Zeng L, Sun S, Sun H. Effects of intraventricular injection of AchRab on the neuronal apoptosis and the expression of nitric oxide synthase in rat brains. Journal of Apoplexy and Nervous Diseases. 2004;21(3):214-6. doi: 10.3969/j.issn.1003-2754.2004.03.007  35. Zheng X, Wei W, Li H, Ding L, Xue X. The Effects of Ze Xie Tang on Cognitive Function and Neuroinflammation in the Brain Tissue of Mice with Cognitive Impairment Induced by a High-Calorie Diet. Journal of Traditional Chinese Medicine. 2024;65(4):395-403. doi: 10.13288/j.11-2166/r.2024.04.011  36. Zhong H, Jiang X, He Z, Mao Z. Based on PI3K/Akt signaling pathway to explore the effects of olanzapine on cognitive function and neuronal damage in schizophrenic rats. Acta Anatomica Sinica. 2022;53(6):719-26. doi: 10.16098/j.issn.0529-1356.2022.06.005  37. Zhong M, Liu C, Qiu B, Ding Y, Cui M. Research on Olanzapine's Neuroprotective Effect on Schizophrenic Model Rats Through PI3K/Akt Pathway. Evaluation and Analysis of Drug-Use in Hospitals of China. 2020;20(5):554-8. doi: 10.14009/j.issn.1672-2124.2020.05.012  38. Zhu J, Liu Z, Zhong R, Li J, Huang X. The Impact and Mechanism of miR-126 on Cognitive Impairment Induced by Sevoflurane Anesthesia in Aged Rats. Chinese Journal of Gerontology. 2024;44(5):1215-9. doi: 10.3969/j.issn.1005-9202.2024.05.044  39. Abdurahman NA. Anti-aging effects of Chinese herbal compound granules on D-galactose-induced aging mice. Master's Degree, Xinjiang Medical University. 2019. Available from:https://d.wanfangdata.com.cn/thesis/ChJUaGVzaXNOZXdTMjAyMzA5MDESCUQwMTcyODA4NhoIanZsY3RoNGg%3D  40. ALMAGHALSA ZM. A Comparative Proteome Study of MicroRNA-21a-5pwithin the Mouse Model After Spinal Cord Injury andits Possible Role in Different Pathways. Doctoral Degree, Shandong University. 2019. Available from:https://d.wanfangdata.com.cn/thesis/ChJUaGVzaXNOZXdTMjAyMzA5MDESCFkzNjc2NjEyGghqdmxjdGg0aA%3D%3D  41. Bai S. ThestemcelldistributionandMRimaginginthe total-body/localbrainirradiatedrats. Doctoral Degree, Zhongshan University. 2009. Available from:https://d.wanfangdata.com.cn/thesis/ChJUaGVzaXNOZXdTMjAyMzA5MDESCFkxNDc3OTgzGghtdmFxOWZiaw%3D%3D  42. Bao X. The role of Liver X receptor β in the regulation ofinflammatory pain. Doctoral Degree, Third Military Medical University. 2016. Available from:https://d.wanfangdata.com.cn/thesis/ChJUaGVzaXNOZXdTMjAyMzA5MDESCFkzMTE1OTkxGghtdmFxOWZiaw%3D%3D  43. Bi J. An Experimental Study of the Treatment of Kangnaolinglf for Alzheimer's disease (AD) of RAT. Doctoral Degree, Shandong University Of Traditional Chinese Medicine. 2004. Available from:https://d.wanfangdata.com.cn/thesis/ChJUaGVzaXNOZXdTMjAyMzA5MDESB1k2MjIzMzcaCGp2bGN0aDRo  44. Castillo-Mariqueo L, Hernández LO, Ojeda RB, Llort LG. P6: Functional relationship between locomotion and structural muscle fibers in Alzheimer's Disease, an experimental report from 3xTg-AD mice. 2024. p. 120-1.  45. Chen B. The Mechanism of Electroacupuncture Improves the Recovery of Cognitive Function Through Cofilin-mediated Cellular Apoptosis After Ischemic Stroke. Doctoral Degree, Fujian University Of Traditional Chinese Medicine. 2017. Available from:https://d.wanfangdata.com.cn/thesis/ChJUaGVzaXNOZXdTMjAyMzA5MDESCFkzMjQzNjI5GghkN3RsaGJuaQ%3D%3D  46. Chen D. Study on the Neuroprotective Effects and Mechanisms of Glycerol in Rats with Subarachnoid Hemorrhage. Doctoral Degree, Chongqing Medical University. 2011. Available from:https://d.wanfangdata.com.cn/thesis/ChJUaGVzaXNOZXdTMjAyMzA5MDESCFkyMDIxMDMxGghtdmFxOWZiaw%3D%3D  47. Chen J. Experimental study of Glycosides of Cistanche on learningmemory ability、 the expression of p-tau protein and proteomics in vascular dementia rat. Doctoral Degree, Shandong University. 2015. Available from:https://d.wanfangdata.com.cn/thesis/ChJUaGVzaXNOZXdTMjAyMzA5MDESCFkyNzkxMTM1GghkN3RsaGJuaQ%3D%3D  48. Chen M. Effects of Novel GSK-3 Inhibitors on Cognitive Dysfunction and Peroxidative Damage in AD Rats‘. Master's Degree, Shandong University. 2020. Available from:https://d.wanfangdata.com.cn/thesis/ChJUaGVzaXNOZXdTMjAyMzA5MDESCFkzNzYxMzk1Ggh2bTI2NnlxZQ%3D%3D  49. Chen S. The Effect of Acupuncture on Learning and Memory Impairment in Hypoxic-ischemic Brain Damage. Doctoral Degree, China Academy of Chinese Medical Sciences. 2006. Available from:https://d.wanfangdata.com.cn/thesis/ChJUaGVzaXNOZXdTMjAyMzA5MDESB1k5MzU4NzQaCG12YXE5ZmJr  50. Chen S. The inhibitory effect of chemerin-9 on experimentalabdominal aortic aneurysm in mice. Master's Degree, Shandong University. 2021. Available from:https://d.wanfangdata.com.cn/thesis/ChJUaGVzaXNOZXdTMjAyMzA5MDESCFkzODE1Mzc3GghkN3RsaGJuaQ%3D%3D  51. Chen W. The Research on Changes of Neuronal IP3R1 ExpressionInduced by Chronic Alcohol Exposure in Mice. Master's Degree, China Medical University. 2013. Available from:https://d.wanfangdata.com.cn/thesis/ChJUaGVzaXNOZXdTMjAyMzA5MDESCFkyMjk2NTA0GghkN3RsaGJuaQ%3D%3D  52. Chen Y. Effects of time-restricted feeding on NF-κB, TNF-α, IL-10 in atherosclerosis mouse model. Master's Degree, Zhengzhou University. 2020. Available from:https://d.wanfangdata.com.cn/thesis/ChJUaGVzaXNOZXdTMjAyMzA5MDESCUQwMTk3NjU1OBoIanZsY3RoNGg%3D  53. Cui C. Oligodendrocytic and astrocytic FGF9 may be closely associated with the disease progression of ALS mice. Master's Degree, Hebei Medical University. 2017. Available from:https://d.wanfangdata.com.cn/thesis/ChJUaGVzaXNOZXdTMjAyMzA5MDESCUQwMTIwMTUyMRoIanZsY3RoNGg%3D  54. Cui X. Proteomic Study of Aging in Mouse Brain Cortex, Plasma Exosomes, and Skeletal Muscle. Master's degree, Zhengzhou University. 2024. Available from:https://d.wanfangdata.com.cn/thesis/ChhUaGVzaXNOZXdTMjAyNDA5MjAxNTE3MjUSCFk0MzQ4MDcyGghscW1xaXRrcA%3D%3D  55. Diao C. Investigation of Metabolic Pathways in Serum and Brain of Diabetic Rats induced by STZ. Master's Degree, Wenzhou Medical University. 2014. Available from:https://d.wanfangdata.com.cn/thesis/ChJUaGVzaXNOZXdTMjAyMzA5MDESCFkyNjkxNDMxGghtdmFxOWZiaw%3D%3D  56. Diao ZZQ, Ge H, Zhao C, Zhou J. The Role and Related Mechanisms of Endoplasmic Reticulum Stress in Hepatocyte Apoptosis in Rats with Fulminant Hepatic Failure. Proceedings of the 5th National Conference on Difficult and Critical Liver Diseases; Nanjing, China2010. p. 429-32.  57. Ding J. Establishment of animal model of depression and change of cerebral PIP2 metabolism. Master's Degree, Hebei Medical University. 2016. Available from:https://d.wanfangdata.com.cn/thesis/ChJUaGVzaXNOZXdTMjAyMzA5MDESB0Q4NDQ3OTgaCGp2bGN0aDRo  58. Du A. Protective effect and mechanism of butylphthalide on rats with chronic alcoholism. Doctoral Degree, Zhengzhou University. 2017. Available from:https://d.wanfangdata.com.cn/thesis/ChJUaGVzaXNOZXdTMjAyMzA5MDESCUQwMTYzNjc1MRoIanZsY3RoNGg%3D  59. Du H. PPAR-y agonist pioglitazone ameliorates radiation-inducedheart injury in rats. Master's Degree, China Medical University. 2013. Available from:https://d.wanfangdata.com.cn/thesis/ChJUaGVzaXNOZXdTMjAyMzA5MDESCFkyMzI0MjQ3GghtdmFxOWZiaw%3D%3D  60. Du J. The effect of resveratrol on Bcl-2 and Bax expression in lumbarspinal cord and cortex of SOD1G93A transgenie mice. Master's Degree, Hebei Medical University. 2014. Available from:https://d.wanfangdata.com.cn/thesis/ChJUaGVzaXNOZXdTMjAyMzA5MDESCFkyNTgzMzM2GghqdmxjdGg0aA%3D%3D  61. Fan D. Studies on Protective Effects and Mechanisms of Methane on Brain Injury due to Carbon Monoxide Poisoning in Rats. Doctoral Degree, Naval Medical University. 2016. Available from:https://d.wanfangdata.com.cn/thesis/ChJUaGVzaXNOZXdTMjAyMzA5MDESCFkzMDcyNDgyGghqdmxjdGg0aA%3D%3D  62. Fan H. Preparation of soluble dietary fiber of Herba lophatheri and its improvement on  acrylamide-induced intestinal and nerve damage in mice Master's Degree, Northwest A & F University. 2022. Available from:https://d.wanfangdata.com.cn/thesis/ChJUaGVzaXNOZXdTMjAyMzA5MDESCUQwMjg3NjY4NBoIZDd0bGhibmk%3D  63. Gan L. Distribution of Neural Progenitor Cells and Neuroregeneration in Mouse Models of Neurodegenerative Diseases Doctoral Degree, Ocean University of China. 2007. Available from:https://d.wanfangdata.com.cn/thesis/ChJUaGVzaXNOZXdTMjAyMzA5MDESB0Q0NTU2NzEaCGQ3dGxoYm5p  64. Gao J. Ameliorative effect of vitamin D3 on cortical AD-like lesions in APP/PS1 mice based on MK5. Master's Degree, Zhengzhou University. 2022. Available from:https://d.wanfangdata.com.cn/thesis/ChJUaGVzaXNOZXdTMjAyMzA5MDESCFk0MDUxMjk5Ggh2bTI2NnlxZQ%3D%3D  65. Guo X. Study on Serotoninergie System Disorder and Abnormal Behavior in Offspring Schizophrenie Model Rats Induced by Pregnancy Infection. Master's Degree, Xinxiang Medical University. 2020. Available from:https://d.wanfangdata.com.cn/thesis/ChJUaGVzaXNOZXdTMjAyMzA5MDESCUQwMjI4MDQ3NBoIanZsY3RoNGg%3D  66. Hao K. Maternal Immune Activation Leads to NMDA Receptor Disorder in Offspring Rats and Relationship with Abnormal Behavior Master's Degree, Xinxiang Medical University. 2019. Available from:https://d.wanfangdata.com.cn/thesis/ChJUaGVzaXNOZXdTMjAyMzA5MDESCUQwMTk0NDU2OBoIanZsY3RoNGg%3D  67. He L. The effects of estrogen receptor beta activity modulationon hippocampal actin polymerization and learning andmemory of female mice. Master's Degree, Army Medical University. 2017. Available from:https://d.wanfangdata.com.cn/thesis/ChJUaGVzaXNOZXdTMjAyMzA5MDESCFkzMjgwMTI3GghtdmFxOWZiaw%3D%3D  68. He P. Study on the effect and mechanism of Shiquandabu Decoction on sarcopenia model rats induced by D-galactose combining with constrained motion. Doctoral Degree, Shanxi University. 2023. Available from:https://link-cnki-net-s.webvpn.cams.cn/doi/10.27284/d.cnki.gsxiu.2023.000053  69. He X. The Protective effeets of fasudil hydrochloride on injured rat cerebral neurons by LPS. Master's Degree, Zhengzhou University. 2011. Available from:https://d.wanfangdata.com.cn/thesis/ChJUaGVzaXNOZXdTMjAyMzA5MDESCFkxOTMwMzA0GghqdmxjdGg0aA%3D%3D  70. He Y. The effects of rhynchophylline on NR1 and NR2B expression of theprimary hippocampal neurons in neonatal rats. Master's Degree, Southern Medical University. 2013. Available from:https://d.wanfangdata.com.cn/thesis/ChJUaGVzaXNOZXdTMjAyMzA5MDESCFkyNDA2MjU2GghqdmxjdGg0aA%3D%3D  71. Hou Z. The Regulation of Qing Nao Tong Luo Recipe on Cerebral Angiophagy in Apolipoprotein E Gene Knockout Mice and the Mechanism Exploration. Doctoral Degree, Guangzhou University of Chinese Medicine. 2016. Available from:https://d.wanfangdata.com.cn/thesis/ChJUaGVzaXNOZXdTMjAyMzA5MDESCFkzMTQ0NTIxGghqdmxjdGg0aA%3D%3D  72. Hu C. Neuregulin 1-β (Nrg1β) protects mice cerebellum against oxidative stress and neuroinflammation. Master's Degree, Shantou University. 2016. Available from:https://d.wanfangdata.com.cn/thesis/ChJUaGVzaXNOZXdTMjAyMzA5MDESCUQwMTAyNDk5MBoIanZsY3RoNGg%3D  73. Hu M. Exon array analysis of lumbar spinal cord from hSOD1-G93A transgenic mice and exploration of pathogenesis. Doctoral Degree, Hebei Medical University. 2010. Available from:https://d.wanfangdata.com.cn/thesis/ChJUaGVzaXNOZXdTMjAyMzA5MDESCFkxODAxOTQ4GghqdmxjdGg0aA%3D%3D  74. Hu W. Role and Mechanisms of Celastrol in Treatment of Obesity in Rats Doctoral Degree, Nanjing Medical University. 2018. Available from:https://d.wanfangdata.com.cn/thesis/ChJUaGVzaXNOZXdTMjAyMzA5MDESCUQwMTU1MTUyMRoIanZsY3RoNGg%3D  75. Huai Y. L-3-n-butylphthalide improves cognitive function deficitsthrough activation of PI3K/Akt cell signaling pathway in thehippocampus of VaD mice. Doctoral Degree, Hebei Medical University. 2013. Available from:https://d.wanfangdata.com.cn/thesis/ChJUaGVzaXNOZXdTMjAyMzA5MDESCFkyMzM3MTAzGghkN3RsaGJuaQ%3D%3D  76. Iki T, Tohda C. Skeletal muscle atrophy induces memory dysfunction via hemopexin action in healthy young mice. Biochemical and Biophysical Research Communications. 2024;733. doi: 10.1016/j.bbrc.2024.150606 PMID:WOS:001313628500001  77. Ji Z. The protective effect and the morphological research ofZBPY Decoction to dendritic spines in a Model of bothAlzheimer's Disease and Spleen Yin Deficiency  Syndrome. Master's Degree, Dalian Medical University. 2007. Available from:https://d.wanfangdata.com.cn/thesis/ChJUaGVzaXNOZXdTMjAyMzA5MDESCFkxMDQ0MjE0GghkN3RsaGJuaQ%3D%3D  78. Jia X. The Effects of Exploratory and learning on Behavioral Recovery and the Expression of bFGF,Ng in Rats after Unilateral Local Cerebral Infarction. Master's Degree, Hebei Medical University. 2008. Available from:https://d.wanfangdata.com.cn/thesis/ChJUaGVzaXNOZXdTMjAyMzA5MDESCFkxMzU0MDM1GghtdmFxOWZiaw%3D%3D  79. Kang Y. Pretreatment of pentoxifylline ameliorate the function of dopaminergic neurons in ventral midbrain of epilepsy rats and the participation of Nrf2-ARE pathway Doctoral Degree, Hebei Medical University. 2016. Available from:https://d.wanfangdata.com.cn/thesis/ChJUaGVzaXNOZXdTMjAyMzA5MDESB0Q4NDM4MjYaCGp2bGN0aDRo  80. Lan J. Proteomic identification of serum proteinsin rats and human associated with chronicmethampetamine-induced physical toxicity. Master's Degree, Southern Medical University. 2011. Available from:https://d.wanfangdata.com.cn/thesis/ChJUaGVzaXNOZXdTMjAyMzA5MDESCFkxOTk3MTc4GghqdmxjdGg0aA%3D%3D  81. Lee H, Kim SY, Lim Y. <i>Solanum</i><i> melongena</i> extract supplementation protected skeletal muscle and brain damage by regulation of BDNF/PGC1α/irisin pathway via brain function-related myokines in high-fat diet induced obese mice. Journal of Nutritional Biochemistry. 2024;124. doi: 10.1016/j.jnutbio.2023.109537 PMID:WOS:001141343600001  82. Lee H, Lim Y. The Potential Role of Myokines/Hepatokines in the Progression of Neuronal Damage in Streptozotocin and High-Fat Diet-Induced Type 2 Diabetes Mellitus Mice. Biomedicines. 2022;10(7). doi: 10.3390/biomedicines10071521 PMID:WOS:000831699300001  83. Li C. The effects and mechanisms of high plasma levels of Aβ1-42 on mononuclear macrophage in Alzheimer’s disease mouse models. Doctoral Degree, Jilin University. 2021. Available from:https://d.wanfangdata.com.cn/thesis/ChJUaGVzaXNOZXdTMjAyMzA5MDESCUQwMjU4MjMzORoIdm0yNjZ5cWU%3D  84. Li D. Circadian disruption worsens the pathological changes and inhibits the treatment effect of Nerve growth factor in adult rats after traumatic brain injury Doctoral Degree, Zhengzhou University. 2016. Available from:https://d.wanfangdata.com.cn/thesis/ChJUaGVzaXNOZXdTMjAyMzA5MDESB0Q4MzcwMDkaCGp2bGN0aDRo  85. Li G. Clinical Study of Yangxue Huoxue Formula in Treating Blood Deficiency Constipation and Its Impact on Colonic ICC in Constipation Model Mice. Doctoral Degree, China Academy of Chinese Medical Sciences. 2008. Available from:https://d.wanfangdata.com.cn/thesis/ChJUaGVzaXNOZXdTMjAyMzA5MDESCFkxODA3ODI4GghtdmFxOWZiaw%3D%3D  86. Li G. The function of cotll in mouse cortical development. Master's Degree, Northwest A&F University. 2017. Available from:https://d.wanfangdata.com.cn/thesis/ChJUaGVzaXNOZXdTMjAyMzA5MDESCFkzMjI0MzU3GghtdmFxOWZiaw%3D%3D  87. Li G. Research on Non-Invasive Ultrasound Brain Neuromodulation Methods in Small Animals. Doctoral Degree, University of Chinese Academy of Sciences. 2018. Available from:https://d.wanfangdata.com.cn/thesis/ChJUaGVzaXNOZXdTMjAyMzA5MDESCFkzNDc3NjEzGghqdmxjdGg0aA%3D%3D  88. Li G. Clinical Study of Yangxue Huoxue Formula in Treating Blood Deficiency Constipation and Its Effect on Colonic ICC in Constipation Model Mice. Doctoral Degree, Huazhong University of Science and Technology. 2018. Available from:https://d.wanfangdata.com.cn/thesis/ChJUaGVzaXNOZXdTMjAyMzA5MDESCUQwMTU0NTc5NBoIanZsY3RoNGg%3D  89. Li H. Neuroprotective effects and the mechanism study of GAS protecting the dopaminergic neurons damaged by PQ and MB Master's Degree, Air Force Medical University. 2007. Available from:https://d.wanfangdata.com.cn/thesis/ChJUaGVzaXNOZXdTMjAyMzA5MDESB0QwMzY0NDAaCG12YXE5ZmJr  90. Li J. Changes of GAP-43 expression in hippocampus of pentylenetetrazole-kindled rats with learningand memory impairment. Master's Degree, Hebei Medical University. 2011. Available from:https://d.wanfangdata.com.cn/thesis/ChJUaGVzaXNOZXdTMjAyMzA5MDESCFkxOTAwNDQ3GghqdmxjdGg0aA%3D%3D  91. Li J. A dual-enzymatically cross-linked injectable gelatin hydrogelladen with BMSCs promotes nerve function repair in mice with traumatic brain injury. Master's Degree, Zhengzhou University. 2021. Available from:https://d.wanfangdata.com.cn/thesis/ChJUaGVzaXNOZXdTMjAyMzA5MDESCFkzODQyNDEwGghkN3RsaGJuaQ%3D%3D  92. Li L. Protective effect of Mfn2 protein on oxidativeapoptosis of hippocampal neurons in epileptic rats. Master's Degree, Zhengzhou University. 2019. Available from:https://d.wanfangdata.com.cn/thesis/ChJUaGVzaXNOZXdTMjAyMzA5MDESCFkzNTYwODQ1GghqdmxjdGg0aA%3D%3D  93. Li L. Salvianic acid a sodium promotes the recovery of motor function after spinal cord injury in rats by reduces microglial inflammation through regulating mip2-vdac1-ndufa12 pathway Doctoral Degree, Qingdao University. 2020. Available from:https://d.wanfangdata.com.cn/thesis/ChJUaGVzaXNOZXdTMjAyMzA5MDESCUQwMjQ4NTc2MhoIZDd0bGhibmk%3D  94. Li N. The effect and mechanism of Ghrelin/GHS-R1a pathway on learning and memory in mice. Doctoral Degree, Qingdao University. 2019. Available from:https://d.wanfangdata.com.cn/thesis/ChJUaGVzaXNOZXdTMjAyMzA5MDESCUQwMTgwMTQ0NhoIanZsY3RoNGg%3D  95. Li P. Protective effects of MG53 combined with hUC-MSCs transplantation in a mouse model of traumatic brain injury Master's Degree, Zhengzhou University. 2018. Available from:https://d.wanfangdata.com.cn/thesis/ChJUaGVzaXNOZXdTMjAyMzA5MDESCUQwMTQ1ODc4MRoIanZsY3RoNGg%3D  96. Li X. Inhibitive Effect of Minocycline on Apoptosis of Cultured RatHippocampal Neurons Induced by Sodium Nitroprusside. Master's Degree, Zhongshan University. 2008. Available from:https://d.wanfangdata.com.cn/thesis/ChJUaGVzaXNOZXdTMjAyMzA5MDESCFkxMjkzODkwGghtdmFxOWZiaw%3D%3D  97. Li X. Protective Effects and Mechanisms ofL-3-n-Butylphthalide on Vascular Dementia Rats. Master's Degree, China Medical University. 2012. Available from:https://d.wanfangdata.com.cn/thesis/ChJUaGVzaXNOZXdTMjAyMzA5MDESCFkyMDkyMjA5GghkN3RsaGJuaQ%3D%3D  98. Li X. A Dissertation Submitted to Huazhong University of Science and Technology for the Degree of Doctor of Medicine. Doctoral Degree, Huazhong University of Science and Technology. 2021. Available from:https://d.wanfangdata.com.cn/thesis/ChJUaGVzaXNOZXdTMjAyMzA5MDESCUQwMjY4NzUzMRoIdm0yNjZ5cWU%3D  99. Li X. Study on the Molecular Mechanism of Hippocampal Injury and the Intervention of D-Pinitol in Diabetic Mice Based on Multi-Omics. Doctoral Degree, Shandong University. 2024. Available from:https://d.wanfangdata.com.cn/thesis/ChhUaGVzaXNOZXdTMjAyNDA5MjAxNTE3MjUSCFk0MzYxOTU0GghscW1xaXRrcA%3D%3D  100. Li X, Lang X, Zhang X. Treadmill Exercise Activates the PI3K/Akt Signaling Pathway to Reduce Aβ Deposition in APP/PS1 Mice. Proceedings of the 11th National Sports Science Conference; Nanjing, China2019. p. 7403-4.  101. Li Y. The positive effects of Venlafaxine on Cognitive deficiency and Cortical Neuroplasticity in Vascular Dementia Rats Master's Degree, Guangzhou Medical University. 2015. Available from:https://d.wanfangdata.com.cn/thesis/ChJUaGVzaXNOZXdTMjAyMzA5MDESB0Q3MTUxMzgaCHZtMjY2eXFl  102. Li Y. Based on the PI3K/AKT/mTOR pathway on autophagy exploring the intervention of the Bushen Jianpi Kaixin formula in rats with AD. Master's Degree, Hubei University of Chinese Medicine. 2022. Available from:https://d.wanfangdata.com.cn/thesis/ChJUaGVzaXNOZXdTMjAyMzA5MDESCUQwMjY4ODY5NBoIZDd0bGhibmk%3D  103. Li Y. Effects of ferulic acid on orthodontic tooth movement in rats and osteogenic differentiation of human periodontal ligament stem cells. Master's degree, Shandong University. 2024. Available from:https://d.wanfangdata.com.cn/thesis/ChhUaGVzaXNOZXdTMjAyNDA5MjAxNTE3MjUSCFk0MzYwNzM4GghscW1xaXRrcA%3D%3D  104. Li Z. Changes of Arc/Arg3.1 and GluR1 in hippocampus of chronicepileptic rats and the impact of memantine. Master's Degree, Hebei Medical University. 2010. Available from:https://d.wanfangdata.com.cn/thesis/ChJUaGVzaXNOZXdTMjAyMzA5MDESCFkxODAwMjQxGghqdmxjdGg0aA%3D%3D  105. Lin F. 1H-proton Magnetic Resonance Spectroscopy Features of Alzheimer's Disease Model in Rats Master's Degree, Nanjing Medical University. 2018. Available from:https://d.wanfangdata.com.cn/thesis/ChJUaGVzaXNOZXdTMjAyMzA5MDESCUQwMTU1MDgxMBoIZDd0bGhibmk%3D  106. Lin Z. Effects of transient receptor potential channel 7 (TRPM7) on the learning and memory deficits induced by amyloid beta-peptide(25-35) in rats and its preliminary mechanism research Master's Degree, Anhui Medical University. 2013. Available from:https://d.wanfangdata.com.cn/thesis/ChJUaGVzaXNOZXdTMjAyMzA5MDESB0QzODczMjMaCGQ3dGxoYm5p  107. Liu C. The effect and mechanism of CX3CL1/CX3CR 1 in ALS mice model Doctoral Degree, Hebei Medical University. 2019. Available from:https://d.wanfangdata.com.cn/thesis/ChJUaGVzaXNOZXdTMjAyMzA5MDESCUQwMTgzOTA3MxoIZDd0bGhibmk%3D  108. Liu H. Mouse Model of Pure Autonomic Failure Induced bya-synuclein Fibrils Seeding in Stellate Ganglion and the Underlying Mechanism. Master's Degree, Zhengzhou University. 2020. Available from:https://d.wanfangdata.com.cn/thesis/ChJUaGVzaXNOZXdTMjAyMzA5MDESCUQwMTk4MDA1NhoIanZsY3RoNGg%3D  109. Liu J. Anti-aging Study of Ginseng Extract in Mouse Model. Master's Degree, Nanchang University. 2022. Available from:https://d.wanfangdata.com.cn/thesis/ChJUaGVzaXNOZXdTMjAyMzA5MDESCUQwMjc5OTI2NRoIZDd0bGhibmk%3D  110. Liu K. Protective effect of Astragalus injection on systemic inflammatory on brain injury LPS induced in C57 Mice. Master's Degree, Southwest University. 2018. Available from:https://d.wanfangdata.com.cn/thesis/ChJUaGVzaXNOZXdTMjAyMzA5MDESCUQwMTUyODk1ORoIanZsY3RoNGg%3D  111. Liu L. The Effect of Xianling Gubao Capsule at Different DosageSchedules on Bone Density in Rats with Osteoporosis. Master's Degree, Hebei Medical University. 2010. Available from:https://d.wanfangdata.com.cn/thesis/ChJUaGVzaXNOZXdTMjAyMzA5MDESCFkxOTIwMjU0GghqdmxjdGg0aA%3D%3D  112. Liu L. The Impact of hUC-MSCs on Learning and Memory in Neonatal Rats with Hypoxic-Ischemic Brain Injury and the Preliminary Exploration of the Underlying Mechanism. Master's Degree, Hubei University of Medicine. 2021. Available from:https://d.wanfangdata.com.cn/thesis/ChJUaGVzaXNOZXdTMjAyMzA5MDESCUQwMjUwMTQ4MxoIZDd0bGhibmk%3D  113. Liu Q. Building rats model of radiation braininjury and study the expression changes of P35 and P25 in hippocampal neurons. Master's Degree, Southern Medical University. 2009. Available from:https://d.wanfangdata.com.cn/thesis/ChJUaGVzaXNOZXdTMjAyMzA5MDESCFkxNTUzNDY5GghtdmFxOWZiaw%3D%3D  114. Liu S. The effects of NBP on AR expression in the mice model of ALS. Master's Degree, Hebei Medical University. 2013. Available from:https://d.wanfangdata.com.cn/thesis/ChJUaGVzaXNOZXdTMjAyMzA5MDESCFkyMzM3NjI3GghqdmxjdGg0aA%3D%3D  115. Liu Y. The study of toxicologic effects of BP897 in Tourette syndrome model mice. Master's Degree, Southwest Medical University. 2011. Available from:https://d.wanfangdata.com.cn/thesis/ChJUaGVzaXNOZXdTMjAyMzA5MDESCFkxODg5MjQwGghtdmFxOWZiaw%3D%3D  116. Liu Y. Mechanism of Floralozone improving cognitive dysfunction in vascular dementia rats by reducing TRPM2 expression and activating NMDAR signaling pathway. Master's Degree, Xinxiang Medical University. 2022. Available from:https://d.wanfangdata.com.cn/thesis/ChJUaGVzaXNOZXdTMjAyMzA5MDESCUQwMjgwNTY3NRoIdm0yNjZ5cWU%3D  117. Longo S, Messi ML, Wang Z-M, Meeker W, Delbono O. Accelerated sarcopenia precedes learning and memory impairments in the P301S mouse model of tauopathies and Alzheimer's disease. Journal of Cachexia Sarcopenia and Muscle. 2024;15(4):1358-75. doi: 10.1002/jcsm.13482 PMID:WOS:001206494300001  118. Lou Y. Preliminary Exploration of the Impact of Different Types of Epileptic Seizures on Cognitive Function in Rats and the Underlying Mechanisms. Doctoral Degree, Hebei Medical University. 2006. Available from:https://d.wanfangdata.com.cn/thesis/ChJUaGVzaXNOZXdTMjAyMzA5MDESB1k5NzAwMjQaCGQ3dGxoYm5p  119. Lu Y. Cognitive Impairment in Offspring of Electrically Kindled Epileptic Pregnant Rats and the Neuroprotective Effects of Astaxanthin. Doctoral Degree, Hebei Medical University. 2015. Available from:https://d.wanfangdata.com.cn/thesis/ChJUaGVzaXNOZXdTMjAyMzA5MDESCFkyNzg0NjI3GghkN3RsaGJuaQ%3D%3D  120. Lu Y. Loss of GCN5 increase neuronal apoptosis and analysis of 1H-magnetic resonance spectroscopy in subarachnoid hemorrhage models following rats Master's Degree, Guangzhou Medical University. 2017. Available from:https://d.wanfangdata.com.cn/thesis/ChJUaGVzaXNOZXdTMjAyMzA5MDESCUQwMjE1NjY3NRoIbXZhcTlmYms%3D  121. Luo Y. Retrospective Clinical and Animal Experimental Study onErshiwuwei Coral Pill in Treating Epilepsy. Master's Degree, Southern Medical University. 2012. Available from:https://d.wanfangdata.com.cn/thesis/ChJUaGVzaXNOZXdTMjAyMzA5MDESCFkyMjU2OTM2GghqdmxjdGg0aA%3D%3D  122. Lv M. Effects of quercetin intervention on cognition function inAPP/PS1 mice under different vitamin D status. Master's Degree, Suzhou University. 2019. Available from:https://d.wanfangdata.com.cn/thesis/ChJUaGVzaXNOZXdTMjAyMzA5MDESCFkzNjUzNTIyGghkN3RsaGJuaQ%3D%3D  123. Lv S. Simvastatin augments myelination in neonatal rat ofhypoxic-ischemic brain damage. Master's Degree, Dalian Medical University. 2007. Available from:https://d.wanfangdata.com.cn/thesis/ChJUaGVzaXNOZXdTMjAyMzA5MDESCFkxMDQ0MTM0GghqdmxjdGg0aA%3D%3D  124. Lv Y. Alterations of Brain-derived neurotrophic factor, tyrosine relatedreceptor kinase B and cAMP response element binding protein in thehippocampus of Gulf War Syndrome rats. Master's Degree, Dalian Medical University. 2011. Available from:https://d.wanfangdata.com.cn/thesis/ChJUaGVzaXNOZXdTMjAyMzA5MDESCFkyMDI5Mzg1GghqdmxjdGg0aA%3D%3D  125. Ma F. Evaluation of the effects of 17β-trenbolone on hippocampal neurons and the underlying mechanisms Doctoral Degree, Shandong Normal University. 2014. Available from:https://d.wanfangdata.com.cn/thesis/ChJUaGVzaXNOZXdTMjAyMzA5MDESB0Q1MzQ1NTcaCGQ3dGxoYm5p  126. Ma J. Role of RARa in ATRA-induced Klf4 expression in vascularsmooth muscle cells. Doctoral Degree, Hebei Medical University. 2012. Available from:https://d.wanfangdata.com.cn/thesis/ChJUaGVzaXNOZXdTMjAyMzA5MDESCFkyMTA1MjI2GghqdmxjdGg0aA%3D%3D  127. Ma M. Correlation between NF-H expression in the corpus callosum and cognitive dysfunction in experimental diabetic rat model. Master's Degree, Hebei Medical University. 2012. Available from:https://d.wanfangdata.com.cn/thesis/ChJUaGVzaXNOZXdTMjAyMzA5MDESCFkyMTA1MjQyGghkN3RsaGJuaQ%3D%3D  128. Ma Y. Research ofrepeated Aβ3-10 plasmid vaccine immunetherapy Tg-APPswe/PSENldE9 mouse. Doctoral Degree, China Medical University. 2010. Available from:https://d.wanfangdata.com.cn/thesis/ChJUaGVzaXNOZXdTMjAyMzA5MDESCFkxODkzMzYwGghkN3RsaGJuaQ%3D%3D  129. Mao Y. Impact of Dexmedetomidine Combined with Mild Hypothermia on Cognitive Function in Rats with Sepsis-Related Encephalopathy. Master's Degree, Nanchang University. 2019. Available from:https://d.wanfangdata.com.cn/thesis/ChJUaGVzaXNOZXdTMjAyMzA5MDESCUQwMTc2NDAyMhoIZDd0bGhibmk%3D  130. Ni X. Lacticaseibacillus rhamnosus alleviates Irritable Bowel Syndrome and Chronic Fatigue Syndrome: strain screening and functional evaluation. Master's degree, Shandong University. 2024. Available from:https://d.wanfangdata.com.cn/thesis/ChhUaGVzaXNOZXdTMjAyNDA5MjAxNTE3MjUSCFk0MzY0NDk3GghscW1xaXRrcA%3D%3D  131. Pei F. Treadmill exercise affects energy metabolism-relatedproteins in the brain of cerebral palsy model mice andimproves exercise function. Master's Degree, Zhengzhou University. 2021. Available from:https://d.wanfangdata.com.cn/thesis/ChJUaGVzaXNOZXdTMjAyMzA5MDESCFkzODQyNjY1GghkN3RsaGJuaQ%3D%3D  132. Qi X. The role of Sodium valproate in the regeneration after the opticnerve injury in rats and the effects on BDNF and GAP-43. Master's Degree, Hebei Medical University. 2014. Available from:https://d.wanfangdata.com.cn/thesis/ChJUaGVzaXNOZXdTMjAyMzA5MDESCFkyNTgzMzU5GghqdmxjdGg0aA%3D%3D  133. Qiao L. Effects of Qidantongmai Tablet on vascular cognitive impairment of rats with chronic cerebral ischemia Master's Degree, Air Force Medical University. 2012. Available from:https://d.wanfangdata.com.cn/thesis/ChJUaGVzaXNOZXdTMjAyMzA5MDESB0QyMjEwNzQaCGQ3dGxoYm5p  134. Qin Y. Ex Vivo Spectroscopy Study on the Neural Mechanisms of Depression in Adult Rats Exposed to Early-Life Stress. Master's Degree, Wuhan Institute of Physics and Mathematics (WIPM) of Chinese Academy of Sciences. 2009. Available from:https://d.wanfangdata.com.cn/thesis/ChJUaGVzaXNOZXdTMjAyMzA5MDESCFkxNjIwMTAyGghtdmFxOWZiaw%3D%3D  135. Qin Y. Effects of Butyl benzene phthalocyanine onthe expressions of homocysteine、C-responsive element banding protein andInterleukin-6 in the hippocampus of theChronic cerebral ischemia in rats. Master's Degree, Zhengzhou University. 2011. Available from:https://d.wanfangdata.com.cn/thesis/ChJUaGVzaXNOZXdTMjAyMzA5MDESCFkxOTMwMDQzGghtdmFxOWZiaw%3D%3D  136. Qu J. Study on Ang-1, Ang-2 and Tie-2 of mice's after carbonmonoxide poisoning. Master's Degree, Dalian Medical University. 2009. Available from:https://d.wanfangdata.com.cn/thesis/ChJUaGVzaXNOZXdTMjAyMzA5MDESCFkxNTU3MTk0GghtdmFxOWZiaw%3D%3D  137. Qu Y. Neuroprotective effect and mechanisms of lactoferrin on MPTP induced mice model of Parkinson's disease. Doctoral Degree, Qingdao University. 2019. Available from:https://d.wanfangdata.com.cn/thesis/ChJUaGVzaXNOZXdTMjAyMzA5MDESCUQwMTgwMTQ4MRoIanZsY3RoNGg%3D  138. Qu Z. Mechanisms of Alzheimer-like Neurodegeneration in Diabetic Rats and the Role of Lithium Chloride. Doctoral Degree, Huazhong University Of Science And Technology. 2005. Available from:https://d.wanfangdata.com.cn/thesis/ChJUaGVzaXNOZXdTMjAyMzA5MDESCEowMTMyNjc0GghkN3RsaGJuaQ%3D%3D  139. Ren M. Metabolomics Study on the Differences in Blood and Tissue Metabolism in Mice with Lactate Accumulation. Master's Degree, Wenzhou Medical University. 2020. Available from:https://d.wanfangdata.com.cn/thesis/ChJUaGVzaXNOZXdTMjAyMzA5MDESCUQwMjE0OTMwORoIZDd0bGhibmk%3D  140. Ren R. The Study of Heterotopie AllotransplantedCryopreserved Fetal Ovary in Rats. Master's Degree, Dalian Medical University. 2002. Available from:https://d.wanfangdata.com.cn/thesis/ChJUaGVzaXNOZXdTMjAyMzA5MDESB1k0NjUzMDMaCG12YXE5ZmJr  141. Ruan Q. Effects of N-acetylneuraminic acid on 2-vessels occlusion induced vascular dementia rats. Master's Degree, Guangxi Medical University. 2019. Available from:https://d.wanfangdata.com.cn/thesis/ChJUaGVzaXNOZXdTMjAyMzA5MDESCFkzNTU2NDM3GghkN3RsaGJuaQ%3D%3D  142. Sha S. Studies of the construction ofAB3-10 DNA vaccine andits effect on preventing Aβdeposition and delayingcognitive impairment in young APP/PS1 mice. Doctoral Degree, China Medical University. 2013. Available from:https://d.wanfangdata.com.cn/thesis/ChJUaGVzaXNOZXdTMjAyMzA5MDESCFkyMjk3Mzg5GghkN3RsaGJuaQ%3D%3D  143. Shen S. The space-time distribution features of cyst of Toxoplasmagondii chronic infection mice and its effect on polyaminemetabolism enzymes and neurotransmitters. Master's Degree, Southern Medical University. 2013. Available from:https://d.wanfangdata.com.cn/thesis/ChJUaGVzaXNOZXdTMjAyMzA5MDESCFkyNDA2MjQ5GghqdmxjdGg0aA%3D%3D  144. Shen Y. D-4F treatment improves neurological outcome and attenuates cardiac deficits in aged mice subject to ischemic stroke. Doctoral Degree, Tianjin Medical University. 2020. Available from:https://d.wanfangdata.com.cn/thesis/ChJUaGVzaXNOZXdTMjAyMzA5MDESCUQwMjE2NDUxORoIanZsY3RoNGg%3D  145. Shi C. Pathological Changes of Cerebral Amyloid Angiopathy andAstrocytes in Rat Models of Alzheimer's Disease. Master's Degree, Southern Medical University. 2015. Available from:https://d.wanfangdata.com.cn/thesis/ChJUaGVzaXNOZXdTMjAyMzA5MDESCFkyOTExNDMxGghqdmxjdGg0aA%3D%3D  146. Song Y. Neuroprotective Effects and Mechanisms of Rho Kinase Inhibitor Fasudil on Aβ-induced AD Rat. Doctoral Degree, Shandong University. 2011. Available from:https://d.wanfangdata.com.cn/thesis/ChJUaGVzaXNOZXdTMjAyMzA5MDESCFkyMDQ1NTE5GghkN3RsaGJuaQ%3D%3D  147. Su R. Mechanisms of Hippocampal MARCKS Expression Changes in Aβ-Induced Dementia in Elderly Rats and the Regulatory Effects of Traditional Chinese Medicine. Doctoral Degree, Beijing University of Chinese Medicine. 2010. Available from:https://d.wanfangdata.com.cn/thesis/ChJUaGVzaXNOZXdTMjAyMzA5MDESCFkxODA0MjE3GghkN3RsaGJuaQ%3D%3D  148. Su X. The Effection of Receptor For Advanced GlycationEnd Products and its ligand HMGB1 in Rat ofSevere Acute Pancreatitis. Master's Degree, Naval Medical University. 2011. Available from:https://d.wanfangdata.com.cn/thesis/ChJUaGVzaXNOZXdTMjAyMzA5MDESCFkxOTA5MzI1GghqdmxjdGg0aA%3D%3D  149. Su X. The structure observation of the blood-brain barrier and theexpression of CD146 in diabetic rats. Master's Degree, Hebei Medical University. 2012. Available from:https://d.wanfangdata.com.cn/thesis/ChJUaGVzaXNOZXdTMjAyMzA5MDESCFkyMTA1MDk4GghqdmxjdGg0aA%3D%3D  150. Sun F. Disorder of GABAergic System and Behavior in Offspring Ratswith Prenatal Infection. Master's Degree, Xinxiang Medical University. 2020. Available from:https://d.wanfangdata.com.cn/thesis/ChJUaGVzaXNOZXdTMjAyMzA5MDESCUQwMjI4MDQ3NRoIanZsY3RoNGg%3D  151. Sun R, Gaerz MC, Oeing C, Mai K, Brachs S. Accurate locomotor activity profiles of group-housed mice derived from home cage monitoring data. Frontiers in Neuroscience. 2024;18. doi: 10.3389/fnins.2024.1456307  152. Sun Y. Protective Effect of Extractive from Gastrodia elata Blume. onRat Cerebral Ischemia-Reperfusion Injury. Master's Degree, Yunnan University of Chinese Medicine. 2008. Available from:https://d.wanfangdata.com.cn/thesis/ChJUaGVzaXNOZXdTMjAyMzA5MDESCFkxNDA1NDczGghtdmFxOWZiaw%3D%3D  153. Tan W. Effects of Hypothermia and Surgical Trauma onSpatial Learning and Memory in Rats. Doctoral Degree, China Medical University. 2010. Available from:https://d.wanfangdata.com.cn/thesis/ChJUaGVzaXNOZXdTMjAyMzA5MDESCFkxNzY5NjY0GghqdmxjdGg0aA%3D%3D  154. Tian S. The experimental study of Icariin on the treatment of hypotestoidism in sub-acutely aging rat Master's Degree, Shantou University. 2007. Available from:https://d.wanfangdata.com.cn/thesis/ChJUaGVzaXNOZXdTMjAyMzA5MDESB0Q1MzI3NTAaCG12YXE5ZmJr  155. Tian X. Experimental Study on the Effects and Mechanisms of Tiantai No. 2 Formula in Ameliorating Alzheimer's Disease in Rats. Doctoral Degree, Hubei University Of Traditional Chinese Medicine. 2009. Available from:https://d.wanfangdata.com.cn/thesis/ChJUaGVzaXNOZXdTMjAyMzA5MDESCFkxNDU0NjAwGghkN3RsaGJuaQ%3D%3D  156. Tian Y. The mechanisms of pseudoginsenoside GQ improving cognitive impairment induced by chronic intermittent hypoxia in mice by regulating microglia polarization. Doctoral Degree, Jilin University. 2024. Available from:https://d.wanfangdata.com.cn/thesis/ChhUaGVzaXNOZXdTMjAyNDA5MjAxNTE3MjUSCUQwMzUwNjY4MBoIbHFtcWl0a3A%3D  157. Wang C. A Study on the Effects of Immunological Leptin and Leptin Receptor on Fat Deposition and Molecular Mechanisms in Rats. Master's Degree, South China Agricultural University. 2011. Available from:https://d.wanfangdata.com.cn/thesis/ChJUaGVzaXNOZXdTMjAyMzA5MDESCFkyMDEzODY1GghqdmxjdGg0aA%3D%3D  158. Wang D. Effects of intracerebroventricular injection of α-synuclein on Nigrostriatal system in normal mice and MPTP-intoxicated mice. Master's Degree, Qingdao University. 2019. Available from:https://d.wanfangdata.com.cn/thesis/ChJUaGVzaXNOZXdTMjAyMzA5MDESCUQwMTgwMzgzORoIanZsY3RoNGg%3D  159. Wang D. Study on the phenomenon of extrahepatic pathological angiogenesis in multiple organs of HPS rats and the effect of adenosine A3 receptor. Master's Degree, Army Medical University. 2022. Available from:https://d.wanfangdata.com.cn/thesis/ChJUaGVzaXNOZXdTMjAyMzA5MDESCUQwMjgzMzExNRoIZDd0bGhibmk%3D  160. Wang G. Study on the Neuroprotective Effects and Mechanism of Histone Deacetylase Inhibitors in Mice with Traumatic Brain Injury. Doctoral Degree, Fudan University. 2013. Available from:https://d.wanfangdata.com.cn/thesis/ChJUaGVzaXNOZXdTMjAyMzA5MDESCFkyNzA0MDM0GghtdmFxOWZiaw%3D%3D  161. Wang G. The effect of sulforaphane on cognitive function decline by modulating PI3K / AKT / GSK-3β signal pathway in diabetic rats Doctoral Degree, Hebei Medical University. 2017. Available from:https://d.wanfangdata.com.cn/thesis/ChJUaGVzaXNOZXdTMjAyMzA5MDESCUQwMTIwMTI1NhoIZDd0bGhibmk%3D  162. Wang G. Investigation on the mechanisms of voluntary exerciseimproving cognitive function in different stages ofADmice model via targeting gut microbiota and  metabonomics. Doctoral Degree, Suzhou University. 2021. Available from:https://d.wanfangdata.com.cn/thesis/ChJUaGVzaXNOZXdTMjAyMzA5MDESCFkzODg3MjA4Ggh2bTI2NnlxZQ%3D%3D  163. Wang H. Changes of Ng expression in hippocampus ofpentylenetetrazole-kindled rats with learning andmemory impairment. Master's Degree, Hebei Medical University. 2008. Available from:https://d.wanfangdata.com.cn/thesis/ChJUaGVzaXNOZXdTMjAyMzA5MDESCFkxMzUzODEyGghqdmxjdGg0aA%3D%3D  164. Wang H. The effect of alpha lipoic acid on the expression of CaMKla in the hippocampus of the chronic epileptic rat model kindled byamygdala stimulating. Master's Degree, Hebei Medical University. 2014. Available from:https://d.wanfangdata.com.cn/thesis/ChJUaGVzaXNOZXdTMjAyMzA5MDESCFkyNTgzMzQzGghqdmxjdGg0aA%3D%3D  165. Wang H. A Study on the Mechanisms of Bisphenol A-Induced Neurotoxicity Using Stem Cell-Derived Neural Network Models and Mouse Models. Doctoral Degree, University of Chinese Academy of Sciences. 2019. Available from:https://d.wanfangdata.com.cn/thesis/ChJUaGVzaXNOZXdTMjAyMzA5MDESCFkzNjAwOTE2GghqdmxjdGg0aA%3D%3D  166. Wang J. Effect of EGB on insulin sensitivity in high-fat diet rats and therelative mechanisms study. Doctoral Degree, Hebei Medical University. 2008. Available from:https://d.wanfangdata.com.cn/thesis/ChJUaGVzaXNOZXdTMjAyMzA5MDESCFkxMzU1MDg2GghqdmxjdGg0aA%3D%3D  167. Wang J. RA reduces 6-hydroxydendrobine-induced dopamine neurondegeneration in rats. Master's Degree, Qingdao University. 2010. Available from:https://d.wanfangdata.com.cn/thesis/ChJUaGVzaXNOZXdTMjAyMzA5MDESCFkyMDQzNjYxGghqdmxjdGg0aA%3D%3D  168. Wang J. The effects of lycopene on apoptosis in rats of vascular dementia. Master's Degree, Hebei Medical University. 2017. Available from:https://d.wanfangdata.com.cn/thesis/ChJUaGVzaXNOZXdTMjAyMzA5MDESCUQwMTIwMTUxNhoIZDd0bGhibmk%3D  169. Wang J. Effects of sevoflurane on the expression of bip、atf6 and chop in temporal lobe of aged rats. Master's Degree, Hebei Medical University. 2018. Available from:https://d.wanfangdata.com.cn/thesis/ChJUaGVzaXNOZXdTMjAyMzA5MDESCUQwMTUxMzgwORoIZDd0bGhibmk%3D  170. Wang K. Taurine protects spatial learning and memory ability in paraquat and maneb co-exposure mouse through inhibition of NOX2. Doctoral Degree, Dalian Medical University. 2019. Available from:https://d.wanfangdata.com.cn/thesis/ChJUaGVzaXNOZXdTMjAyMzA5MDESCUQwMjM2NTk2MhoIanZsY3RoNGg%3D  171. Wang L. The intestinal metabolism ,absorption distribution and effects on alzheimer's disease mouse models of evodia evodia extract Doctoral Degree, Jilin Agricultural University. 2019. Available from:https://d.wanfangdata.com.cn/thesis/ChJUaGVzaXNOZXdTMjAyMzA5MDESCUQwMjE3MDUzNRoIdm0yNjZ5cWU%3D  172. Wang M. The role of PI3K-AMPA receptor GluR2 signaling pathway in sevoflurane-induced POCD in aged rats. Master's Degree, Tianjin Medical University. 2014. Available from:https://d.wanfangdata.com.cn/thesis/ChJUaGVzaXNOZXdTMjAyMzA5MDESCFkyNjk4NzQ0GghqdmxjdGg0aA%3D%3D  173. Wang M. Alterations in calcium concentration and DHPR,RyRondiaphragm dysfunction in a rat model of sepsis. Doctoral Degree, China Medical University. 2017. Available from:https://d.wanfangdata.com.cn/thesis/ChJUaGVzaXNOZXdTMjAyMzA5MDESCFkzMjY4MzQ2GghqdmxjdGg0aA%3D%3D  174. Wang N. Study of Brain Metabolism in Different Stages of Diabetes Using Nuclear Magnetic Resonance Spectroscopy. Master's Degree, Wenzhou Medical University. 2014. Available from:https://d.wanfangdata.com.cn/thesis/ChJUaGVzaXNOZXdTMjAyMzA5MDESCFkyNjkxOTg1GghtdmFxOWZiaw%3D%3D  175. Wang P. The experimental exploration of the mechanism of damagedcognition of epileptic rats and the intervening effects of nimodipine. Doctoral Degree, Hebei Medical University. 2008. Available from:https://d.wanfangdata.com.cn/thesis/ChJUaGVzaXNOZXdTMjAyMzA5MDESCFkxMzU1MTAzGghkN3RsaGJuaQ%3D%3D  176. Wang Q. Melatonin recovers the period length prolonged by MK-801 inconstant darkness condition through the sleep homeostatie systemand the underlying mechanism analysis. Master's Degree, Shandong University. 2021. Available from:https://d.wanfangdata.com.cn/thesis/ChJUaGVzaXNOZXdTMjAyMzA5MDESCFkzODE3OTA4GghkN3RsaGJuaQ%3D%3D  177. Wang R. Melatonin recovers the period length prolonged by mk-801 in constant darkness condition through the sleep homeostatic system and the underlying mechanism analysis. Doctoral Degree, Hebei Medical University. 2010. Available from:https://d.wanfangdata.com.cn/thesis/ChJUaGVzaXNOZXdTMjAyMzA5MDESCFkxODAxOTE3GghqdmxjdGg0aA%3D%3D  178. Wang T. NT一4 attenuates neuroinnammation via TrkB／P13K／Foxo1 pathway after germinal matrix hemorrhage in neonatal rats. Master's Degree, Suzhou University. 2020. Available from:https://d.wanfangdata.com.cn/thesis/ChJUaGVzaXNOZXdTMjAyMzA5MDESCFkzNzcyNTQzGghqdmxjdGg0aA%3D%3D  179. Wang X. Neuroprotective Effects of Flavopiridol in a Mouse Model of Niemann-Pick Disease Type C. Master's Degree, Huazhong University of Science and Technology. 2006. Available from:https://d.wanfangdata.com.cn/thesis/ChJUaGVzaXNOZXdTMjAyMzA5MDESB1k5NjE2OTEaCG12YXE5ZmJr  180. Wang X. Mechanisms ofCerebrum Dysfunction Induced by DeepHypothemic Low Flow Cardiopulmonary Bypass in Rabbitsand the Protective Efects ofL-carnitine. Master's Degree, Army Medical University. 2006. Available from:https://d.wanfangdata.com.cn/thesis/ChJUaGVzaXNOZXdTMjAyMzA5MDESB1k5NTk1MjEaCG12YXE5ZmJr  181. Wang X. Study on the differentiation of bone marrow mesenchymal stemcells of rat induced by astragalus mongholicus and itsmechanisms. Doctoral Degree, Hebei Medical University. 2008. Available from:https://d.wanfangdata.com.cn/thesis/ChJUaGVzaXNOZXdTMjAyMzA5MDESCFkxMzU1MTQxGghtdmFxOWZiaw%3D%3D  182. Wang X. A Study on the Role of Lentivirus-Mediated TNF-α SiRNA in Neurodegeneration in a Mouse Model of Niemann-Pick Disease Type C. Doctoral Degree, Huazhong University of Science and Technology. 2009. Available from:https://d.wanfangdata.com.cn/thesis/ChJUaGVzaXNOZXdTMjAyMzA5MDESB0QwODg5MjEaCGQ3dGxoYm5p  183. Wang X. Experimental study of butylphthalide on learningand memory disorder in rats with vascular dementia. Master's Degree, Zhengzhou University. 2011. Available from:https://d.wanfangdata.com.cn/thesis/ChJUaGVzaXNOZXdTMjAyMzA5MDESCFkxOTMwMDI0GghkN3RsaGJuaQ%3D%3D  184. Wang X. The role and mechanism of rictor in spatial learning and memory impairment in Ctnnd2. Doctoral Degree, Chongqing Medical University. 2021. Available from:https://d.wanfangdata.com.cn/thesis/ChJUaGVzaXNOZXdTMjAyMzA5MDESCUQwMjU5NjI2ORoIZDd0bGhibmk%3D  185. Wang X. Neurorepair effects of human oligodendrocyteprogenitor cells and neural stem cells on developmentalbrain injury in rats. Doctoral Degree, Southern Medical University. 2022. Available from:https://d.wanfangdata.com.cn/thesis/ChJUaGVzaXNOZXdTMjAyMzA5MDESCFk0MDI3MDUzGghkN3RsaGJuaQ%3D%3D  186. Wang Y. Clearance ofAmyloid-beta in Alzheimer’s disease mouse brain andrelationship between leisure activity and cognitve impairment. Doctoral Degree, Army Medical University. 2006. Available from:https://d.wanfangdata.com.cn/thesis/ChJUaGVzaXNOZXdTMjAyMzA5MDESB1k5NTk3OTQaCGQ3dGxoYm5p  187. Wang Y. Synaptic Plasticity Changes in Rats after Cerebral Ischemia and the Role of Jiedu Tongluo Formula. Master's Degree, Beijing University of Chinese Medicine. 2006. Available from:https://d.wanfangdata.com.cn/thesis/ChJUaGVzaXNOZXdTMjAyMzA5MDESB1k4Njg1NjMaCG12YXE5ZmJr  188. Wang Y. Prior stressor exposure delays the recovery ofsurgery-induced cognitive impairment and prolongs neuroinflammation in aged rats. Doctoral Degree, China Medical University. 2017. Available from:https://d.wanfangdata.com.cn/thesis/ChJUaGVzaXNOZXdTMjAyMzA5MDESCFkzMjY4ODQ3GghkN3RsaGJuaQ%3D%3D  189. Wang Y. Effects of sleep deprivation on behavior and NO、NOS in rats and Study on TCM Syndromes of Insomnia and anxiety. Master's Degree, Shaanxi University of Chinese Medicine. 2018. Available from:https://d.wanfangdata.com.cn/thesis/ChJUaGVzaXNOZXdTMjAyMzA5MDESCUQwMTU1ODMzNxoIanZsY3RoNGg%3D  190. Wang Z. Influences of IRE1α and p-JNK expression on vascular remodeling in hypertensive rats with hyperhomocystinemia and the effect of enalapril folic acid tablets intervention Master's Degree, Air Force Medical University. 2017. Available from:https://d.wanfangdata.com.cn/thesis/ChJUaGVzaXNOZXdTMjAyMzA5MDESCUQwMTI3Mzk1MBoIanZsY3RoNGg%3D  191. Wang Z. Biological Effects and Mechanisms of GDF11 on Neural Stem Cells in Mice. Doctoral Degree, Peking Union Medical College. 2022. Available from:https://d.wanfangdata.com.cn/thesis/ChJUaGVzaXNOZXdTMjAyMzA5MDESCFk0MDUxOTkyGghkN3RsaGJuaQ%3D%3D  192. Wei W. Mechanism Study on Hypothyroxinemia Induced byMild lodine Deficiency during Pregnant and LactationImpairing Neurodevelopment and Long-termPlasticity in the Hippocampus of Rat Offspring. Master's Degree, China Medical University. 2013. Available from:https://d.wanfangdata.com.cn/thesis/ChJUaGVzaXNOZXdTMjAyMzA5MDESCFkyMjk2Nzg1GghtdmFxOWZiaw%3D%3D  193. Wen S. The Relationship Between Neural Stem Cells and Blood Vessels in the Mouse Brain. Master's Degree, Henan University. 2011. Available from:https://d.wanfangdata.com.cn/thesis/ChJUaGVzaXNOZXdTMjAyMzA5MDESB0QxNDYxMjgaCGp2bGN0aDRo  194. Wu S. Protective role and mechanism of Glycyrrhizic acid in neonatal mice with hypoxic ischemic brain injury based on Rac1/STAT3 signaling. Master's Degree, Southeast University. 2021. Available from:https://d.wanfangdata.com.cn/thesis/ChJUaGVzaXNOZXdTMjAyMzA5MDESCUQwMjYyNjM1NRoIZDd0bGhibmk%3D  195. Xi L. Thyroid hormone Reduces Hippocampal Apoptosisin Rats with Chronic Cerebral Ischemia via Upregulating the Expression of Bcl-2. Master's Degree, Southern Medical University. 2013. Available from:https://d.wanfangdata.com.cn/thesis/ChJUaGVzaXNOZXdTMjAyMzA5MDESCFkyNDA2MTM3GghkN3RsaGJuaQ%3D%3D  196. Xian Y. Effect of umbilical cord mesenchymal stem cells combined with resveratrol on type 1 diabetes mellitus renal protection in mice. Doctoral Degree, Qingdao University. 2018. Available from:https://d.wanfangdata.com.cn/thesis/ChJUaGVzaXNOZXdTMjAyMzA5MDESCUQwMTgwMTQxORoIanZsY3RoNGg%3D  197. Xie K. Protective Mechanism of Cerebral Ischemia-reperfusionInjury in Rats during Peroperative Period. Doctoral Degree, Shandong University. 2019. Available from:https://d.wanfangdata.com.cn/thesis/ChJUaGVzaXNOZXdTMjAyMzA5MDESCFkzNjc2NTAyGghqdmxjdGg0aA%3D%3D  198. Xie T. Effect of Methane on Neuroinflammatory Injury and ItsPotential Mechanism in Mice. Doctoral Degree, Naval Medical University. 2020. Available from:https://d.wanfangdata.com.cn/thesis/ChJUaGVzaXNOZXdTMjAyMzA5MDESCFkzNzE2MzM0GghqdmxjdGg0aA%3D%3D  199. Xu B. Exploring the Molecular Mechanisms of Anesthetics on Cognitive Function in Aging Rats Through Inflammation and Oxidative Stress. Doctoral Degree, Shandong University. 2016. Available from:https://d.wanfangdata.com.cn/thesis/ChJUaGVzaXNOZXdTMjAyMzA5MDESCFkzMTU2NTA4GghkN3RsaGJuaQ%3D%3D  200. Xu H. Brain Imaging Study of Schizophrenia Animal Models and Long-Term Insulin-Treated Type 1 Diabetes Animal Models. Doctoral Degree, University of Chinese Academy of Sciences. 2022. Available from:https://d.wanfangdata.com.cn/thesis/ChJUaGVzaXNOZXdTMjAyMzA5MDESCFk0MDIyODk5GghkN3RsaGJuaQ%3D%3D  201. Xu J. Oxiracetam ameliorates cognitive deficits through activating PI3K/Akt signaling pathway in vascular dementia rats. Doctoral Degree, Hebei Medical University. 2016. Available from:https://d.wanfangdata.com.cn/thesis/ChJUaGVzaXNOZXdTMjAyMzA5MDESB0Q4NDM4NTUaCHZtMjY2eXFl  202. Xu Q. Observation of basilar membrane in the choroid plexus oflateral ventricle and cerebral small blood vessel andinvestigation of the expressions of a-SMA and CD31 in thecerebral small blood vessel from diabetic rats. Master's Degree, Hebei Medical University. 2011. Available from:https://d.wanfangdata.com.cn/thesis/ChJUaGVzaXNOZXdTMjAyMzA5MDESCFkxOTAwNDQ2GghtdmFxOWZiaw%3D%3D  203. Xu S. Effect of Modified Taohe Chengqi Decoction on preventing diabetic macroangiopathy in rats based on AGEs-RAGE pathway. Doctoral Degree, Guangzhou University of Chinese Medicine. 2017. Available from:https://d.wanfangdata.com.cn/thesis/ChJUaGVzaXNOZXdTMjAyMzA5MDESCFkzMjMxODY3GghqdmxjdGg0aA%3D%3D  204. Xu X. Establishment of a Mouse Model and Study on the Pathogenesis of Idiopathic Basal Ganglia Calcification. Doctoral Degree, Huazhong University of Science and Technology. 2019. Available from:https://d.wanfangdata.com.cn/thesis/ChJUaGVzaXNOZXdTMjAyMzA5MDESCUQwMjAzMjIwNxoIanZsY3RoNGg%3D  205. Xu Y. The influence of the treatment and the expresstion of Nrf2 in EAE mice which were treated by alpha lipoic acid Master's Degree, Hebei Medical University. 2016. Available from:https://d.wanfangdata.com.cn/thesis/ChJUaGVzaXNOZXdTMjAyMzA5MDESB0Q4NDQ0NjUaCGp2bGN0aDRo  206. Xue H. A study on gangliside improving the cognitive function inneonatal rat model of cerebral hypoxic ischemia. Master's Degree, Zhengzhou University. 2010. Available from:https://d.wanfangdata.com.cn/thesis/ChJUaGVzaXNOZXdTMjAyMzA5MDESCFkxODMyODE1GghkN3RsaGJuaQ%3D%3D  207. Xue Y. Effects and Mechanisms ofVitamin D; on AD-likeDegeneration and Behavior on APP/PS1 Mice. Doctoral Degree, Zhengzhou University. 2021. Available from:https://d.wanfangdata.com.cn/thesis/ChJUaGVzaXNOZXdTMjAyMzA5MDESCFk0MDUxNTg1GghkN3RsaGJuaQ%3D%3D  208. Yang D. The impacts of a novel ATP-sensitive potassium channel opener,iptakalim, on restraint-induced depression-like behavior in mice. Master's Degree, Nanjing Medical University. 2017. Available from:https://d.wanfangdata.com.cn/thesis/ChJUaGVzaXNOZXdTMjAyMzA5MDESCFkzMjQ1MDE0GghqdmxjdGg0aA%3D%3D  209. Yang X. Experimental study of establishing the mouse models of acute epilepsy induced by kainic acid Master's Degree, Air Force Medical University. 2008. Available from:https://d.wanfangdata.com.cn/thesis/ChJUaGVzaXNOZXdTMjAyMzA5MDESB0QyMTg2NDgaCGp2bGN0aDRo  210. Yang Y. Effects of CD200 in acute phase of experimental cerebral infarction mice and underlying mechanisms. Doctoral Degree, Hebei Medical University. 2019. Available from:https://d.wanfangdata.com.cn/thesis/ChJUaGVzaXNOZXdTMjAyMzA5MDESCUQwMTgzOTA0NRoIanZsY3RoNGg%3D  211. Yao G. Effect of GM1 on PI3K/Akt signaling pathway in the hippocampal neurone of rat with mild hypothermic cardiopulmonary bypass Master's Degree, Air Force Medical University. 2012. Available from:https://d.wanfangdata.com.cn/thesis/ChJUaGVzaXNOZXdTMjAyMzA5MDESB0QyMjA5MzgaCG12YXE5ZmJr  212. Yin J. Neuroprotective effects of Dl-3-n-Butylphthalide in a transgenicmouse model of familial amyotrophic lateral sclerosis. Master's Degree, Hebei Medical University. 2012. Available from:https://d.wanfangdata.com.cn/thesis/ChJUaGVzaXNOZXdTMjAyMzA5MDESCFkyMTA1MTAzGghqdmxjdGg0aA%3D%3D  213. Yu A. The effects of severe traumatic brain injury repairon spleen intercepted circulating endothelial progenitorcells and plateau environment. Doctoral Degree, Army Medical University. 2015. Available from:https://d.wanfangdata.com.cn/thesis/ChJUaGVzaXNOZXdTMjAyMzA5MDESCFkyODgwNzg0GghtdmFxOWZiaw%3D%3D  214. Yu H. The research of Effeet on Daixiean ofTCM on MetabolicSyndrome and Its detriment of neuron, microvessel in Aged Rat. Doctoral Degree, Beijing University of Chinese Medicine. 2006. Available from:https://d.wanfangdata.com.cn/thesis/ChJUaGVzaXNOZXdTMjAyMzA5MDESB1k4Njg3ODIaCG12YXE5ZmJr  215. Yu J. Study on the transcriptome sequencing and pedigree of CD133 positive neural stem cells from central spinal canal in C57 mice. Master's Degree, Nanchang University. 2018. Available from:https://d.wanfangdata.com.cn/thesis/ChJUaGVzaXNOZXdTMjAyMzA5MDESCUQwMTUyMjI2OBoIanZsY3RoNGg%3D  216. Yu L. Effects of preconditioning with sevoflurane inhalation onpostoperative cognition disorders of aged rats. Master's Degree, Hebei Medical University. 2014. Available from:https://d.wanfangdata.com.cn/thesis/ChJUaGVzaXNOZXdTMjAyMzA5MDESCFkyNTgyNDA1GghkN3RsaGJuaQ%3D%3D  217. Yu Q. The protective effect and mechanism of exogenous hydrogen sulfide on aging cerebral ischemia/ reperfusion injury Doctoral Degree, Air Force Medical University. 2018. Available from:https://d.wanfangdata.com.cn/thesis/ChJUaGVzaXNOZXdTMjAyMzA5MDESCUQwMTQ4MTkwOBoIanZsY3RoNGg%3D  218. Yu S. SIRT1 expression and the effects of resveratrol on SIRT1 in themotor cortex and lumbar spinal cord of SOD1-G93A mice. Master's Degree, Hebei Medical University. 2014. Available from:https://d.wanfangdata.com.cn/thesis/ChJUaGVzaXNOZXdTMjAyMzA5MDESCFkyNTgyNTQzGghqdmxjdGg0aA%3D%3D  219. Yuan H. Cognitive Dysfunction Associated with Synapse—Related Proteins Expression in Rats Following Subarachnoid Hemorrhage. Doctoral Degree, Shandong University. 2011. Available from:https://d.wanfangdata.com.cn/thesis/ChJUaGVzaXNOZXdTMjAyMzA5MDESCFkyMDQ1NTIyGghkN3RsaGJuaQ%3D%3D  220. Yuan X. Mechanism of pathological a-synuclein spreading via theurogenital nerves initiating multiple system atrophy-likesyndromes in mice. Master's Degree, Zhengzhou University. 2019. Available from:https://d.wanfangdata.com.cn/thesis/ChJUaGVzaXNOZXdTMjAyMzA5MDESCFkzNTYwNjczGghqdmxjdGg0aA%3D%3D  221. Zang Y. Protective Effects of Total Salvianolic Acids and DL-0108 on Multi-Infarct Dementia (MID) Rat Models. Master's Degree, Peking Union Medical College. 2005. Available from:https://d.wanfangdata.com.cn/thesis/ChJUaGVzaXNOZXdTMjAyMzA5MDESB1k3NjIwNTQaCGQ3dGxoYm5p  222. Zeng M. (1) Effect and mechanism of NAMPT inhibitor FK866 on improving cognitive function in aged mice  (2) Roles of NAMPT in hippocampal neurons on cognitive function in mice Master's Degree, Zhejiang University. 2020. Available from:https://d.wanfangdata.com.cn/thesis/ChJUaGVzaXNOZXdTMjAyMzA5MDESCUQwMjkzMjg0NRoIdm0yNjZ5cWU%3D  223. Zeng P. The proteomic basis of brain damage in alcoholic hypertensive rats and the important role of matrix metalloproteinase 2 Doctoral Degree, Huazhong University Of Science And Technology. 2019. Available from:https://d.wanfangdata.com.cn/thesis/ChJUaGVzaXNOZXdTMjAyMzA5MDESCUQwMTc4NTQzNxoIZDd0bGhibmk%3D  224. Zhang F. Dissecting the effect of GHS-R1a deficiency on context memory linking of mice under control and neuro-inflammatory states Master's Degree, Qingdao University. 2021. Available from:https://d.wanfangdata.com.cn/thesis/ChJUaGVzaXNOZXdTMjAyMzA5MDESCUQwMjQ4NDUyMxoIZDd0bGhibmk%3D  225. Zhang H. Study on Effects of Three Supplementationson Morphology and Function of Soleus in Hindlimb-Unloading Rats. Master's Degree, Northwest University. 2007. Available from:https://d.wanfangdata.com.cn/thesis/ChJUaGVzaXNOZXdTMjAyMzA5MDESCFkxMDkwODcyGghqdmxjdGg0aA%3D%3D  226. Zhang H. The effects of NBP on the lifespan and CDK5 P35 expression in the mice model of ALS. Master's Degree, Hebei Medical University. 2012. Available from:https://d.wanfangdata.com.cn/thesis/ChJUaGVzaXNOZXdTMjAyMzA5MDESCFkyMTA1MTAyGghqdmxjdGg0aA%3D%3D  227. Zhang H. Relationship Between Regional Changes in Rat Brain Metabolism, Cellular Architecture, and Disease Susceptibility. Doctoral Degree, University of Chinese Academy of Sciences. 2018. Available from:https://d.wanfangdata.com.cn/thesis/ChJUaGVzaXNOZXdTMjAyMzA5MDESCFkzNjAwMzY2GghqdmxjdGg0aA%3D%3D  228. Zhang H. Establishment of an animal model of ischemic and hypoxic encephalopathy accompanied with temporal lobe epilepsy. Master's Degree, Dalian Medical University. 2019. Available from:https://d.wanfangdata.com.cn/thesis/ChJUaGVzaXNOZXdTMjAyMzA5MDESCUQwMTczMjI0NBoIanZsY3RoNGg%3D  229. Zhang L. Physiological Testosterone Retards urine Cardiomyocyte Aging:an Effect Mediated via Androgen Receptor-Independent Pathway. Doctoral Degree, Southern Medical University. 2011. Available from:https://d.wanfangdata.com.cn/thesis/ChJUaGVzaXNOZXdTMjAyMzA5MDESCFkxOTk3NzkxGghqdmxjdGg0aA%3D%3D  230. Zhang L. Study on the mechanism of long term memorydamaged by the pathway of cAMP-PKA-CREB inhippocampus in rats exposed to Aluminum. Doctoral Degree, China Medical University. 2011. Available from:https://d.wanfangdata.com.cn/thesis/ChJUaGVzaXNOZXdTMjAyMzA5MDESCFkyMjk3NjcyGghtdmFxOWZiaw%3D%3D  231. Zhang S. The Role of Pl3K/Akt SignalTransduction Pathway In theCognitive Impairment of Epileptic Rats and The InterveningEffects of Donepezil. Doctoral Degree, Hebei Medical University. 2009. Available from:https://d.wanfangdata.com.cn/thesis/ChJUaGVzaXNOZXdTMjAyMzA5MDESCFkxNjM3NzExGgh2bTI2NnlxZQ%3D%3D  232. Zhang S. Influence of triptolide on drebrin and cofilin expression in the hippocampus of rats after injection of β-amyloid. Master's Degree, Nanchang University. 2014. Available from:https://d.wanfangdata.com.cn/thesis/ChJUaGVzaXNOZXdTMjAyMzA5MDESB0Q1NTYzOTQaCGp2bGN0aDRo  233. Zhang T. A Study on FUS Point Mutations in Rats Related to Amyotrophic Lateral Sclerosis/Frontotemporal Dementia. Doctoral Degree, University of Chinese Academy of Sciences. 2018. Available from:https://d.wanfangdata.com.cn/thesis/ChJUaGVzaXNOZXdTMjAyMzA5MDESCFkzNjgzNjYwGghkN3RsaGJuaQ%3D%3D  234. Zhang X. Study on expression ofTSP-1 and TGF-βl ofdiabetic complications in diabetie rats. Doctoral Degree, Zhejiang University. 2011. Available from:https://d.wanfangdata.com.cn/thesis/ChJUaGVzaXNOZXdTMjAyMzA5MDESCFkyMTE5MDgwGghtdmFxOWZiaw%3D%3D  235. Zhang Z. The effects of hypoxia on the proliferation of BMSCsextracted from C57 mice of different age. Master's Degree, Southern Medical University. 2015. Available from:https://d.wanfangdata.com.cn/thesis/ChJUaGVzaXNOZXdTMjAyMzA5MDESCFkyOTExNDU2GghqdmxjdGg0aA%3D%3D  236. Zhao J. Effects of serum amyloid Pcomponent on atherosclerosis in ApoE mice. Master's Degree, Southern Medical University. 2017. Available from:https://d.wanfangdata.com.cn/thesis/ChJUaGVzaXNOZXdTMjAyMzA5MDESCFkzMjgxMDU4GghqdmxjdGg0aA%3D%3D  237. Zhao T. Effect of valsartan on age-rage, oxidative stress and endodermic function in brain tissue of diabetic rats. Master's Degree, Shandong University. 2007. Available from:https://d.wanfangdata.com.cn/thesis/ChJUaGVzaXNOZXdTMjAyMzA5MDESCFkxMDY1ODg4GghtdmFxOWZiaw%3D%3D  238. Zhao W. Protective effects of magnolol on the PTZ-kindled rats and the expression changes of bcl-2 ,bax in the hippocampus. Master's Degree, Hebei Medical University. 2015. Available from:https://d.wanfangdata.com.cn/thesis/ChJUaGVzaXNOZXdTMjAyMzA5MDESCFkyNzg1ODQzGghtdmFxOWZiaw%3D%3D  239. Zhao X. Effects of sevoflurane postconditioning on cognition function in myocardial ischemia-reperfusion rats. Master's Degree, Shanxi Medical University. 2022. Available from:https://d.wanfangdata.com.cn/thesis/ChJUaGVzaXNOZXdTMjAyMzA5MDESCUQwMjc3MjYxMBoIdm0yNjZ5cWU%3D  240. Zheng H. The effect and regulation of α-DG glycosylation on chronic social defeat induced depressive-like behaviors of mice. Doctoral Degree, Huazhong University of Science and Technology. 2018. Available from:https://d.wanfangdata.com.cn/thesis/ChJUaGVzaXNOZXdTMjAyMzA5MDESCUQwMTU0NjI1MhoIanZsY3RoNGg%3D  241. Zhou N. Co-transplantation of neural stem cells and olfactory ensheathing cells promotes neurological function repair and its mechanism in PD rats Master's Degree, Qingdao University. 2019. Available from:https://d.wanfangdata.com.cn/thesis/ChJUaGVzaXNOZXdTMjAyMzA5MDESCUQwMTgwMzg0MxoIanZsY3RoNGg%3D  242. Zhou Y. Neuroprotective Effect of Modified Dihuang Yinzi on Vascular Dementia Model Rats Master's Degree, Hebei University of Chinese Medicine. 2020. Available from:https://d.wanfangdata.com.cn/thesis/ChJUaGVzaXNOZXdTMjAyMzA5MDESCUQwMjE3MjAyORoIZDd0bGhibmk%3D  243. Zhou Y. Diffusion of Pathological TDP-43 Along Corticospinal TractAxons Induces ALS Like Phenotype in Atg5 + Mice. Master's Degree, Zhengzhou University. 2021. Available from:https://d.wanfangdata.com.cn/thesis/ChJUaGVzaXNOZXdTMjAyMzA5MDESCFkzODQzNDg5GghkN3RsaGJuaQ%3D%3D  244. Zhu F. The effects of berberine chloride on the spatialmemory impairment, microglia activation andinflammatory factors' expression in the ratmodel of Alzheimer's disease. Doctoral Degree, Sun Yat-sen University. 2007. Available from:https://d.wanfangdata.com.cn/thesis/ChJUaGVzaXNOZXdTMjAyMzA5MDESCFkxMDg3MjYxGghtdmFxOWZiaw%3D%3D  245. Zhu F. The animal research on the role of microglia activationin pathogenesis of negative symptoms of schizophrenia. Doctoral Degree, Central South University. 2014. Available from:https://d.wanfangdata.com.cn/thesis/ChJUaGVzaXNOZXdTMjAyMzA5MDESCFkyNjg0MDQ1GghtdmFxOWZiaw%3D%3D  246. Zhu M. L-carnitine ameliorates muscle wasting of cancer cachexia through the AKT/p70S6K and AKT/FOXO3a pathways in tumor-bearing mice Master's Degree, Army Medical University. 2019. Available from:https://d.wanfangdata.com.cn/thesis/ChJUaGVzaXNOZXdTMjAyMzA5MDESCUQwMTk5NTk1NxoIdm0yNjZ5cWU%3D  247. Zou Y. The Impact of Ischemic Postconditioning on Cerebral Ischemia-Reperfusion Injury in Rats. Doctoral Degree, Naval Medical University. 2008. Available from:https://d.wanfangdata.com.cn/thesis/ChJUaGVzaXNOZXdTMjAyMzA5MDESCFkxMzc0MjMzGghtdmFxOWZiaw%3D%3D |
| **Reviews** | 174 | 1. Cao X, Yang B, Cong B, Liu H. The progress of treatment for brain metastases of triple-negative breast cancer. CHINA ONCOLOGY. 2024;34(8):777-84. doi: 10.19401/j.cnki.1007-3639.2024.08.007  2. Shen Y, Zhang Y, Liu C, Gu S. Research Progress on Advanced Glycation End Products in Aging-related Diseases. Int J Geriatr. 2024;45(4).  3. Chen L, Li J, Yu H, Zou M, Liu J, Tian F, et al. Research Progress on Risk Factors for Sarcopenic Dysphagia in the Elderly. Practical Geriatrics. 2023;37(12):1255-9.  4. Cong X, Song J, Xu L, Zhao W. Research progress on current situation and the non-pharmaceutical intervention of oral frailty among older people in community. Journal of Nurses Training. 2025;40(3):280-4. doi: 10.16821/j.cnki.hsjx.2025.03.010  5. Cui H, Yang W, Wang C, Wang X, Cheng W, Cheng G. Research Progress of TCM in Prevention and Treatment of Alzheimer’s Disease by Regulating Different Signaling Pathways. ACTA CHINESE MEDICINE. 2024;39(8):1687-94. doi: 10.16368/j.issn.1674-8999.2024.08.277  6. Fu Z, Zhu Z, Zhao G. Research Progress in Diagnosis, Prevention and Treatment of Perioperative Neurocognitive Disorder in Elderly Frail Patients. Chinese Journal of Microcirculation. 2025;35(1):89-96. doi: 10.3969/j.issn.1005-1740.2025.01.015  7. Guo Q, Luo Y, Diao P, Luo X, Lan L. Research progress on pathogenesis of heart failure combined with cognitive fraity. Chinese Journal of Cardiovascular Research. 2024;22(11).  8. Huang Q, He X, Zhang H. Research progress on the relationship between sarcopenia and dysphagia. CHINESE EVIDENCE-BASED NURSING. 2025;11(02):244-8.  9. Huang S, Chen X. Research Progress on Schizophrenia Comorbid with Sarcopenia. Journal of International Psychiatry. 2025;52(01):9-12. doi: 10.13479/j.cnki.jip.2025.01.086  10. Jing S, Zhang S, Li P. Research Progress on the Mechanism and Exercise Intervention of Sarcopenia Combined with Neurological Related Diseases. Sports and Health. 2024;3(8):17-20.  11. Lei J, Zhang F, Wang J. Introduction - Research Progress on the Impact of Environmental Exposure on Common Geriatric Syndromes. Practical Geriatrics. 2025;39(1):1-2. doi: 10.3969/j.issn.1003-9198.2025.01.001  12. Lei S, Chen P, Chen X. Research Progress on the Potential Mechanisms of Exercise Regulating BDNF Expression to Improve Alzheimer’s Disease. Chinese Journal of Cell Biology. 2024;46(6):1249-62. doi: 10.11844/cjcb.2024.06.0019  13. Li D, Zhou M. Stroke-associated sarcopenia research progress. Medical Journal of Chinese People's Health. 2023;35(24):8-11.  14. Xian L, Li J, Yan H, Tang H, Li Y. Research Progress on the Correlation between Sarcopenia and Cognitive Impairment in the Elderly. Chinese Journal of Geriatric Heart Brain and Vessel Diseases. 2024;26(4).  15. Wang Y, Li Z, Li M, Zhang J, Qi X. Research Progress on the Pathogenesis of the Association between Sarcopenia and Cognitive Impairment in the Elderly. Practical Geriatrics. 2024;38(8).  16. Qiao X, Wang Y, Wang L, Han T, Yan Y. Research Progress on Respiratory Sarcopenia. Journal of Clinical Pulmonary Medicine. 2024;29(3).  17. Teng J, Liu X, Ye S, He J, Cai J, Fan D, et al. Research Progress on Brain and Spinal Cord Pathology in Amyotrophic Lateral Sclerosis Plus Syndrome. China Journal of Modern Medicine. 2024;26(11):1-4. doi: 10.3969/j.issn.1672-9463.2024.11.001  18. Wan Y, Shen X. Research Progress on the Assessment of Frailty in the Elderly Patients. Chinese Journal of Gerontology. 2024;44(17):4349-51.  19. Wang Y, Li Z, Li M, Zhang J, Qi X. Research Progress on the Mechanisms of Sarcopenia Associated with Cognitive Impairment in the Elderly. Practical Geriatrics. 2024;38(8).  20. Wang J, Chen P, He L, Sun Y. Research progress in STAT3 signal pathway in neurodegenerative diseases. Central South Pharmacy. 2024;22(1):6-16. doi: 10.7539/j.issn.1672-2981.2024.01.002  21. Wang L, Wu Le, Jia J. Pharmacological Effects of Roucongrong (Cistanches Herba) and Its Research Progress in Central Nervous System Diseases. CHINESE ARCHIVES OF TRADITIONAL CHINESE MEDICINE. 2024;42(3):50-4. doi: 10.13193/j.issn.1673-7717.2024.03.008  22. Wang S, Cui K, Li C, Fang G. Research Progress on the Improvement of Alzheimer’s Disease and Its Mechanism of Action through Aerobic Exercise Based on Insulin Resistance. CHINA SPORT SCIENCE AND TECHNOLOGY. 2024;60(7):21-34. doi: 10.16470/j.csst.2024072  23. Wen P, Zhang H, Zhao R. Research Progress on Sarcopenia in the Elderly. The Medical Forum. 2024;28(5).  24. Zhang K, Liu Q, Wu J. Research advances on the correlation between sarcopenia and cognitive impairment. Chinese Journal of Geriatrics. 2024;43(4).  25. Wu W, Xie X, Wang L, Su Y. Research Progress on Dynapenia in the Elderly. Shanghai Nursing. 2025;25(02):78-82.  26. Xian L, Li J, Yan H, Tang H, Li Y. Research Progress on the Correlation between Sarcopenia and Cognitive Impairment in the Elderly. Chinese Journal of Geriatric Heart Brain and Vessel Diseases. 2024;26(4).  27. Xie L, Chai Z, Gong Q, Wang X, Ru Y, Xiao B, et al. Research Progress on Pharmacological Effects and Mechanism of Acteoside on Central Nervous System Diseases. CHINESE ARCHIVES OF TRADITIONAL CHINESE MEDICINE. 2024;42(7):179-82. doi: 10.13193/j.issn.1673-7717.2024.07.036  28. Xie Y, Xu J, Xu F, Li C, Chen C, Shao C. Research progress in relationship between sarcopenia and prognosis of elderly stroke patients. Chinese Journal of Multiple Organ Diseases in the Elderly. 2024;23(1).  29. Zhang J, Chen C, Yang Y, Chen Y, Yang M. Research progress on correlation between sarcopenia and cognitive impairment and its mechanism. Laboratory Medicine and Clinic. 2023;20(24).  30. Zhang J, Quan Y, Xu B. Progress in evaluation and intervention of sarcopenia with frailty in patients with liver cirrhosis. Tianjin Journal of Nursing. 2024;32(1).  31. Zhang J, Chen C, Yang Y, Chen Y, Yang M. Research progress on correlation between sarcopenia and cognitive impairment and its mechanism. Laboratory Medicine and Clinic. 2023;20(24).  32. Zhang K, Liu Q, Wu J. Research advances on the correlation between sarcopenia and cognitive impairment. Chinese Journal of Geriatrics. 2024;43(4).  33. Zhao H, Sun S, Qin G, Ding Y, Zhao D. Mechanism of Traditional Chinese Medicine in Treatment of Neurodegenerative Diseases by  Regulating Polarization Balance of Microglia: A Review. Chinese Journal of Experimental Traditional Medical Formulae. 2024;30(2):244-53. doi: 10.13422/j.cnki.syfjx.20232294  34. Amini N, Ibn Hach M, Lapauw L, Dupont J, Vercauteren L, Verschueren S, et al. Meta-analysis on the interrelationship between sarcopenia and mild cognitive impairment, Alzheimer's disease and other forms of dementia. Journal of Cachexia, Sarcopenia and Muscle. 2024;15(4):1240-53. doi: 10.1002/jcsm.13485  35. Chan WLS, Pin TW, Chan JYH, Siu GCH, Tsang SMH. The Ability of Physical Performance Measures to Identify Fall Risk in Older Adults Living With Dementia: A Systematic Review and Meta-Analysis. Journal of the American Medical Directors Association. 2024;25(8). doi: 10.1016/j.jamda.2024.105100  36. De la Cruz-Gongora V, Palazuelos-Gonzalez R, Dominguez-Flores O. Micronutrient Deficiencies in Older Adults in Latin-America: A Narrative Review. Food and Nutrition Bulletin. 2024;45(2_SUPPL):S26-S38. doi: 10.1177/03795721231214587 PMID:WOS:001131833100001  37. de Smit MJ, Nijholt W, Bakker MH, Visser A. The predictive value of masticatory function for adverse health outcomes in older adults: a systematic review. Journal of Nutrition Health & Aging. 2024;28(5). doi: 10.1016/j.jnha.2024.100210 PMID:WOS:001292026800001  38. Dong M, Liu X, Choi Y, Li N. Effects of Otago Exercise Program and aquatic exercise on fall risk in older adults: A systematic review. Archives of Gerontology and Geriatrics. 2025;132. doi: 10.1016/j.archger.2025.105799  39. Ganggaya KS, Vanoh D, Ishak WRW. Prevalence of sarcopenia and depressive symptoms among older adults: a scoping review. Psychogeriatrics. 2024;24(2):473-95. doi: 10.1111/psyg.13060  40. Guan Z, Minnetti M, Heymsfield SB, Poggiogalle E, Prado CM, Sim M, et al. Beyond Traditional Body Composition Metrics: Load-Capacity Indices Emerge as Predictors of Cardiometabolic Outcomes-A Systematic Review and Meta-Analysis. Advances in Nutrition. 2025;16(2). doi: 10.1016/j.advnut.2024.100364 PMID:WOS:001421648600001  41. Huang C, Wu B, Zhang C, Wei Z, Su L, Zhang J, et al. Motoric Cognitive Risk Syndrome as a Predictor of Adverse Health Outcomes: A Systematic Review and Meta-Analysis. Gerontology. 2024;70(7):669-88. doi: 10.1159/000538314  42. Jayakody O, Blumen HM, Breslin M, Wang C, Verghese J. Risk factors associated with the Motoric Cognitive Risk syndrome: A meta-analysis of data from a cross-national study. Journal of the American Geriatrics Society. 2024;72(9):2656-66. doi: 10.1111/jgs.19032  43. Lanctot KL, Hahn-Pedersen JH, Eichinger CS, Freeman C, Clark A, Tarazona LRS, et al. Burden of Illness in People with Alzheimer's Disease: A Systematic Review of Epidemiology, Comorbidities and Mortality. Jpad-Journal of Prevention of Alzheimers Disease. 2024;11(1):97-107. doi: 10.14283/jpad.2023.61 PMID:WOS:000996935700001  44. Lim NEK, Yeo BSY, Lee RS, Lim JX, Chan YH, Kandiah N, et al. Motoric cognitive risk syndrome as a predictive factor of cognitive impairment and dementia – A systematic review and meta-analysis. Ageing Research Reviews. 2024;101. doi: 10.1016/j.arr.2024.102470  45. Lim SK, Choi K, Heo NH, Kim Y, Lim JY. Characteristics of fragility hip fracture-related falls in the older adults: A systematic review. Journal of Nutrition, Health and Aging. 2024;28(10). doi: 10.1016/j.jnha.2024.100357  46. Liu J, Zhu Y, Tan JK, Ismail AH, Ibrahim R, Hassan NH. Factors Associated with Frailty in Older Adults in Community and Nursing Home Settings: A Systematic Review with a Meta-Analysis. Journal of Clinical Medicine. 2024;13(8). doi: 10.3390/jcm13082382 PMID:WOS:001210660100001  47. Liu W, Qin R, Qiu Y, Luan T, Qiu B, Yan K, et al. Multidimensional frailty as a predictor of mortality among older adults: a systematic review and meta-analysis. Bmc Geriatrics. 2024;24(1). doi: 10.1186/s12877-024-05377-4 PMID:WOS:001325141800001  48. Luo Y, Wang Y, Tang S, Xu L, Zhao X, Han M, et al. Prevalence of sarcopenic obesity in the older non-hospitalized population: a systematic review and meta-analysis. Bmc Geriatrics. 2024;24(1). doi: 10.1186/s12877-024-04952-z PMID:WOS:001206332400004  49. Meng X, Wang Z, Lyu L. Bidirectional association between sarcopenia and depression: A systematic review and meta-analysis. Archives of Gerontology and Geriatrics. 2025;132. doi: 10.1016/j.archger.2025.105787 PMID:WOS:001434826300001  50. Nazareth CCG, Scalli ACAM, de Oliveira MPB, Gomes AFS, Brito-Costa S, Furtado GE, et al. Differences in lean mass and sarcopenia between individuals with Alzheimer's disease and those without dementia: A systematic review and meta-analysis of observational studies. Journal of Alzheimers Disease. 2025;103(1):92-107. doi: 10.1177/13872877241299051 PMID:WOS:001410734400017  51. Romeo M, Dallio M, Cipullo M, Coppola A, Mazzarella C, Mammone S, et al. Nutritional and Psychological Support as a Multidisciplinary Coordinated Approach in the Management of Chronic Liver Disease: A Scoping Review. Nutrition Reviews. 2025. doi: 10.1093/nutrit/nuaf001 PMID:WOS:001431518800001  52. Shao C, Zhao F, Chen J. A scoping review of operative limb dysfunction assessment and early functional exercise after cardiac implantable electronic device placement. Chinese Journal of Practical Nursing. 2024;40(25):1992-2001. doi: 10.3760/cma.j.cn211501-20240318-00615  53. Smit MJD, Nijholt W, Bakker MH, Visser A. The predictive value of masticatory function for adverse health outcomes in older adults: a systematic review. Journal of Nutrition, Health and Aging. 2024;28(5). doi: 10.1016/j.jnha.2024.100210  54. Ueshima J, Nagano F, Wakabayashi H, Maeda K, Arai H. Effectiveness of non-pharmacological therapies for preventing frailty in older people: An umbrella review. Archives of Gerontology and Geriatrics. 2025;128. doi: 10.1016/j.archger.2024.105628  55. Veronese N, Ragusa FS, Pegreffi F, Dominguez LJ, Barbagallo M, Zanetti M, et al. Sarcopenic obesity and health outcomes: An umbrella review of systematic reviews with meta-analysis. Journal of Cachexia, Sarcopenia and Muscle. 2024;15(4):1264-74. doi: 10.1002/jcsm.13502  56. Wang L, Su L, Shi L, Zhao D, Zhang C, Wu B. Measurement Practice of Slow Gait Speed for Motoric Cognitive Risk Syndrome: A Systematic Review. Journal of the American Medical Directors Association. 2025;26(1). doi: 10.1016/j.jamda.2024.105361  57. Yang Y-B, Zheng Y-B, Sun J, Yang L-L, Li J, Gong Y-M, et al. To nap or not? Evidence from a meta-analysis of cohort studies of habitual daytime napping and health outcomes. Sleep Medicine Reviews. 2024;78. doi: 10.1016/j.smrv.2024.101989 PMID:WOS:001421994000001  58. Zhong Y-J, Meng Q, Su C-H. Mechanism-Driven Strategies for Reducing Fall Risk in the Elderly: A Multidisciplinary Review of Exercise Interventions. Healthcare. 2024;12(23). doi: 10.3390/healthcare12232394 PMID:WOS:001376310500001  59. Chen L, Wei N, Wang X. Research Progress on the Impact of Sarcopenia on Adverse Health Outcomes in the Elderly. Journal of Modern Medicine & Health. 2021;37(16):2740-4.  60. Cui D, Qiu F, Qiu F, Li Z, Zhang Y, Li Y, et al. Physical Activity and Functional Rehabilitation for Aging People Based on ICF:A Scoping Review. Chinese Journal of Rehabilitation Theory and Practice. 2021;27(4).  61. Qin F. A Brief Review of Aspirin Adverse Reactions. Psychological Doctor. 2018;24(4):2.  62. Tang L, Zhou L, Zhang P. Research progress of sarcopenic obesity and chronic liver disease. Chinese Journal of Hepatology. 2023;31(4).  63. Yang W, Jiang Q, Li F, Wu F. Ｒesearch on common biomarkers of sarcopenia and cognitive impairment. Chinese Journal of Multiple Organ Diseases in the Elderly. 2022;21(06):460-3.  64. Yuan Y, Yang J. Effect of physical activity on health conditions and functioning for aging people with chronic diseases and functioning:a systematic review of systematic reviews. Chinese Journal of Rehabilitation Theory and Practice. 2022;28(9).  65. Zhang J, Zheng Y, Wang Q, Zhou S, Wang Q, Shen G, et al. Research progress on the correlations between sarcopenia and cognitive impairment. Chinese Journal of Clinical Healthcare. 2022;25(05):600-4.  66. Zhang L, Mo Y, Wang J. Research Progress on the Correlation Between Sarcopenia and Cognitive Impairment. Journal of Qilu Nursing. 2021;27(07):142-5.  67. Adamis D, Eikelenboom E. Supplementary Material for: The Role of Insulin-Like Growth Factor 1 in Delirium: A Systematic Review and Meta-Analysis. Figshare; 2023.  68. Adamis D, Eikelenboom P. The Role of Insulin-Like Growth Factor 1 in Delirium: A Systematic Review and Meta-Analysis. Dementia and Geriatric Cognitive Disorders. 2023;51(6):449-59. doi: 10.1159/000527061  69. Ahn S, Hui CS, 정혜정. Current Research Trends on Prevalence, Correlates with Cognitive Function, and Intervention on Sarcopenia in Community-dwelling Older Adults: Systematic Review. Journal of the Korea Gerontological Society. 2016;36(3):727-49. doi: PMID:KJD:ART002138150  70. Amini N, Ibn Hach M, Dupont J, Lapauw L, Vercauteren L, Verschueren S, et al. THE INTERRELATIONSHIP BETWEEN SARCOPENIA AND MILD COGNITIVE IMPAIRMENT, ALZHEIMER'S DISEASE AND DEMENTIA: A SYSTEMATIC REVIEW. Aging Clinical and Experimental Research. 2023;35:S365-S6. doi: 10.1007/s40520-023-02442-7  71. Banda KJ, Chu H, Chen R, Kang XL, Jen H-J, Liu D, et al. Prevalence of Oropharyngeal Dysphagia and Risk of Pneumonia, Malnutrition, and Mortality in Adults Aged 60 Years and Older: A Meta-Analysis. Gerontology. 2022;68(8):841-53. doi: 10.1159/000520326 PMID:WOS:000730948400001  72. Banda KJ, Chu H, Chen R, Kang XL, Jen HJ, Liu D, et al. Supplementary Material for: Prevalence of Oropharyngeal Dysphagia and Risk of Pneumonia, Malnutrition, and Mortality in Adults Aged 60 Years and Older: A Meta-Analysis. Figshare; 2021.  73. Bauman A, Merom D, Bull FC, Buchner DM, Fiatarone Singh MA. Updating the Evidence for Physical Activity: Summative Reviews of the Epidemiological Evidence, Prevalence, and Interventions to Promote "Active Aging". The Gerontologist. 2016;56:S268-S80. doi: 10.1093/geront/gnw031  74. Beck AM, Dent E, Baldwin C. Nutritional intervention as part of functional rehabilitation in older people with reduced functional ability: a systematic review and meta-analysis of randomised controlled studies. Journal of human nutrition and dietetics : the official journal of the British Dietetic Association. 2016;29(6):733-45. doi: 10.1111/jhn.12382  75. Bouaziz W, Vogel T, Schmitt E, Kaltenbach G, Geny B, Lang PO. Health benefits of aerobic training programs in adults aged 70 or over: A systematic review. Presse Medicale. 2017;46(9):794-807. doi: 10.1016/j.lpm.2017.05.028  76. Braun T, Thiel C, Peter RS, Bahns C, Büchele G, Rapp K, et al. Association of clinical outcome assessments of mobility capacity and incident disability in community-dwelling older adults - a systematic review and meta-analysis. Ageing Research Reviews. 2022;81. doi: 10.1016/j.arr.2022.101704  77. Buvat J, Maggi M, Guay A, Torres LO. Testosterone Deficiency in Men: Systematic Review and Standard Operating Procedures for Diagnosis and Treatment. Journal of Sexual Medicine. 2013;10(1):245-84. doi: 10.1111/j.1743-6109.2012.02783.x PMID:WOS:000313984900020  78. Cabett Cipolli G, Sanches Yassuda M, Aprahamian I. Sarcopenia Is Associated with Cognitive Impairment in Older Adults: A Systematic Review and Meta-Analysis. Journal of Nutrition, Health and Aging. 2019;23(6):525-31. doi: 10.1007/s12603-019-1188-8  79. Campi R, Berni A, Amparore D, Bertolo R, Capitanio U, Carbonara U, et al. Impact of frailty on perioperative and oncologic outcomes in patients undergoing surgery or ablation for renal cancer: a systematic review. Minerva Urology and Nephrology. 2022;74(22):146-60. doi: 10.23736/S2724-6051.21.04583-3  80. Capurso C, Bellanti F, Lo Buglio A, Vendemiale G. The Mediterranean Diet Slows Down the Progression of Aging and Helps to Prevent the Onset of Frailty: A Narrative Review. Nutrients. 2020;12(1). doi: 10.3390/nu12010035 PMID:WOS:000516825500035  81. Caristia S, Filigheddu N, Barone-Adesi F, Sarro A, Testa T, Magnani C, et al. Vitamin D as a biomarker of ill health among the over-50s: A systematic review of cohort studies. Nutrients. 2019;11(10). doi: 10.3390/nu11102384  82. Chang K-V, Hsu T-H, Wu W-T, Huang K-C, Han D-S. Association Between Sarcopenia and Cognitive Impairment: A Systematic Review and Meta-Analysis. Journal of the American Medical Directors Association. 2016;17(12). doi: 10.1016/j.jamda.2016.09.013 PMID:WOS:000389198200025  83. Chang K-V, Hsu T-H, Wu W-T, Huang K-C, Han D-S. Is sarcopenia associated with depression? A systematic review and meta-analysis of observational studies. Age and Ageing. 2017;46(5):738-46. doi: 10.1093/ageing/afx094 PMID:WOS:000408341000009  84. Chang KV, Hsu TH, Wu WT, Huang KC, Han DS. Association Between Sarcopenia and Cognitive Impairment: A Systematic Review and Meta-Analysis. Journal of the American Medical Directors Association. 2016;17(12):1164.e7-.e15. doi: 10.1016/j.jamda.2016.09.013  85. Charoenngam N, Rittiphairoj T, Jaroenlapnopparat A, Mettler SK, Ponvilawan B, Okoli U, et al. Mortality Risk After Atypical Femoral Fracture: A Systematic Review and Meta-analysis. Endocrine Practice. 2022;28(10):1072-7. doi: 10.1016/j.eprac.2022.08.005  86. Chen Z, Li W-Y, Ho M, Chau P-H. The Prevalence of Sarcopenia in Chinese Older Adults: Meta-Analysis and Meta-Regression. Nutrients. 2021;13(5). doi: 10.3390/nu13051441 PMID:WOS:000662423100001  87. Coelho-Júnior HJ, Trichopoulou A, Panza F. Cross-sectional and longitudinal associations between adherence to Mediterranean diet with physical performance and cognitive function in older adults: A systematic review and meta-analysis. Ageing Research Reviews. 2021;70. doi: 10.1016/j.arr.2021.101395  88. Crosby BJ, Lopez P, Galvao DA, Newton RU, Taaffe DR, Meniawy TM, et al. Associations of Physical Activity and Exercise with Health-related Outcomes in Patients with Melanoma During and After Treatment: A Systematic Review. Integrative Cancer Therapies. 2021;20. doi: 10.1177/15347354211040757 PMID:WOS:000693679100001  89. Cuesta-Triana F, Verdejo-Bravo C, Fernández-Pérez C, Martín-Sánchez FJ. Effect of Milk and Other Dairy Products on the Risk of Frailty, Sarcopenia, and Cognitive Performance Decline in the Elderly: A Systematic Review. Advances in nutrition (Bethesda, Md). 2019;10(2):S105-S19. doi: 10.1093/advances/nmy105  90. Cui M, Zhang S, Liu Y, Gang X, Wang G. Grip Strength and the Risk of Cognitive Decline and Dementia: A Systematic Review and Meta-Analysis of Longitudinal Cohort Studies. Front Aging Neurosci. 2021;13:625551. doi: 10.3389/fnagi.2021.625551 PMID:33613270  91. Da Silva JA, Martinez L, Rolland Y, Vellas B, Barreto PS. A NARRATIVE REVIEW OF MITOCHONDRIAL DYSFUNCTION AND INTRINSIC CAPACITY. Journal of Nutrition, Health and Aging. 2022;26(4):477-8. doi: 10.1007/s12603-022-1772-1  92. De Sire A, Moretti A, Giamattei MT, Gimigliano F, Iolascon G. Are dietary supplements and nutraceuticals effective for musculoskeletal health? a scoping review. Osteoporosis International. 2016;27(SUPPL 1):S103. doi: 10.1007/s00198-016-3530-x  93. Desai P, Mullerpatan R. Functioning of Older Adults in Low and Middle Income Countries: A Literature Review. Critical Reviews in Physical and Rehabilitation Medicine. 2022;34(1):69-82. doi: 10.1615/CritRevPhysRehabilMed.2022043231  94. Devnani P. Treatment of REM behaviour disorder - An evidence based review. Sleep Medicine. 2013;14:e20-e2. doi: 10.1016/j.sleep.2013.11.013  95. Fan Y, Shu X, Leung KCM, Lo ECM. Associations of general health conditions with masticatory performance and maximum bite force in older adults: A systematic review of cross-sectional studies. Journal of Dentistry. 2022;123. doi: 10.1016/j.jdent.2022.104186 PMID:WOS:000814634600003  96. Ferreira PH, Ferreira M, Maher C, Hopper J, Huxley R, Alcantara C, et al. What is the research involving twins and low back pain telling us? a systematic review. Physiotherapy (United Kingdom). 2011;97:eS340. doi: 10.1016/j.physio.2011.04.002  97. Fhon JRS, Silva ARF, Lima EFC, Santos Neto APD, Henao-Castaño Á M, Fajardo-Ramos E, et al. Association between Sarcopenia, Falls, and Cognitive Impairment in Older People: A Systematic Review with Meta-Analysis. Int J Environ Res Public Health. 2023;20(5). doi: 10.3390/ijerph20054156 PMID:36901167  98. Fritz NE, McCarthy CJ, Adamo DE. Handgrip strength as a means of monitoring progression of cognitive decline – A scoping review. Ageing Research Reviews. 2017;35:112-23. doi: 10.1016/j.arr.2017.01.004  99. Fuentes-Abolafio IJ, Stubbs B, Pérez-Belmonte LM, Bernal-López MR, Gómez-Huelgas R, Cuesta-Vargas A. Functional parameters indicative of mild cognitive impairment: a systematic review using instrumented kinematic assessment. BMC geriatrics. 2020;20(1):282. doi: 10.1186/s12877-020-01678-6  100. Fuentes-Abolafio IJ, Stubbs B, Pérez-Belmonte LM, Bernal-López MR, Gómez-Huelgas R, Cuesta-Vargas A. Functional objective parameters which may discriminate patients with mild cognitive impairment from cognitively healthy individuals: A systematic review and meta-analysis using an instrumented kinematic assessment. Age and Ageing. 2021;50(2):380-93. doi: 10.1093/ageing/afaa135  101. Gao Q, Hu K, Yan C, Zhao B, Mei F, Chen F, et al. Associated factors of sarcopenia in community-dwelling older adults: A systematic review and meta-analysis. Nutrients. 2021;13(12). doi: 10.3390/nu13124291  102. George PP, Lun P, Ong SP, Lim WS. A Rapid Review of the Measurement of Intrinsic Capacity in Older Adults. J Nutr Health Aging. 2021;25(6):774-82. doi: 10.1007/s12603-021-1622-6 PMID:34179933  103. Hanna L, Nguo K, Furness K, Porter J, Huggins CE. Association between skeletal muscle mass and quality of life in adults with cancer: a systematic review and meta-analysis. Journal of Cachexia Sarcopenia and Muscle. 2022;13(2):839-57. doi: 10.1002/jcsm.12928 PMID:WOS:000754458800001  104. Henwood TR, Keogh JW, Reid N, Jordan W, Senior HE. Assessing sarcopenic prevalence and risk factors in residential aged care: methodology and feasibility. Journal of Cachexia Sarcopenia and Muscle. 2014;5(3):229-36. doi: 10.1007/s13539-014-0144-z PMID:WOS:000342061700009  105. Herold F, Toerpel A, Schega L, Mueller NG. Functional and/or structural brain changes in response to resistance exercises and resistance training lead to cognitive improvements - a systematic review. European Review of Aging and Physical Activity. 2019;16. doi: 10.1186/s11556-019-0217-2 PMID:WOS:000475676700001  106. Huang SF, Chen C. Increasing physical activity prevents cognitive function decline: A review. Journal of Internal Medicine of Taiwan. 2020;31(2):122-30. doi: 10.6314/JIMT.202004_31(2).07  107. Inskip M, Mavros Y, Sachdev PS, Fiatarone Singh MA. Exercise for individuals with Lewy body Dementia: A systematic review. PLoS ONE. 2016;11(6). doi: 10.1371/journal.pone.0156520  108. Iolascon G, Gimigliano R, Bianco M, de Sire A, Moretti A, Giusti A, et al. Are dietary supplements and nutraceuticals effective for musculoskeletal health and cognitive function? A scoping review. Journal of Nutrition, Health and Aging. 2017;21(5):527-38. doi: 10.1007/s12603-016-0823-x  109. Jose Colho-Junior H, Trichopoulou A, Panza F. Cross-sectional and longitudinal associations between adherence to Mediterranean diet with physical performance and cognitive function in older adults: A systematic review and meta-analysis. Ageing Research Reviews. 2021;70. doi: 10.1016/j.arr.2021.101395 PMID:WOS:000684836300002  110. Kalyani HHN, Sullivan K, Moyle G, Brauer S, Jeffrey ER, Roeder L, et al. Effects of Dance on Gait, Cognition, and Dual-Tasking in Parkinson's Disease: A Systematic Review and Meta-Analysis. Journal of Parkinson's Disease. 2019;9(2):335-49. doi: 10.3233/JPD-181516  111. Kearney F, Harwood R, Gladman J, Lincoln N, Masud T. The relationship between executive function and falls and gait abnormalities in older adults: A systematic review. European Geriatric Medicine. 2012;3:S50-S1. doi: 10.1016/j.eurger.2012.07.060  112. Kearney F, Harwood RH, Gladman JR, Lincoln N, Masud T. Executive function and its relationship to falls and gait abnormalities in older adults: A systematic review. Age and Ageing. 2012;41:ii18. doi: 10.1093/ageing/afs112  113. Kearney FC, Harwood RH, Gladman JRF, Lincoln N, Masud T. The relationship between executive function and falls and gait abnormalities in older adults: A systematic review. Dementia and Geriatric Cognitive Disorders. 2013;36(1-2):20-35. doi: 10.1159/000350031  114. Kikkert LHJ, Vuillerme N, van Campen JP, Hortobágyi T, Lamoth CJ. Walking ability to predict future cognitive decline in old adults: A scoping review. Ageing Research Reviews. 2016;27:1-14. doi: 10.1016/j.arr.2016.02.001  115. Kim C, Placide S, Marcantonio ER, Afilalo J, Popma J, Kim D. Frailty assessment predicts long-term mortality after cardiac surgery: A systematic review. Journal of the American Geriatrics Society. 2015;63:S234. doi: 10.1111/jgs.13439  116. Kobayashi-Cuya KE, Sakurai R, Suzuki H, Ogawa S, Takebayashi T, Fujiwara Y. Observational Evidence of the Association Between Handgrip Strength, Hand Dexterity, and Cognitive Performance in Community-Dwelling Older Adults: A Systematic Review. J Epidemiol. 2018;28(9):373-81. doi: 10.2188/jea.JE20170041 PMID:29526916  117. Kueper JK, Speechley M, Lingum NR, Montero-Odasso M. Motor function and incident dementia: A systematic review and meta-analysis. Age and Ageing. 2017;46(5):729-38. doi: 10.1093/ageing/afx084  118. Lahoud T, Yu AY-D, King S. Masticatory dysfunction in older adults: A scoping review. Journal of Oral Rehabilitation. 2023;50(8):724-37. doi: 10.1111/joor.13493 PMID:WOS:000994041200001  119. Lahoud T, Yu AYD, King S. Masticatory dysfunction in older adults: A scoping review. Journal of oral rehabilitation. 2023;50(8):724-37. doi: 10.1111/joor.13493  120. Lanctot KL, Hahn-Pedersen JH, Eichinger CS, Freeman C, Clark A, Tarazona LRS, et al. Burden of Illness in People with Alzheimer's Disease: A Systematic Review of Epidemiology, Comorbidities and Mortality. Jpad-Journal of Prevention of Alzheimers Disease. 2023. doi: 10.14283/jpad.2023.61 PMID:WOS:000996935700001  121. Lee C, Ahn J, Lee BC. A Systematic Review of the Long-Term Effects of Using Smartphone- and Tablet-Based Rehabilitation Technology for Balance and Gait Training and Exercise Programs. Bioengineering (Basel). 2023;10(10). doi: 10.3390/bioengineering10101142 PMID:37892872  122. Li Z, Tong X, Ma Y, Bao T, Yue J. Prevalence of depression in patients with sarcopenia and correlation between the two diseases: systematic review and meta-analysis. Journal of Cachexia Sarcopenia and Muscle. 2022;13(1):128-44. doi: 10.1002/jcsm.12908 PMID:WOS:000740207800001  123. Liang Y, Shang S, Gao Y, Zhai J, Cheng X, Yang C, et al. Measurements of Intrinsic Capacity in Older Adults: A Scoping Review and Quality Assessment. Journal of the American Medical Directors Association. 2023;24(3):267-76.e2. doi: 10.1016/j.jamda.2022.09.011  124. Limpuangthip N, Komin O. Association between oral hypofunction and general health: a systematic review. BMC oral health. 2023;23(1):591. doi: 10.1186/s12903-023-03305-3  125. Lin RJ, Klepin HD. Evidence-Based Minireview: Longitudinal geriatric assessment in quality care for older patients with hematologic malignancies. Hematology Am Soc Hematol Educ Program. 2019;2019(1):59-62. doi: 10.1182/hematology.2019000076 PMID:31808857  126. Liu C, Wong PY, Chung YL, Chow SK-H, Cheung WH, Law SW, et al. Deciphering the "obesity paradox" in the elderly: A systematic review and meta-analysis of sarcopenic obesity. Obesity Reviews. 2023;24(2). doi: 10.1111/obr.13534 PMID:WOS:000891719000001  127. Liu J, Zhu Y, Tan JK, Ismail AH, Ibrahim R, Hassan NH. Factors Associated with Sarcopenia among Elderly Individuals Residing in Community and Nursing Home Settings: A Systematic Review with a Meta-Analysis. Nutrients. 2023;15(20). doi: 10.3390/nu15204335  128. Ma L. Meta-Analysis and functional components study of Ginkgo biloba tablet in the treatment of the blood stasis syndrome. Doctoral Degree, Air Force Medical University. 2020. Available from:https://d.wanfangdata.com.cn/thesis/ChJUaGVzaXNOZXdTMjAyMzA5MDESCUQwMjUyMzcxNRoIanZsY3RoNGg%3D  129. Masse FAA, Ansai JH, Fiogbe E, Rossi PG, Vilarinho ACG, Takahashi ACM, et al. Progression of Gait Changes in Older Adults With Mild Cognitive Impairment: A Systematic Review. Journal of geriatric physical therapy (2001). 2021;44(2):119-24. doi: 10.1519/JPT.0000000000000281  130. Mata Diz JB, Oliveira Leopoldino AA, Moreira BdS, Henschke N, Dias RC, Maximo Pereira LS, et al. Prevalence of sarcopenia in older Brazilians: A systematic review and meta-analysis. Geriatrics & Gerontology International. 2017;17(1):5-16. doi: 10.1111/ggi.12720 PMID:WOS:000394584100001  131. Mathewson SL, Azevedo PS, Gordon AL, Phillips BE, Greig CA. Overcoming protein-energy malnutrition in older adults in the residential care setting: A narrative review of causes and interventions. Ageing Research Reviews. 2021;70. doi: 10.1016/j.arr.2021.101401 PMID:WOS:000684836300005  132. Mullin DS, Cockburn A, Welstead M, Luciano M, Russ TC, Muniz-Terrera G. Mechanisms of motoric cognitive risk-Hypotheses based on a systematic review and meta-analysis of longitudinal cohort studies of older adults. Alzheimers Dement. 2022;18(12):2413-27. doi: 10.1002/alz.12547 PMID:35142038  133. Mullin DS, Cockburn A, Welstead M, Luciano M, Russ TC, Muniz-Terrera G. Mechanisms of motoric cognitive risk—Hypotheses based on a systematic review and meta-analysis of longitudinal cohort studies of older adults. Alzheimer's and Dementia. 2022;18(12):2413-27. doi: 10.1002/alz.12547  134. Nct. Biology, Identity & Opportunity Study. https://clinicaltrialsgov/show/NCT03412162. 2018. doi: PMID:CN-01522353  135. Nowson CA, Service C, Appleton J, Grieger JA. The impact of dietary factors on indices of chronic disease in older people: A systematic review. Journal of Nutrition, Health and Aging. 2018;22(2):282-96. doi: 10.1007/s12603-017-0920-5  136. O'Neill M, Duffy O, Henderson M, Kernohan WG. Identification of eating, drinking and swallowing difficulties for people living with early-stage dementia: A systematic review. International Journal of Language & Communication Disorders. 2023. doi: 10.1111/1460-6984.12924 PMID:WOS:001033707500001  137. Pacifico J, Geerlings MAJ, Reijnierse EM, Phassouliotis C, Lim WK, Maier AB. Prevalence of sarcopenia as a comorbid disease: A systematic review and meta-analysis. Experimental Gerontology. 2020;131. doi: 10.1016/j.exger.2019.110801 PMID:WOS:000508630900012  138. Pana A, Sourtzi P, Kalokairinou A, Pastroudis A, Chatzopoulos S-T, Velonaki VS. Association between muscle strength and sleep quality and duration among middle-aged and older adults: a systematic review. European Geriatric Medicine. 2021;12(1):27-44. doi: 10.1007/s41999-020-00399-8 PMID:WOS:000572617800001  139. Parmar MP, Kaur M, Kochhar G, Reddy KSV, Rajagopal ER, Batura U, et al. A SYSTEMIC REVIEW OF PREVALENCE OF DEPRESSION IN PATIENT TAKING LONG TERM DIALYSIS. European Journal of Molecular and Clinical Medicine. 2022;9(4):2376-90. doi:  140. Peng T-C, Chen W-L, Wu L-W, Chang Y-W, Kao T-W. Sarcopenia and cognitive impairment: A systematic review and meta-analysis. Clinical Nutrition. 2020;39(9):2695-701. doi: 10.1016/j.clnu.2019.12.014 PMID:WOS:000577928100006  141. Raeesi S, Hashemi R, Vahabi Z, Abdolahi M, Sedighiyan M. Is Percutaneous Endoscopic Gastrostomy Tube Feeding Beneficial for Improving Survival in Patients With Dementia? A Systematic Review and Meta-Analysis of Current Pieces of Evidence. Acta Medica Iranica. 2022;60(1):5-17. doi: 10.18502/acta.v60i1.8322  142. Ramírez F, Gutiérrez M. Dual-Task Gait as a Predictive Tool for Cognitive Impairment in Older Adults: A Systematic Review. Front Aging Neurosci. 2021;13:769462. doi: 10.3389/fnagi.2021.769462 PMID:35002676  143. Robertson DA, Savva GM, Kenny RA. Frailty and cognitive impairment-A review of the evidence and causal mechanisms. Ageing Research Reviews. 2013;12(4):840-51. doi: 10.1016/j.arr.2013.06.004 PMID:WOS:000328872400002  144. Sacha J, Sacha M, Sobon J, Borysiuk Z, Feusette P. Is It Time to Begin a Public Campaign Concerning Frailty and Pre-frailty? A Review Article. Frontiers in Physiology. 2017;8. doi: 10.3389/fphys.2017.00484 PMID:WOS:000405171300001  145. Sampogna G, Di Vincenzo M, Giallonardo V, Perris F, Volpicelli A, Del Vecchio V, et al. The Psychiatric Consequences of Long-COVID: A Scoping Review. Journal of Personalized Medicine. 2022;12(11). doi: 10.3390/jpm12111767  146. Sawicka AK, Renzi G, Olek RA. The bright and the dark sides of L-carnitine supplementation: a systematic review. Journal of the International Society of Sports Nutrition. 2020;17(1). doi: 10.1186/s12970-020-00377-2 PMID:WOS:000574271000001  147. Shaughnessy KA, Hackney KJ, Clark BC, Kraemer WJ, Terbizan DJ, Bailey RR, et al. A Narrative Review of Handgrip Strength and Cognitive Functioning: Bringing a New Characteristic to Muscle Memory. J Alzheimers Dis. 2020;73(4):1265-78. doi: 10.3233/jad-190856 PMID:31929158  148. Sousa-Fraguas MC, Rodriguez-Fuentes G, Conejo NM. Frailty and cognitive impairment in Parkinson's disease: a systematic review. Neurological Sciences. 2022;43(12):6693-706. doi: 10.1007/s10072-022-06347-7 PMID:WOS:000849147600002  149. Stanziano DC, Whitehurst M, Graham P, Roos BA. A review of selected longitudinal studies on aging: past findings and future directions. J Am Geriatr Soc. 2010;58 Suppl 2(Suppl 2):S292-7. doi: 10.1111/j.1532-5415.2010.02936.x PMID:21029056  150. Stubbs B, Eggermont L, Soundy A, Probst M, Vandenbulcke M, Vancampfort D. What are the factors associated with physical activity (PA) participation in community dwelling adults with dementia? A systematic review of PA correlates. Arch Gerontol Geriatr. 2014;59(2):195-203. doi: 10.1016/j.archger.2014.06.006 PMID:25034708  151. Takahashi S, Yonekura Y, Takanashi N, Tanno K. Risk Factors of Long-Term Care Insurance Certification in Japan: A Scoping Review. International Journal of Environmental Research and Public Health. 2022;19(4). doi: 10.3390/ijerph19042162  152. Tamura Y, Omura T, Toyoshima K, Araki A. Nutrition Management in Older Adults with Diabetes: A Review on the Importance of Shifting Prevention Strategies from Metabolic Syndrome to Frailty. Nutrients. 2020;12(11). doi: 10.3390/nu12113367 PMID:WOS:000594508600001  153. Tian Q, Resnick SM, Mielke MM, Yaffe K, Launer LJ, Jonsson PV, et al. Association of Dual Decline in Memory and Gait Speed With Risk for Dementia Among Adults Older Than 60 Years: A Multicohort Individual-Level Meta-analysis. JAMA Netw Open. 2020;3(2):e1921636. doi: 10.1001/jamanetworkopen.2019.21636 PMID:32083691  154. Ustevic C, Rajovic N, Stanisavljevic D, Tiosavljevic D, Pavlovic A, Tasic R, et al. From Sarcopenia to Depressive Symptoms in Elderly: A Path Analysis. Int J Environ Res Public Health. 2023;20(2). doi: 10.3390/ijerph20020972 PMID:36673727  155. van Dijk GM, Dekker J, Veenhof C, van den Ende CH. Course of functional status and pain in osteoarthritis of the hip or knee: a systematic review of the literature. Arthritis Rheum. 2006;55(5):779-85. doi: 10.1002/art.22244 PMID:17013827  156. Van Grootven B, van Achterberg T. Prediction models for functional status in community dwelling older adults: a systematic review. BMC Geriatr. 2022;22(1):465. doi: 10.1186/s12877-022-03156-7 PMID:35637447  157. Vicente BM, Lucio dos Santos Quaresma MV, Maria de Melo C, Lima Ribeiro SM. The dietary inflammatory index (DII®) and its association with cognition, frailty, and risk of disabilities in older adults: A systematic review. Clinical Nutrition ESPEN. 2020;40:7-16. doi: 10.1016/j.clnesp.2020.10.003  158. Volaklis K, Mamadjanov T, Meisinger C, Linseisen J. Association between muscular strength and depressive symptoms: Anarrative review. Wiener Klinische Wochenschrift. 2019;131(11-12):255-64. doi: 10.1007/s00508-019-1491-8 PMID:WOS:000471654600003  159. Waite SJ, Maitland S, Thomas A, Yarnall AJ. Sarcopenia and frailty in individuals with dementia: A systematic review. Archives of Gerontology and Geriatrics. 2021;92. doi: 10.1016/j.archger.2020.104268 PMID:WOS:000600899700029  160. Wan SN, Thiam CN, Ang QX, Engkasan J, Ong T. Incident sarcopenia in hospitalized older people: A systematic review. Plos One. 2023;18(8). doi: 10.1371/journal.pone.0289379 PMID:WOS:001043323500046  161. Wan SN, Thiam CN, Ang QX, Engkasan J, Ong T. Studies included in the systematic review. Figshare; 2023.  162. Wan SN, Thiam CN, Ang QX, Engkasan J, Ong T. PRISMA diagram for the systematic review. Figshare; 2023.  163. West EC, Williams LJ, Corney KB, Pasco JA. Is sarcopenia associated with anxiety symptoms and disorders? A systematic review and meta-analysis protocol. Bmj Open. 2021;11(11). doi: 10.1136/bmjopen-2021-054125 PMID:WOS:000717735500009  164. Xia L, Zhao R, Wan Q, Wu Y, Zhou Y, Wang Y, et al. Sarcopenia and adverse health-related outcomes: An umbrella review of meta-analyses of observational studies. Cancer Medicine. 2020;9(21):7964-78. doi: 10.1002/cam4.3428  165. Xu BY, Yan S, Low LL, Vasanwala FF, Low SG. Predictors of poor functional outcomes and mortality in patients with hip fracture: a systematic review. Bmc Musculoskeletal Disorders. 2019;20(1). doi: 10.1186/s12891-019-2950-0 PMID:WOS:000499939500001  166. Yang J, Jiang F, Yang M, Chen Z. Sarcopenia and nervous system disorders. Journal of Neurology. 2022;269(11):5787-97. doi: 10.1007/s00415-022-11268-8 PMID:WOS:000824263100002  167. Yang Y, Xiao M, Leng L, Jiang S, Feng L, Pan G, et al. A systematic review and meta-analysis of the prevalence and correlation of mild cognitive impairment in sarcopenia. Journal of Cachexia, Sarcopenia and Muscle. 2023;14(1):45-56. doi: 10.1002/jcsm.13143  168. Yu JT. Modifiable risk factors for incident dementia and cognitive impairment: An umbrella review of evidence. Journal of Prevention of Alzheimer's Disease. 2021;8(SUPPL 1):S147. doi: 10.14283/jpad.2021.58  169. Yuan S, Larsson SC. Epidemiology of sarcopenia: Prevalence, risk factors, and consequences. Metabolism: Clinical and Experimental. 2023;144. doi: 10.1016/j.metabol.2023.155533  170. Zhang X, Xiao H, Chen Y. Evaluation of a WeChat‐based life review programme for cancer patients: a quasi‐experimental study. Journal of advanced nursing (john wiley & sons, inc). 2019;75(7):1563‐74. doi: 10.1111/jan.14018 PMID:CN-02417307  171. Zhang YR, Xu W, Zhang W, Wang HF, Ou YN, Qu Y, et al. Modifiable risk factors for incident dementia and cognitive impairment: An umbrella review of evidence. Journal of Affective Disorders. 2022;314:160-7. doi: 10.1016/j.jad.2022.07.008  172. Xu J, Li L, Wu S, Yang J, Yang J, Yang W, et al. Correlation between sarcopenia and cognitive dysfunction: a systematic review. Chinese Journal of Evidence-Based Medicine. 2023;23(03):341-5.  173. Chen F. A systematic review of the relationship between sarcopenia and cognitive impairment Master's Degree, Lanzhou University. 2020. Available from:https://link-cnki-net-s.webvpn.cams.cn/doi/10.27204/d.cnki.glzhu.2020.003011  174. Chen X, Cao M, Liu M, Liu S, Zhao Z, Chen H. Association between sarcopenia and cognitive impairment in the older people: a meta-analysis. European Geriatric Medicine. 2022;13(4):771-87. doi: 10.1007/s41999-022-00661-1 |
| **Irrelevant articles** | 5465 | 1. Emerging Link between Sarcopenia Progression and Cognitive Impairment in Patients Undergoing Hemodialysis: A Prospective Cohort Study. 2023. doi: PMID:GRANTS:17530885  2. Upcoming Issue Preview. Journal of Practical Radiology. 2024;40(8):1319. doi: 10.3969/j.issn.1002-1671.2024.08.025  3. Huang Q, Tang J, Ling L, Zhu A, Li D, Lv R. The role of muscle-brain axis in the relationship between sarcopenia and cognitive impairment in the elderly. Chinese Journal of Geriatrics. 2024;43(11).  4. Mijit A, Cheng X, Wu H. Oral hypofunction in the elderly: A Review of the Literature. Chin J Ge iat Dent. 2024;22(4):240-5. doi: 10.19749/j.cn.cjgd.1672-2973.2024.04.011  5. An X, Wang J, Xu X, Gui S, Sui X, Qiu H, et al. Association of Sarcopenia with Executive Function and Episodic Memory. Heilongjiang Medical Journal. 2025;49(1):3-7. doi: 10.3969/j.issn.1004-5775.2025.01.001  6. Bi Y, Yang Y, Leshi Liu, Ma D, Zhao J, Zhong Q, et al. Study on the causal relationship between sarcopenia and cognitive impairment by  using two-sample Mendelian randomization. China Modern Doctor. 2024;62(36):60-5. doi: 10.3969/j.issn.1673-9701.2024.36.013  7. Cai X, Li M, Song X. Construction and Evaluation of a Sarcopenia Risk Prediction Model for Patients with Moderate to Severe Senile Dementia Treated with Donepezil. Physician Online. 2024;14(12):86-9. doi: 10.3969/j.issn.2095-7165.2024.12.025  8. Cai Y, Pu X, Li W. Study on the relationship between the severity of white matter hyperintensities and muscle mass loss associated with sarcopenia in the elderly. China Medical Herald. 2024;21(32):35-9+45. doi: 10.20047/j.issn1673-7210.2024.32.05  9. Shen M, Tao Q, Cao W, Zhai J, Ye L. Qualitative Study on the Current Status of Health Education for Sarcopenia in Community-Dwelling Elderly. Modern Nurse. 2024;31(4).  10. Chen L, Guo M, Li Z, Huang X. Mutual facilitation of Alzheimer disease and sarcopenia:roles of myokines,amyloid proteins,and other factors. Chinese Journal of Pathophysiology. 2024;40(1).  11. Chen W, Liu Z, Wang T, Du Y, Zhang G. A qualitative study of factors influencing motor behavior in patients with stroke and sarcopenia. Geriatrics Research. 2024;5(5).  12. Chen Y, Yuan W, Chen M, Xu D, Li Z, Bai H, et al. Malnutrition status and related influencing factors of elderly individuals with possible sarcopenia in a long-term care facility in Shanghai. Geriatrics & Health Care. 2024;30(3).  13. Chen Y, Du J, Xu G, Liang Y. Analysis of clinical characteristics of anti-leucine-rich glioma inactivated protein 1 antibody encephalitis in China. Clinical Research and Practice. 2024;9(18):86-9. doi: 10.19347/j.cnki.2096-1413.202418022  14. Chen H, Hu Q, Ye Q, Chen Z. The Application Value of Nutritional Rehabilitation Programs in Sarcopenia with Cognitive Impairment. ModernPractical Medicine. 2025;37(1).  15. Cui Y. Rheumatoid Arthritis Patients Should Guard Against Sarcopenia. Health Guide. 2024;30(4):50. doi: 10.3969/j.issn.1006-9038.2024.04.032  16. Deng D, Chen J, Qin C, Zhou Z, Lu W, Wang Y, et al. Association between cognitive function and the risk of sarcopenia in rural elderly people. Chinese Journal of Clinical Healthcare. 2024;27(6):734-9. doi: 10.3969/J.issn.1672-6790.2024.06.004  17. Deng P, Rong X, Wang H, Pan J, Huang R, Peng Y, et al. A case of type I sialidosis presenting with myoclonic seizures. Chinese Journal of Neurology. 2025;58(2):175-8. doi: 10.3760/cma.j.cn113694-20240810-00539  18. Fan H, Ren Z, Zhang H. Analysis of risk factors and prognosis of dialysis-related sarcopenia in elderly diabetic nephropathy patients. Geriatrics & Health Care. 2024;30(4).  19. Fang Y, Wan R, Huang J, Gao J, Liu Z. Exploring the Relationship between Intestinal Microbiota and Cognitive Impairment Associated with Sarcopenia in the Elderly Based on the Theory of "Spleen Housing Nutrients and Intentions". Journal of Basic Chinese Medicine. 2024:1-9. doi: 10.19945/j.cnki.issn.1006-3250.20240619.002  20. Fang G, Wang S, Du Q. The Role and Mechanism of Calcium Homeostasis in the Improvement of Alzheimer's Disease by Exercise. China Sport Science. 2024;44(9):71-80. doi: 10.16469/J.css.2024KX026  21. Feng Y. Anti-inflammatory diet and geriatric syndrome. Geriatrics & Health Care. 2024;30(3).  22. Yu H, Liu S, Liu C, Teng X, Zhang H, Fu X. Value of nutrition management based on PHES scale in patients with liver cirrhosis mild hepatic encephalopathy. Clinical Education of General Practice. 2024;22(12).  23. Gui H, Wu Z. Effects of propofol combined with dexmedetomidine used during laparoscopic myomectomy of patients on their postopera-tive pain and stress response. Chinese Journal of Family Planning. 2024;32(7):1524-7,32. doi: 10.3969/j.issn.1004-8189.2024.07.011  24. He Q, Chen B, Liang F, Kang Z, Zhou Y, Ji A, et al. Relationship between Alzheimer’s disease and sarcopenia and body mass index: analysis of GWAS datasets for European populations Chinese Journal of Tissue Engineering Research. 2025:1-11.  25. He Q, Xia Y, Lu B, Zhang Z. Relationship between sarcopenia and Alzheimer disease based on bioinformatics. Chinese Journal of Osteoporosis and Bone Mineral Research. 2024;17(5).  26. He Y, Kang P, Zhang L. Effects of serratus anterior muscle plane block combined with general anesthesia on cognitive function in elderly patients after thoracoscopic radical resection of lung adenocarcinoma. Geriatrics & Health Care. 2024;30(6):1564-8. doi: 10.3969/j.issn.1008-8296.2024.06.013  27. Chen L, Guo M, Li Z, Huang X. Mutual facilitation of Alzheimer disease and sarcopenia:roles of myokines,amyloid proteins,and other factors. Chinese Journal of Pathophysiology. 2024;40(1).  28. Jiang Y, Luo Y, Lin X, Wang Y, Gao Z, Lv H, et al. Effect of dementia on postoperative complications in older patients with hip fractures. Chinese Journal of Tissue Engineering Research. 2024;28(18):2895-900. doi: 10.12307/2024.027  29. Kang J, Wang M, Zhu J, Hu W, Zhu C, He M, et al. Analysis of risk factors for prolonged hospital stay and construction of prediction model based on data from nutritionDay worldwide 2020 to 2022 in China. Chinese Journal of Clinical Nutrition. 2025;33(1):1-15. doi: 10.3760/cma.j.cn115822-20241119-00208  30. Li D, Hu Y, Liu X, Yu G. Network pharmacology analysis and experimental validation of Anemarrhenae Rhizoma in treating Alzheimer's disease. Journal of Zhejiang University(Medical Sciences). 2024;53(1):84-97. doi: 10.3724/zdxbyxb-2023-0362  31. Li G, Ma S, Fang Z, Si B, Shu Q, Yan Z. Effect of TPVB combined with PCIA on hemodynamics in elderly patients undergoing thoracoscopic surgery under ultrasound localization. Journal of Harbin Medical University. 2024;58(1):64-9. doi: 10.20010/j.issn.1000-1905.2024.01.0064  32. Li H, Ren D, Yu L, Wen Y, Ding C, Miao Y, et al. Association between cognitive frailty and sarcopenia in hospitalized elderly population. Chinese Journal of Practical Internal Medicine. 2024;44(4).  33. Li R, Su J, Li J, Deng W, Zhang Y. A Mendelian Randomization Study on Alzheimer's Disease and Sarcopenia. Journal of Kunming Medical University. 2024;45(8).  34. Li Y, Qin H, Liu X, He Y, Chen F, Liu T. Report of a case of Heidenhain variant Creutzfeldt-Jakob disease and review of literature. Chinese Journal of Neurology. 2024;57(6):600-6. doi: 10.3760/cma.j.cn113694-20240104-00010  35. Liang L, Bi F, Song H, Wang J. The impact of lumboquadrate block combined with intravenous general anesthesia on anesthesia indices and postoperative safety in patients undergoing percutaneous nephrolithotomy for kidney stones. International Journal of Urology and Nephrology. 2025;45(1):44-8. doi: 10.3760/cma.j.cn431460-20240527-00011  36. Liu L, Zhang Y, Tian Y, Wu L, Meng L, Zhao T. Developments and reliability and validity tests of a sarcopenia cognitive behavior questionnaire for community elderly. Modern Preventive Medicine. 2024;51(6).  37. Liu M, Sun X, Wang J, Xing S, Nie P, Zhang T, et al. Current situation and influencing factors of daily living activities in the elderly living in nursing homes. Chinese Journal of Geriatric Care. 2024;22(5).  38. Liu Q, Yang Q, Duan Y, Han L, Xie Q, Cheng L. Heterogeneous trajectory of sarcopenia and associated factors among older adults with multimorbidity:an empirical study based on CHARLS survey data. Modern Preventive Medicine. 2024;51(12).  39. Liu W, Li R, Wang S, Zhu M. Application and influencing factors analysis of comprehensive elderly assessment in elderly hospitalized patients. Capital Food Medicine. 2024;31(12).  40. Xing Y, Ma L. Research advances on the correlation between mild cognitive impairment and sarcopenia. Chinese Journal of Geriatrics. 2024;43(5).  41. Ma L, Liu H, Xu R, Wang H. Prevalence and associated factors of sarcopenia in hospitalized older patients. Chinese Journal of Clinical Healthcare. 2024;27(3).  42. Nie Q. The application of grape seed extract in various animal disease models. Livestock and Poultry Industry. 2025;36(2):21-6. doi: 10.19567/j.cnki.1008-0414.2025.02.005  43. Peng P, Chen X, Zhou Y, Tian X, Tang Y, Deng D. Influencing factors for dysphagia in the elderly and establishment of a predictive model. Journal of Chongqing Medical University. 2025:1-10. doi: 10.13406/j.cnki.cyxb.003733  44. Qu S, Wang X, Wen W, Zhang W, Liu Y, Liu Y. Electro-clinical aspects of epilepsy with fixation-off sensitivity. Chinese Journal of Neurology. 2024;57(12):1309-16. doi: 10.3760/cma.j.cn113694-20240708-00467  45. Cai X, Li M, Song X. Construction and Evaluation of a Sarcopenia Risk Prediction Model for Patients with Moderate to Severe Senile Dementia Treated with Donepezil. Journal of Doctors Online. 2024;14(12).  46. Sun F, Du K, Yang L, Wang Z. The Impact of Dexmedetomidine Combined with Sufentanil on H-FABP, CK-MB, cTnI Levels and Cognitive Function in Elderly Patients Undergoing Heart Valve Replacement Surgery. Chinese Journal of Gerontology. 2023;43(23):5706-9. doi: 10.3969/j.issn.1005-9202.2023.23.021  47. Sun L, Liu A, Liu J. Study on the characteristics of juvenile and early adult dentatorubral-pallidoluysian atrophy. Chinese Journal of Neuroimmunology and Neurology. 2024;31(4):288-94. doi: 10.3969/j.issn.1006-2963.2024.04.007  48. Sun M, Zhou R, Zhang X, Cheng Y. Late-onset methylmalonic acidaemia CblC type:Two case reports and literature review. Journal of Jilin University(Medicine Edition). 2024;50(5):1420-5. doi: 10.13481/j.1671-587X.20240528  49. Sun Q, Wu Y, Cui L. Progress in the application of nutritional combined with resistance training in aged patients with sarcopenia. Chinese Journal of Geriatric Care. 2024;22(4).  50. Sun X, Xu J, Qin D, Xu M, Duan L. Study on the relationship between sarcopenia and multi-dimensional weakness in community elderly. Chinese Journal of Geriatric Care. 2024;22(4).  51. Tian C, Chu Z, Zhang T, Zhao B, Zhao X, Zhao C, et al. Clinical and molecular genetic analysis of 18 cases of cerebrotendinous xanthomatosis. Chinese Journal of Neurology. 2024;57(11):1217-26. doi: 10.3760/cma.j.cn113694-20240325-00192  52. Tian M, Long Q, Zeng C, Liu D, Wang P, Yuan L. Based on network pharmacology to explore mechanism of Dabuyuanjian against AD and verification of AMPK/SIRT1 signaling pathway. Chinese Journal of Immunology. 2024;40(8):1692-700. doi: 10.3969/j.issn.1000-484X.2024.08.020  53. Tong Q, Xiao Wang, Yu P, Yu J, Sheng Y, Zhao X, et al. Construction of a prediction model for the risk of sarcopenia in community and hospitalized elderly patients with chronic diseases. Chinese Journal of Geriatrics. 2024;43(11).  54. Wang C. The Impact of Dual-Task Intervention on Gait Posture in Sarcopenic Population. Journal of Medical Biomechanics. 2024;39(S01).  55. Wang H, Li G, Pang X, Wang J, Zhao R, Chang X, et al. Neuronal intranuclear inclusion disease presented with stroke-like onset: a case report. Chinese Journal of Neurology. 2023;56(12):1414-8. doi: 10.3760/cma.j.cn113694-20230824-00076  56. Wang J, Zhou Z, Zhou L, Jin L, Sun W, Wang Y. The value of gait markers other than gait speed in screening for sarcopenia with cognitive impairment. Chinese Journal of Geriatrics. 2024;43(10).  57. Wang L, Liu L. Clinical effect of Wuling powder in the treatment of hydrocephalus after stroke. Clinical Research and Practice. 2024;9(33):155-8. doi: 10.19347/j.cnki.2096-1413.202433039  58. Wang L, Ye L, Wang L, Le X, Zhan Y. Application value of cognitive-exercise dual-guided therapy in patients with maintenance hemodialysis obesity. Nursing Practice and Research. 2024;21(9).  59. Wang M, Wang D, Li X, Xu X, Zhao X, Zhang Z. Impact of preoperative sarcopenia on clinical outcomes after radical surgery in gastric cancer patients and its relationship with postoperative cognitive dysfunction. China Journal of General Surgery. 2024;33(10).  60. Wang W, Zhu D, Feng Y, Zhang Y. Study on The Correlation Between Serum Vitamin D Levels and Combined Sarcopenia in Elderly Patients with Type 2 Diabetes Mellitus. Food and Nutrition in China. 2024;30(12):84-8. doi: 10.3969/j.issn.1006-9577.2024.12.015  61. Wang W, Zhang M, Hu X, Liu Z, Gao C. The Effects of Probucol Combined with Atorvastatin Calcium on Cognitive Function, Blood Pressure Variability, and Target Organ Function in Elderly Patients with Hypertension and Lacunar Cerebral Infarction. Chinese Journal of Prevention and Control of Chronic Diseases. 2024;32(6):454-9. doi: 10.16386/j.cjpccd.issn.1004-6194.2024.06.011  62. Wang Y, Li X, Ji Y, Cui L, Cai Y. Differential effects of APOE polymorphism in neurotoxicity-responsive astrocytes induced by inflammatory factor. Journal of Jilin University(Medicine Edition). 2024;50(1):33-41. doi: 10.13481/j.1671-587X.20240105  63. Wang J, Zhou Z, Zhou L, Jin L, Sun W, Wang Y. The value of gait markers other than gait speed in screening for sarcopenia with cognitive impairment. Chinese Journal of Geriatrics. 2024;43(10).  64. Wei H, Yang Y. Effects of butorphanol combined with dexmedetomidine used during laparoscopic myomectomy of overweight patients on their immune function,postoperative cognition and inflammatory response. Chinese Journal of Family Planning. 2024;32(8):1813-8. doi: 10.3969/j.issn.1004-8189.2024.08.018  65. Wei L, Zhao J, Chen C, Zheng L. Sporadic Creutzfeldt-Jakob disease with hearing loss as the first symptom: one case report and literature review. Chinese Journal of Neuromedicine. 2025;24(1):65-7. doi: 10.3760/cma.j.cn115354-20240816-00489  66. Wei X, Qian F, Wu Y, Xu H, Wang C, Xu Y, et al. Kufor-Rakeb syndrome caused by ATP13A2 gene mutation: a case report and literature review. Chinese Journal of Neurology. 2024;57(5):467-72. doi: 10.3760/cma.j.cn113694-20230827-00091  67. Weng T, Zong M, Shen L, Wang Y, Qian C, Li Y, et al. Analysis of related factors of frailty in very elderly patients with multimorbidity. Chinese Journal of Geriatrics. 2024;43(7).  68. Wu X, Xu L, Chen Z, Zhu X, Zhao L. Development and Validation of a Risk Prediction Model for the Syndrome of Physical and Cognitive Decline in the Elderly. Journal of Nursing(China). 2024;31(10):73-8. doi: 10.16460/j.issn1008-9969.2024.10.073  69. Wu H, Lin J. Disease Burden and Harm of Osteoarthritis. Medical Journal of Peking Union Medical College Hospital. 2025;16(1):5-12. doi: 10.12290/xhyxzz.2024-0969  70. Wu H, Yu B. Management of geriatric syndromes from a multidisciplinary perspective: Challenges, practices, and development directions. Geriatrics & Health Care. 2024;30(3).  71. Wu J. Prevention and Management of Hypoglycemia in Elderly Patients with Diabetes. Today's Health. 2024;(2):10-1.  72. Wu Y, Zhang L. The Application of Nursing Intervention Based on the Interactive Achievement-Standard Mode in Patients After Percutaneous Coronary Intervention for Myocardial Infarction. Clinical Nursing Research. 2024;33(18):157-9. doi: 10.3969/j.issn.2097-1958.2024.18.053  73. Xiao H, Liu Z, Zhang Y. The Efficacy Analysis of Aripiprazole Combined with Risperidone in the Treatment of Patients with First-Episode Schizophrenia. The Journal of Medical Theory and Practice. 2024;37(2):218-21. doi: 10.19381/j.issn.1001-7585.2024.02.012  74. Xiao X, Song H, Zhou J. Analysis of risk factors for tumor-associated sarcopenia:a study based on the NHANES database. Chinese Journal of Practical Surgery. 2024;44(2).  75. Xie E, Tao H. Application trends of blood flow restriction training in clinical rehabilitation. Chinese Journal of Tissue Engineering Research. 2024;28(2):258-62. doi: 10.12307/2023.872  76. Xie H, Xia C, Xia Z, Zhang N, Shen J, Zhao H, et al. Correlation analysis of muscle mass and functional mobility in patients with cerebral small vessel disease. Chinese Journal of Cerebrovascular Diseases. 2024;21(8):514-24. doi: 10.3969/j.issn.1672-5921.2024.08.002  77. Xing Y, Ma L. Research advances on the correlation between mild cognitive impairment and sarcopenia. Chinese Journal of Geriatrics. 2024;43(5).  78. Yang Y, Yu Y, Xing F, Cai F, Xue X, Wang X, et al. Impact of intervention based on planned behavior theory on disease cognition,exercise self-efficacy,and daily living ability in elderly patients with sarcopenia. Modern Preventive Medicine. 2024;51(14).  79. Yang Y, Shi X, Ma Q. Impact of common geriatric syndromes on adverse prognosis in hemodialysis patients. Chinese Journal of Geriatrics. 2024;43(3).  80. Yi Z, Zou Z, Cheng X, Xu X, Zhou N, Luo H, et al. Influencing factors and interventional measures for cognitive frailty in patients on maintenance hemodialysis. Chinese Journal of Blood Purification. 2024;23(4).  81. You R, Cao Z, Tang X, Zhou L. Current status of clinical treatment and nutritional intervention in maintenance hemodialysis patients with sarcopenia. China Medicine. 2024;19(7).  82. Yu H, Liu S, Liu C, Teng X, Zhang H, Fu X. Value of nutrition management based on PHES scale in patients with liver cirrhosis mild hepatic encephalopathy. Clinical Education of General Practice. 2024;22(12).  83. Yuan W, Zheng C, Mei Z, Xu J, Xia W. Prevention and Treatment of Sarcopenia in the Elderly Based on the Concept of Preventive Treatment. Hunan Journal of Traditional Chinese Medicine. 2024;40(10).  84. Zhang H, Zhang K, Li X, Wang X, Yu C, Zhang K, et al. Construction and validation of a nomogramdiagnostic model for osteosarcopenia in maintenance hemodialysis patients. Chinese Journal of Modern Nursing. 2024;30(24):3242-9. doi: 10.3760/cma.j.cn115682-20240327-01627  85. Zhang J, Zhou J, Ji C, Wu D, Wang K. Progressive myoclonic epilepsy: a retrospective study of newly-diagnosed adult patients from a single center. Chinese Journal of Medical Genetics. 2024;41(4):432-6. doi: 10.3760/cma.j.cn511374-20230214-00073  86. Zhang M, Li S, Wang Q, Chai S, Lin Z. Neuroprotective mechanism of Esketamine modulating PI3K/AKT path-way on postoperative cognitive impairment induced by Isoflurane. China Modern Medicine. 2024;31(30):74-7. doi: 10.3969/j.issn.1674-4721.2024.30.018  87. Zhang P, Zhao Z, Cheng M, Lian Y, Tao Z. Clinical analysis of 12 cases of intracranial hypotension syndrome complicated with novel coronavirus pneumonia. Anhui Medical and Pharmaceutical Journal. 2024;28(12):2502-7. doi: 10.3969/j.issn.1009-6469.2024.12.034  88. Deng D, Chen J, Qin C, Zhou Z, Lu W, Wang Y, et al. Association between cognitive function and the risk of sarcopenia in rural elderly people. Chinese Journal of Clinical Healthcare. 2024;27(6).  89. Li R, Su J, Li J, Deng W, Zhang Y. A Mendelian Randomization Study on Alzheimer’s Disease and Sarcopenia. Journal of Kunming Medical University. 2024;45(8).  90. Zhang Y, Huang S, Wu R, Han Z, Shi L. Based on CiteSpace and VOSviewer Visual Analysis of Domestic Sarcopenia Research Hotspot and Trend. Advances in Clinical Medicine. 2024;14(2):3227-38. doi: 10.12677/acm.2024.142457  91. Wang M, Wang D, Li X, Xu X, Zhao X, Zhang Z. Impact of preoperative sarcopenia on clinical outcomes after radical surgery in gastric cancer patients and its relationship with postoperative cognitive dysfunction. China Journal of General Surgery. 2024;33(10).  92. He Q, Xia Y, Lu B, Zhang Z. Relationship between sarcopenia and Alzheimer disease based on bioinformatics. Chinese Journal Of Osteoporosis And Bone Mineral Research. 2024;17(5).  93. Zhao A, Wu J. Nutritional coping strategies for sleep disorders in the elderly. Geriatrics & Health Care. 2024;30(3).  94. Zhao B. Application of Cisatracurium Closed-Loop Target-Controlled Infusion Combined with Neural Block of the Uterosacral Ligament Region in Anesthesia for Elderly Patients with Uterine Fibroids. The Journal of Medical Theory and Practice. 2024;37(14):2427-9. doi: 10.19381/j.issn.1001-7585.2024.14.031  95. Zhao X, Wang D, Sun Q, Jin W, Wang W, Ren Y. Efficacy and safety of different stimulus dosage of modified electroconvulsive therapy in the treatment of depressive episode. Journal of Jinan University (Natural Science & Medicine Edition). 2024;45(6):589-95. doi: 10.11778/j.jdxb.20230138  96. Zhen X, Tian H, Xu J, Xu X, Zhang L, Yu Y. Robot-assisted deep brain stimulation for involuntary motor cerebral palsy: report of two cases and literature review. Chinese Journal of Brain Diseases and Rehabilitation (Electronic Edition). 2024;14(2):124-6. doi: 10.3877/cma.j.issn.2095-123X.2024.02.010  97. Zheng Y, Zhang Y, Zhang J, Wang Q, Lou X, Chen R, et al. Research on comorbidity of mild cognitive impairment and sarcopenia among old people in rural areas of Anhui Province. Journal of Shandong First Medical University & Shandong Academy of Medical Sciences. 2023;44(12).  98. Health TCCCBoCIEaPAfMa. Chinese expert consensus on the prevention and treatment of postoperative delirium of cardiovascular surgery. National Medical Journal of China. 2023;103(45):3635-44. doi: 10.3760/cma.j.cn112137-20230719-00028  99. Zhou R, Zhu M, Wang L. Effects of ropivacaine mixed with tramadol for quadratus lumborum block on postoperative cognition and rapid recovery in elderly patients with prostate cancer. Chinese Journal of Clinical Pharmacy. 2024;33(3):199-204. doi: 10.19577/j.1007-4406.2024.03.006  100. Bi Y, Yang Y, Liu L, Ma D, Zhao J, Zhong Q, et al. Study on the causal relationship between sarcopenia and cognitive impairment by using two-sample Mendelian randomization. China Modern Doctor. 2024;62(36).  101. Zhu J, Li C. A Case of Alzheimer's Disease with Intestinal Necrosis and Rhabdomyolysis. Chinese Journal of Digestion and Medical Imageology (Electronic Edition). 2024;14(4):382-4. doi: 10.3877/cma.j.issn.2095-2015.2024.04.019  102. Zhu M, Ying Q, Wang L, Zhou R. Effects of flurbiprofen combined with quadratus lumborum block on postoperative neurocognitive function and inflammatory response in elderly patients with nephrectomy under general anesthesia. Chinese Journal of New Drugs and Clinical Remedies. 2024;43(6):450-4. doi: 10.14109/j.cnki.xyylc.2024.06.11  103. Abbadi A, Kokoroskos E, Stamets M, Vetrano DL, Orsini N, Elmståhl S, et al. Validation of the Health Assessment Tool (HAT) based on four aging cohorts from the Swedish National study on Aging and Care. BMC Med. 2024;22(1):236. doi: 10.1186/s12916-024-03454-4 PMID:38858697  104. Abe T, Fujiwara Y, Kitamura A, Nofuji Y, Nishita Y, Makizako H, et al. Higher-level competence: Results from the Integrated Longitudinal Studies on Aging in Japan (ILSA-J) on the shape of associations with impaired physical and cognitive functions. Geriatrics and Gerontology International. 2024;24(4):352-8. doi: 10.1111/ggi.14839  105. Abudouaini H, Yang J, Li M, Zhang P, Lin K, Jiang Y, et al. Real-Time Ultrasound-Guided CT-Monitored Percutaneous Cervical Disc Injection: an Emerging Approach for Accurate Diagnosis of Cervical Discogenic Diseases. Journal of pain research. 2024;17:3975‐83. doi: 10.2147/JPR.S480008 PMID:CN-02787873  106. Acevedo-Fontanez AI, Rosano C, Yaffe K, Jeffrey Carr J, Terry JG, Nair S, et al. Abdominal Myosteatosis and Cognitive Function: Insights From CARDIA Study. Circulation. 2024;149. doi: 10.1161/circ.149.suppl_1.P338  107. Actrn. Effect of Accelerated Pacing Rates on Exercise Tolerance, Quality of Life and Arrhythmia Burden in Patients with Evidence of Heart Failure with Preserved Ejection Fraction. https://trialsearchwhoint/Trial2aspx?TrialID=ACTRN12623001187639. 2023. doi: PMID:CN-02629117  108. Actrn. A Hybrid Effectiveness-Implementation Randomised Control Trial of the 'Left Write Hook' program for Adult Survivors of Child Sexual Abuse. https://trialsearchwhoint/Trial2aspx?TrialID=ACTRN12624000862549. 2024. doi: PMID:CN-02728024  109. Actrn. An On-track Trial to Assess Driving from Alcohol. https://trialsearchwhoint/Trial2aspx?TrialID=ACTRN12624001163594. 2024. doi: PMID:CN-02759250  110. Adhiyaman V, Hobson P. Increasing number of deaths related to Parkinson's disease (PD) and Parkinsonism. Age and Ageing. 2024;53. doi: 10.1093/ageing/afad246.122 PMID:WOS:001154362300086  111. Ahmadi S, Afshar PF, Malakouti K, Azadbakht M. The relationship between intrinsic capacity and functional ability in older adults. BMC geriatrics. 2025;25(1):57. doi: 10.1186/s12877-025-05709-y  112. Ainsworth NJ, Oughli H, Lavretsky H, Blumberger DM, Brown PJ, Butters MA, et al. The Cognitive Profile of Older Adults With Treatment-Resistant Depression: an Analysis of the OPTIMUM Randomized Controlled Trial. American journal of geriatric psychiatry. 2024. doi: 10.1016/j.jagp.2024.09.018 PMID:CN-02770277  113. Akaida S, Taniguchi Y, Nakai Y, Kiuchi Y, Tateishi M, Shiratsuchi D, et al. Independent Association between Cognitive Frailty and Cardio-Ankle Vascular Index in Community-Dwelling Older Adults. Gerontology. 2024;70(5):499-506. doi: 10.1159/000536653  114. Akkan Suzan A, Ozen Barut B. The relationship between polypharmacy and physical performance in patients with early-stage Alzheimer’s disease. Current Medical Research and Opinion. 2024;40(2):253-8. doi: 10.1080/03007995.2023.2293109  115. Alarab A, Salhab I, Darawy D, Abu Aker M, Al Naji R, Hroub N. Maitland Mobilization versus Core Stability Exercises in Management Chronic Nonspecific LBP. Biomedical and pharmacology journal. 2024;17(2):1115‐24. doi: 10.13005/bpj/2927 PMID:CN-02727395  116. Alberton CL, Andrade LS, Xavier BEB, Pinheiro VHG, Cuesta-Vargas AI, Pinto SS. Land- and water-based aerobic exercise program on health-related outcomes in breast cancer survivors (WaterMama): study protocol for a randomized clinical trial. Trials. 2024;25(1):536. doi: 10.1186/s13063-024-08389-y PMID:CN-02739532  117. Albrecht F, Johansson H, Ekman U, Poulakis K, Bezuidenhout L, Pereira JB, et al. Investigating underlying brain structures and influence of mild and subjective cognitive impairment on dual-task performance in people with Parkinson's disease. Scientific reports. 2024;14(1):9513. doi: 10.1038/s41598-024-60050-5  118. Ali P, Pieruccini-Faria F, Annweiler C, Dinomais M, Son S, Wilson SK, et al. Smaller cingulate grey matter mediates the association between dual-task gait and incident dementia. Brain : a journal of neurology. 2024. doi: 10.1093/brain/awae356  119. Ali P, Renaud P, Montero-Odasso M, Gautier J, Dinomais M, Annweiler C. Gait performance in older adults across the cognitive spectrum: Results from the GAIT cohort. J Am Geriatr Soc. 2024;72(11):3437-47. doi: 10.1111/jgs.19162 PMID:39206968  120. Almeida JKA, Brech GC, Luna NMS, Iborra RT, Soares-Junior JM, Baracat EC, et al. Advanced glycation end products consumption and the decline of functional capacity in patients with Parkinson's disease: Cross-sectional study. Clinics (Sao Paulo). 2024;79:100320. doi: 10.1016/j.clinsp.2023.100320 PMID:38301537  121. Almevall A, Almevall AD, Öhlin J, Gustafson Y, Zingmark K, Niklasson J, et al. Self-rated health in old age, related factors and survival: A 20-Year longitudinal study within the Silver-MONICA cohort. Archives of Gerontology and Geriatrics. 2024;122. doi: 10.1016/j.archger.2024.105392  122. Almutairi GR, Almegbas NR, Alosaimi RM, Alqahtani MA, Batook SG, Alfageh IA, et al. Comorbidities, medications, depression, and physical performance measures associated with severe cognitive impairments in community-dwelling adults. PLoS ONE. 2024;19(9 September). doi: 10.1371/journal.pone.0309765  123. Aly M, Sakamoto M, Kamijo K. Grip strength, working memory, and emotion perception in middle-aged males. Prog Brain Res. 2024;286:89-105. doi: 10.1016/bs.pbr.2023.12.004 PMID:38876580  124. An R, Huang X, Zhang S, Gao Y, Li L, Wan Q. Can motor decline be a modifiable marker of clinical progression in subjective cognitive decline? A national prospective cohort study. Asian Journal of Psychiatry. 2024;94. doi: 10.1016/j.ajp.2024.103978  125. An TJ, Lim J, Lee H, Ji S, Jung H-W, Baek JY, et al. Breathlessness, Frailty, and Sarcopenia in Older Adults. Chest. 2024;166(6):1476-86. doi: 10.1016/j.chest.2024.07.180 PMID:WOS:001408628100001  126. Anandavadivelan P, Cardinale D, Blomhoff R, Sunde B, Lassen K, Kleive D, et al. Blood flow restriction Exercise in the perioperative setting to Prevent loss of muscle mass in patients with pancreatic, biliary tract, and liver cancer: study protocol for the PREV-Ex randomized controlled trial. Trials. 2024;25(1):356. doi: 10.1186/s13063-024-08207-5 PMID:CN-02708254  127. Anandavadivelan P, Mijwel S, Wiklander M, Kjoe PLM, Luijendijk M, Bergh J, et al. Five-year follow-up of the OptiTrain trial on concurrent resistance and high-intensity interval training during chemotherapy for patients with breast cancer. Scientific reports. 2024;14(1):15333. doi: 10.1038/s41598-024-65436-z PMID:CN-02724151  128. Antonellis P, Weightman MM, Fino PC, Chen S, Lester ME, Hoppes CW, et al. Relation Between Cognitive Assessment and Clinical Physical Performance Measures After Mild Traumatic Brain Injury. Archives of Physical Medicine and Rehabilitation. 2024;105(5):868-75. doi: 10.1016/j.apmr.2023.10.013  129. Arai H, Okada S, Fukuoka T, Nozoe M, Kamiya K, Matsumoto S, et al. Association of Baseline Skeletal Muscle Mass Index With Adverse Events and Rehabilitation Outcomes in Patients Admitted for Rehabilitation. Archives of Rehabilitation Research and Clinical Translation. 2024;6(1). doi: 10.1016/j.arrct.2023.100314  130. Ashaat EA, Ahmed HA, Elaraby NM, Fayez A, Metwally AM, Mekkawy MK, et al. The Diagnostic Value of Whole-Exome Sequencing in a Spectrum of Rare Neurological Disorders Associated with Cerebellar Atrophy. Molecular Neurobiology. 2024;61(8):4949-61. doi: 10.1007/s12035-023-03866-y  131. Asiello JD, Kelley CM, Cannone K, McInnis L, Begin A, Dittrich M, et al. Addressing the functional needs of left ventricular assist device candidates: Development and feasibility of an occupational therapy pre-operative evaluation. Heart Lung. 2024;64:198-207. doi: 10.1016/j.hrtlng.2024.01.006 PMID:38301417  132. Atciyurt K, Heybeli C, Smith L, Veronese N, Soysal P. The prevalence, risk factors and clinical implications of dehydration in older patients: a cross-sectional study. Acta Clinica Belgica: International Journal of Clinical and Laboratory Medicine. 2024;79(1):12-8. doi: 10.1080/17843286.2023.2275922  133. Aydin AE, Dost FS, Kaya D, Ates Bulut E, Mutlay F, Isik AT. Sarcopenia in older patients with idiopathic normal pressure hydrocephalus: an observational study from a single geriatric clinic in Turkey. Acta Neurologica Belgica. 2024;124(5):1623-9. doi: 10.1007/s13760-024-02583-0  134. Aznar-Gimeno R, Perez-Lasierra JL, Perez-Lazaro P, Bosque-Lopez I, Azpiroz-Puente M, Salvo-Ibanez P, et al. Gait-Based AI Models for Detecting Sarcopenia and Cognitive Decline Using Sensor Fusion. Diagnostics. 2024;14(24). doi: 10.3390/diagnostics14242886 PMID:WOS:001384922100001  135. Backman WD, DiCaro MV, Zuo X, Peralta A, Orkaby AR. Aligning goals with care: Advance directives in older adults with implantable cardioverter-defibrillators. PACE - Pacing and Clinical Electrophysiology. 2024;47(5):697-701. doi: 10.1111/pace.14983  136. Bai W, Ma R, Yang Y, Xu J, Qin L. Enhancing predictive validity of motoric cognitive risk syndrome for incident dementia and all-cause mortality with handgrip strength: insights from a prospective cohort study. Frontiers in Aging Neuroscience. 2024;16. doi: 10.3389/fnagi.2024.1421656  137. Balasubramanian I, Malhotra C. Can Timely Outpatient Visits Reduce Readmissions and Mortality Among Heart Failure Patients? Journal of General Internal Medicine. 2024;39(13):2478-86. doi: 10.1007/s11606-024-08755-1  138. Balietti M, Galeazzi R, Giacconi R, Santillo E, Giuli C. Early Benefits with Potential Long-Term Risks of a Comprehensive Intervention on Serum Cortisol Levels and Cognitive Performance in Patients with Alzheimer's Disease. Journal of Alzheimer's disease reports. 2023;7(1):1445‐53. doi: 10.3233/ADR-230125 PMID:CN-02662845  139. Bao R, Leahy AA, Lubans DR, Diallo TMO, Beauchamp MR, Smith JJ, et al. Mediators of the association between physical activity and executive functions in primary school children. Journal of sports sciences. 2024;42(21):2029‐38. doi: 10.1080/02640414.2024.2422203 PMID:CN-02774143  140. Barichella M, Cereda E, Ferri V, Bolliri C, Cereda V, Colombo A, et al. Sarcopenia, low muscle strength, cognitive functions, and quality of life in parkinsonian syndromes. Nutrition. 2024;128. doi: 10.1016/j.nut.2024.112568  141. Barone R, Bramato G, Gnoni V, Giugno A, Urso D, Zecca C, et al. Sarcopenia in subjects with Alzheimer's disease: prevalence and comparison of agreement between EGWSOP1, EGWSOP2, and FNIH criteria. BMC geriatrics. 2024;24(1):278. doi: 10.1186/s12877-024-04890-w  142. Barros RdS, Teixeira GTM, Pinto JM, Sampaio RX, Mendes FAdS, Garcia PA. FACTORS PREDICTING HOSPITAL ADMISSION AND DEATH IN OLDER ADULTS WITH COGNITIVE IMPAIRMENT: A LONGITUDINAL STUDY. Texto & Contexto - Enfermagem. 2024;33:e20230149-e. doi: 10.1590/1980-265x-tce-2023-0149en PMID:SCIELO:S0104-07072024000100310  143. Beauchamp M, Kirkwood R, Duong M, Ho T, Raina P, Kruisselbrink R, et al. Long-Term Functional Limitations and Predictors of Recovery After COVID-19: A Multicenter Prospective Cohort Study. American Journal of Medicine. 2024;137(10):990-1000. doi: 10.1016/j.amjmed.2024.06.005  144. Bektan Kanat B, Suzan V, Ulugerger Avci G, Unal D, Emiroglu Gedik T, Suna Erdincler D, et al. Systemic inflammatory response index and monocyte-to-high density lipoprotein ratio- new biomarkers remarking the inflammation in primary sarcopenia: The SIMPS study. Bratisl Lek Listy. 2024;125(5):331-6. doi: 10.4149/bll_2024_49 PMID:38624059  145. Belkin AA, Belkin VA, Vasilchenko IE, Pinchuk EA. Results of a cohort single-center randomized study of the modulating effect of the drug Mexidol in the rehabilitation of patients who suffered acute cerebral insufficiency. Zhurnal nevrologii I psikhiatrii imeni SS Korsakova. 2024;124(4):108‐17. doi: 10.17116/jnevro2024124041108 PMID:CN-02692432  146. Ben Ayed I, Ammar A, Boujelbane MA, Salem A, Naija S, Amor SB, et al. Acute Effect of Simultaneous Exercise and Cognitive Tasks on Cognitive Functions in Elderly Individuals with Mild Cognitive Impairment. Diseases. 2024;12(7). doi: 10.3390/diseases12070148 PMID:CN-02736923  147. Bhatt VR, Wichman C, Koll TT, Fisher AL, Wildes TM, Berger A, et al. Longitudinal changes in cognitive and physical function and health-related quality of life in older adults with acute myeloid leukemia. Journal of Geriatric Oncology. 2024;15(1). doi: 10.1016/j.jgo.2023.101676  148. Bhattarai U, Gautam A, Shrestha M, Rayamajhi A, Basnet R, Saravanan M, et al. Factors Associated with Subjective Aging Among Older Outpatients In Northern - India. J Frailty Sarcopenia Falls. 2024;9(2):122-30. doi: 10.22540/jfsf-09-122 PMID:38835619  149. Biase JKADA, Brech GC, Luna NMS, Iborra RT, Soares-Junior JM, Baracat EC, et al. Advanced glycation end products consumption and the decline of functional capacity in patients with Parkinson's disease: Cross-sectional study. Clinics. 2024;79. doi: 10.1016/j.clinsp.2023.100320  150. Blanco-Rambo E, Bandeira-Guimarães M, Rambo ES, Formighieri C, Steffens T, Cadore EL, et al. Effects of social distancing provoked by COVID-19 pandemic in the functional capacity and cognitive function in nonagenarians and centenarians. Journal of Bodywork and Movement Therapies. 2024;37:46-50. doi: 10.1016/j.jbmt.2023.11.013  151. Blumen HM, Jayakody O, Ayers E, Barzilai N, Habeck C, Milman S, et al. Cognitive reserve proxies are associated with age-related cognitive decline - Not age-related gait speed decline. Neurobiol Aging. 2024;141:46-54. doi: 10.1016/j.neurobiolaging.2024.05.012 PMID:38820770  152. Bollinger RM, Chen SW, Krauss MJ, Keleman AA, Kehrer-Dunlap A, Kaesler M, et al. The Association Between Postural Sway and Preclinical Alzheimer Disease Among Community-Dwelling Older Adults. J Gerontol A Biol Sci Med Sci. 2024;79(7). doi: 10.1093/gerona/glae091 PMID:38554257  153. Booranasuksakul U, Macdonald IA, Stephan BCM, Siervo M. Body Composition, Sarcopenic Obesity, and Cognitive Function in Older Adults: Findings From the National Health and Nutrition Examination Survey (NHANES) 1999-2002 and 2011-2014. Journal of the American Nutrition Association. 2024;43(6):539-52. doi: 10.1080/27697061.2024.2333310 PMID:WOS:001195290000001  154. Booranasuksakul U, Tsintzas K, Macdonald I, Stephan BCM, Siervo M. Application of a New Definition of Sarcopenic Obesity in Middle-Aged and Older Adults and Association with Cognitive Function: Findings from the National Health and Nutrition Examination Survey 1999-2002. Proceedings of the Nutrition Society. 2024;83(OCE4). doi: 10.1017/S0029665124006931  155. Borda MG, Duque G, Pérez-Zepeda MU, Baldera JP, Westman E, Zettergren A, et al. Using magnetic resonance imaging to measure head muscles: An innovative method to opportunistically determine muscle mass and detect sarcopenia. Journal of Cachexia, Sarcopenia and Muscle. 2024;15(1):189-97. doi: 10.1002/jcsm.13362  156. Borda MG, Patricio Baldera J, Patino-Hernandez D, Westman E, Pérez-Zepeda MU, Tarazona-Santabalbina FJ, et al. Temporal Muscle Thickness Predicts Mortality and Disability in Older Adults Diagnosed with Mild Dementia. J Frailty Aging. 2024;13(4):441-7. doi: 10.14283/jfa.2024.39 PMID:39574265  157. Bott R, Zylstra J, Knight W, Whyte GP, Lane AM, Moss C, et al. Prehabilitation of Patients With Oesophageal Malignancy Undergoing Peri-Operative Treatment (Pre-EMPT): Outcomes From a Prospective Controlled Trial. Journal of Surgical Oncology. 2025. doi: 10.1002/jso.28079 PMID:WOS:001408133600001  158. Boucaud-Maitre D, Simo N, Villeneuve R, Rambhojan C, Thibault N, Joseph SP, et al. Clinical profiles of older adults in French Caribbean nursing homes: a descriptive cross-sectional study. Frontiers in Medicine. 2024;11. doi: 10.3389/fmed.2024.1428443  159. Boucaud-Maitre D, Simo-Tabue N, Mounsamy L, Rambhojan C, Letchimy L, Rinaldo L, et al. Malnutrition and its determinants among older adults living in foster families in Guadeloupe (French West Indies). A crosssectional study. PLoS ONE. 2024;19(6 June). doi: 10.1371/journal.pone.0304998  160. Bower WF, D'Souza AN, Barson E, Marston C, Granger CL, Beach L, et al. Previously healthy unvaccinated adults have significant functional limitations in the medium and long term after mild COVID-19. Aust J Gen Pract. 2024;53(7):491-7. doi: 10.31128/ajgp-07-23-6900 PMID:38957067  161. Britting S, Kob R, Goerlitz A, Sieber CC, Freiberger E, Rohleder N. Chronic stress and functional health in older adults with concerns about falling: a study protocol of a randomized controlled trial with multicomponent exercise intervention (FEARFALL). Trials. 2024;25(1). doi: 10.1186/s13063-024-08462-6 PMID:WOS:001316534900001  162. Brobakken MF, Nygård M, Vedul-Kjelsås E, Harvey PD, Wang E. Everyday function in schizophrenia: The impact of aerobic endurance and skeletal muscle strength. Schizophrenia Research. 2024;270:144-51. doi: 10.1016/j.schres.2024.06.027  163. Busquets A, Ferrer-Uris B, Durduran T, Bešlija F, Añón-Hidalgo M, Angulo-Barroso R. Study protocol to examine the effects of acute exercise on motor learning and brain activity in children with developmental coordination disorder (ExLe-Brain-DCD). PloS one. 2024;19(5):e0302242. doi: 10.1371/journal.pone.0302242 PMID:CN-02698918  164. Butera KA, Gustavson AM, Forster JE, Malone D, Stevens-Lapsley JE. Admission Cognition and Function Predict Change in Physical Function Following Skilled Nursing Rehabilitation. Journal of the American Medical Directors Association. 2024;25(1):17-23. doi: 10.1016/j.jamda.2023.09.011  165. Camacho-Ruíz J, V A, Rendón-Macías ME, Bernabe-García M, González-Bautista E, Manuel-Apolinar L, et al. [Cognitive function groups in older people and risk factors]. Rev Med Inst Mex Seguro Soc. 2024;62(3):1-11. doi: 10.5281/zenodo.10998801 PMID:39528344  166. Cao YF, Shi GP, Zhang H, Sun MZ, Wang ZD, Chu XF, et al. Association between Perceived Stress and Motoric Cognitive Risk Syndrome in an Elderly Population: Rugao Longevity and Aging Study. Dementia and Geriatric Cognitive Disorders. 2024;53(2):74-82. doi: 10.1159/000537937  167. Casals C, Ávila-Cabeza-de-Vaca L, González-Mariscal A, Marín-Galindo A, Costilla M, Ponce-Gonzalez JG, et al. Effects of an educational intervention on frailty status, physical function, physical activity, sleep patterns, and nutritional status of older adults with frailty or pre-frailty: the FRAGSALUD study. Frontiers in public health. 2023;11:1267666. doi: 10.3389/fpubh.2023.1267666 PMID:CN-02634592  168. Cascais I, Garrido C, Morais L, Amorim R, Lima R, Mansilha HF, et al. Myotonic dystrophy type 1 (Steinert disease): 29 years of experience at a tertiary pediatric hospital. European Journal of Paediatric Neurology. 2024;48:85-90. doi: 10.1016/j.ejpn.2023.12.001  169. Casemiro FG, de Carvalho LPN, Matiello FdB, Resende MC, Rodrigues RAP. Influence of frailty and cognitive decline on dual task performance in older adults: an analytical cross-sectional study. Revista Latino-Americana De Enfermagem. 2025;33. doi: 10.1590/1518-8345.7159.4485 PMID:WOS:001428480400001  170. Castano LAA, Vilas-Boas VF, De Lima VC, Coelho-Junior HJ, Uchida MC. Sarcopenia-Related Parameters in Older Adults With End-Stage Renal Disease: A Case-Control Study. Journal of Sport Rehabilitation. 2025. doi: 10.1123/japa.2024-0298 PMID:WOS:001429339700001  171. Castellote-Caballero Y, Carcelén Fraile MDC, Aibar-Almazán A, Afanador-Restrepo DF, González-Martín AM. Effect of combined physical-cognitive training on the functional and cognitive capacity of older people with mild cognitive impairment: a randomized controlled trial. BMC medicine. 2024;22(1):281. doi: 10.1186/s12916-024-03469-x PMID:CN-02724894  172. Catikkas NM, Tunc M, Soysal P. The prevalence of excessive daytime sleepiness and associated factors in older diabetic patients. Aging Clinical and Experimental Research. 2023;35(12):3263-. doi: 10.1007/s40520-023-02602-9 PMID:WOS:001117912400002  173. Cezon-Serrano N, Arnal-Gomez A, Arjona-Tinaut L, Cebria i Iranzo MA. Functional and emotional impact of COVID-19 lockdown on older adults with sarcopenia living in a nursing home: A 15-month follow-up. Nursing & Health Sciences. 2023;25(4):597-608. doi: 10.1111/nhs.13050 PMID:WOS:001066524800001  174. Chang JR, Yao ZF, Hsieh S, Nordling TEM. Age Prediction Using Resting-State Functional MRI. Neuroinformatics. 2024;22(2):119-34. doi: 10.1007/s12021-024-09653-x  175. Chantanachai T, Sturnieks DL, Lord SR, Menant J, Delbaere K, Sachdev PS, et al. Cognitive and physical declines and falls in older people with and without mild cognitive impairment: a 7-year longitudinal study. International Psychogeriatrics. 2024;36(4):306-16. doi: 10.1017/S1041610223000315  176. Chauhan P, Das SK. Efficacy of Simultaneous Application of Repetitive Transcranial Magnetic Stimulation and Virtual Reality Training on Sensory-motor and Cognitive Deficits among Stroke Patients: a Protocol for a Randomised Controlled Trial. Journal of clinical and diagnostic research. 2024;18(7):YK05‐YK8. doi: 10.7860/JCDR/2024/68577.19686 PMID:CN-02733970  177. Chen J, Park J-H, Lin C-Y, Lai T-F, Kim D-R, Shin M-J, et al. Whole-Body and Segmental Phase Angles and Cognitive Function in the Older Korean Population: Cross-Sectional Analysis. Jmir Public Health and Surveillance. 2024;10. doi: 10.2196/63457 PMID:WOS:001390229800001  178. Chen R, Chen H, Li S, Cao P, He L, Zhao Y, et al. The impact of ankle-foot orthoses on mobility of dual-task walking in stroke patients? A cross-sectional two-factor factorial design clinical trial. Neuropsychological rehabilitation. 2024:1‐25. doi: 10.1080/09602011.2024.2343155 PMID:CN-02693698  179. Chen S, Ou R, Wei Q, Fu J, Zhao B, Chen X, et al. Identification of risk factors and development of a predictive nomogram for sarcopenia in Alzheimer's disease. Alzheimers & Dementia. 2025;21(2). doi: 10.1002/alz.14503 PMID:WOS:001392697100001  180. Chen X. Research on Risk Factors and Predictive Models for Sarcopenic Dysphagia in the Elderly. Master's Degree, Chongqing Medical University. 2024. Available from:https://link.cnki.net/doi/10.27674/d.cnki.gcyku.2024.000598  181. Chen Y, Liao J, Zeng Y, Ma H, Jiang C, Yu S, et al. The combined effect of diabetes mellitus and sarcopenia on depression and cognitive function: insights from the CHARLS cohort, 2011–2020. European Geriatric Medicine. 2024;15(6):1881-90. doi: 10.1007/s41999-024-01039-1  182. Chen Y, Nie C, Wei X, Dan X, Zhang N, Che L, et al., editors. The Combined Predictive Value of Depressive Mood and Frailty Status on Sarcopenia. The 6th Shanghai International Nursing Conference; 2024; Shanghai, China.  183. Chen Y, Yu X, Lu C, Shi W. Brain Function Status may be Related to Pelvic Floor Function and Pregnancy Weight Gain in Postpartum Women with Pelvic Floor Dysfunction. Clinical and Experimental Obstetrics and Gynecology. 2024;51(11). doi: 10.31083/j.ceog5111249  184. Chen ZJ, Tang FP, Chang SY, Chung HL, Tsai WH, Chou SS, et al. Resilience-happiness nexus in community-dwelling middle-aged and older adults: Results from Gan-Dau Healthy Longevity Plan. Archives of Gerontology and Geriatrics. 2024;116. doi: 10.1016/j.archger.2023.105162  185. Cheng Y, Arteaga-Reyes C, Clancy U, Garcia DJ, Valdés Hernández MDC, Thrippleton MJ, et al. Clinical Relevance of ‘Cap’ and ‘Track’ Development after Recent Small Subcortical Infarct. Annals of Neurology. 2025. doi: 10.1002/ana.27182  186. Chew J, Tan CH, Chew P, Ng KP, Ali N, Lim WS. Cognitive frailty in older adults: examining the impact of frailty criteria on neuropsychological profile, functional outcomes, activity levels, and quality of life. Eur Geriatr Med. 2024;15(6):1803-15. doi: 10.1007/s41999-024-01040-8 PMID:39287749  187. ChiCtr. The effect of vitamin K2 fortified nutritional intervention on muscle condition and clinical outcomes in elderly patients with muscle wasting syndrome. https://trialsearchwhoint/Trial2aspx?TrialID=ChiCTR2400081931. 2024. doi: PMID:CN-02690554  188. ChiCtr. Clinical study on the treatment of postpartum rectus abdominis separation (DRA) with pelvic floor neuromuscular stimulator combined with Bo's abdominal acupuncture. https://trialsearchwhoint/Trial2aspx?TrialID=ChiCTR2400091828. 2024. doi: PMID:CN-02782787  189. ChiCtr. Investigating the Construction of an Elderly Comprehensive Continuous Health Service System through the Integration of Artificial Intelligence and Health Medical Big Data Platform. https://trialsearchwhoint/Trial2aspx?TrialID=ChiCTR2400086823. 2024. doi: PMID:CN-02744081  190. ChiCtr. A prospective, double-blind, randomized controlled study evaluating the improvement of nutritional status with silkworm pupa tablets compared with placebo among Alzheimer's Disease. https://trialsearchwhoint/Trial2aspx?TrialID=ChiCTR2500095304. 2025. doi: PMID:CN-02801158  191. Choi JY, Lee HY, Lee JH, Hong Y, Park SK, Ryu DR, et al. Characteristics According to Frailty Status Among Older Korean Patients With Hypertension. Journal of Korean medical science. 2024;39(10):e84. doi: 10.3346/jkms.2024.39.e84 PMID:CN-02673188  192. Choi Y-A. Handgrip Strength and Cognitive Recovery in Older Stroke Survivors: A Prospective Study. Medicina-Lithuania. 2024;60(10). doi: 10.3390/medicina60101697 PMID:WOS:001341914100001  193. Chong E, Goh EF, Lim WS. Functional Dependency as a Marker for Positive SARC-F Screen among Older Persons at the Emergency Department. Annals of Geriatric Medicine and Research. 2024;28(4):401-9. doi: 10.4235/agmr.24.0091  194. Chu H, Huang C, Guan Y, Xie F, Chen M, Guo Q. The associations between nutritional status and physical frailty and Alzheimer's disease plasma biomarkers in older cognitively unimpaired adults with positive of amyloid-β PET. Clin Nutr. 2024;43(7):1647-56. doi: 10.1016/j.clnu.2024.05.024 PMID:38810424  195. Ciatto L, Dauccio B, Tavilla G, Bartolomeo S, Lo Buono V, De Cola MC, et al. Improving manual dexterity using ergonomic wearable glove in patients with multiple sclerosis: a quasi-randomized clinical trial. Multiple sclerosis and related disorders. 2024;92:105938. doi: 10.1016/j.msard.2024.105938 PMID:CN-02762351  196. Coca-Pulido A, Solis-Urra P, Fernandez-Gamez B, Olvera-Rojas M, Bellón D, Sclafani A, et al. Fitness, Gray Matter Volume, and Executive Function in Cognitively Normal Older Adults: cross-Sectional Findings From the AGUEDA Trial. Scandinavian journal of medicine & science in sports. 2024;34(10):e14746. doi: 10.1111/sms.14746 PMID:CN-02778686  197. Colcord KA, Gilsanz P, George KM, Kawas CH, Jiang L, Whitmer RA, et al. The Importance of Racially and Ethnically Inclusive Gait Speed Reference Values in Individuals 90 Years and Older: LifeAfter90. Journal of geriatric physical therapy (2001). 2024;47(4):202-13. doi: 10.1519/JPT.0000000000000416  198. Cooley S, Nelson BM, Rosenow A, Westerhaus E, Cade WT, Reeds DN, et al. Exercise Training to Improve Brain Health in Older People Living With HIV: study Protocol for a Randomized Controlled Trial. JMIR research protocols. 2023;12:e41421. doi: 10.2196/41421 PMID:CN-02637758  199. Cramer SC, Parodi L, Moslemi Z, Braun RG, Aldridge CM, Shahbaba B, et al. Genetic Variation and Stroke Recovery: The STRONG Study. Stroke. 2024;55(8):2094-102. doi: 10.1161/strokeaha.124.047643 PMID:38979623  200. Crowe C, Naughton C, de Foubert M, Cummins H, McCullagh R, Skelton DA, et al. Treatment effect analysis of the Frailty Care Bundle (FCB) in a cohort of patients in acute care settings. Aging Clin Exp Res. 2024;36(1):187. doi: 10.1007/s40520-024-02840-5 PMID:39254891  201. Ctri. Detailed risk evaluation for back pain in dentist and results of new posture feedback device along with physiotherapy exercise as treatment to reduce pain and enhance physical function in dentist having back pain. https://trialsearchwhoint/Trial2aspx?TrialID=CTRI/2024/03/064774. 2024. doi: PMID:CN-02684379  202. Cui M, Wang J, Deng M, Meng H, Fan Y, Ku C, et al. Longitudinal relationship between grip strength and cognitive function in a European population older than 50 years: A cross-lagged panel model. Arch Gerontol Geriatr. 2024;122:105396. doi: 10.1016/j.archger.2024.105396 PMID:38484671  203. Cui Y, Xu Z, Cui Z, Guo Y, Wu P, Zhou X. Development and validation of a frailty risk model for patients with mild cognitive impairment. Scientific reports. 2025;15(1):3814. doi: 10.1038/s41598-025-88275-y  204. Dag B, Naz I, Felekoglu E, Emuk Y, Kopruluoglu M, Sahin H. Associations of Upper-Extremity Exercise Capacity and Grip Strength With Cognitive Domains in Patients With COPD. Respiratory Care. 2024;69(5):595-602. doi: 10.4187/respcare.11610  205. Damm M, George K, Rosendahl J, Greinert R. Subclinical Cognitive Impairment in Chronic Pancreatitis Is Associated With Reduced Mobility and Quality of Life. Clin Transl Gastroenterol. 2024;15(4):e00685. doi: 10.14309/ctg.0000000000000685 PMID:38299610  206. de Andrade ML, Silva SM, de Moraes JF, de Jesus SC, Dos Santos Pedro E, Corrêa JCF, et al. Predictors of the Social Participation of Adults with Down Syndrome: An Analysis Based on the Biopsychosocial Model of the International Classification of Functioning, Disability, and Health. American journal of physical medicine & rehabilitation. 2024;103(3):238-44. doi: 10.1097/PHM.0000000000002341  207. de Assunção Cortez Corrêa LC, Raffin J, Vellas B, Guerra RO, de Souto Barreto P. Sex-specific associations of neurodegeneration and inflammatory biomarkers with intrinsic capacity in older adults: findings from the 4-year longitudinal Multidomain Alzheimer's Prevention Trial (MAPT). Maturitas. 2025;193:108191. doi: 10.1016/j.maturitas.2024.108191 PMID:CN-02802800  208. Debabi C, Yedes A, Njima OB, Methneni M, Ketata H, Mazlout M, et al. QUALITY OF RECOVERY AFTER HIP FRACTURE SURGERY: PERICAPSULAR NERVE GROUP BLOCK VERSUS FASCIA ILIACA COMPARTMENT BLOCK. Regional anesthesia and pain medicine. 2023;48:A23‐A4. doi: 10.1136/rapm-2023-ESRA.40 PMID:CN-02698573  209. Debeaudrap P, Etoundi N, Tegbe J, Assoumou N, Dialo Z, Tanon A, et al. The association between HIV infection, disability and lifestyle activity among middle-aged and older adults: an analytical cross-sectional study in Ivory Coast (the VIRAGE study). Bmc Public Health. 2024;24(1). doi: 10.1186/s12889-024-19020-9 PMID:WOS:001241792600001  210. Deeg DJH, Hoogendijk EO, van Schoor NM, Schaap LA, Lima Passos V. Joint Trajectories of Performance-Based and Self-Reported Physical Functioning in Older Adults: A 20-Year Longitudinal Study in the Netherlands. Journal of aging and health. 2024:8982643241273298. doi: 10.1177/08982643241273298  211. Del Corral T, Menor-Rodríguez N, Fernández-Vega S, Díaz-Ramos C, Aguilar-Zafra S, López-de-Uralde-Villanueva I. Longitudinal study of changes observed in quality of life, psychological state cognition and pulmonary and functional capacity after COVID-19 infection: A six- to seven-month prospective cohort. Journal of clinical nursing. 2024;33(1):89-102. doi: 10.1111/jocn.16352  212. Di Cesare F, Mancuso J, Silver B, Loudon PT. Assessment of Cognitive and Neurologic Recovery in Ischemic Stroke Drug Trials: results from a Randomized, Double-blind, Placebo-controlled Study. Innovations in clinical neuroscience. 2016;13(9‐10):32‐43. doi: PMID:CN-02643713  213. Diabetes编辑部 J. 2024年Journal of Diabetes第3期中文导读. 中华内分泌代谢杂志. 2024;40(4). doi:  214. Dias CRC, Rocha SV, Cardoso JP, Ferreira BMP, Conceição AF, Dos Santos CA. INDICATORS OF FUNCTIONAL FITNESS AND COGNITIVE COMMITMENT IN ELDERLY. Revista Brasileira de Medicina do Esporte. 2024;30. doi: 10.1590/1517-8692202430022022_0414i  215. Dias NAGP. Effects of a Physical Exercise Program on Fine and Gross Motor Coordination in Untrained Elderly People2024.  216. Diaz Posada NA, Cano Rosales DJ, Amaya Muñoz MC, Buitrago Gomez MA, Villabona SJ, Camacho López PA. Handgrip Evaluation Before and After Pulmonary Rehabilitation Therapy in Patients With Chronic Obstructive Pulmonary Disease (COPD). Cureus. 2024;16(9):e69404. doi: 10.7759/cureus.69404 PMID:39403653  217. Dong Q, Li Y, Song Y, Zhang Y, Han X, Ren Y, et al. Cognitive Reserve Relationship with Physical Performance in Dementia-Free Older Adults: The MIND-China Study. Journal of Alzheimer's Disease Reports. 2024;8(1):1329-38. doi: 10.3233/ADR-240064  218. Dong X, Yu Y, Li J, Chai X, Shan W, Yan H, et al. A study of the correlation between sarcopenia and cognitive impairment in older individuals over 60 years: cross-sectional and longitudinal validation. Frontiers in Aging Neuroscience. 2024;16. doi: 10.3389/fnagi.2024.1489185 PMID:WOS:001375445800001  219. Dreyer S, Lewis C, Fahey K, Martin H, Pavone L, Anastasopoulos S, et al. The relationship between patient-specific factors and functional progression of COVID-19 survivors admitted to an inpatient rehabilitation facility. PM and R. 2024;16(11):1231-9. doi: 10.1002/pmrj.13187  220. Drks. Effect of daily taurine supplementation for 6 months on age and metabolic biomarkers as well as physical fitness in 55-75-year-old women and men: the TauAge Study. https://trialsearchwhoint/Trial2aspx?TrialID=DRKS00035066. 2024. doi: PMID:CN-02752880  221. Egger M, Finsterhölzl M, Buetikofer A, Wippenbeck F, Müller F, Jahn K, et al. Balance function in critical illness survivors and evaluation of psychometric properties of the Mini-BESTest. Scientific reports. 2024;14(1):12089. doi: 10.1038/s41598-024-61745-5  222. Eligulashvili A, Gordon M, Lee JS, Lee J, Mehrotra-Varma S, Mehrotra-Varma J, et al. Long-term outcomes of hospitalized patients with SARS-CoV-2/COVID-19 with and without neurological involvement: 3-year follow-up assessment. PLoS Medicine. 2024;21(4 April). doi: 10.1371/journal.pmed.1004263  223. Ergene TY, Akay Ü, Karadibak D, Özsoy İ. A Comparative Study of Cognitive and Motor Performance in Liver Recipients. Prog Transplant. 2024;34(3):103-10. doi: 10.1177/15269248241268716 PMID:39106359  224. Euctr BE. The effect of corticosteroids on early recovery after major surgery in elderly patients_ CORTERAS STUDY. https://trialsearchwhoint/Trial2aspx?TrialID=EUCTR2021-004737-37-BE. 2021. doi: PMID:CN-02771050  225. Euctr DK. A research study looking at the effect of semaglutide on the immune system and other biological processes in people with Alzheimer’s disease. https://trialsearchwhoint/Trial2aspx?TrialID=EUCTR2022-003384-24-DK. 2023. doi: PMID:CN-02629169  226. Fabea L, Intiful FD, Hatsu IE, Larry-Afutu J, Boateng L. Body composition is related to cognitive function among young adults in Ghana. BMC psychology. 2024;12(1):114. doi: 10.1186/s40359-024-01569-0  227. Farajnia S, Rajabi H, Ghaffari M, Beladi-Moghadam N, Fayazmilani R. Impact of cognitive-aerobic exercise training on brain-derived neurotrophic factor, dual-tasking abilities, and mood state in individuals with multiple sclerosis. Physiology & behavior. 2025;290:114756. doi: 10.1016/j.physbeh.2024.114756 PMID:CN-02788304  228. Faria LO, de Sousa Fortes L, Albuquerque MR. The Influence of Mental Fatigue on Physical Performance and Its Relationship with Rating Perceived Effort and Enjoyment in Older Adults. Research quarterly for exercise and sport. 2024:1‐15. doi: 10.1080/02701367.2024.2409932 PMID:CN-02770696  229. Feijen S, Burtin C, Gillebert CR, Bogaerts K. Cognitive, psychological and physical functioning in post COVID-19 patients with different levels of fatigue: a descriptive abstract. 2024.  230. Ferreira CFS. Associação entre sarcopenia, tempo sentado e atividade física em pessoas idosasAssociation Between Sarcopenia, Sitting Time and Physical Activity in Elderly People2024.  231. Ferreira MEC, Lima-Junior D, Faro H, Roelands B, Fortes LS. Prolonged cognitive effort impairs inhibitory control and causes significant mental fatigue after an endurance session with an auditive distractor in professional soccer players. Psychology of sport and exercise. 2024;70. doi: 10.1016/j.psychsport.2023.102533 PMID:CN-02745012  232. Flynn S, Stein ML, Peyton J, Park R, Staffa S, Bernier R, et al. The Effect of Just-In-Time Rapid Cycle Deliberate Practice (JIT-RCDP) Simulation Training on Pediatric Anesthesia Trainee Cognitive Task Load During Infant Intubation. Anesthesia and analgesia. 2023;136(5):746‐9. doi: 10.1213/01.ane.0000977652.27377.97 PMID:CN-02773607  233. Fogarty M. Ameliorating Synaptic and Mitochondrial Dysfunctions of the Respiratory Neuromotor System in Alzheimer's Disease. 2023. doi: PMID:GRANTS:17350002  234. Friedmann E, Gee NR, Simonsick EM, Kitner-Triolo MH, Resnick B, Gurlu M, et al. Pet Attachment and Maintenance of Physical and Cognitive Function in Community-Residing Older Adults: Evidence From the Baltimore Longitudinal Study of Aging (BLSA). Anthrozoos. 2025;38(1):153-69. doi: 10.1080/08927936.2024.2395125  235. Fujita T, Kasahara R, Kurita M, Jinbo R, Yamamoto Y, Ohira Y, et al. Vitality index predicts walking independence in patients with hip fracture A retrospective study. Medicine (United States). 2024;103(51):e41042. doi: 10.1097/MD.0000000000041042  236. Fukase Y, Kamide N, Sakamoto M, Ando M, Ichikura K, Shiba Y, et al. An in-person survey of the influence of the COVID-19 pandemic on physical function, functional capacity, cognitive function, and mental health among community-dwelling older adults in Japan from 2016 to 2022. BMC Geriatr. 2024;24(1):457. doi: 10.1186/s12877-024-05055-5 PMID:38789923  237. Funaki T, Saji M, Higuchi R, Takamisawa I, Nanasato M, Tamura H, et al. Impact of osteoporotic risk in men undergoing transcatheter aortic valve replacement: a report from the LAPLACE-TAVI registry. Cardiovasc Interv Ther. 2024;39(4):460-7. doi: 10.1007/s12928-024-01011-7 PMID:38755510  238. Gao P, Ma LZ, Tan L, Yu JT. Physical Frailty, Genetic Predisposition, and Incident Dementia: A Prospective Cohort Study of 274,194 Adults. Neurology. 2024;102(17). doi: 10.1212/WNL.0000000000204357  239. Gao R, Zhan M, Ke S, Wu K, He G, Qi L, et al. Potential risk factors for mild cognitive impairment among patients with type 2 diabetes experiencing hypoglycemia. Diabetes Res Clin Pract. 2024;207:111036. doi: 10.1016/j.diabres.2023.111036 PMID:38049036  240. Gao WX, Liu J, Wang J, Jin YL, Yeung SLA, Lam TH, et al. Association of intrinsic capacity with incident type 2 diabetes mellitus in older Chinese: Guangzhou Biobank Cohort Study. Archives of Gerontology and Geriatrics. 2025;129. doi: 10.1016/j.archger.2024.105687  241. Garbsch R, Schäfer H, Kotewitsch M, Mooren JM, Waranski M, Teschler M, et al. Sex-specific differences of cardiopulmonary fitness and pulmonary function in exercise-based rehabilitation of patients with long-term post-COVID-19 syndrome. BMC Medicine. 2024;22(1). doi: 10.1186/s12916-024-03658-8  242. Garbsch R, Schäfer H, Mooren FC, Schmitz B. Analysis of fat oxidation capacity during cardiopulmonary exercise testing indicates long-lasting metabolic disturbance in patients with post-covid-19 syndrome. Clinical Nutrition. 2024;43(12):26-35. doi: 10.1016/j.clnu.2024.10.010  243. García-Agustin D, Rodríguez-Rodríguez V. Association between gait speed deterioration and EEG abnormalities. Age and Ageing. 2024;53:iii23. doi: 10.1093/ageing/afae139.087  244. Garofalo M, Panicucci C, Imarisio A, Nuzzo T, Brolatti N, De Stefano ME, et al. D- and L-amino acid blood concentrations are affected in children with Duchenne muscular dystrophy. Biochimica Clinica. 2024;1:S37. doi:  245. Ge H, Yang S, Su W, Guan W, Dong S, Chang W, et al. The relationship between sarcopenia and mental health status in Chinese older adults: the mediating role of activities of daily living. Bmc Geriatrics. 2025;25(1). doi: 10.1186/s12877-025-05723-0 PMID:WOS:001410771100001  246. Ge Y, You Q, Gao F, Liu G, Wang L, Li B, et al. Muscle density, but not size, is independently associated with cognitive health in older adults with hip fractures. JBMR Plus. 2024;8(5). doi: 10.1093/jbmrpl/ziae047  247. Genovesi A, Barichella M, Cereda E, Ferri V, Bolliri C, Pusani C, et al. Sarcopenia in patients with parkinsonian syndromes: relationship with cognitive functions, disease-related fatigue and quality of life. 2024. p. 991.  248. Gharibzadeh S, Routen A, Razieh C, Zaccardi F, Lawson C, Gillies C, et al. Long term health outcomes in people with diabetes 12 months after hospitalisation with COVID-19 in the UK: a prospective cohort study. eClinicalMedicine. 2025;79. doi: 10.1016/j.eclinm.2024.103005  249. Giudici KV, de Souto Barreto P, Guyonnet S, Beard JR, Takeda C, Cantet C, et al. Predictive Capacity of the Integrated Care for Older People Screening Tool for Intrinsic Capacity Impairments: Results From the INSPIRE-T Cohort. The journals of gerontology Series A, Biological sciences and medical sciences. 2024;79(7). doi: 10.1093/gerona/glae112  250. Goggin KP, Lu L, Lee DE, Howell CR, Srivastava D, Brinkman TM, et al. Severe Sepsis During Treatment for Childhood Leukemia and Sequelae Among Adult Survivors. JAMA Network Open. 2024;7(3):E242727. doi: 10.1001/jamanetworkopen.2024.2727  251. Gökçe E, Kaushal N, Fontanille T, Vrinceanu T, Saillant K, Vints WAJ, et al. The mediating role of lower body muscle strength and IGF-1 level in the relationship between age and cognition. A MIDUS substudy. Experimental Gerontology. 2024;189. doi: 10.1016/j.exger.2024.112399  252. Gonabal V, Aggarwal S, Rani D, Panwar M. Comparison of ultrasound-guided suprainguinal fascia iliaca compartment block and pericapsular nerve group block for postoperative analgesia and associated cognitive dysfunction following hip and proximal femur surgery. Journal of anaesthesiology, clinical pharmacology. 2024;40(3):432‐8. doi: 10.4103/joacp.joacp_230_23 PMID:CN-02747866  253. González-Fernández FT, Castillo-Rodriguez A, González-Víllora S, Hortigüela-Alcalá D. The Influence of a Warm-Up on Vigilance in University Students. Motor Control. 2024;28(1):78-90. doi: 10.1123/mc.2023-0027 PMID:37931618  254. Gordon JI, Wang C, Yilmaz AS, Peng J, Bednash JS, Brummel NE. Associations Between Biomarkers of Inflammation at Hospital Discharge With Cognitive and Physical Function in Survivors of Critical Illness. American Journal of Respiratory and Critical Care Medicine. 2024;209. doi:  255. Grasset L, Bouteloup V, Cacciamani F, Pellegrin I, Planche V, Chêne G, et al. Associations between Blood-Based Biomarkers and Cognitive and Functional Trajectories among Participants of the MEMENTO Cohort. Neurology. 2024;102(9). doi: 10.1212/WNL.0000000000209307  256. Griffen C, Cullen T, Hattersley J, Weickert MO, Dallaway A, Duncan M, et al. Effects of resistance exercise and whey protein supplementation on cognitive function in older men: secondary analysis of a randomised, double-blind, placebo-controlled trial. Experimental gerontology. 2024;193:112477. doi: 10.1016/j.exger.2024.112477 PMID:CN-02708599  257. Gu Y. The Impact of Preoperative Sarcopenia on Perioperative Neurocognitive Disorders. Master's Degree, Chengdu Medical College. 2024. Available from:https://d.wanfangdata.com.cn/thesis/ChhUaGVzaXNOZXdTMjAyNDA5MjAxNTE3MjUSCUQwMzY1OTAyNRoIbHFtcWl0a3A%3D  258. Guan Z, Stephan BCM, Donini LM, Prado CM, Sim M, Siervo M. Exploring the Association between Sarcopenic Obesity and Cardiovascular Risk: A Summary of Findings from Longitudinal Studies and Potential Mechanisms. Proceedings of the Nutrition Society. 2024. doi: 10.1017/S0029665124007559  259. Guazzarini AG, Mancinetti F, Bastiani P, Scamosci M, Cecchetti R, Boccardi V, et al. Tai chi, irisin and cognitive performance: a clinical and biological investigation in older adults. Aging Clin Exp Res. 2024;36(1):90. doi: 10.1007/s40520-024-02743-5 PMID:38598000  260. Guimarães CM, Filho ALLN, Perrucho F, Dias C, Aguiar C, Meira e Cruz M, et al. Neck circumference, epworth sleepiness scale, and their relation with falling in robust older adults aged > 65 years. Sleep Medicine. 2024;115:77. doi: 10.1016/j.sleep.2023.11.243  261. Guo J, Zhang Y, Yang Y, Lin L, Shen T. Prevalence and risk factors of cognitive frailty in patients with cardiovascular disease: A hospital-based cross-sectional study. Medicine. 2024;103(49). doi: 10.1097/md.0000000000040761 PMID:WOS:001377183300047  262. Gupta A, Nicholas R, McGing JJ, Nixon AV, Mallinson JE, McKeever TM, et al. DYNamic Assessment of Multi-Organ level dysfunction in patients recovering from COVID-19: DYNAMO COVID-19. Experimental Physiology. 2024;109(8):1274-91. doi: 10.1113/EP091590  263. Guzman-Carreras A, San Miguel-Agudo J, Paz-Cabezas M, Bernabeu-Wittel M, Munoz-Rivas N, Sanchez-Sauce B, et al. Low Muscle Strength Assessed with Dynamometry in Elderly Polypathological Patients with Acute Heart Failure: PROFUND-IC Registry. Journal of Clinical Medicine. 2024;13(16). doi: 10.3390/jcm13164873 PMID:WOS:001307224200001  264. Haeri NS, Perera S, Nadkarni NK, Greenspan SL. Association of inflammatory markers with muscle and cognitive function in early and late-aging older adults. Journal of Nutrition, Health and Aging. 2024;28(5). doi: 10.1016/j.jnha.2024.100207  265. Hajek A, König HH. Factors Leading to a Decrease in Grip Strength Among the Oldest Old: A Large, Representative, Longitudinal Survey. Journal of the American Medical Directors Association. 2024;25(4):672-5. doi: 10.1016/j.jamda.2023.08.021  266. Hallberg S, Söreskog E, Borgström F, Cederholm T, Hedström M. Association between institutionalization by 4 months post-discharge walking capacity and lean body mass in elderly hip fracture patients: Evidence from a Swedish Registry Based Study. SAGE Open Medicine. 2024;12. doi: 10.1177/20503121241258409  267. Halvorson BD, Bao Y, Singh KK, Frisbee SJ, Hachinski V, Whitehead SN, et al. Thromboxane-induced cerebral microvascular rarefaction predicts depressive symptom emergence in metabolic disease. Journal of applied physiology (Bethesda, Md : 1985). 2024;136(1):122-40. doi: 10.1152/japplphysiol.00410.2023  268. Hamill SN, Dong Y, Zhu H, Huang Y, Soares A, Waller JL, et al. Biomarkers. Alzheimer's & dementia. 2024;20 Suppl 2:e083897. doi: 10.1002/alz.083897 PMID:CN-02796242  269. Han SJ, Suh JH. Association between language function and body composition characteristics in patients with subacute left hemispheric stroke. Neurology Asia. 2023;28(4):917-26. doi: 10.54029/2023upt PMID:WOS:001167427800015  270. Hao W, Shan YF, Kimura T, Ukawa S, Ohira H, Okabayashi S, et al. Dual decline in subjective gait speed and domain-specific cognition is associated with higher risk of incident dementia in older Japanese adults: A 15-year age-specific cohort study. Archives of Gerontology and Geriatrics. 2024;117. doi: 10.1016/j.archger.2023.105254  271. Harrison WT, Cline JM, Caudell DL, Huber HF, Shively CA, Register TC, et al. Alzheimer disease-like neuropathologic changes in a geriatric baboon (Papio hamadryas). Journal of Veterinary Science. 2024;25(5). doi: 10.4142/jvs.24080  272. Hatanaka S, Osuka Y, Kojima N, Motokawa K, Hayakawa M, Mikami Y, et al. Relationship between phase angle and lower-extremity function in older adults: Itabashi Longitudinal Study on Aging. Nutrition. 2024;119. doi: 10.1016/j.nut.2023.112289  273. Hatanaka S, Sasai H, Shida T, Osuka Y, Kojima N, Ohta T, et al. Association between dynapenia and cognitive decline in community-dwelling older Japanese adults: The IRIDE Cohort Study. Geriatrics and Gerontology International. 2024;24(S1):123-9. doi: 10.1111/ggi.14749  274. Hayes E, Dent E, Shannon OM, Zhong LZ, Bozanich T, Blekkenhorst LC, et al. Lower intake of plant-derived nitrate is associated with higher odds of frailty: a cross- sectional study in community-dwelling older women. Proceedings of the Nutrition Society. 2024;83(OCE2):E234. doi: 10.1017/S0029665124004476  275. He M, Lian T, Guo P, Zhang Y, Huang Y, Qi J, et al. Association between nutritional status and gait performance in Alzheimer's disease. CNS Neuroscience and Therapeutics. 2024;30(4). doi: 10.1111/cns.14502  276. Hermansen M, Nygaard M, Tan Q, Jeune B, Semkovska M, Christensen K, et al. Cognitively high-performing oldest old individuals are physically active and have strong motor skills-A study of the Danish 1905 and 1915 birth cohorts. Arch Gerontol Geriatr. 2024;122:105398. doi: 10.1016/j.archger.2024.105398 PMID:38460266  277. Hettiarachchi J, Verstraeten LMG, Paci J, Reijnierse EM, Meskers CGM, Maier AB. Body Weight and Composition Changes in Geriatric Rehabilitation Are Dependent on Sarcopenia and Malnutrition: RESORT. Journal of the American Medical Directors Association. 2024;25(8). doi: 10.1016/j.jamda.2024.105030 PMID:WOS:001253765200001  278. Honma K, Honda Y, Nagase M, Nakao Y, Sota K, Sasanuma N, et al. Pre-stroke patient characteristics that influence skeletal muscle quality: A cross-sectional study. Geriatrics and Gerontology International. 2025;25(2):213-9. doi: 10.1111/ggi.15060  279. Hopkins J, McVeigh J, Hill K, Ellis KA, Jacques A, Burton E. Associations between physical activity, sedentary behaviour and cognitive domain performance of people living with mild cognitive impairment in the community. Australian occupational therapy journal. 2024;71(4):527‐39. doi: 10.1111/1440-1630.12944 PMID:CN-02734308  280. Hosoya M, Toi S, Yoshizawa H, Kitagawa K. Slow Gait Speed Predicts Incident Dementia, Mortality, and Long-Term Functional Outcome in Cerebral Small-Vessel Disease. J Alzheimers Dis. 2024;101(2):499-508. doi: 10.3233/jad-240304 PMID:39213068  281. Hsieh P-I, Huang T-H, Chiou J-M, Chen J-H, Chen Y-C. Cohort profile: the Taiwan Initiative for Geriatric Epidemiological Research- a prospective cohort study on cognition. Epidemiology and Health. 2024;46. doi: 10.4178/epih.e2024057 PMID:WOS:001338448200003  282. Hsu P-S, Lee W-J, Peng L-N, Lu W-H, Meng L-C, Hsiao F-Y, et al. Safeguarding vitality and cognition: The role of sarcopenia in intrinsic capacity decline among octogenarians from multiple cohorts. Journal of Nutrition Health & Aging. 2024;28(6). doi: 10.1016/j.jnha.2024.100268 PMID:WOS:001290929600001  283. Hu W, Zhang WB, Liu BP, Jia CX. Associations and Mediating Pathways Between Childhood Adversity and Risk of Dementia: A Cohort Study in the UK Biobank. The journals of gerontology Series A, Biological sciences and medical sciences. 2024;79(8). doi: 10.1093/gerona/glae121  284. Hu Z, Tang L, Zhan Y. Cognition as mediator of pulmonary function and risk of sarcopenia among older adults. BMC public health. 2024;24(1):1347. doi: 10.1186/s12889-024-18848-5  285. Huang L. Distribution characteristics and related factors analysis of traditional Chinese medicine syndrome elements in Parkinson's disease. Master's Degree, Fujian University of Traditional Chinese Medicine. 2024. Available from:https://d.wanfangdata.com.cn/thesis/ChhUaGVzaXNOZXdTMjAyNDA5MjAxNTE3MjUSCFk0MzQyMDIzGghscW1xaXRrcA%3D%3D  286. Huang L, Shen X, Zou Y, Wang Y. Effects of BMI and grip strength on older adults' falls-A longitudinal study based on CHARLS. Frontiers in Public Health. 2024;12. doi: 10.3389/fpubh.2024.1415360 PMID:WOS:001378897000001  287. Huang Y, Zhang R, Hong X, Liu S, Zhang S, Guo M, et al. Correlation between sarcopenia index and cognitive function in older adult women: A cross-sectional study using NHANES data. Journal of Clinical Neuroscience. 2024;122:73-9. doi: 10.1016/j.jocn.2024.02.026  288. Humphry N, Wilson T, Bye K, Draper J, Hewitt J. Feasibility of screening for frailty, sarcopenia and nutritional status in elective surgery for colorectal cancer. Age and Ageing. 2024;53:25-. doi: 10.1093/ageing/afae139.093 PMID:WOS:001288218000048  289. Ikeda K, Tanaka K, Tajima S, Takakura T, Sugihara M, Ono K. Dizziness and unstable gait in the older adults are associated with vestibular hypofunction, muscle dysfunction and sleep disturbance: impact on prevention of accidental falls. BMC Geriatr. 2024;24(1):1042. doi: 10.1186/s12877-024-05620-y PMID:39731063  290. Imbalzano G, Ledda C, Tangari MM, Artusi CA, Montanaro E, Rizzone MG, et al. Unraveling the stride: exploring the influence of neurogenic orthostatic hypotension on gait and balance in Parkinson’s disease. Clinical Autonomic Research. 2024;34(6):593-601. doi: 10.1007/s10286-024-01071-y  291. Isaia G, Presta R, Brunetti E, Cacciatore CM, Carbonara F, Berardo E, et al. Nutritional screening on hospital admission and one-year clinical outcomes in a prospective cohort of older patients. Clinical Nutrition ESPEN. 2024;64:221-8. doi: 10.1016/j.clnesp.2024.10.006  292. Ishimoto R, Mutsuzaki H, Shimizu Y, Takeuchi R, Matsumoto S, Hada Y. Association between Sarcopenia and Balance in Patients Undergoing Inpatient Rehabilitation after Hip Fractures: A Retrospective Cohort Study. Medicina (Kaunas, Lithuania). 2024;60(5). doi: 10.3390/medicina60050742  293. Isrctn. Personalised exercise rehabilitation for people with multiple long-term conditions: main Trial. https://trialsearchwhoint/Trial2aspx?TrialID=ISRCTN59323331. 2024. doi: PMID:CN-02763997  294. Isrctn. Investigating and optimising physical function with weight loss. https://trialsearchwhoint/Trial2aspx?TrialID=ISRCTN10203365. 2024. doi: PMID:CN-02756760  295. Isrctn. A clinical trial to investigate how the DailyColors(TM) dietary supplement affects markers of health in older adults. https://trialsearchwhoint/Trial2aspx?TrialID=ISRCTN10734674. 2024. doi: PMID:CN-02785763  296. Isrctn. Exercise responses with transcutaneous spinal cord stimulation (using electrodes placed on the skin surface) following spinal cord injury. https://trialsearchwhoint/Trial2aspx?TrialID=ISRCTN17856698. 2024. doi: PMID:CN-02663841  297. Ito N, Nishioka E, Yunoki N, Momoki C, Oyamada H, Urata Y, et al. Factors contributing to frailty in institutionalized older adults: a multi-institutional cross-sectional study. Nihon Ronen Igakkai zasshi Japanese journal of geriatrics. 2024;61(3):345-54. doi: 10.3143/geriatrics.61.345  298. Jakobsson J, Burtin C, Hedlund M, Boraxbekk CJ, Westman J, Karalija N, et al. Effects and mechanisms of supramaximal high-intensity interval training on extrapulmonary manifestations in people with and without chronic obstructive pulmonary disease (COPD-HIIT): study protocol for a multi-centre, randomized controlled trial. Trials. 2024;25(1):664. doi: 10.1186/s13063-024-08481-3 PMID:39375781  299. Jehu DA, Langston R, Sams R, Young L, Hamrick M, Zhu H, et al. The Impact of Dual-Tasks and Disease Severity on Posture, Gait, and Functional Mobility among People Living with Dementia in Residential Care Facilities: A Pilot Study. Sensors (Basel, Switzerland). 2024;24(9). doi: 10.3390/s24092691  300. Jelaska J, Vučković M, Gugić Ordulj I, Kolak E, Šolić Šegvić L, Đapić Kolak Z, et al. Unlocking Cognitive Potential: Association of Sarcopenia and Mediterranean Diet on Cognitive Function in Community-Dwelling Elderly of the Dalmatian Region. Nutrients. 2024;16(7). doi: 10.3390/nu16070991  301. Jerez-Roig J, Farrés-Godayol P, Yildirim M, Escribà-Salvans A, Moreno-Martin P, Goutan-Roura E, et al. Prevalence of urinary incontinence and associated factors in nursing homes: a multicentre cross-sectional study. BMC geriatrics. 2024;24(1):169. doi: 10.1186/s12877-024-04748-1  302. Jiao K. Genomics and Machine Learning-based Exploration of Mitochondrial Metabolism-related Markers Expression after Spinal Cord Injury. Master's Degree, Naval Medical University of the People's Liberation Army of China. 2024. Available from:https://d.wanfangdata.com.cn/thesis/ChhUaGVzaXNOZXdTMjAyNDA5MjAxNTE3MjUSCFk0MzM5MjY2GghscW1xaXRrcA%3D%3D  303. Jimenez EL, Alvarez MN, Colino RM, Lopez MC, Jimenez CG, Rodriguez PP, et al. Muscle mass loss measured with portable ultrasound in hospitalized older adults: The ECOSARC study. Journal of Nutrition Health & Aging. 2024;28(1). doi: 10.1016/j.jnha.2023.100010 PMID:WOS:001185251200001  304. Jin X, Shi Z, Huang X, Zhou Y, Li L, editors. Application of Multi-component Exercise Combined with Cognitive Training Program in Elderly Patients with Psychiatric Disorders and Sarcopenia. The 6th Shanghai International Nursing Conference; 2024; Shanghai, China.  305. Ju Y, Lin X, Zhang K, Yang D, Cao M, Jin H, et al. The role of comprehensive geriatric assessment in the identification of different nutritional status in geriatric patients: a real-world, cross-sectional study. Frontiers in Nutrition. 2024;10. doi: 10.3389/fnut.2023.1166361 PMID:WOS:001148477800001  306. Kala S, Aggarwal A, Singh Rajput B, Kala C, Barman SK. Safety and Efficacy of Autologous Bone Marrow Derived Mononuclear Cell Transplant in the Management of Various Neurological Disorders. Cureus. 2024;16(12):e75617. doi: 10.7759/cureus.75617 PMID:39803099  307. Kamper RS, Nygaard H, Ekmann A, Schultz M, Hansen SK, Hansen P, et al. Feasibility of Assessing Older Patients in the Acute Setting: Findings From the Copenhagen PROTECT Study. Journal of the American Medical Directors Association. 2023;24(12):1898-903. doi: 10.1016/j.jamda.2023.07.002 PMID:WOS:001125655700001  308. Kanat BB, Suzan V, Avci GU, Unal D, Gedik TE, Erdincler DS, et al. Systemic inflammatory response index and monocyte-to-high density lipoprotein ratio- new biomarkers remarking the inflammation in primary sarcopenia: The SIMPS study. Bratislava Medical Journal-Bratislavske Lekarske Listy. 2024;125(5):331-6. doi: 10.4149/bll_2024_49 PMID:WOS:001235873600007  309. Kang M-g, Jung H-W, Kim B-J. A link between systemic low-grade inflammation and frailty in older adults: clinical evidence from a nationwide population-based study. Korean Journal of Internal Medicine. 2024;39(6). doi: 10.3904/kjim.2024.050 PMID:WOS:001274863300001  310. Kang MC, Deutz NEP, Kirschner SK, Engelen M. Metabolic kinetics and muscle and brain health markers in older adults, and the role of age and presence of chronic morbidities: A large cross-sectional cohort study. Clin Nutr. 2024;43(12):36-47. doi: 10.1016/j.clnu.2024.10.015 PMID:39423760  311. Kantilafti M, Hadjikou A, Chrysostomou S. The association between malnutrition, depression and cognitive decline in free-living elderly people in Cyprus: a cross-sectional study. BMC public health. 2024;24(1):3556. doi: 10.1186/s12889-024-21132-1  312. Kapan A, Ristic M, Leser A, Felsinger R, Waldhoer T. Assessment of muscle fatigability using isometric repetitive handgrip strength in frail older adults. A cross-sectional study. Journal of Translational Medicine. 2025;23(1). doi: 10.1186/s12967-025-06239-2  313. Kato M, Ono S, Seko H, Kito K, Omote T, Omote M, et al. Relationship between cachexia and short physical performance battery scores in patients with heart failure attending comprehensive outpatient cardiac rehabilitation. Heart and Vessels. 2024;39(9):778-84. doi: 10.1007/s00380-024-02400-x  314. Kawaguchi Y, Watanabe A, Shiratori T, Kaku R, Ueda K, Okamoto K, et al. Myostatin expression in lung cancer induces sarcopenia and promotes cancer progression. General Thoracic and Cardiovascular Surgery. 2024;72(4):232-9. doi: 10.1007/s11748-023-01969-w  315. Kawamura J, Tanaka T, Kanno S, Osawa K, Okabayashi K, Hirano H, et al. Relationship between a gum-chewing routine and oral, physical, and cognitive functions of community-dwelling older adults: A Kashiwa cohort study. Geriatrics and Gerontology International. 2024;24(1):68-74. doi: 10.1111/ggi.14757  316. Khedr EM, Karamallah G, El-Mokhtar M, Mahmoud DM, El-Deen HB. 108. Serum Biomarkers and Their Association with Clinical, Cognitive, and Quality of Life Parameters in Disease-Modifying Therapy-Naive Multiple Sclerosis Patients. 2024.  317. Kim HJ, Kim HY. Nomogram for predicting changes in cognitive function in community dwelling older adults with mild cognitive impairment based on Korea Longitudinal Study of Ageing Panel Data: a retrospective study. Journal of Korean Academy of Nursing. 2025;55(1):50-63. doi: 10.4040/jkan.24059  318. Kim HR, Kim MJ, Jeon JW, Ham YR, Na KR, Park H, et al. Association between Serum GDF-15 and Cognitive Dysfunction in Hemodialysis Patients. Biomedicines. 2024;12(2). doi: 10.3390/biomedicines12020358 PMID:WOS:001174957300001  319. Kim SM, Choi S, Lee G, Oh YH, Son JS, Ko A, et al. Association of changes in predicted body composition with subsequent risk of dementia. Annals of Clinical and Translational Neurology. 2024;11(8):1952-63. doi: 10.1002/acn3.52096  320. Kirk B, Harrison S, Zanker J, Burghardt AJ, Orwoll ES, Cawthon PM, et al. Interactions between bone and muscle quality on mortality risk in 1,353 men (aged 77-101) over 6 years: A prospective cohort study utilizing highresolution bone imaging and stable muscle isotopes. Journal of Bone and Mineral Research. 2024;39:217-8. doi:  321. Kirolos A, Harawa PP, Chimowa T, Divala O, Freyne B, Jones AG, et al. Long-term outcomes after severe childhood malnutrition in adolescents in Malawi (LOSCM): a prospective observational cohort study. The lancet Child & adolescent health. 2024;8(4):280‐9. doi: 10.1016/S2352-4642(23)00339-5 PMID:CN-02671326  322. Kocyigit SE, Bulut EA, Aydin AE, Dost FS, Kaya D, Isik AT. The relationship between cognitive frailty, physical frailty and malnutrition in Turkish older adults. Nutrition. 2024;126. doi: 10.1016/j.nut.2024.112504 PMID:WOS:001296171200001  323. Koevska V, Nikolikj-Dimitrova E, Mitrevska B, Gjeracaroska-Savevska C, Gocevska M, Kalcovska B. Effect of Exercises on Quality of Life in Patients with Postmenopausal Osteoporosis - Randomized Trial. Open access Macedonian journal of medical sciences. 2019;7(7):1160‐5. doi: 10.3889/oamjms.2019.271 PMID:CN-02646192  324. Koga HK, Grodstein F, Williams DR, Manson JE, Tindle HA, Shadyab AH, et al. Longitudinal Associations between Optimism and Objective Measures of Physical Functioning in Women. JAMA Psychiatry. 2024;81(5):489-97. doi: 10.1001/jamapsychiatry.2023.5068  325. Komalasari DR, Jalayondeja C, Jalayondeja W, Romadhon YA. Predictors of Quality of Life among Older Residents in Rural and Urban Areas in Indonesia: An Approach Using the International Classification of Functioning, Disability, and Health. Journal of preventive medicine and public health = Yebang Uihakhoe chi. 2024. doi: 10.3961/jpmph.24.423  326. Komalasari R, Mpofu E, Chen Rita Chang H, Talluntondok EB, Uligraff DK, Zhan R, et al. Higher Dynamic Balance Performance Was Associated With Cognitive Function Among U.S. Community-Dwelling Low-Income Older Adults. SAGE Open Nurs. 2024;10:23779608241296629. doi: 10.1177/23779608241296629 PMID:39539986  327. Komorita Y, Ide H, Yoshinari M, Ohta Y, Nakamichi I, Fujisawa R, et al. Decreased serum creatinine to cystatin C ratio is associated with low tongue pressure. Geriatrics and Gerontology International. 2024;24(1):102-8. doi: 10.1111/ggi.14780  328. Konvalinkova R, Srp M, Doleckova K, Capek V, Gal O, Hoskovcova M, et al. The impact of expiratory muscle strength training on voluntary cough effectiveness in Huntington's disease. Eur J Neurol. 2024;31(12):e16500. doi: 10.1111/ene.16500 PMID:39344651  329. Koponen S, Nykänen I, Savela RM, Välimäki T, Suominen AL, Schwab U. Family caregivers’ better nutritional status is associated with care recipients’ better nutritional status. Clinical Nutrition ESPEN. 2024;62:199-205. doi: 10.1016/j.clnesp.2024.05.016  330. Kratz AL, Ehde DM, Alschuler KN, Pickup K, Ginell K, Fritz NE. Optimizing Detection and Prediction of Cognitive Function in Multiple Sclerosis With Ambulatory Cognitive Tests: Protocol for the Longitudinal Observational CogDetect-MS Study. JMIR Res Protoc. 2024;13:e59876. doi: 10.2196/59876 PMID:39325510  331. Krell-Roesch J, Syrjanen JA, Moeller T, Krafft J, Barisch-Fritz B, Kremers WK, et al. Self-reported physical activity and gait in older adults without dementia: A longitudinal study. Health Science Reports. 2024;7(11). doi: 10.1002/hsr2.70108  332. Kuparasundram S, Ng T, Lim ZH, Samuel G, Ng YS. Associations between physical performance tests and affect in Asian community-dwelling older adults: a Cross-Sectional Analysis. Archives of Physical Medicine and Rehabilitation. 2024;105(4):e87. doi: 10.1016/j.apmr.2024.02.244  333. Laksmi PW, Purnamasari D, Sofian N, Sari NK, Kurniawan M, Sukrisman L, et al. Physio-cognitive decline syndrome among middle-aged diabetes patients: Handgrip strength significantly correlates with glycaemic control and cognitive score. Heliyon. 2024;10(2):e24018. doi: 10.1016/j.heliyon.2024.e24018 PMID:38293379  334. Lam K, Kleijwegt H, Bollens-Lund E, Nicholas LH, Covinsky KE, Ankuda CK. Long-term outcomes after rehabilitation in Medicare Advantage and fee-for-service beneficiaries. Journal of the American Geriatrics Society. 2024;72(6):1697-706. doi: 10.1111/jgs.18917  335. Lange U, Morena N, Ladner-Merz S. Cognitive training and physical therapy for fibromyalgia : results of the KogTraP pilot study. Zeitschrift fur Rheumatologie. 2024;83(9):721‐30. doi: 10.1007/s00393-024-01566-z PMID:CN-02759043  336. Lanzani CL, Simonini M, Citterio L, Zagato L, Brioni E, Damanti S, et al. Ageing, renal function and frailty: analysis of the FRASNET study. Nephrology Dialysis Transplantation. 2024;39:i1022-3. doi: 10.1093/ndt/gfae069.623  337. Larun L, Brurberg KG, Odgaard-Jensen J, Price JR. Exercise therapy for chronic fatigue syndrome. Cochrane Database of Systematic Reviews. 2024;(12). doi: 10.1002/14651858.CD003200.pub9 PMID:CD003200  338. Lee J. Handgrip strength and cognitive function among older adults with COPD. European Respiratory Journal. 2024;64:PA2282. doi: 10.1183/13993003.congress-2024.PA2282  339. Lee L, Jones A, Patel T, Hillier LM, Heckman GA, Costa AP. Frailty prevalence and efficient screening in primary care-based memory clinics. Fam Pract. 2023;40(5-6):689-97. doi: 10.1093/fampra/cmad035 PMID:37002941  340. Lefter N, Abdulan IM, Maștaleru A, Leon MM, Rusu C. Physical Activity and Cognitive Impairment in a Group of Adults with Down Syndrome from North-Eastern Romania. Journal of Clinical Medicine. 2024;13(19). doi: 10.3390/jcm13195829  341. Legdeur N, Badissi M, Venkatraghavan V, Woodworth DC, Orlhac F, Vidal JS, et al. The Temporal Relation of Physical Function with Cognition and the Influence of Brain Health in the Oldest-Old. Gerontology. 2025;71(1):13-27. doi: 10.1159/000542395 PMID:39504937  342. Lei L, Zhou Y, Ye L, Yang Y. Contribution of social activity participation to the relationship between sensory impairment, physical performance and cognitive decline: a longitudinal study in China. Frontiers in Aging Neuroscience. 2024;16. doi: 10.3389/fnagi.2024.1498354  343. Leroy V, Ayers E, Adhikari D, Verghese J. Association of Sleep Disturbances With Prevalent and Incident Motoric Cognitive Risk Syndrome in Community-Residing Older Adults. Neurology. 2024;103(11). doi: 10.1212/WNL.0000000000210054  344. Li J, Gao Y, Li X, Yu Y, Li G, Yuan H. Prevalence, associated factors and clinical implications of subjective cognitive decline linked to frailty in patients receiving maintenance hemodialysis: a cross-sectional study. BMC Nephrology. 2025;26(1). doi: 10.1186/s12882-025-04020-7  345. Li J, Wang Y, Zhai M, Qin M, Zhao D, Xiang Q, et al. Risk factors and a nomogram for predicting cognitive frailty in Chinese patients with lung cancer receiving drug therapy: A single-center cross-sectional study. Thoracic Cancer. 2024;15(11):884-94. doi: 10.1111/1759-7714.15256 PMID:WOS:001179941100001  346. Li M. Research on the Relationship between Sarcopenia and Self-Management Behaviors in Elderly Patients with Type 2 Diabetes. Master's Degree, Peking Union Medical College. 2024. Available from:https://link.cnki.net/doi/10.27648/d.cnki.gzxhu.2024.000703  347. Li N, Zhang J, Du Y, Li J, Wang A, Zhao X. Gait speed after mild stroke/transient ischemic attack was associated with long-term adverse outcomes: A cohort study. Annals of Clinical and Translational Neurology. 2024;11(12):3163-74. doi: 10.1002/acn3.52222  348. Li R, Chen X, Tang H, Luo S, Lian R, Zhang W, et al. Sarcopenic obesity and falls in older adults: A validation study of ESPEN/EASO criteria and modifications in Western China communities. Archives of Gerontology and Geriatrics. 2024;127. doi: 10.1016/j.archger.2024.105557  349. Li S. Observational study on the effect of blood glucose control on neurovascular coupling function in middle-aged and elderly patients with type 2 diabetes mellitus. Master's Degree, Hainan Medical University. 2024. Available from:https://d.wanfangdata.com.cn/thesis/ChhUaGVzaXNOZXdTMjAyNDA5MjAxNTE3MjUSCUQwMzU3NTM0MhoIbHFtcWl0a3A%3D  350. Li S, Wang P, Cai Z, Jiang W, Xin X, Wang X, et al. Correlates of physical activity levels, muscle strength, working memory, and cognitive function in older adults. Frontiers in Aging Neuroscience. 2023;15. doi: 10.3389/fnagi.2023.1283864 PMID:WOS:001133143900001  351. Li S, Yan H, Pan Y, Zhang Y. Association of the sarcopenia index with cognitive impairment in a middle-aged to older patients with acute ischemic stroke or transient ischemic attack: A multicenter cohort study. Journal of Nutrition, Health and Aging. 2024;28(7). doi: 10.1016/j.jnha.2024.100241  352. Li X. Causal relationships of vitamin D with disability in activities of daily living and mortality among Chinese oldest old: a cohort and one sample Mendelian randomization study. Doctoral Degree, Jilin University. 2024. Available from:https://d.wanfangdata.com.cn/thesis/ChhUaGVzaXNOZXdTMjAyNDA5MjAxNTE3MjUSCUQwMzUwMTk4NBoIbHFtcWl0a3A%3D  353. Li X. Involvement of insulin-degrading enzyme in the pathogenesis of Parkinson's disease associated with CHCHD2 gene mutation. Master's Degree, Zhengzhou University. 2024. Available from:https://d.wanfangdata.com.cn/thesis/ChhUaGVzaXNOZXdTMjAyNDA5MjAxNTE3MjUSCFk0MzQ4MDczGghscW1xaXRrcA%3D%3D  354. Li Y. Clinical analysis of 13 children with febrile infection-related epilepsy syndrome. Master's Degree, Zhengzhou University. 2024. Available from:https://d.wanfangdata.com.cn/thesis/ChhUaGVzaXNOZXdTMjAyNDA5MjAxNTE3MjUSCFk0MzQ4MjI4GghscW1xaXRrcA%3D%3D  355. Li Y, Guo M, Fei Y, Liu Y, Al-Ghammari A, Chen S, et al. Association between oral health and physio-cognitive decline syndrome of older adults in China and its sex differences: a cross-sectional study. BMC geriatrics. 2025;25(1):137. doi: 10.1186/s12877-025-05801-3  356. Li Y, Zhu L, Zhang C, Zhao H, Wang W, Guo L, et al. The Grip Strength Loss Rate and the Subsequent Cognitive Decline Rate in Older Adults: The Moderating Role of Social Isolation. Innov Aging. 2024;8(8):igae055. doi: 10.1093/geroni/igae055 PMID:39144546  357. Liao J, Wang J, Jia S, Cai Z, Liu H. Correlation of muscle strength, working memory, and activities of daily living in older adults. Frontiers in Aging Neuroscience. 2024;16. doi: 10.3389/fnagi.2024.1453527 PMID:WOS:001325877000001  358. Lieber RL. Pre-clinical study for the use of D3-Creatine as a biomarker in cachexia clinical trials. 2024. doi: PMID:GRANTS:17702088  359. Liegl G, Fischer F, Canaud B, Woodward M, Barth C, Davenport A, et al. Comparing patient-reported and performance-based physical function assessments in hemodialysis: insights from the CONVINCE trial. Nephrology dialysis transplantation. 2024;39:i2487. doi: 10.1093/ndt/gfae069.1560 PMID:CN-02726335  360. Lim ST, Kwak HB, Kang JH, Chang E, Joa KL, Park HJ, et al. Effects of physical activity participation on cognitive impairment in older adults population with disabilities. Frontiers in public health. 2024;12:1293023. doi: 10.3389/fpubh.2024.1293023  361. Lim ZH, Yu J, Kuparasundram S, Mahendran R, Ng TKS. Associations between Physical Performance Tests with Cognitive Changes: The Moderating Effect of Cognitive Status. Curr Alzheimer Res. 2024;21(6):423-36. doi: 10.2174/0115672050342857241025091918 PMID:39572921  362. Lin CL, Wu HC, Yu NC, Liu YC, Chiu IY, Chien WC. Risk factors for falls in older adults with type 2 diabetes: A cross-sectional study. Medicine (United States). 2024;103(50):e40895. doi: 10.1097/MD.0000000000040895  363. Lin W-L, Hsueh T-P, Wang Y-C, Chiu J-T, Yan S-S, Wang Y-F, et al. Implications of comprehensive geriatric assessment and Traditional Chinese Medicine constitution types for integrative geriatric care. Archives of Gerontology and Geriatrics. 2025;129. doi: 10.1016/j.archger.2024.105697 PMID:WOS:001368987900001  364. Lin WL, Chen JJ, Wu LM, Huang WT, Guo HR, Nguyen THY. A sarcopenia screening test predicts mortality among hospitalized cancer patients. Physiological Reports. 2024;12(15). doi: 10.14814/phy2.16173  365. Lin Y-C, Chen Z-J, Tung H-H, Ye Y-J, Lai H-Y, Hsiao F-Y, et al. Association between possible sarcopenia and domain-specific cognitive impairment in middle-aged and older adults: Insights from the Gan-Dau Healthy Longevity Plan. Experimental Gerontology. 2024;194. doi: 10.1016/j.exger.2024.112487 PMID:WOS:001262818100001  366. Lindholm B, Basna R, Ekström H, Elmståhl S, Siennicki-Lantz A. Gait Speed Reserve in the general population-based 'Good Aging in Skåne' cohort study-distribution and associated factors. Geroscience. 2025;47(1):965-76. doi: 10.1007/s11357-024-01318-6 PMID:39192005  367. Liu H, Li W, Zhu M, Wen X, Jin J, Wang H, et al. Myokines and Biomarkers of Frailty in Older Inpatients with Undernutrition: A Prospective Study. Journal of Frailty and Aging. 2024;13(2):82-90. doi: 10.14283/jfa.2024.9  368. Liu X, Jiang T, Jiang Y, Li L, Cao Y. Prevalence of mild cognitive impairment and modifiable risk factors: A cross-sectional study in rural older adults with diabetes. Geriatric nursing (New York, NY). 2024;59:549-56. doi: 10.1016/j.gerinurse.2024.08.010  369. Liu X, Ni J, Wang B, Yin R, Tang J, Chu Q, et al. A prediction model for the risk of developing mild cognitive impairment in older adults with sarcopenia: evidence from the CHARLS. Aging Clin Exp Res. 2025;37(1):69. doi: 10.1007/s40520-025-02980-2 PMID:40055290  370. Liu Y. Nutrition bears heavy responsibility in elderly health, and the silver age's rosy future is already emerging. 2025-01-03.  371. Liu Y. Effects of threshold inspiratory muscle training combined with continuous positive airway pressure on sleep and cognitive function in stroke patients with obstructive sleep apneahypopnea syndrome. Master's Degree, Zhengzhou University. 2024. Available from:https://d.wanfangdata.com.cn/thesis/ChhUaGVzaXNOZXdTMjAyNDA5MjAxNTE3MjUSCFk0MzQ2NzE4GghscW1xaXRrcA%3D%3D  372. Liu Y, Zhang L, Yang S, Liu R, Yi L, Liu M, et al. Predictive role of gait parameters and MRI markers in assessing cognitive decline in CSVD patients. BMC geriatrics. 2025;25(1):116. doi: 10.1186/s12877-025-05738-7  373. Liu YH, Ma LL, Hu LK, Cui L, Li YL, Chen N, et al. The joint effects of sarcopenia and cardiometabolic risk factors on declined cognitive function: Evidence from a 7-year cohort study. Journal of Affective Disorders. 2024;344:644-52. doi: 10.1016/j.jad.2023.10.056  374. Lo AX, Wadley VG, Brown CJ, Long DL, Crowe M, Howard VJ, et al. Life-Space Mobility: Normative Values From a National Cohort of U.S. Older Adults. The journals of gerontology Series A, Biological sciences and medical sciences. 2024;79(2). doi: 10.1093/gerona/glad176  375. Long L, Xiong B, Luo Z, Yang H, She Q. Association between Pan-Immune Inflammation Value and Sarcopenia in Hypertensive Patients, NHANES 1999-2018. J Clin Hypertens (Greenwich). 2025;27(1):e14944. doi: 10.1111/jch.14944 PMID:39552179  376. López Jiménez E, Neira Álvarez M, Menéndez Colino R, Checa López M, Grau Jiménez C, Pérez Rodríguez P, et al. Muscle mass loss measured with portable ultrasound in hospitalized older adults: The ECOSARC study. Journal of Nutrition, Health and Aging. 2024;28(1). doi: 10.1016/j.jnha.2023.100010  377. López-Daza D, Posada-Alvarez C, Guzmán-Silva MP, Agudelo-Martinez A, Merchan-Chaverra R, Cuellar-Fernandez Y, et al. Why wait for nutritional monitoring? results in patients with neurological disease with monthly evaluations. 2024. p. 1243.  378. Low S, Ng TP, Goh KS, Moh A, Khoo J, Ang K, et al. Reduced skeletal muscle mass to visceral fat area ratio is independently associated with reduced cognitive function in type 2 diabetes mellitus. Journal of Diabetes and its Complications. 2024;38(2). doi: 10.1016/j.jdiacomp.2023.108672  379. Lozada-Martinez ID, Vindas-Meza L, Castelblanco-Toro S, Salazar-Uribe JC, Anaya JM. The impact of nutritional status on centenarians’ physical, mental, and functional health. Clinical Nutrition Open Science. 2025;60:10-20. doi: 10.1016/j.nutos.2025.01.010  380. Lu JK, Wang W, Soh J, Sandalova E, Lim ZM, Seetharaman SK, et al. Characterizing biomarkers of ageing in Singaporeans: the ABIOS observational study protocol. GeroScience. 2025. doi: 10.1007/s11357-025-01511-1  381. Luan T. Lipid levels and neutrophil/lymphocyte ratio in predicting the risk of thromboembism in patients with nonvalvular atrial fibrillation. Master's Degree, Jilin University. 2024. Available from:https://d.wanfangdata.com.cn/thesis/ChhUaGVzaXNOZXdTMjAyNDA5MjAxNTE3MjUSCUQwMzUwNzAxNRoIbHFtcWl0a3A%3D  382. Lundberg K, Elmståhl S, Wranker LS, Ekström H. The Association between Physical Frailty and Cognitive Performance in Older Adults Aged 60 to 96 Years: Data from the “Good Aging in Skåne” (GÅS) Swedish Population Study. Annals of Geriatric Medicine and Research. 2024;28(3):330-41. doi: 10.4235/agmr.24.0055  383. Lunt EK, Gordon AL, Greenhaff PL, Gladman JFR. The influence of immobility on muscle loss in older people with frailty and fragility fractures. GeroScience. 2024;46(6):5473-84. doi: 10.1007/s11357-024-01177-1  384. Luo J. Study on properties of recycled pulp andphoto functional materials of waste cotton textiles. Master's Degree, Qilu University of Technology. 2024. Available from:https://d.wanfangdata.com.cn/thesis/ChhUaGVzaXNOZXdTMjAyNDA5MjAxNTE3MjUSCFk0MzI2MzkxGghscW1xaXRrcA%3D%3D  385. Lv T, Dong Y, Zhang H, Li Y. Knowledge, attitude, and practice regarding Sarcopenia in maintenance hemodialysis patients in Northeastern China. Scientific Reports. 2024;14(1). doi: 10.1038/s41598-024-75395-0 PMID:WOS:001339991400013  386. Ma Y, Wu X, Zhao Y, Hong W, Luan Y, Song P, et al. Relationships between muscle strength, lung function, and cognitive function in Chinese middle-aged and older adults: A study based on the China health and retirement longitudinal study (CHARLS). Journal of the Formosan Medical Association. 2025;124(2):171-7. doi: 10.1016/j.jfma.2024.04.001  387. Maeda D, Fujimoto Y, Nakade T, Abe T, Ishihara S, Jujo K, et al. Frailty, Sarcopenia, Cachexia, and Malnutrition in Heart Failure. Korean Circulation Journal. 2024;54(7):363-81. doi: 10.4070/kcj.2024.0089 PMID:WOS:001267846100001  388. Magalhães VRT. Impacto de um programa do exercício físico comunitário para idosos na progressão da sarcopenia: 2 anos de follow-upImpact of a Community Physical Exercise Program for the Elderly on the Progression of Sarcopenia: 2 years of Follow-Up2024.  389. Makizako H, Akaida S, Tateishi M, Shiratsuchi D, Kiyama R, Kubozono T, et al. A Three-Year Longitudinal Follow-Up Study: Does Mild Cognitive Impairment Accelerate Age-Related Changes in Physical Function and Body Composition? Cureus. 2024;16(9):e68605. doi: 10.7759/cureus.68605 PMID:39371775  390. Mammadzada N, Tasci I. Sedentary behavior and associated factors on admissions to internal medicine wards. Internal and emergency medicine. 2024;19(8):2203-11. doi: 10.1007/s11739-024-03737-x  391. Maniscalco L, Veronese N, Ragusa FS, Vernuccio L, Dominguez LJ, Smith L, et al. Sarcopenia using muscle mass prediction model and cognitive impairment: A longitudinal analysis from the English longitudinal study on ageing. Archives of Gerontology and Geriatrics. 2024;117. doi: 10.1016/j.archger.2023.105160  392. Marcu FM, Ciobanu D, Boca IC, Sirbu E, Deme PA, Hreniuc NC, et al. Rehabilitation therapy versus drug-only therapy in patients with multiple sclerosis. Turk J Med Sci. 2024;54(1):157-64. doi: 10.55730/1300-0144.5776 PMID:38812628  393. Matsuno K, Asaoka D, Sugano K, Takahashi K, Miyauchi K. Rationale and design of Juntendo Sarcopenia Registration to explore the predictors and prognosis of sarcopenia and frailty in the elderly in TOKYO (JUSTICE-TOKYO). Geriatrics and Gerontology International. 2024;24(1):168-72. doi: 10.1111/ggi.14779  394. Matsushita Y, Watanabe Y, Shirahase R, Yamazaki Y. Relationship between Body Mass Index and Sarcopenia with Oral Function Decline in Older Japanese Patients Who Regularly Attend a General Dental Clinic. Journal of Frailty and Aging. 2024;13(1):21-30. doi: 10.14283/jfa.2024.5  395. Mbabazi P, Banturaki G, Naikoba S, Nasuuna EM, Manabe YC, Greene M, et al. Sex Differences in the Prevalence of Geriatric Syndromes Among Older People Living with HIV Attending an Urban Outpatient Clinic in Kampala, Uganda. HIV/AIDS - Research and Palliative Care. 2024;16:455-65. doi: 10.2147/HIV.S489598  396. Mc Ardle R, Taylor L, Cavadino A, Rochester L, Del Din S, Kerse N. Characterizing Walking Behaviors in Aged Residential Care Using Accelerometry, With Comparison Across Care Levels, Cognitive Status, and Physical Function: cross-Sectional Study. JMIR aging. 2024;7:e53020. doi: 10.2196/53020 PMID:CN-02706887  397. McCourt O, Cairns DA, Moore S, Parrish C, Pawlyn C, Seymour F, et al. Health-Related Quality of Life (HRQoL) in Fit, Unfit and Frail Patients Enrolled in Fitness (UK-MRA Myeloma XIV): a Cross Sectional Study. Blood. 2023;142:3368. doi: 10.1182/blood-2023-187202 PMID:CN-02664197  398. McGowan T, Danielson J, Gehrmann F, Hilton N, Lunn E, McLennan K, et al. Outcomes after childhood stroke in an inpatient paediatric rehabilitation unit: A retrospective study. J Paediatr Child Health. 2024;60(11):691-7. doi: 10.1111/jpc.16658 PMID:39243228  399. Mei CTY, Ying SSS, Yanshan DL, Van Koh S, Karthikeyan G, Jiawen OX, et al. Prevalence and factors associated with sarcopenia among older adults in a postacute hospital in Singapore. PLoS ONE. 2024;19(1 January). doi: 10.1371/journal.pone.0291702  400. Mejia AC, Sapienza S, Conde PM, Pavelka L, Krueger R, Klucken J. Modeling self-reported mobility in Parkinson's Disease through sensor-derived gait parameters. 2024. p. 41-2.  401. Mendez-Guerrero O, Carrasco AC, Navarro-Alvarez N. THE IMPACT OF ZINC DEFICIENCY AND NUTRITIONAL STATUS ON THE DEVELOPMENT OF OVERT HEPATIC ENCEPHALOPATHY IN CIRRHOSIS PATIENTS: A PROSPECTIVE COHORT STUDY. Hepatology. 2024;80:S1726-S7. doi: 10.1097/HEP.0000000000001077  402. Mengist B, Lotfaliany M, Pasco JA, Agustini B, Berk M, Williams LJ, et al. Gait speed, handgrip strength, and their combination, and risk of depression in later life: Evidence from a prospective study of community-dwelling older adults. Journal of Affective Disorders. 2025;369:218-26. doi: 10.1016/j.jad.2024.09.155  403. Merchant RA, Chan YH, Anbarasan D, Vellas B. Association of intrinsic capacity with functional ability, sarcopenia and systemic inflammation in pre-frail older adults. Frontiers in Medicine. 2024;11. doi: 10.3389/fmed.2024.1374197  404. Mielenz TJ, Jia H, DiGuiseppi CG, Strogatz D, Andrews HF, Molnar LJ, et al. Frailty and poor physical functioning as risk factors for driving cessation. Front Public Health. 2024;12:1298539. doi: 10.3389/fpubh.2024.1298539 PMID:38765490  405. Milte CM, Lamb KE, McNaughton SA. Cross-sectional associations between fruit and vegetable intake and successful ageing across six countries: findings from the WHO Study on global AGEing and adult health (SAGE). Public Health Nutrition. 2024;27(1). doi: 10.1017/s1368980024001976 PMID:WOS:001366196300001  406. Ming A, Clemens V, Lorek E, Wall J, Alhajjar A, Galazky I, et al. Game-Based Assessment of Peripheral Neuropathy Combining Sensor-Equipped Insoles, Video Games, and AI: Proof-of-Concept Study. Journal of Medical Internet Research. 2024;26. doi: 10.2196/52323  407. Ming A, Schubert T, Marr V, Hötzsch J, Stober S, Mertens PR. Video game-based application for fall risk assessment: a proof-of-concept cohort study. eClinicalMedicine. 2024;78. doi: 10.1016/j.eclinm.2024.102947  408. Miura K, Matsushita T, Nishioka S, Nakashima R, Onizuka S. Association between sarcopenia at discharge and functional outcomes 1 month and 6 months after discharge in patients in convalescent rehabilitation wards. Geriatrics & Gerontology International. 2024;24(7):715-21. doi: 10.1111/ggi.14921 PMID:WOS:001242951300001  409. Mogahed HG, Hamoda RE, Elkalla RA. Virtual reality on pain and anxiety after modified radical mastectomy in menopause. Research journal of pharmacy and technology. 2024;17(4):1657‐61. doi: 10.52711/0974-360X.2024.00262 PMID:CN-02747932  410. Monterrosa-Castro A, Castilla-Casalins A, Blanco-Teherán C. Association between psychophysical and cognitive aspects with sleep complaints in postmenopausal Colombian women. Sleep Medicine. 2024;115:87. doi: 10.1016/j.sleep.2023.11.268  411. Moon S, Oh E, Chung D, Hong GRS. Changes in instrumental activities daily living limitations and their associated factors according to gender in community-residing older adults: A longitudinal cohort study. PLoS ONE. 2024;19(1 January). doi: 10.1371/journal.pone.0296796  412. Morel E, Lingenberg A, Armand S, Assal F, Allali G. Normal pressure hydrocephalus and cognitive impairment: The gait phenotype matters too. Eur J Neurol. 2024;31(8):e16328. doi: 10.1111/ene.16328 PMID:38720477  413. Moreno-Gonzalez R, Cruzado JM, Corsonello A, Fabbietti P, Tap L, Mattace-Raso F, et al. Kidney function and other associated factors of sarcopenia in community-dwelling older adults: The SCOPE study. European Journal of Internal Medicine. 2024;123:81-93. doi: 10.1016/j.ejim.2023.12.002 PMID:WOS:001240592500001  414. Mustafa A, Singh A. Overlooked Burden of Undernutrition Among Older Adults in India. J Nutr Gerontol Geriatr. 2024;43(2):116-33. doi: 10.1080/21551197.2024.2358759 PMID:38819374  415. Naaktgeboren WR, Koevoets EW, Stuiver MM, van Harten WH, Aaronson NK, van der Wall E, et al. Effects of physical exercise during adjuvant chemotherapy for breast cancer on long-term tested and perceived cognition: results of a pragmatic follow-up study. Breast cancer research and treatment. 2024;205(1):75‐86. doi: 10.1007/s10549-023-07220-7 PMID:CN-02658561  416. Naidoo S, Naidoo N. Physical activity and ageing: The role of physiotherapy in promoting healthy ageing. South African Journal of Physiotherapy. 2025;81(1):1-8. doi: 10.4102/sajp.v81i1.2114 PMID:SCIELO:S2410-82192025000100005  417. Naz S, Song J, Lee IJ, Khan MA, Khan A, Shafi MA. The Association of Nutritional Status with Mortality in Geriatric Patients With Cancer - A 4.5-Year Prospective Study Using Validated Screening Tools. American Journal of Gastroenterology. 2024;119(10):S1585-S6. doi: 10.14309/01.ajg.0001038244.34483.97  418. Nct. The Impact of 6-months of Resistance Training on Brain and Muscle Health in Older Adults With MCI. https://clinicaltrialsgov/ct2/show/NCT06252844. 2023. doi: PMID:CN-02680133  419. Nct. Impact of Nutrition, Sleep, and Physical Activity on Intellectual Function and Muscle Mass in Older Adults. https://clinicaltrialsgov/ct2/show/NCT06135740. 2023. doi: PMID:CN-02651717  420. Nct. The Impact of Exercise Intervention on Physical Function Falls, and Physical Restraint for Long-term Care Residents. https://clinicaltrialsgov/ct2/show/NCT06096467. 2023. doi: PMID:CN-02651472  421. Nct. Assessment of Body Composition, Fatigue, Mobility and Functional Status in Post-Stroke Individuals. https://clinicaltrialsgov/ct2/show/NCT06255145. 2023. doi: PMID:CN-02687584  422. Nct. Pre-operative Risk Assessment Combined With Targeted Intervention in the Chinese Elderly With Spine Surgery. https://clinicaltrialsgov/ct2/show/NCT06140797. 2023. doi: PMID:CN-02629345  423. Nct. Effects of Transcranial Electrical Stimulation in Stroke Individuals. https://clinicaltrialsgov/ct2/show/NCT06134921. 2023. doi: PMID:CN-02679647  424. Nct. Effect of Vinyasa Yoga on Frailty and Anthropometric Measurements in Elderly Individuals. https://clinicaltrialsgov/ct2/show/NCT06189430. 2023. doi: PMID:CN-02652091  425. Nct. Brain Imaging and Behavioural Changes Following Cued-movement Training of Finger Sequences in Healthy Older Adults. https://clinicaltrialsgov/ct2/show/NCT06174740. 2023. doi: PMID:CN-02635318  426. Nct. Modified Ketogenic Diet in Amnestic Mild Cognitive Impairments. https://clinicaltrialsgov/ct2/show/NCT06444568. 2024. doi: PMID:CN-02707503  427. Nct. Effects of Integrated Exercise on Sarcopenia, Depression Symptoms, and Quality of Life. https://clinicaltrialsgov/ct2/show/NCT06637228. 2024. doi: PMID:CN-02767264  428. Nct. Effects of Multimodal Music Intervention on Mild Cognitive Impairment Elderly. https://clinicaltrialsgov/ct2/show/NCT06324227. 2024. doi: PMID:CN-02681946  429. Nct. A Theory-based Home-based Multi-component Exercise Training Among Older Adults With Type 2 Diabetes Mellitus. https://clinicaltrialsgov/ct2/show/NCT06393244. 2024. doi: PMID:CN-02695364  430. Nct. Aging Resilience Through Microbiota Optimization and Regulation. https://clinicaltrialsgov/ct2/show/NCT06649981. 2024. doi: PMID:CN-02771138  431. Nct. E-Based Physical Exercise in Patients With Multiple Sclerosis and Comorbidity. https://clinicaltrialsgov/ct2/show/NCT06298201. 2024. doi: PMID:CN-02681266  432. Nct. Metabolic Investigation, Physical Performance, Physical Training At Different Times of the Day in Obese Women. https://clinicaltrialsgov/ct2/show/NCT06601660. 2024. doi: PMID:CN-02760796  433. Nct. CONSTELLATIONS Living Lab: improving the Care Transitions of Older Adults Living with Neurocognitive Disorders. https://clinicaltrialsgov/ct2/show/NCT06608589. 2024. doi: PMID:CN-02781976  434. Nct. The Effect of Dual-task Training on Balance, Exercise Capacity, Cognitive Status, and Quality of Life. https://clinicaltrialsgov/ct2/show/NCT06721429. 2024. doi: PMID:CN-02791084  435. Nct. PROSTATE-IQ: parallel RandOmized STudy of Personalized Apalutamide Treatment and Evaluation to Improve Quality of Life in Post-Operative Radiation With Androgen Axis Suppression. A Phase III Multi-center Study for Men With Detectable PSA After Prostatectomy for Prostate Cancer. https://clinicaltrialsgov/ct2/show/NCT06274047. 2024. doi: PMID:CN-02680636  436. Nct. Effects of Metformin on Androgens and Other Steroid Hormones in Affected Subjects With Autism. https://clinicaltrialsgov/ct2/show/NCT06762041. 2024. doi: PMID:CN-02800250  437. Nct. Effectiveness of Inspiratory Muscle Training for People with Ischemic Heart Disease Revascularized by Percutaneous Transluminal Coronary Angioplasty. https://clinicaltrialsgov/ct2/show/NCT06681740. 2024. doi: PMID:CN-02780103  438. Nct. Comparison of Two Non-invasive Neuromodulation Techniques as Synergistic Therapy to Cognitive Stimulation in Amnestic Mild Cognitive Impairment (aMCI). https://clinicaltrialsgov/ct2/show/NCT06467253. 2024. doi: PMID:CN-02720182  439. Nct. Efficacy of Cognitive Behavioral Therapy for Insomnia to Treat Insomnia Symptoms in Individuals With Multiple Sclerosis. https://clinicaltrialsgov/ct2/show/NCT06428006. 2024. doi: PMID:CN-02702399  440. Nct. Robotic vs. Traditional Verticalization in Patients With Severe Acquired Brain Injury: a Randomized Controlled Trial. https://clinicaltrialsgov/ct2/show/NCT06469983. 2024. doi: PMID:CN-02722792  441. Nct. Comparison of Krill and Fish Oil on Clinical and Biochemical Outcomes in Depression. https://clinicaltrialsgov/ct2/show/NCT06414226. 2024. doi: PMID:CN-02699990  442. Nct. Five Times Sit-To-Stand Test for Patients with Pediatric-Onset Multiple Sclerosis. https://clinicaltrialsgov/ct2/show/NCT06648499. 2024. doi: PMID:CN-02767708  443. Nct. Identification of Neuroinflammation and Neuroimaging Biomarkers Through Data Driven Artificial Intelligence Techniques for Unraveling the Heterogeneity of Aged Subjects at Risk of Dementia and to Better Inform Prevention Strategies. https://clinicaltrialsgov/ct2/show/NCT06746909. 2024. doi: PMID:CN-02794617  444. Nct. Effect of Two Years of Resistance Training on Health Status in Postmenopausal Women: longitudinal Study Active Aging. https://clinicaltrialsgov/ct2/show/NCT06621368. 2024. doi: PMID:CN-02766665  445. Nct. The Role of Precision Anesthesia Strategy on Perioperative Organ Protection in Hip Surgery. https://clinicaltrialsgov/ct2/show/NCT06696404. 2024. doi: PMID:CN-02790237  446. Nct. Impact of Screening and Multicomponent Exercise on Fall Rates, Fractures, and Cardiovascular Health in Diabetes. https://clinicaltrialsgov/ct2/show/NCT06745544. 2024. doi: PMID:CN-02794559  447. Nct. Cerebral and Anti-inflammatory Response Through Exercise - Mechanisms In Depressive Disorders. https://clinicaltrialsgov/ct2/show/NCT06450704. 2024. doi: PMID:CN-02709071  448. Nct. The Role of Executive Functioning in Complex Post-Traumatic Stress Disorder Among Female Survivors of Intimate Partner Violence. https://clinicaltrialsgov/ct2/show/NCT06706882. 2024. doi: PMID:CN-02790620  449. Nct. Black Seed Oil in ADHD. https://clinicaltrialsgov/ct2/show/NCT06542887. 2024. doi: PMID:CN-02741770  450. Nct. Effects of HRV Biofeedback, Interoceptive Training, and Mindfulness on Stress in University Students and Staff. https://clinicaltrialsgov/ct2/show/NCT06695715. 2024. doi: PMID:CN-02792798  451. Nct. Effect of Pully System on Hemiplegic Children. https://clinicaltrialsgov/ct2/show/NCT06434246. 2024. doi: PMID:CN-02705872  452. Nct. Effect of Qigong on Sleep Quality in Fibromyalgia. https://clinicaltrialsgov/ct2/show/NCT06347042. 2024. doi: PMID:CN-02685327  453. Nct. Mindfulness Meditation: alleviating Symptoms and Inflammation in Nurses. https://clinicaltrialsgov/ct2/show/NCT06635278. 2024. doi: PMID:CN-02767189  454. Nct. Japi: cognitive, Emotional and Social Stimulation for Preschool Children. https://clinicaltrialsgov/ct2/show/NCT06420544. 2024. doi: PMID:CN-02702191  455. Nct. To Drill or Not to Drill: do Memory Drills Help Train the Ability to "remember to Remember" in Veterans. https://clinicaltrialsgov/ct2/show/NCT06656637. 2024. doi: PMID:CN-02779159  456. Nct. Upper Limb Nerve Cryoneurolysis is Non Inferior to the Usual Care and Has Therapeutic Add Value in Dealing with Shoulder Pain and Functional Problems Caused by Spasticity and Motor Impairment. https://clinicaltrialsgov/ct2/show/NCT06782464. 2024. doi: PMID:CN-02802536  457. Nct. Moving on! A Tailored Treatment in a Primary Care Setting for Individuals With Anxiety Disorders? https://clinicaltrialsgov/ct2/show/NCT06715852. 2024. doi: PMID:CN-02790953  458. Nct. Evaluating the Practice Resource for Driving After Stroke. https://clinicaltrialsgov/ct2/show/NCT06303765. 2024. doi: PMID:CN-02687663  459. Nct. TrackFrailty Project. https://clinicaltrialsgov/ct2/show/NCT06812286. 2025. doi: PMID:CN-02809247  460. Nct. Neuroarchitectural Recovery Model of Post-stroke Patients. https://clinicaltrialsgov/ct2/show/NCT06825598. 2025. doi: PMID:CN-02811435  461. Nct. Reducing Disparities in Urinary Control Symptoms for Minority Women. https://clinicaltrialsgov/ct2/show/NCT06798311. 2025. doi: PMID:CN-02808724  462. Nicholson V, Steele M, Wilson P. Motor imagery does not effectively improve walking-related performance in older adults: a randomised controlled trial. Annals of physical and rehabilitation medicine. 2025;68(4). doi: 10.1016/j.rehab.2024.101899 PMID:CN-02801063  463. Nishida T, Fujikawa Y, Nagamune Y. The Impact of Lesser Trochanter Displacement on Hip Flexor Strength Recovery in Patients With Trochanteric Fracture. Cureus. 2024;16(11):e73095. doi: 10.7759/cureus.73095 PMID:39651004  464. Nishimoto K, Tsutsumimoto K, Nakakubo S, Kiuchi Y, Misu Y, Ohata T, et al. Association between physical, cognitive, and social activities with the incident of sarcopenia among community-dwelling older adults: a 4-year longitudinal study. European Geriatric Medicine. 2024;15(5):1331-8. doi: 10.1007/s41999-024-00985-0 PMID:WOS:001278331800003  465. Nishimura A, Masuda C, Murauchi C, Ishii M, Murata Y, Kawasaki T, et al. Relationship Between Frailty and Diabetic Pharmacologic Therapy in Older Adults with Type 2 Diabetes: A Cross-Sectional Study. Drugs and Aging. 2024;41(6):531-42. doi: 10.1007/s40266-024-01119-8  466. Nl O. A Randomised, Placebo Controlled, Ascending, Repeat Dose Study in Healthy Volunteers Investigating Safety, Tolerability, Pharmacokinetics and Pharmacodynamics of GSK356278. https://trialsearchwhoint/Trial2aspx?TrialID=NL-OMON35301. 2011. doi: PMID:CN-02713748  467. Nl O. Randomised, double blind, placebo controlled, multicentre study to evaluate the efficacy and safety of givinostat in ambulant patients with Duchenne Muscular Dystrophy EPIDYS (Epigenetic Rescue of Dystrophin Dysfunction). https://trialsearchwhoint/Trial2aspx?TrialID=NL-OMON50281. 2016. doi: PMID:CN-02719199  468. Nl O. Neuralgic amyotrophy: central reorganization and rehabilitation after peripheral dysfunction. https://trialsearchwhoint/Trial2aspx?TrialID=NL-OMON44371. 2018. doi: PMID:CN-02717619  469. Nl O. A Multiple Ascending Dose Study to Evaluate Safety, Tolerability, Pharmacokinetics, and Pharmacodynamics of ENX-101 at Plasma Steady State in Healthy Volunteers. https://trialsearchwhoint/Trial2aspx?TrialID=NL-OMON51175. 2021. doi: PMID:CN-02719581  470. Ohno K, Sawada S, Fujimaki N, Sakai K, Wakui S, Shibata N, et al. The Association Between Mild Cognitive Impairment and Physical Function in Older Japanese Adults Aged 75 Years or Older Living in Independent Senior Housing: A Cross-Sectional Study. Healthcare (Basel). 2024;12(21). doi: 10.3390/healthcare12212106 PMID:39517319  471. Oliva EN, Salutari P, Candoni A, Freyrie A, Capelli D, Di Raimondo F, et al. Quality of Life in Elderly Patients with Acute Myeloid Leukemia Undergoing Induction Chemotherapy. Blood. 2015;126(23):2120. doi: 10.1182/blood.V126.23.2120.2120 PMID:CN-02798811  472. Omana-Guzman I, Kammar-Garcia A, Gutierrez-Robledo LM, Rosas-Carrasco O. Undernutrition risk and obesity increase the risk of osteosarcopenia in Mexican adults aged 50 and over: a prospective cohort study. Frontiers in Nutrition. 2025;11. doi: 10.3389/fnut.2024.1499453 PMID:WOS:001410750300001  473. Oppermann J, Tschentscher V, Welzel J, Geritz J, Hansen C, Gold R, et al. Clinical and device-based predictors of improved experience of activities of daily living after a multidisciplinary inpatient treatment for people with Parkinson’s disease: a cohort study. Therapeutic Advances in Neurological Disorders. 2024;17. doi: 10.1177/17562864241277157  474. Orellana-Jaén J, Mora-Fernández M, Carrasco-Páez L. Effects of a motor and cognitive training program on executive function and different biomarkers related to muscle-brain crosstalk in breast cancer survivors: 3-arm randomised controlled BRAINonFIT study protocol. Contemporary clinical trials. 2024;146:107672. doi: 10.1016/j.cct.2024.107672 PMID:CN-02754031  475. Orsso CE, Vieira FT, Basuray N, Duke RL, Pakseresht M, Rubin DA, et al. The metabolic load-capacity model and cardiometabolic health in children and youth with obesity. Pediatric Obesity. 2024;19(3). doi: 10.1111/ijpo.13098  476. Özden F, Özkeskin M, Sarı Z, Ekici E, Yüceyar N. Association of Urinary Incontinence with Sensory-Motor Performance in Women with Multiple Sclerosis. Int Urogynecol J. 2024;35(12):2305-11. doi: 10.1007/s00192-024-05854-9 PMID:38976027  477. Pajunen H, Veitonmäki T, Huhtala H, Nikkola J, Pöyhönen A, Murtola T. Prognostic factors of renal cell cancer in elderly patients: a population-based cohort study. Scientific reports. 2024;14(1):6295. doi: 10.1038/s41598-024-56835-3  478. Pan Y, Li X, Zhang L, Li Y, Tang Z, Ma L. Declined intrinsic capacity predicts long-term mortality in Chinese older adults: Beijing Longitudinal Study of Aging. Maturitas. 2024;188. doi: 10.1016/j.maturitas.2024.108082  479. Papageorgiou M, Lyrakou M, Kyriacou A, Biver E, Yannakoulia M. Fermented Dairy Products, Musculoskeletal and Mental Health in Older Adults: is There Evidence to Support Benefits that go Beyond Those of Non-Fermented Dairy Products? Calcified Tissue International. 2024;115(5):480-97. doi: 10.1007/s00223-024-01291-4 PMID:WOS:001317328900001  480. Papamichail P, Sagredaki ML, Bouzineki C, Kanellopoulou S, Lyros E, Christakou A. The Effectiveness of an Exercise Program on Muscle Strength and Range of Motion on Upper Limbs, Functional Ability and Depression at Early Stage of Dementia. Journal of clinical medicine. 2024;13(14). doi: 10.3390/jcm13144136 PMID:CN-02748624  481. Park HJ, Thapa N, Bae S, Yang JG, Choi J, Noh ES, et al. Association between Physical Function, Mental Function and Frailty in Community-Dwelling Older Adults: A Cross-Sectional Study. Journal of Clinical Medicine. 2024;13(11). doi: 10.3390/jcm13113207  482. Park JY, Lengacher CA, Rodriguez CS, Meng H, Kip KE, Morgan S, et al. The Moderating Role of Genetics on the Effectiveness of the Mindfulness-Based Stress Reduction for Breast Cancer (MBSR(BC)) Program on Cognitive Impairment. Biological research for nursing. 2024:10998004241289629. doi: 10.1177/10998004241289629 PMID:CN-02768138  483. Park S, Baek S, Lee SY, Moon K, Kim JH, Lim C. Development and Performance Comparison of Cognitive Function Evaluation Models Using Machine Learning Algorithms. 2024.  484. Parker-Autry C, Mezes C, Namugosa M. E-FRAILTY REPLACESGAIT SPEEDTOIDENTIFY FRAILTY IN GERIATRIC INCONTINENCE. Urogynecology. 2024;30(10):S214. doi:  485. Patani K, Tungikar S, Sinha R. Effectiveness of proprioceptive and exteroceptive stimulation to improve motor performance in stroke. Pravara medical review. 2024;16(1):83‐8. doi: 10.36848/PMR/2024/11111.110777 PMID:CN-02698914  486. Paulsen AJ, Pinto AA, Schubert CR, Chappell RJ, Chen Y, Engelman CD, et al. Midlife sensory and motor functions improve prediction of blood-based measures of neurodegeneration and Alzheimer's disease in late middle-age. Alzheimers Dement (Amst). 2024;16(1):e12564. doi: 10.1002/dad2.12564 PMID:38476637  487. Pavon-Pulido N, Dominguez L, Blasco-Garcia JD, Veronese N, Lucas-Ochoa A-M, Fernandez-Villalba E, et al. Identification of Predictors of Sarcopenia in Older Adults Using Machine Learning: English Longitudinal Study of Ageing. Journal of Clinical Medicine. 2024;13(22). doi: 10.3390/jcm13226794 PMID:WOS:001365300200001  488. Pearce RB, Gontsarova A, Richardson D, Methley AM, Watt H, Tsang K, et al. Shunting for idiopathic normal pressure hydrocephalus. Cochrane Database of Systematic Reviews. 2024;(8). doi: 10.1002/14651858.CD014923.pub2 PMID:CD014923  489. Peng T-C, Chiou J-M, Chen YC, Chen J-H. Handgrip strength asymmetry and cognitive impairment risk: Insights from a seven-year prospective cohort study. Journal of Nutrition Health & Aging. 2024;28(1). doi: 10.1016/j.jnha.2023.100004 PMID:WOS:001185753800001  490. Pereira M, Tocino MLS, Mas-Fontao S, Manso P, Burgos M, Carneiro D, et al. Dependency and frailty in the older haemodialysis patient. Bmc Geriatrics. 2024;24(1). doi: 10.1186/s12877-024-04973-8 PMID:WOS:001218457800003  491. Petracca M, Petsas N, Sellitto G, Ruotolo I, Livi C, Bonanno V, et al. Telerehabilitation and onsite rehabilitation effectively improve quality of life, fatigue, balance, and cognition in people with multiple sclerosis: an interventional study. Frontiers in neurology. 2024;15. doi: 10.3389/fneur.2024.1394867 PMID:CN-02761083  492. Phang HJ, Heimler SR, Scandalis LM, Wing D, Moran R, Nichols JF, et al. Protocol for the San Diego Nathan Shock Center Clinical Cohort: A new resource for studies of human aging. BMJ Open. 2024;14(6). doi: 10.1136/bmjopen-2023-082659  493. Phannarus H, Chansaengpetch S, Virojskulchai T, Pengsorn N, Chaopanitwet P, Vannachavee U, et al. Prevalence of Geriatric Syndromes and Satisfaction of Service in Older People Receiving Annual Health Screening at the Check-up Clinic, Siriraj Hospital. Siriraj Medical Journal. 2025;77(1):51-63. doi: 10.33192/smj.v77i1.271734  494. Pigłowska M, Corsonello A, Kostka T, Roller-Wirnsberger R, Wirnsberger G, Ärnlöv J, et al. Limited predictive value of bioelectrical phase angle for the development of sarcopenia in older Europeans. J Nutr Health Aging. 2024;28(12):100386. doi: 10.1016/j.jnha.2024.100386 PMID:39413686  495. Pimentel GD, Dela Vega MCM, Mendes H, Mainardi LG. Elevated neutrophil-to-lymphocyte ratio negatively impacts quality of life and increases fatigue in hospitalized mix cancer patients. 2024. p. 1289.  496. Ploug M, Qvist N, Jacobsen BG, Kroijer R, Nielsen J, Knudsen T. The impact of iron deficiency on patients under evaluation for colorectal cancer, a prospective cross-sectional study. Scandinavian Journal of Gastroenterology. 2024;59(9):1055-61. doi: 10.1080/00365521.2024.2373117  497. Podlewska AM, Batzu L, Soukup T, Sevdalis N, Bakolis I, Derbyshire-Fox F, et al. The PD-Ballet study: study protocol for a randomised controlled single-blind hybrid type 2 clinical trial evaluating the effects of ballet dancing on motor and non-motor symptoms in Parkinson’s disease. BMC complementary medicine and therapies. 2024;24(1). doi: 10.1186/s12906-023-04296-y PMID:CN-02654559  498. Pollak C, Verghese J, Blumen HM. Longitudinal Associations of Social Support and Gait Speed Decline in Aging. J Gerontol A Biol Sci Med Sci. 2024;79(3). doi: 10.1093/gerona/glad250 PMID:37886832  499. Pratt J, Dalla Via J, Sale C, Gebre AK, Stephan BCM, Laws S, et al. Apolipoprotein ɛ4 Is Associated With Increased Risk of Fall- and Fracture-Related Hospitalization: The Perth Longitudinal Study of Ageing Women. The journals of gerontology Series A, Biological sciences and medical sciences. 2024;79(8). doi: 10.1093/gerona/glae134  500. Price H, Edwards E, Thomas C, Gray L. Using poisons Centre data to identify potential safety risks associated with the use of monitored dosage systems. Age and Ageing. 2024;53:19-. doi: 10.1093/ageing/afae139.072 PMID:WOS:001288218000089  501. Puranen T, Hiltunen K, Pitkälä KH, Roitto HM, Mäntylä P, Saarela RKT. Association of oral frailty with falls in long-term care residents. European Geriatric Medicine. 2025;16(1):191-5. doi: 10.1007/s41999-024-01088-6  502. Qaisar R, Hussain MA, Franzese F, Karim A, Ahmad F, Awad A, et al. Predictors of the onset of low handgrip strength in Europe: a longitudinal study of 42,183 older adults from 15 countries. Aging Clinical and Experimental Research. 2024;36(1). doi: 10.1007/s40520-024-02800-z  503. Qaisar R, Iqbal MS, Karim A, Ahmad F. Resistance Exercise Reduces Sarcopenia by Repairing Leaky Gut in Patients with Alzheimer's Disease. Arch Med Res. 2024;55(5):103025. doi: 10.1016/j.arcmed.2024.103025 PMID:38879906  504. Qi Y-M, Li H-T, Chang S-M, Hu S-J, Du S-C, Liu C-D, et al. Sarcopenia is a risk factor for postoperative delirium in geriatric hip fracture patients: a retrospective study. Frontiers in Medicine. 2025;11. doi: 10.3389/fmed.2024.1526240 PMID:WOS:001400265400001  505. Qiao YS, Blackwell TL, Cawthon PM, Coen PM, Cummings SR, Distefano G, et al. Associations of accelerometry-measured and self-reported physical activity and sedentary behavior with skeletal muscle energetics: The Study of Muscle, Mobility and Aging (SOMMA). Journal of Sport and Health Science. 2024;13(5):621-30. doi: 10.1016/j.jshs.2024.02.001  506. Qin X. Application of exercise intervention based on multi-theory model in postoperative patients with colorectal cancer. Master's Degree, Jilin University. 2024. Available from:https://d.wanfangdata.com.cn/thesis/ChhUaGVzaXNOZXdTMjAyNDA5MjAxNTE3MjUSCUQwMzUwNjY0NhoIbHFtcWl0a3A%3D  507. Ragusa FS, Veronese N, Vernuccio L, Dominguez LJ, Smith L, Bolzetta F, et al. Mild cognitive impairment predicts the onset of Sarcopenia: a longitudinal analysis from the English Longitudinal Study on Ageing. Aging Clin Exp Res. 2024;36(1):129. doi: 10.1007/s40520-024-02781-z PMID:38856870  508. Rahimi F, Saadat M, Hessam M, Ravanbakhsh M, Monjezi S. Post-COVID-19 physical and cognitive impairments and associations with quality of life: a cross-sectional study. Front Sports Act Living. 2024;6:1246585. doi: 10.3389/fspor.2024.1246585 PMID:38504691  509. Rasheed M, Sayers K, King L, Maher J, Ryan S, Donnellan C. Chronic Kidney Disease and Frailty: Insights from a Cross-Sectional Study in Older Adults. Age and Ageing. 2024;53:iv61. doi: 10.1093/ageing/afae178.244  510. Reinoso-Parraga PP, Arain SJ, Perkisas S, Colino RM, Montalvo JIG, Deniz VM, et al. Assessing quantitative sonographic changes in the muscle mass of geriatric patients hospitalised using point of care ultrasound. Age and Ageing. 2024;53. doi: 10.1093/ageing/afae139.002 PMID:WOS:001288218000035  511. Ridley B, Nonino F, Baldin E, Casetta I, Iuliano G, Filippini G. Azathioprine for people with multiple sclerosis. Cochrane Database of Systematic Reviews. 2024;(12). doi: 10.1002/14651858.CD015005.pub2 PMID:CD015005  512. Rivan NFM, Ludin AFM, Clark BC, Shahar S. Predictors for the development of motoric cognitive risk syndrome in older adults. BMC geriatrics. 2024;24(1):575. doi: 10.1186/s12877-024-05179-8  513. Rivasi G, Ceolin L, Turrin G, Tortu V, D'Andria MF, Testa GD, et al. Prevalence and correlates of frailty in older hypertensive outpatients according to different tools: the HYPER-FRAIL pilot study. Journal of Hypertension. 2024;42(1):86-94. doi: 10.1097/HJH.0000000000003559  514. Rodrigues RN, Furtado G, Carballeira E, Sanchez-Sanchez JL, Herrero AC, Silva FM, et al. Protective effects of elastic band training-detraining on Fall risk, power, body composition, and cognition in older adults with mild cognitive impairment: a 40-week trial. Journal of bodywork and movement therapies. 2025;42:23‐33. doi: 10.1016/j.jbmt.2024.11.022 PMID:CN-02799108  515. Rosko AE, Huang Y, Wall SA, Mims A, Woyach J, Presley C, et al. Predictive ability of the Cancer and Aging Research Group chemotherapy toxicity calculator in hematologic malignancy. J Geriatr Oncol. 2025;16(1):102144. doi: 10.1016/j.jgo.2024.102144 PMID:39505607  516. Rubio-Zarapuz A, Apolo-Arenas MD, Tomas-Carus P, Tornero-Aguilera JF, Clemente-Suárez VJ, Parraca JA. Comparative Analysis of Psychophysiological Responses in Fibromyalgia Patients: evaluating Neuromodulation Alone, Neuromodulation Combined with Virtual Reality, and Exercise Interventions. Medicina (Kaunas, Lithuania). 2024;60(3). doi: 10.3390/medicina60030404 PMID:CN-02679342  517. Sahin UK, Acaröz S. Predictors of the Disability in Activities of Daily Living in Nursing Home Residents: A Descriptive Study. Experimental aging research. 2024:1-14. doi: 10.1080/0361073X.2024.2421686  518. Sala G, Nishita Y, Tange C, Zhang S, Ando F, Shimokata H, et al. Differential Longitudinal Associations Between Domains of Cognitive Function and Physical Function: A 20-Year Follow-Up Study. The journals of gerontology Series B, Psychological sciences and social sciences. 2024;79(1). doi: 10.1093/geronb/gbad156  519. Salazar-Talla L, Alcantara-Diaz AL, Urrunaga-Pastor D, Runzer-Colmenares FM, Parodi JF. Motoric cognitive risk syndrome as a predictor of mortality in older male adults with cancer: A prospective cohort study in Peru. Geriatric nursing (New York, NY). 2024;60:497-503. doi: 10.1016/j.gerinurse.2024.10.007  520. Salinas-Rodríguez A, Rojas-Botero ML, Rivera-Almaraz A, Fernández-Niño JA, Montañez-Hernández JC, Manrique-Espinoza B. Long-term inequalities in health among older Mexican adults: An outcome-wide analysis. SSM - Population Health. 2024;26. doi: 10.1016/j.ssmph.2024.101684  521. Samuelsson J, Marseglia A, Wallengren O, Lindberg O, Dartora C, Cedres N, et al. Association of body composition with neuroimaging biomarkers and cognitive function; a population-based study of 70-year-olds. eBioMedicine. 2025;112. doi: 10.1016/j.ebiom.2024.105555  522. Sankar K, Billah AAM, Sankar V, Singaram V, Viswanathan S. Impact of Vortioxetine and Fluoxetine on Cognition and Health Related Quality of Life among Major Depressive Disorder Patients with and without Metabolic Syndrome. Journal of young pharmacists. 2024;16(1):72‐80. doi: 10.5530/jyp.2024.16.10 PMID:CN-02667594  523. Sardella A, Bellone F, Mandraffino G, Malacarne F, Maltese G, Squadrito G, et al. Is the pentagon-copying task more than a cognitive feature? Associations with handgrip strength, gait speed and frailty in older adults. Gerontology. 2024;70(1):1-6. doi: 10.1159/000534555 PMID:WOS:001095984000001  524. Sato R, Vatic M, da Fonseca GWP, Anker SD, von Haehling S. Biological basis and treatment of frailty and sarcopenia. Cardiovascular Research. 2024;120(9):982-98. doi: 10.1093/cvr/cvae073 PMID:WOS:001237192500001  525. Sawaya Y, Hirose T, Shiba T, Sato R, Yin L, Kubo A, et al. Decrease in the usual walking speed and body fat percentage associated with a deterioration in long-term care insurance certification levels. PeerJ. 2024;12(6). doi: 10.7717/peerj.17529  526. Schmitz B, Garbsch R, Schaefer H, Kotewitsch M, Mooren J, Waranski M, et al. Sex-specific differences in exercise-based rehabilitation of patients with Post-COVID-19 Syndrome. European Journal of Preventive Cardiology. 2024;31:I305. doi: 10.1093/eurjpc/zwae175.212  527. Schubert CR, Pinto AA, Paulsen AJ, Chappell RJ, Chen Y, Engelman CD, et al. Midlife sensory and motor functions improve long-term predictions of cognitive decline and incidence of cognitive impairment. Alzheimers Dement (Amst). 2024;16(1):e12543. doi: 10.1002/dad2.12543 PMID:38288267  528. Scott FAM, Butler M, Rogers JP. The limited clinical utility of a routine creatine kinase (CK) on admission to a psychiatric inpatient unit. BMC Psychiatry. 2024;24(1). doi: 10.1186/s12888-024-06386-8  529. Sebastião E, Siqueira V, Bakare JO, Kamari M, Motl RW. Cognitive Function in Frail Older Adults With Multiple Sclerosis: an Exploratory Study Using Secondary Data Analysis. International journal of MS care. 2024;26(Q4):315‐20. doi: 10.7224/1537-2073.2023-085 PMID:CN-02784932  530. Sefcik JS, Coates MC, Petrovsky DV, Glasofer A, Okoye S, Vader DT, et al. Factors Associated With Outdoor Frequency Among U.S. Community-Dwelling Medicare Beneficiaries: A Cross-Sectional Study. Research in gerontological nursing. 2025;18(1):40-52. doi: 10.3928/19404921-20241211-06  531. Seferoğlu M, Aksoy MK, Tunç A. Hand Grip Strength as a Predictive Tool for Upper Extremity Functionality, Balance, and Quality of Life in People With Multiple Sclerosis. Int J MS Care. 2024;26(Q3):134-9. doi: 10.7224/1537-2073.2022-030 PMID:38872997  532. Sevlever GE, Crivelli L, Salinas RM, Charamelo A, Delgado C. S4: the LatAm-FINGERS Initiative: the First Non-Pharmacological Randomized Controlled Trial to Prevent Cognitive Decline Across Latin America. International psychogeriatrics. 2024;36:10‐3. doi: 10.1017/S1041610224001029 PMID:CN-02805820  533. Shaji S, Elayaperumal I, Reddy YD. GERIATRIC ASSESSMENT AND NUTRITIONAL STATUS OF ELDERLY PATIENTS (AGE>65 YEARS) ON HEMODIALYSIS FROM A TERITIARY CARE CENTRE IN SOUTH INDIA. 2025. p. S407.  534. Shin G, Yoon J, Choi Y, Leigh JH. The relative contribution of environmental factors and human factors on driving cessation among older people in South Korea. Archives of Physical Medicine and Rehabilitation. 2024;105(4):e77. doi: 10.1016/j.apmr.2024.02.216  535. Shiraishi A, Yoshimura Y, Nagano F, Matsumoto A, Shimazu S, Kido Y, et al. Impaired oral health associations with cognitive and motor decline in activities of daily living independence during hospitalization: insights from a post-stroke cohort. Journal of Stroke and Cerebrovascular Diseases. 2024;33(11). doi: 10.1016/j.jstrokecerebrovasdis.2024.107966  536. Shoji T, Kogure K, Toda N, Hakoshima M, Katsuyama H, Yanai H, et al. Association between comorbidities associated with diabetes and higher-level functional status in older patients with type 2 diabetes mellitus: a cross sectional study. European Geriatric Medicine. 2024;15(4):1101-10. doi: 10.1007/s41999-024-00937-8  537. Shyu SW, Lin CF, Yang SH, Chu WM, Hsu CY, Lin SY, et al. Association of oral health with geriatric syndromes and clinical outcomes in hospitalized older adults. J Nutr Health Aging. 2024;28(11):100385. doi: 10.1016/j.jnha.2024.100385 PMID:39471775  538. Siegmund LA, Novosel LM, Bena JF, Morrison SL. Frailty prevalence and associated factors in community dwelling older adults: An examination of the frailty care model. Geriatric Nursing. 2025;61:672-80. doi: 10.1016/j.gerinurse.2024.10.041 PMID:WOS:001423790700001  539. Silva EAM, Batista LR, Braga MAF, Teixeira-Salmela LF, Faria C, Faria-Fortini I. Predicting self-perceived manual ability at three and six months after stroke: A prospective longitudinal study. J Stroke Cerebrovasc Dis. 2024;33(1):107479. doi: 10.1016/j.jstrokecerebrovasdis.2023.107479 PMID:37984045  540. Sinha S, Gabriel VA, Arora RK, Shin W, Scott J, Bharadia SK, et al. Interventions for postburn pruritus. Cochrane Database of Systematic Reviews. 2024;(6). doi: 10.1002/14651858.CD013468.pub2 PMID:CD013468  541. Skow LF, Sharrett AR, Gottesman RF, Coresh J, Deal JA, Palta P, et al. Mid-Life Vascular Risk and Rate of Physical Function Decline Among Older Adults: The Atherosclerosis Risk in Communities (ARIC) Study. J Gerontol A Biol Sci Med Sci. 2024;79(2). doi: 10.1093/gerona/glad210 PMID:37659100  542. Smith SY, Aylwin CF, Daniels TF, Greer JL, Kunces LJ, Lili L, et al. Kavalactones support motivation to move during intensive training in males preparing for military special operations forces. Journal of the International Society of Sports Nutrition. 2024;21(1):2377194. doi: 10.1080/15502783.2024.2377194 PMID:CN-02727329  543. Sobrini-Morillo P, Ravot C, Herlédan C, Sánchez-Castellano C, Cruz-Jentoft AJ, Falandry C. Real-world experience with CDK4-6 inhibition in the old and oldest old with a diagnosis of breast cancer. Semin Oncol. 2024;51(3-4):95-105. doi: 10.1053/j.seminoncol.2024.01.003 PMID:38604898  544. Sobue Y, Suzuki M, Ohashi Y, Ishikawa H, Asai S, Imagama S. FRAILTY IS ASSOCIATED WITH A LACK OF OPPORTUNITIES FOR VOCALIZATION IN RHEUMATOID ARTHRITIS PATIENTS. Annals of the Rheumatic Diseases. 2024;83:751. doi: 10.1136/annrheumdis-2024-eular.152  545. Solaro C, Di Giovanni R, Grange E, Brichetto G, Mueller M, Tacchino A, et al. Influence of cognition on the correlation between objective and subjective upper limb measures in people with multiple sclerosis. Neurological Sciences. 2024;45(6):2783-9. doi: 10.1007/s10072-023-07286-7  546. Song X, Huang S, Li M, Chen X. Evaluation of Sarcopenia screening indices as predictors of mortality in older patients with Alzheimer's disease. BMC Geriatr. 2024;24(1):996. doi: 10.1186/s12877-024-05589-8 PMID:39633305  547. Soysal P, Smith L. The prevalence and co-existence of geriatric syndromes in older patients with dementia compared to those without dementia. Aging Clinical and Experimental Research. 2024;36(1). doi: 10.1007/s40520-024-02724-8 PMID:WOS:001181465700001  548. Spangler HB, Lynch DH, Howard AG, Tien HC, Du S, Zhang B, et al. Association Between Mid-arm Muscle Circumference and Cognitive Function: A Longitudinal Study of Chinese Adults. Journal of Geriatric Psychiatry and Neurology. 2024;37(4):272-81. doi: 10.1177/08919887231218087  549. Spiga F, Davies AL, Tomlinson E, Moore THM, Dawson S, Breheny K, et al. Interventions to prevent obesity in children aged 5 to 11 years old. Cochrane Database of Systematic Reviews. 2024;(5). doi: 10.1002/14651858.CD015328.pub2 PMID:CD015328  550. Stauder M, Hiersche KJ, Hayes SM. Examining cross-sectional and longitudinal relationships between multidomain physical fitness metrics, education, and cognition in Black older adults. Neuropsychol Dev Cogn B Aging Neuropsychol Cogn. 2024;31(4):646-60. doi: 10.1080/13825585.2023.2225848 PMID:37345613  551. Stephan Y, Sutin AR, Luchetti M, Aschwanden D, Karakose S, Terracciano A. Balance, Strength, and Risk of Dementia: Findings From the Health and Retirement Study and the English Longitudinal Study of Ageing. The journals of gerontology Series A, Biological sciences and medical sciences. 2024;79(8). doi: 10.1093/gerona/glae165  552. Strand BH, Håberg AK, Eyjólfsdóttir HS, Kok A, Skirbekk V, Huxhold O, et al. Spousal bereavement and its effects on later life physical and cognitive capability: the Tromsø study. GeroScience. 2024;46(6):6055-69. doi: 10.1007/s11357-024-01150-y  553. Stuckenschneider T, Sanders ML, Devenney KE, Aaronson JA, Abeln V, Claassen J, et al. NeuroExercise: the Effect of a 12-Month Exercise Intervention on Cognition in Mild Cognitive Impairment-A Multicenter Randomized Controlled Trial. Frontiers in aging neuroscience. 2020;12:621947. doi: 10.3389/fnagi.2020.621947 PMID:CN-02647900  554. Su S, Zhou Y, Wang K, Liu A, Lei L, Ma H, et al. Effects of household solid fuel use on sarcopenia in middle-aged and older adults: evidence from a nationwide cohort study. Frontiers in public health. 2024;12:1337979. doi: 10.3389/fpubh.2024.1337979  555. Su Y, Yuki M, Huang H, Luo N, Wang L. Development of a Screening Tool for Oral Frailty in Community-Dwelling Older Adults: A Cross-Sectional Study. Journal of the American Medical Directors Association. 2024;25(9). doi: 10.1016/j.jamda.2024.105171 PMID:WOS:001288311400001  556. Sullivan AM, Gibbs HM. Comparison of Malnutrition Assessment Tools for Identification of Malnutrition in Community-Dwelling Older Adults With Dementia. 2024.  557. Sultana M, Camicioli R, Dixon RA, Whitehead S, Pieruccini-Faria F, Petrotchenko E, et al. A Metabolomics Analysis of a Novel Phenotype of Older Adults at Higher Risk of Dementia. Journal of Alzheimer's Disease. 2024;99(s2):S317-S25. doi: 10.3233/JAD-230683  558. Sultanik P, Lherault G, Bouzbib C, Ratziu V, Pais R, Mouri S, et al. Prevalence and prognosis of patients with MASLD-related cirrhosis after an ICU hospitalization in France: A single-centre prospective study. Alimentary Pharmacology and Therapeutics. 2024;60(6):796-810. doi: 10.1111/apt.18165  559. Swinnen N, de Bruin ED, Guimarães V, Dumoulin C, De Jong J, Akkerman R, et al. The feasibility of a stepping exergame prototype for older adults with major neurocognitive disorder residing in a long-term care facility: a mixed methods pilot study. Disability and rehabilitation. 2024;46(5):896‐910. doi: 10.1080/09638288.2023.2182916 PMID:CN-02745801  560. t6g86c RBR. Evaluation of the effects and experience of using the SOPeD Exercise program for Feet and Ankles by individuals with Diabetic Foot: a randomized clinical trial. https://trialsearchwhoint/Trial2aspx?TrialID=RBR-7t6g86c. 2024. doi: PMID:CN-02732134  561. Tabue Teguo M, Letchimy L, Rinaldo L, Bonnet M, Tchero H, Simo-Tabue N, et al. Malnutrition and Its Determinants among Older Adults Living in French Caribbean Nursing Homes: A Cross-Sectional Study. Nutrients. 2024;16(14). doi: 10.3390/nu16142208  562. Tajimi T, Hirabayashi N, Furuta Y, Nakazawa T, Honda T, Hata J, et al. Association of sarcopenia with regional brain atrophy and white matter lesions in a general older population: the Hisayama Study. GeroScience. 2025;47(1):1187-98. doi: 10.1007/s11357-024-01289-8  563. Takahashi J, Kawai H, Ejiri M, Fujiwara Y, Hirano H, Sasai H, et al. Predicting the incidence of mild cognitive impairment with a computer-based cognitive assessment tool in community-dwelling older adults: The Otassha study. PLoS ONE. 2024;19(1 January). doi: 10.1371/journal.pone.0297433  564. Takimoto K, Takebayashi H, Yoshikawa Y, Sasano H, Tsujishita S, Ikeda K. Association between Motoric Cognitive Risk Syndrome and Indicators of Reflecting Independent Living among Community-Dwelling Older Adults in Japan: A Cross-Sectional Study. Healthcare. 2024;12(18). doi: 10.3390/healthcare12181808 PMID:WOS:001326245800001  565. Tan LF, Chan YH, Denishkrshna A, Merchant RA. Association between different skeletal muscle mass indices, physical function, and inflammation in obese pre-frail older adults. Archives of Gerontology and Geriatrics. 2024;118. doi: 10.1016/j.archger.2023.105289  566. Tan You Mei C, Seah Si Ying S, Yanshan DL, Koh SV, Karthikeyan G, Xia Jiawen O, et al. Prevalence and factors associated with sarcopenia among older adults in a post-acute hospital in Singapore. PLoS One. 2024;19(1):e0291702. doi: 10.1371/journal.pone.0291702 PMID:38285652  567. Tanguay AFN, Gardam O, Archibald J, Ayson G, Atance CM. Using an episodic specificity induction to improve children's future thinking. Frontiers in psychology. 2023;14:1249090. doi: 10.3389/fpsyg.2023.1249090 PMID:CN-02778498  568. Taniguchi Y, Kitamura A, Hata T, Fujita K, Abe T, Nofuji Y, et al. Frailty Trajectories and Its Associated Factors in Japanese Older Adults. Journal of Frailty and Aging. 2024;13(3):233-9. doi: 10.14283/jfa.2024.51  569. Tariq A, Zadeh SAM, Ammar M, Mousavizadeh N, Hajary A, Mohamadi S. Relationship between multiple morbidities and performance on the Timed Up and Go test in elderly patients: a cross-sectional study. Bmj Open. 2025;15(1). doi: 10.1136/bmjopen-2024-088950 PMID:WOS:001415437600001  570. Tesarz J, Lange H, Kirchner M, Görlach A, Eich W, Friederich HC. Efficacy of supervised immersive virtual reality-based training for the treatment of chronic fatigue in post-COVID syndrome: study protocol for a double-blind randomized controlled trial (IFATICO Trial). Trials. 2024;25(1):232. doi: 10.1186/s13063-024-08032-w PMID:CN-02686610  571. Theodorakis N, Nikolaou M, Hitas C, Anagnostou D, Kreouzi M, Kalantzi S, et al. Comprehensive Peri-Operative Risk Assessment and Management of Geriatric Patients. Diagnostics. 2024;14(19). doi: 10.3390/diagnostics14192153  572. Theodoridis X, Poulia KA, Chourdakis M. What's new about hydration in dementia? Current opinion in clinical nutrition and metabolic care. 2025;28(1):20-4. doi: 10.1097/MCO.0000000000001089  573. Thiel U, Stiebler M, Labott BK, Bappert J, Langhans C, Halfpaap N, et al. DiADEM-Dance against Dementia-Effect of a Six-Month Dance Intervention on Physical Fitness in Older Adults with Mild Cognitive Impairment: a Randomized, Controlled Trial. Journal of personalized medicine. 2024;14(8). doi: 10.3390/jpm14080888 PMID:CN-02747011  574. Thompson C, Trushina E, Fairweather D, Block D, Wang Z, Foster N, et al. Iron Deficiency in Collegiate Athletes Obtaining Preparticipation Hemoglobinopathy Screening in the Upper Midwest. Pediatr Blood Cancer. 2025;72(2):e31437. doi: 10.1002/pbc.31437 PMID:39529284  575. Thu Ya M, Hasegawa Y, Sta Maria MT, Hattori H, Kusunoki H, Nagai K, et al. Predicting cognitive function changes from oral health status: a longitudinal cohort study. Scientific reports. 2024;14(1):24153. doi: 10.1038/s41598-024-75169-8  576. Tian Q, Greig EE, Walker KA, Fishbein KW, Spencer RG, Resnick SM, et al. Plasma metabolomic markers underlying skeletal muscle mitochondrial function relationships with cognition and motor function. Age and Ageing. 2024;53(4). doi: 10.1093/ageing/afae079  577. Tian Q, Lee PR, Yang Q, Moore AZ, Landman BA, Resnick SM, et al. The mediation roles of intermuscular fat and inflammation in muscle mitochondrial associations with cognition and mobility. Journal of Cachexia, Sarcopenia and Muscle. 2024;15(1):138-48. doi: 10.1002/jcsm.13413  578. Tiftik T, Kara M, Mülkoğlu C, Çiftçi İ, Çelik ÖF, Durmuş ME, et al. The Paradoxical Relationship Among Diabetes Mellitus, Osteoporosis and Sarcopenia: The PARADOS Study. Clinical Nutrition ESPEN. 2025;65:258-63. doi: 10.1016/j.clnesp.2024.12.009  579. Todhunter-Brown A, Sellers CE, Baer GD, Choo PL, Cowie J, Cheyne JD, et al. Physical rehabilitation approaches for the recovery of function and mobility following stroke. Cochrane Database of Systematic Reviews. 2025;(2). doi: 10.1002/14651858.CD001920.pub4 PMID:CD001920  580. Togashi S, Ohinata H, Noguchi T, Wakabayashi H, Nakamichi M, Shimizu A, et al. Polypharmacy, Potentially Inappropriate Medications, and Dysphagia in Older Inpatients: A Multi-Center Cohort Study. Annals of Geriatric Medicine and Research. 2024;28(1):86-94. doi: 10.4235/agmr.23.0203  581. Topcuoglu C, Vardar Yagli N, Aykan HH, Ertugrul I, Karagoz T, Saglam M. Exploring frailty: muscle strength, functional capacity, activities of daily living and cognition in adult congenital heart disease. Disability and rehabilitation. 2024:1-7. doi: 10.1080/09638288.2024.2417775  582. Toyoshima K, Tamura Y, Murao Y, Kodera R, Oba K, Ishikawa J, et al. Risk factor of disability as new certification of long-term care needs in older Japanese adults with diabetes mellitus: A longitudinal study. Geriatrics and Gerontology International. 2024;24(10):1030-8. doi: 10.1111/ggi.14969  583. Trinh JQ, Nilles JD, Ellithi M, Haddadin MM, Maness-Harris L, Gundabolu K, et al. Metabolic syndrome and symptom burden in allogeneic hematopoietic stem cell transplantation survivors. Future Oncol. 2024;20(40):3403-8. doi: 10.1080/14796694.2024.2431476 PMID:39580643  584. Tuan SH, Chang LH, Sun SF, Lin KL, Tsai YJ. Using exergame-based exercise to prevent and postpone the loss of muscle mass, muscle strength, cognition, and functional performance among elders in rural long-term care facilities: a protocol for a randomized controlled trial. Frontiers in medicine. 2022;9:1071409. doi: 10.3389/fmed.2022.1071409 PMID:CN-02637310  585. Turco I, Johnson-Akeju O, Namirembe G. Postoperative Delirium and Long-Term Cognition Using MoCA and PROMIS Applied Cognition- Abilities Scores. Anesthesia and analgesia. 2023;136(5):519‐20. doi: 10.1213/01.ane.0000977652.27377.97 PMID:CN-02776225  586. Úbeda-D'Ocasar E, González-Gerstner D, Cimadevilla-Fernández-Pola E, Ojedo-Martín C, Hernández-Lougedo J, Hervás-Pérez JP. Effects of Diathermy on Pain in Women with Fibromyalgia: a Randomized Controlled Trial. Biomedicines. 2024;12(7). doi: 10.3390/biomedicines12071465 PMID:CN-02733057  587. Ullah S, Noureddine Z, Sathian B, Narayanankutty K, Asirvatham T, Abubacker M, et al. Rehabilitation and functional outcomes of COVID-19 patients in a rehabilitation hospital in Qatar. Qatar Med J. 2024;2024(3):45. doi: 10.5339/qmj.2024.45 PMID:39372687  588. Umehara K, Yamamoto A, Kitagawa T, Yoshimaru K, Ishikawa A. Effect of physical frailty on maximum phonation time in community-dwelling older individuals who visited a neurology outpatient clinic. Journal of oral rehabilitation. 2024;51(7):1221-8. doi: 10.1111/joor.13698  589. Valenzuela A, Lera L, Marquez C, Albal C. Nutritional Status of Institutionalized Elderly and Its Relationship with Functional Status During 2019. Revista Medica De Chile. 2024;152(3):360-75. doi: PMID:WOS:001300985100009  590. van der Steen JT, van der Wouden JC, Methley AM, Smaling HA, Vink AC, Bruinsma MS. Music‐based therapeutic interventions for people with dementia. Cochrane Database of Systematic Reviews. 2025;(3). doi: 10.1002/14651858.CD003477.pub5 PMID:CD003477  591. Varan HD, Ceker E, Cataltepe E, Gungor F, Fadiloglu A, Borazan FY. Predictive value of adductor pollicis muscle thickness for ultrasound-based sarcopenia in older adults. Nutrition in Clinical Practice. 2024;39(3):619-25. doi: 10.1002/ncp.11149  592. Vazquez JP, Verghese J, Barzilai N, Milman S, Blumen HM. White Matter Hyperintensities Are Associated with Slower Gait Speed in Older Adults Without Dementia. Neuro-degenerative diseases. 2024. doi: 10.1159/000538944  593. Vázquez-Fernández A, Caballero F, Yévenes-Briones H, Struijk E, Baylin A, Fung T, et al. Plant and Animal Protein Intake and Transitions From Multimorbidity to Frailty and Mortality in Older Adults. Journal of Cachexia, Sarcopenia and Muscle. 2025;16(1). doi: 10.1002/jcsm.13729  594. Verstraeten LMG, Kreeftmeijer J, van Wijngaarden JP, Meskers CGM, Maier AB. Geriatric Syndromes Frequently (Co)-Occur in Geriatric Rehabilitation Inpatients: Restoring Health of Acutely Unwell Adults (RESORT) and Enhancing Muscle Power in Geriatric Rehabilitation (EMPOWER-GR). Arch Phys Med Rehabil. 2024;105(10):1854-61. doi: 10.1016/j.apmr.2024.05.021 PMID:38851557  595. Vityala Y, Tagaev T, Ramanujam SK, Kalapala IH, Kaggallu AB, Sultana S, et al. The association of insulin resistance and mild cognitive impairment in elderly patients with alzheimer's disease. 2024.  596. Vogt L, Amburgey K, Widjaja E, Dowling J. 42P Characterizing MRI brain abnormalities in X-linked myotubular myopathy. 2024.  597. Vordenberg SE, Davis RC, Strominger J, Marcus SC, Kim HM, Blow FC, et al. Clinician contributions to central nervous system-active polypharmacy among older adults with dementia in the United States. Journal of the American Geriatrics Society. 2025;73(2):422-30. doi: 10.1111/jgs.19256  598. Wan W. Effect of preoperative sarcopenia on postoperative delirium in elderly patients undergoing gastrointestinal surgery and factors influencing postoperative delirium. Master's Degree, Nanchang University. 2024. Available from:https://link.cnki.net/doi/10.27232/d.cnki.gnchu.2024.003677  599. Wang D. EFFECT OF URBAN PUBLIC SPACE LANDSCAPE ON ADJUVANT TREATMENT OF PATIENTS WITH DEPRESSION. Psychiatria Danubina. 2022;34:S872‐S3. doi: PMID:CN-02668778  600. Wang D. Study on the Efficacy and Anti-fatigue Mechanism of Rosemary Essential Oil. Master's Degree, Jilin University. 2024. Available from:https://d.wanfangdata.com.cn/thesis/ChhUaGVzaXNOZXdTMjAyNDA5MjAxNTE3MjUSCUQwMzUwNzQ0NRoIbHFtcWl0a3A%3D  601. Wang FY, Fan LJ, Huo LN, Lin Y, Zhang RG, Yang YH, et al. Predictors of mood disturbance in older adults: a longitudinal cohort study. Eur Geriatr Med. 2025. doi: 10.1007/s41999-025-01178-z PMID:40042774  602. Wang HY, Bai M, Wang J, Li Z, Wang J, Wang Q. Swallowing-related cough and health status of old adults: Results from a nationwide cross-sectional study. Chinese Medical Journal. 2024;137(20):2492-4. doi: 10.1097/CM9.0000000000003281  603. Wang L, Bian X, Liu L, He Q, Xu J, Chen X, et al. Association between cognitive function and skeletal muscle in patients undergoing maintenance hemodialysis. Frontiers in Endocrinology. 2024;15. doi: 10.3389/fendo.2024.1324867  604. Wang R, Marseglia A, Skoog J, Lindberg O, Pereira JB, Shams S, et al. Neuroimaging Correlates of 3 Distinct Physical-Cognitive Phenotypes in Cognitively Normal Older Adults: The Gothenburg H70 Cohort Study. Neurology. 2024;104(1). doi: 10.1212/WNL.0000000000210121  605. Wang R, Marseglia A, Skoog J, Lindberg O, Pereira JB, Shams S, et al. Neuroimaging Correlates of 3 Distinct Physical-Cognitive Phenotypes in Cognitively Normal Older Adults: The Gothenburg H70 Cohort Study. Neurology. 2025;104(1):e210121. doi: 10.1212/wnl.0000000000210121 PMID:39642342  606. Wang T, Geng J, Zeng X, Han R, Huh YE, Peng J. Exploring causal effects of sarcopenia on risk and progression of Parkinson disease by Mendelian randomization. Npj Parkinsons Disease. 2024;10(1). doi: 10.1038/s41531-024-00782-3 PMID:WOS:001299844700001  607. Wang X. Comment on “Relationships between muscle strength, lung function, and cognitive function in Chinese middle-aged and older adults: A study based on the China health and retirement longitudinal study (CHARLS)”: Subtitle: Muscle strength, lung function, and cognitive function. Journal of the Formosan Medical Association. 2024. doi: 10.1016/j.jfma.2024.11.002  608. Wang X, Qi C, Li X, Li D, Ding H, Shen J, et al. The role of dietary fats on cognition and sarcopenia in the elderly. Asia Pacific Journal of Clinical Nutrition. 2024;33(2):272-82. doi: PMID:WOS:001239232100011  609. Wang X, Wu L, Zhou H, He J. Grip strength and depressive symptoms in Chinese middle-aged and older adults: the mediating effects of cognitive function. Frontiers in Aging Neuroscience. 2024;16. doi: 10.3389/fnagi.2024.1455546  610. Wang Y. Clinical Features and Risk Factors Analysis of MOG-Antibody Associated Disease with Epilepsy. Master's Degree, Zhengzhou University. 2024. Available from:https://d.wanfangdata.com.cn/thesis/ChhUaGVzaXNOZXdTMjAyNDA5MjAxNTE3MjUSCFk0MzQ1MzEwGghscW1xaXRrcA%3D%3D  611. Wang Y, Dou L, Wang N, Zhao Y, Nie Y. An analysis of factors influencing cognitive dysfunction among older adults in Northwest China based on logistic regression and decision tree modelling. BMC geriatrics. 2024;24(1):405. doi: 10.1186/s12877-024-05024-y  612. Wang Y, Mu D, Wang Y. Association of low muscle mass with cognitive function and mortality in USA seniors: results from NHANES 1999-2002. BMC Geriatr. 2024;24(1):420. doi: 10.1186/s12877-024-05035-9 PMID:38734596  613. Wang Z, Tian J, Dai W, Zhang N, Wang J, Li Z. The Effectiveness and Safety Analysis of Duloxetine in Treating Comorbid Depression in Parkinson's Disease: A Retrospective Study. Actas Esp Psiquiatr. 2024;52(5):607-15. doi: 10.62641/aep.v52i5.1634 PMID:39403917  614. Wang Z, Wu Y, Zhu J, Fang Y. Machine learning-based prediction of sarcopenia in community-dwelling middle-aged and older adults: findings from the CHARLS. Psychogeriatrics. 2025;25(1). doi: 10.1111/psyg.13205  615. Washington SE, Bodde AE, Helsel BC, Bollinger RM, Smith N, Ptomey LT, et al. The association of dementia risk symptoms and functional activity in adults with Down syndrome. Alzheimer's and Dementia: Translational Research and Clinical Interventions. 2024;10(4). doi: 10.1002/trc2.70007  616. Webber K, Patel S, Kizer JR, Eastell R, Psaty BM, Newman AB, et al. Associations of Serum GDF-15 Levels with Physical Performance, Mobility Disability, Cognition, Cardiovascular Disease, and Mortality in Older Adults. medRxiv. 2024. doi: 10.1101/2024.08.07.24311629 PMID:39148825  617. Wei X, Li C, Liu D, Chen J, Ju Y, Liu J, et al. Profile of non-invasive physical health indicators associated with cognitive performance in Chinese older adults: evidence from the China Health and Retirement Longitudinal Study. BMC public health. 2025;25(1):420. doi: 10.1186/s12889-025-21479-z  618. Westbury LD, Harvey NC, Beaudart C, Bruyère O, Cauley JA, Cawthon P, et al. Predictive value of sarcopenia components for all-cause mortality: findings from population-based cohorts. Aging Clinical and Experimental Research. 2024;36(1). doi: 10.1007/s40520-024-02783-x  619. Wiersinga JHI, Diab HM, Peters MJL, Trappenburg MC, Rhodius-Meester HFM, Muller M. Cerebral small vessel disease and its relationship with all-cause mortality risk: Results from the Amsterdam Ageing cohort. Arch Gerontol Geriatr. 2025;129:105669. doi: 10.1016/j.archger.2024.105669 PMID:39481219  620. Wortmann F, Koch P, Khandanpour C, Von Bubnoff N. Virtual Reality-Assisted Exercise Therapy during Inpatient Treatment after Intensive Chemotherapy for Acute Myeloid Leukemia (AML) and after Autologous or Allogeneic Stem Cell Transplantation. Blood. 2023;142:5091. doi: 10.1182/blood-2023-174677 PMID:CN-02664243  621. Wu D, Yu X, Li F, Qiao W, Chen X. Geriatric syndrome awareness and its determinants in China: a cross-sectional study. BMC geriatrics. 2024;24(1):712. doi: 10.1186/s12877-024-05291-9  622. Wu F, Liu Y, Lin C, Haghbin N, Xia L, Li Y, et al. Correlation between fat-to-muscle mass ratio and cognitive impairment in elderly patients with type 2 diabetes mellitus: a cross-sectional study. BMC geriatrics. 2024;24(1):352. doi: 10.1186/s12877-024-04941-2  623. Wu J, Chen J, Wu J, Hsu CL. Intraindividual Variability Differentiated Older Adults with Physical Frailty and the Role of Education in the Maintenance of Cognitive Intraindividual Variability. 2024.  624. Wu J, Chen J, Wu J, Hsu CL. Intraindividual variability differentiated older adults with physical frailty and the role of education in the maintenance of cognitive intraindividual variability. PLoS One. 2025;20(3):e0304545. doi: 10.1371/journal.pone.0304545 PMID:40067791  625. Wu L, Wang Z, Zhou X, Kong Q, Zhang Y, Xu S, et al. Mismatch of MRI White Matter Hyperintensities and Gait Function in Patients With Cerebral Small Vessel Disease. Journal of Magnetic Resonance Imaging. 2024;60(2):550-8. doi: 10.1002/jmri.29121  626. Wu Y, Wang Z, Fang Y. Association of Performance on Multiple Cognitive Domains with Sarcopenia among Middle-Aged and Older Adults. Dement Geriatr Cogn Disord. 2024;53(3):162-7. doi: 10.1159/000538751 PMID:38593753  627. Wu Y, Xing Y, Long T, Huang J, Zhang Q, Liu H, et al. Association of Novel Surrogate Markers of Insulin Resistance and the Risk of Dementia in Older Adults: A Population-Based Study. Aging Medicine and Healthcare. 2024;15(3):137-44. doi: 10.33879/AMH.153.2023.06055  628. Xin W, Xu D, Dou Z, Jacques A, Umbella J, Fan Y, et al. Association between functional ability, chronic diseases and lifestyle risk factors in older community dwelling adults: protocol for a prospective observational Chinese cohort study. 2024.  629. Xin X, Liu Q, Jia S, Li S, Wang P, Wang X, et al. Correlation of muscle strength, information processing speed and cognitive function in the elderly with cognitive impairment--evidence from EEG. Front Aging Neurosci. 2025;17:1496725. doi: 10.3389/fnagi.2025.1496725 PMID:39906715  630. Xu J. Metabolic signature of frailty in old age and risk of developing serious adverse health outcomes association study based on UK Biobank. Master's Degree, Guangdong Medical University. 2024. Available from:https://d.wanfangdata.com.cn/thesis/ChhUaGVzaXNOZXdTMjAyNDA5MjAxNTE3MjUSCUQwMzQ4NTU4MRoIbHFtcWl0a3A%3D  631. Xu K, Hernández B, Arpawong TE, Camuzeaux S, Chekmeneva E, Crimmins EM, et al. Assessing Metabolic Ageing via DNA Methylation Surrogate Markers: A Multicohort Study in Britain, Ireland and the USA. Aging Cell. 2025. doi: 10.1111/acel.14484  632. Xu L, Xu W, Qin L. Association of cystatin C kidney function measures with motoric cognitive risk syndrome: evidence from two cohort studies. Journal of Nutrition, Health and Aging. 2025;29(3). doi: 10.1016/j.jnha.2025.100484  633. Xu Q, Yin W, Zhou X, Wang S, Chen S, Yang J, et al. Transcranial direct current stimulation for patients with walking difficulties caused by cerebral small vessel disease: a randomized controlled study. Frontiers in aging neuroscience. 2024;16:1511287. doi: 10.3389/fnagi.2024.1511287 PMID:CN-02802705  634. Xu S, Gong Z, Wang F, Cao M, Liu J, Chen C, et al. Intervention of muscle-building and antifrailty exercise combined with Baduanjin for frailty of different functional levels: study protocol for a randomised controlled trial. BMJ open. 2023;13(12):e074827. doi: 10.1136/bmjopen-2023-074827 PMID:CN-02635651  635. Xu S, Wen S, Yang Y, He J, Yang H, Qu Y, et al. Association between Body Composition Patterns, Cardiovascular Disease, and Risk of Neurodegenerative Disease in the UK Biobank. Neurology. 2024;103(4). doi: 10.1212/WNL.0000000000209659  636. Xu X, Li D, Zhang S. Retrospective study for correlation analysis of nutritional status with osteoporosis, sarcopenia and cognitive impairment in elderly patients with coronary heart disease. Frontiers in Cardiovascular Medicine. 2024;10. doi: 10.3389/fcvm.2023.1335572 PMID:WOS:001161679700001  637. Xu Z, Jia S, Huang N, Ma Y, Qin D, Dong B. Association between balance impairment and incidence of motoric cognitive risk syndrome in the China Health and Retirement Longitudinal Study. Journal of Nutrition, Health and Aging. 2025;29(3). doi: 10.1016/j.jnha.2024.100476  638. Xuekelati S, Maimaitiwusiman Z, Xiang H, Wumaer A, Bai X, Wang H. Handgrip strength: A simple and effective tool to recognize decreased intrinsic capacity in Chinese older adults. Exp Gerontol. 2024;196:112567. doi: 10.1016/j.exger.2024.112567 PMID:39236871  639. Yamada K, Iwata K, Yoshimura Y, Ota H, Oki Y, Mitani Y, et al. Activities of daily living limitation and functional decline during hospitalization predict 180-day readmission and mortality in older patients with pneumonia: A single-center, retrospective cohort study. Respiratory Medicine. 2024;234. doi: 10.1016/j.rmed.2024.107830  640. Yamaguchi R, Makino K, Katayama O, Yamagiwa D, Shimada H. Physical inactivity, depressive symptoms, and progression to sarcopenia in older adults: a 4-year longitudinal study. Journal of Nutrition, Health and Aging. 2025;29(3). doi: 10.1016/j.jnha.2024.100452  641. Yan M. The effect and mechanism of FKkBP5 on septiccardiomyopathy and myocardial microvascularendothelial cell permeability. Master's Degree, Shandong University. 2024. Available from:https://d.wanfangdata.com.cn/thesis/ChhUaGVzaXNOZXdTMjAyNDA5MjAxNTE3MjUSCFk0MzYzMjY4GghscW1xaXRrcA%3D%3D  642. Yan Y, Xu Y, Wang X, Wang Y, Huang C, Lin R, et al. The effect of multi-component exercise intervention in older people with Parkinson's disease and mild cognitive impairment: a randomized controlled study. Geriatric nursing (New York, NY). 2024;60:137‐45. doi: 10.1016/j.gerinurse.2024.08.028 PMID:CN-02751675  643. Yang J. Effects of vestibular activation trainingon motor function, language and cognition in children with global developmental delay. Master's Degree, Zhengzhou University. 2024. Available from:https://d.wanfangdata.com.cn/thesis/ChhUaGVzaXNOZXdTMjAyNDA5MjAxNTE3MjUSCFk0MzQ1ODg1GghscW1xaXRrcA%3D%3D  644. Yang J, Wang Y, Shi X, Liu Y, Ge S, Li S, et al. Prevalence of sarcopenic obesity among older adults in communities of China: A multicenter, cross-sectional study. Nutrition in Clinical Practice. 2024;39(6):1375-87. doi: 10.1002/ncp.11214  645. Yang L, Xu Y, Zhao H, Wang K, Zheng C. Longitudinal patterns of cognitive function and depression: insights from the China Health and Retirement Longitudinal Study. Journal of global health. 2025;15:04060. doi: 10.7189/jogh.15.04060  646. Yang S, Li J, Fu P, Sun Y, Liu Y, Zhou C. Bidirectional associations of grip strength-gait speed with mild cognitive impairment and specific cognitive abilities among older adults: A longitudinal analysis. Arch Gerontol Geriatr. 2025;131:105733. doi: 10.1016/j.archger.2024.105733 PMID:39742819  647. Yang X. Protective Effects and Mechanisms of Idebenone Derivatives against Oxidative Stress in Cardiomyocytes. Master's Degree, Guangdong Medical University. 2024. Available from:https://d.wanfangdata.com.cn/thesis/ChhUaGVzaXNOZXdTMjAyNDA5MjAxNTE3MjUSCUQwMzQ4NTQ4NhoIbHFtcWl0a3A%3D  648. Yang Y, Drake SA, Wang J, Shen GC, Miao H, Morgan RO, et al. Comparing total medical costs of surgical treatment versus nonoperative care for femoral neck fractures among Alzheimer's disease patients: A retrospective cohort study. Geriatric nursing (New York, NY). 2025;61:499-505. doi: 10.1016/j.gerinurse.2024.12.023  649. Yao Y. ALLEVIATING THE ANXIETY OF COLLEGE STUDENTS BY MUSIC DRAMA EDUCATION. Psychiatria Danubina. 2022;34:S866‐S7. doi: PMID:CN-02668775  650. Yao Z, Wang J, Zhang T, Ai H, Abdelrahman Z, Wu X, et al. Age, sex, and APOE gene-specific associations between dynapenic obesity and dementia in a large cohort. J Nutr Health Aging. 2024;28(8):100313. doi: 10.1016/j.jnha.2024.100313 PMID:38986174  651. Ye C, Chen G, Huang W, Liu Y. Association between skeletal muscle mass to visceral fat area ratio and depression: A cross-sectional study based on the National Health and Nutrition Examination Survey. Journal of Affective Disorders. 2025;372:314-23. doi: 10.1016/j.jad.2024.12.041 PMID:WOS:001388749600001  652. Yen YH, Yen FS, Ko FS, Wei JCC, Huang Y, Yu TS, et al. Microvascular disease and its association with dementia in patients with type 2 diabetes: A nationwide cohort study in Taiwan. Diabetes, Obesity and Metabolism. 2024;26(11):5399-407. doi: 10.1111/dom.15908  653. Yeung SSY, Ma SL, Wang X, Chen Y, Tsui SKW, Tang NLS, et al. Telomere length among Chinese aged 75+years. Gerontology. 2023;69(12):1414-23. doi: 10.1159/000534644 PMID:WOS:001095988500001  654. Yorozuya K, Nakashima D, Fujii K, Noritake K, Kubo Y, Tsubouchi Y, et al. Associations Between Cognitive Function and Muscle Quality Among Community-Dwelling Older Adults: A Cross-Sectional Study. Experimental aging research. 2025;51(2):150-61. doi: 10.1080/0361073X.2024.2334645  655. Yoshimura Y, Nagano F, Matsumoto A, Shimazu S, Shiraishi A, Kido Y, et al. Hemoglobin levels and cognitive trajectory: unveiling prognostic insights in post-stroke geriatric cohort. Journal of Stroke and Cerebrovascular Diseases. 2024;33(9). doi: 10.1016/j.jstrokecerebrovasdis.2024.107856  656. Yoshimura Y, Wakabayashi H, Nagano F, Matsumoto A, Shimazu S, Shiraishi A, et al. Coexistence of low body mass index and poor oral health negatively affects activities of daily living, swallowing, and cognition after stroke. Geriatrics and Gerontology International. 2024;24(10):1045-52. doi: 10.1111/ggi.14971  657. Yoshimura Y, Wakabayashi H, Nagano F, Matsumoto A, Shimazu S, Shiraishi A, et al. Dual burden of sarcopenia and impaired oral status on activities of daily living, cognition and swallowing outcomes in post-stroke patients. Archives of Gerontology and Geriatrics. 2025;129. doi: 10.1016/j.archger.2024.105648  658. Yoshimura Y, Wakabayashi H, Nagano F, Matsumoto A, Shimazu S, Shiraishi A, et al. Hyponatremia as a predictor of cognitive deterioration in hospitalized post-stroke patients. Journal of Clinical Neuroscience. 2024;124:115-21. doi: 10.1016/j.jocn.2024.04.027  659. You J, Guo Y, Wang YJ, Zhang Y, Wang HF, Wang LB, et al. Clinical trajectories preceding incident dementia up to 15 years before diagnosis: a large prospective cohort study. Molecular Psychiatry. 2024;29(10):3097-105. doi: 10.1038/s41380-024-02570-0  660. Yu L, Cao S, Song B, Hu Y. Predicting grip strength-related frailty in middle-aged and older Chinese adults using interpretable machine learning models: a prospective cohort study. Frontiers in public health. 2024;12:1489848. doi: 10.3389/fpubh.2024.1489848  661. Yuan H, Jiang Y, Li Y, Bi L, Zhu S. Development and validation of a nomogram for predicting motoric cognitive risk syndrome among community-dwelling older adults in China: a cross-sectional study. Frontiers in public health. 2024;12:1482931. doi: 10.3389/fpubh.2024.1482931  662. Zang L. Effect of acetaminophen on postoperative delirium inelderly patients undergoing hip replacement under general anesthesia. Master's Degree, Zhengzhou University. 2024. Available from:https://d.wanfangdata.com.cn/thesis/ChhUaGVzaXNOZXdTMjAyNDA5MjAxNTE3MjUSCFk0MzQ2ODkwGghscW1xaXRrcA%3D%3D  663. Zanotto T, Pradeep Kumar D, Golan D, Wilken J, Doniger GM, Zarif M, et al. Does cognitive performance explain the gap between physiological and perceived fall-risk in people with multiple sclerosis? Multiple Sclerosis and Related Disorders. 2025;95. doi: 10.1016/j.msard.2025.106322  664. Zeng N, Li C, Mei H, Wu S, Liu C, Wang X, et al. Bidirectional Association between Sarcopenia and Depressive Symptoms among Chinese Middle- and Older-Aged Adults: Longitudinal Observational Study. Brain Sciences. 2024;14(6). doi: 10.3390/brainsci14060593 PMID:WOS:001255040100001  665. Zeng Z, Hsu CL, van Schooten KS, Yang Y. Sex differences in the associations of accelerometer-determined physical activity with physical and cognitive function in older adults living in long-term care. Frontiers in public health. 2024;12:1446286. doi: 10.3389/fpubh.2024.1446286 PMID:CN-02780574  666. Zhai Y-J, Li F, Lin C-Y, Wu F, Qiu H-N, Li J-B, et al. The mediating role of body surface area-adjusted basal metabolic rate: effects of low muscle mass and central obesity on cognitive impairment in Chinese patients with type 2 diabetes mellitus. Frontiers in Endocrinology. 2025;15. doi: 10.3389/fendo.2024.1513035 PMID:WOS:001414703000001  667. Zhai YJ, Li F, Lin CY, Wu F, Qiu HN, Li JB, et al. The mediating role of body surface area-adjusted basal metabolic rate: effects of low muscle mass and central obesity on cognitive impairment in Chinese patients with type 2 diabetes mellitus. Frontiers in Endocrinology. 2024;15. doi: 10.3389/fendo.2024.1513035  668. Zhan QN, Chu WW, Guo Q, Zhao JL. The Association of β2-Microglobulin with Physical Performance in Chinese Hemodialysis Patients with and without Diabetes. Kidney360. 2024. doi: 10.34067/KID.0000000669  669. Zhang C, Feng X, Zhang X, Chen Y, Kong J, Lou Y. Research progress on the correlation between estrogen and estrogen receptor on postmenopausal sarcopenia. Frontiers in Endocrinology. 2024;15. doi: 10.3389/fendo.2024.1494972 PMID:WOS:001370090700001  670. Zhang H, Hu Z, Jiang S, Hao M, Li Y, Liu Y, et al. Social frailty and the incidence of motoric cognitive risk syndrome in older adults. Alzheimers Dement. 2024;20(4):2329-39. doi: 10.1002/alz.13696 PMID:38284799  671. Zhang J, Jia X, Li Y, Li H, Yang Q. The longitudinal bidirectional association between sarcopenia and cognitive function in community-dwelling older adults: Findings from the China Health and Retirement Longitudinal Study. J Glob Health. 2023;13:04182. doi: 10.7189/jogh.13.04182 PMID:38148730  672. Zhang J, Na X, Li Z, Ji JS, Li G, Yang H, et al. Sarcopenic obesity is part of obesity paradox in dementia development: evidence from a population-based cohort study. BMC Medicine. 2024;22(1). doi: 10.1186/s12916-024-03357-4  673. Zhang J, Tam WWS, Lu J, Chen J, Kusuyama J, Dong Y, et al. Cognitive Risk Stratification Score in Middle-aged and Older Adults with Type 2 Diabetes: a Cross-Sectional Study. The Journal of clinical endocrinology and metabolism. 2025. doi: 10.1210/clinem/dgaf063  674. Zhang Q. Quantitative assessment of motor symptoms in Parkinson's disease and its correlation with non-motor symptoms. Master's Degree, Shandong University. 2024. Available from:https://d.wanfangdata.com.cn/thesis/ChhUaGVzaXNOZXdTMjAyNDA5MjAxNTE3MjUSCFk0MzYyODM1GghscW1xaXRrcA%3D%3D  675. Zhang S, Otsuka R, Tange C, Nishita Y, Shimokata H, Satake S, et al. Implication of grip strength assessment for the management of body weight in disability prevention in older adults. Journal of Cachexia, Sarcopenia and Muscle. 2024;15(1):208-19. doi: 10.1002/jcsm.13396  676. Zhang X, Yang D, Luo J, Meng M, Chen S, Li X, et al. Determinants of sedentary behavior in community-dwelling older adults with type 2 diabetes based on the behavioral change wheel: a path analysis. BMC geriatrics. 2024;24(1):502. doi: 10.1186/s12877-024-05076-0  677. Zhang X-M. The association between sarcopenia index and cognitive function among Chinese older adults. Journal of Nutrition Health & Aging. 2024;28(9). doi: 10.1016/j.jnha.2024.100331 PMID:WOS:001347794700001  678. Zhang Y, Liu R, Wang W, Wu RJ, Dai WT, Zhang CL, et al. Association of Myostatin With Complications and Cognition in Lung Cancer Patients With Sarcopenia. J Surg Res. 2024;302:240-9. doi: 10.1016/j.jss.2024.07.054 PMID:39111127  679. Zhang Y, Wang R, Liu T, Wang R. Exercise as a Therapeutic Strategy for Obesity: Central and Peripheral Mechanisms. Metabolites. 2024;14(11). doi: 10.3390/metabo14110589 PMID:WOS:001365315400001  680. Zhang Y, Zhu R, Ge L, Zhang X, Tian D, Pan F, et al. Association of handgrip strength asymmetry and weakness with cognitive function: a nationally representative cohort study. Maturitas. 2024;187:108057. doi: 10.1016/j.maturitas.2024.108057 PMID:38908060  681. Zhao X, Yu J, Zhou Z. Separate and combined associations of obesity and handgrip strength with cognitive function in older adults: A national cross-sectional study in China. J Sports Sci. 2024;42(2):109-15. doi: 10.1080/02640414.2024.2321420 PMID:38388361  682. Zhao X, Zhang H, Yu J, Liu N. Independent and combined associations of handgrip strength and walking speed with cognitive function in older adults: evidence from a national cross-sectional study. Aging Ment Health. 2024;28(12):1659-66. doi: 10.1080/13607863.2024.2360018 PMID:38835194  683. Zhao X, Zuo M, Zhan F, Fan P, Liu S, Taylor M, et al. Cognition mediates the relationship between white matter hyperintensity and motor function in patients with cerebral small vessel disease: a cross-sectional study. Quantitative Imaging in Medicine and Surgery. 2024;14(10):7306-17. doi: 10.21037/qims-24-1058  684. Zhao Y, Nogueira MS, Chen Q, Dai Q, Cai Q, Wen W, et al. Association between F<sub>2</sub>-Isoprostane Metabolites and Weight Change in Older Women: A Longitudinal Analysis. Gerontology. 2024;70(2):134-42. doi: 10.1159/000534258 PMID:WOS:001162772700006  685. Zhou C, Peng J, Qian Z, Zhan L, Yuan J, Zha Y. Associations of dynapenic abdominal obesity and its components with cognitive impairment among hemodialysis patients. BMC geriatrics. 2025;25(1):107. doi: 10.1186/s12877-024-05580-3  686. Zhou L, Zhang Y, Ge M, Zhang G, Cheng R, Liu Y, et al. The associations of daytime napping and motoric cognitive risk syndrome: Findings from the China Health and Retirement Longitudinal Study. Experimental Gerontology. 2024;191. doi: 10.1016/j.exger.2024.112426  687. Zhu Y, Yin H, Zhong X, Zhang Q, Wang L, Lu R, et al. Exploring the mediating roles of depression and cognitive function in the association between sarcopenia and frailty: A Cox survival analysis approach. Journal of advanced research. 2024. doi: 10.1016/j.jare.2024.12.021  688. Zong H. Correlation Analysis and Mechanism Research BetweenCircadian Rhythm and Cognitive impairment in Parkinson's Disease. Master's Degree, Zhengzhou University. 2024. Available from:https://d.wanfangdata.com.cn/thesis/ChhUaGVzaXNOZXdTMjAyNDA5MjAxNTE3MjUSCFk0MzQ2NTA3GghscW1xaXRrcA%3D%3D  689. Chen F. A case of spastic pseudosclerosis. Clinical Misdiagnosis & Mistherapy. 2000;13(6):402. doi: 10.3969/j.issn.1002-3429.2000.06.083  690. 45th Annual SER Meeting. American Journal of Epidemiology. 2012;175. doi:  691. Singapore Health and Biomedical Congress, SHBC 2013. Annals of the Academy of Medicine Singapore. 2013;42:S1. doi:  692. Announcement for the 2014 Peking Union Medical College-Johns Hopkins Geriatric Forum. Medical Journal of Peking Union Medical College Hospital. 2014;(3):342-.  693. Novel "Molecular Tweezers" Show Promise in Treating Various Diseases and Preventing Harmful Protein Aggregation. Technology and Market. 2015;(7):4-.  694. Abstracts of the 11th International Congress of the European Union Geriatric Medicine Society - Geriatric Medicine for Future Europeans - Successful Aging Creates New Challenges. European Geriatric Medicine. 2015;6. doi:  695. Peking Union Medical College-Johns Hopkins Geriatric Forum. Chinese Journal of Multiple Organ Diseases in the Elderly. 2016;15(08):556.  696. Identifying low muscle mass in patients with hip fracture: validation of bioelectrical impedance analysis and anthropometry compared to dual energy X-ray absorptiometry. Journal of nutrition, health and aging 20 (7) (pp 685-690), 2016 Date of publication: 01 jul 2016. 2016. doi: 10.1007/s12603-016-0686-1 PMID:CN-01194909  697. Does hand and foot tremor indicate Parkinson's disease? Science and Technology of Family. 2017;(3):41.  698. Winter Conference 2016: Diet, Nutrition and Mental Health and Wellbeing. Proceedings of the Nutrition Society. 2017;76(OCE1). doi:  699. British Geriatrics Society Communications to the Autumn Meeting 2017. Age and Ageing. 2018;47. doi:  700. Table of Contents for Practical Geriatric Medicine, 2019, Volume 33 (Issues 1-12). Practical Geriatrics. 2019;33(12).  701. Abbreviations That Can Be Used Directly in This Issue (I). Chinese Journal of Modern Nursing. 2019;25(27):3462.  702. Over 50, how to exercise better? Care Health. 2019;(8):32-5.  703. Abstracts of the 15th International Congress of the European Geriatric Medicine Society. European Geriatric Medicine. 2019;10. doi:  704. Repeated infusions of ketamine show cumulative benefits for depression. Brown university psychopharmacology update. 2019;30(7):3‐. doi: 10.1002/pu.30446 PMID:CN-02417291  705. Chinese Journal of Geriatric Multi-organ Diseases, volume 19, 2020. Chinese Journal of Multiple Organ Diseases in the Elderly. 2020;19(12):961-78.  706. Winter Conference Live 2020: Micronutrient Malnutrition Across the Life Course, Sarcopenia and Frailty. Proceedings of the Nutrition Society. 2021;80(OCE1). doi:  707. Chen C, Liu H, Lin Y, Fan F, Wang Q. Association between sarcopenia and post-stroke cognitive impairment in elderly patients with first-time acute minor ischemic stroke. Chinese Journal of Geriatrics. 2021;40(4).  708. Clinical Guidelines for Prevention and Treatment of Type 2 Diabetes Mellitus in the Elderly in China (2022 Edition). Chin J Diabetes. 2022;30(01):2-51.  709. “Food and medicine continuum”-Why we should promote cross-cultural communication between the global East and West. Chinese Traditional and Herbal Drugs（English version）. 2022;14(1). doi:  710. 65 centimeters for a brisk walk. Builders' Monthly. 2022;43(11):64. doi: 10.3969/j.issn.1002-3232.2022.11.028  711. Clinical characteristics with inflammation profiling of long COVID and association with 1-year recovery following hospitalisation in the UK: a prospective observational study. Lancet Respir Med. 2022;10(8):761-75. doi: 10.1016/s2213-2600(22)00127-8 PMID:35472304  712. Liang X, Fang M, Yang Y, Zhu T, Jiang H. Effect of Functional Electrical Stimulation Combined with Circulatory System on Limb Function,Balance Ability and Cognitive Function in Elderly Patients with Cerebral-stroke Sarcopenia. Progress in Modern Biomedicine. 2022;22(9).  713. Development and Application of Interactive Assessment and Intervention Technology for Elderly Syndrome Based on Mobile Internet. China Science and Technology Achievements. 2023;24(8):7-10. doi: 10.3772/j.issn.1009-5659.2023.08.007  714. Irish Gerontological Society 70th Annual and Scientific Meeting - Looking to the Years Ahead: Europe's fastest growing older population. Age and Ageing. 2023;52. doi:  715. Liwaidin A, Wusuer D. Clinical Analysis of Acute Demyelinating Encephalomyelitis (ADEM). Xinjiang Medical Journal. 2013;43(10):74-5. doi: 10.3969/j.issn.1001-5183.2013.10.026  716. Abdukerim A, Yuluduz. Obstructive Sleep Apnea-Hypopnea Syndrome and Laryngopharyngeal Reflux in Children. International Journal of Otolaryngology-Head and Neck Surgery. 2015;39(5):260-3. doi: 10.3760/cma.j.issn.1673-4106.2015.05.004  717. Bai H, Wu Q. Pedigree of a Family with Familial Senile Dementia. Chinese Journal of Geriatrics. 2005;24(5):381. doi: 10.3760/j:issn:0254-9026.2005.05.021  718. Bai J, Xu Y, Zhang Y, Wang R. Clinical Research on Galantamine for the Treatment of Vascular Dementia. China Health Care & Nutrition. 2013;23(6):2861-2. doi: 10.3969/j.issn.1004-7484(x).2013.06.002  719. Bao Q, Yan Y, Ding X. Relationship between sarcopenia and levels of homocysteine, irisin and malnutrition-inflammation score in maintenance hemodialysis patients Chinese Journal of Blood Purification. 2022;21(10):744-8.  720. Bao J. Common Characteristics of Nonagenarians. Special Health. 2012;(14):88.  721. Bao Y. Clinical Study on Kangzhen Zhijing No. 1 Granules in the Treatment of 30 Cases of Parkinson's Disease. Journal of Traditional Chinese Medicine. 2001;42(5):312-3. doi: 10.3321/j.issn:1001-1668.2001.05.031  722. Bi Z, Cao Y, Liu C, Gui M, Lin J, Bu B. Myasthenia gravis associated with autoimmune encephalitis:clinical features of two cases and a literature review. Chinese Journal of Neuroimmunology and Neurology. 2021;28(4):293-6. doi: 10.3969/j.issn.1006-2963.2021.04.005  723. Cai L. Prevention and treatment of constipation in the elderly. Health literature. 2021;22(8):50.  724. Cai R, Chen M, Qi H, Zhang Z. Analysis on Application of Herbal Compounds Containing Renshen(Ginseng)in Treating Geriatric Syndromes. Acta Chinese Medicine. 2020;35(8).  725. Cai S, Gao D. Improvement of Lance-Adams Syndrome with Perampanel in a Case Report. Special Health. 2022;(7):181.  726. Cai X. Acupuncture Can Effectively Treat Parkinson's Disease. Family & Traditional Chinese Medicine. 2015;22(5):42-3.  727. Cao Y. Parkinson's care. Diet Health. 2016;3(10):119-20.  728. Cao F, Ma L, Li J, Li J. Impact of Dexmedetomidine-Assisted General Anesthesia on Early Postoperative Cognitive Function in Elderly Patients with Colorectal Cancer. Shandong Medical Journal. 2015;(40):76-8. doi: 10.3969/j.issn.1002-266X.2015.40.030  729. Cao G. Sweet as sugar health treasure-monk fruit. Cooking Knowledge. 2003;(3):50.  730. Cao H. Study Progress of Multiple Sclerosis. Medical Recapitulate. 2007;13(21):1636-8. doi: 10.3969/j.issn.1006-2084.2007.21.016  731. Cao L, Shi Y, Pang G. Research Progress on the Clinical Application of Glutamine in Neurological Diseases. Medical & Pharmaceutical Journal of Chinese People's Liberation Army. 2020;32(6):113-6. doi: 10.3969/j.issn.2095-140X.2020.06.025  732. Zeng J, Tian Y, Wang Y. Clinical Experience in the Diagnosis and Treatment of 10 Cases of Pediatric Pentafluorophenol Intoxication. Journal of Pediatric Pharmacy. 2015;21(1):64,后插1. doi: 10.13407/j.cnki.jpp.1672-108X.2015.01.022  733. Zeng Y, Xue X, Yu M, Lan T, Chen Y. Cellular Behavioromics: A Research Strategy for the Scientization of Traditional Chinese Medicine from a Systems Theory Perspective. Chinese Journal of Pharmacology and Toxicology. 2023;37(z1):53-6. doi: 10.3867/j.issn.1000-3002.2023.z1.066  734. Chang F, Bai T, Lv X, Hu Y, Wang M. Research Progress on Mongolian Medicine for Senile Dementia and Cognitive Impairment-Related Diseases. Acta Neuropharmacologica. 2017;7(2):33-4. doi: 10.3969/j.issn.2095-1396.2017.02.039  735. Shen D, Zou P, Hou B, Yang X, Liu M, Cui L. Comparative study on grey matter structure MRI between amyotrophic lateral sclerosis patients with limb-onset and bulbar-onset. Chinese Journal of Contemporary Neurology and Neurosurgery. 2022;22(7):601-7. doi: 10.3969/j.issn.1672-6731.2022.07.009  736. Shen L, Zhao G, Tang B, Liu X, Yan X, Hu Z, et al. Clinical Features and Maspardin Gene Mutation Analysis in Six Cases of Hereditary Spastic Paraplegia with Thin Corpus Callosum. Chinese Journal of Neurology. 2005;38(8):521-2. doi: 10.3760/j.issn:1006-7876.2005.08.016  737. Shen M, Tao Q, Cao W, Zhai J. Investigation and analysis of sarcopenia cognitive status of the community nurses in Jiading District. Shanghai Medical & Pharmaceutical Journal. 2022;43(10).  738. Shen X, Yu X, Sheng S. Study on Sterilization Methods for Huperzia serrata Shoot Tips in Tissue Culture. China Journal of Chinese Materia Medica. 2002;27(6):458-9. doi: 10.3321/j.issn:1001-5302.2002.06.020  739. Chen A, Chen L, Yuan W, Qiu J. Case Analysis of Rehabilitation Therapy for Multiple Sclerosis Patients Based on the Perceived Exertion Scale. Chinese Journal of Rehabilitation Medicine. 2017;32(1):105-9. doi: 10.3969/j.issn.1001-1242.2017.01.025  740. Chen C. Research progress of correlation between Parkinson's disease and sarcopenia. Journal of Apoplexy and Nervous Diseases. 2022;39(4):375-8. doi: 10.19845/j.cnki.zfysjjbzz.2022.0098  741. Chen C, Li N, Peng R. Research progress of correlation between Parkinson's disease and sarcopenia. Journal of Apoplexy and Nervous Diseases. 2022;39(4).  742. Chen C. Clinical analysis of 25 cases of upper gastrointestinal adverse reactions caused by non-steroidal anti-inflammatory drugs. Anhui Journal of Preventive Medicine. 2007;13(1):80-封3.  743. Chen D, Chen J, Du W, Jin Y. A Case Report of Cerebral Amyloid Angiopathy Presenting with Progressive Supranuclear Palsy-like Symptoms. Stroke and Nervous Diseases. 2014;21(4):247-8. doi: 10.3969/j.issn.1007-0478.2014.04.019  744. Chen G, Fu F, Liang J, Huang H, Li L, Lin L. Assessment implement and its related factors in type 2 diabetic patients with mild cognitive impairment. Chinese Journal of Endocrinology and Metabolism. 2010;26(1):22-6. doi: 10.3760/cma.j.issn.1000-6699.2010.01.007  745. Chen J, Bai J. Relationship between physical activity and complications in patients with chronic obstructive pulmonary disease:a hospital-based survey. Chinese Journal of Public Health. 2021;37(11):1687-90. doi: 10.11847/zgggws1121310  746. Chen J. Home rehabilitation nursing method of tremor paralysis. Family & Traditional Chinese Medicine. 2018;25(1):54-5.  747. Chen J, Li Y. A case of senile mixed neurosyphilis. Journal of Military Surgeon in Southwest China. 2016;18(4):391-2. doi: 10.3969/j.issn.1672-7193.2016.04.036  748. Chen L, Yi F, Chen L, Qiang W. Minimally invasive percutaneous nephrolithotomy combined with flexible cystoscopy for the treatment of complicated renal calculi and its impact on patients' quality of life. China Medical Herald. 2018;15(33):51-5.  749. Chen L, Zhang B, Chen L. Effect of early intervention program of integrated Chinese and Western medicine on cognition and motor function of patients with moderate and severe craniocerebral injury during rehabilitation. Guiding Journal of Traditional Chinese Medicine and Pharmacy. 2014;20(16):73-5.  750. Chen L, Xi H, Huang H, Zhang F, Liu Y, Chen D, et al. Clinical practice of brain key points for neural network restoration. Chinese Journal of Tissue Engineering Research. 2011;15(6):1127-31. doi: 10.3969/j.issn.1673-8225.2011.06.040  751. Chen M. Two Methods of Enteral Nutrition Support by Nasal Feeding and Analysis of Complications in Patients with Alzheimer's Disease and Dysphagia. Systems Medicine. 2021;6(9):57-9. doi: 10.19368/j.cnki.2096-1782.2021.09.057  752. Chen M, Chen X. Rehabilitation nursing status of "mask face" in patients with Parkinson's disease. Journal of Qilu Nursing. 2018;24(19):95-7. doi: 10.3969/j.issn.1006-7256.2018.19.036  753. Chen M, Sun J, Chen Y, Bai H, Li Z, Gu J, et al. The prevalence of sarcopenia and related risk factors in the ambulatory elderly in a nursing home in Shanghai. Chinese Journal of Clinical Healthcare. 2023;26(4).  754. Chen N, Dai L, Jiang Y, Wu Y. Pathogenic Role of UPR (Unfolded Protein Response) Among Hereditary Leukoencephalopathy and Neurodegenerative Disorders After Endoplasmic Reticulum Stress. Progress in Biochemistry and Biophysics. 2012;39(8):764-70. doi: 10.3724/sp.J.1206.2012.00097  755. Chen S, Qiu T. A comparative study of Aripiprazole and Quetiapine in treatment of geriatric schizophrenic patients. Medical Journal of Chinese People's Health. 2012;24(15):1826-7. doi: 10.3969/j.issn.1672-0369.2012.15.013  756. Chen W, Zhang L, Gu Z. Research advancement in aging characteristics and related effects of regular physical activity. Journal of Shandong Sport University. 2011;27(01):38-43. doi: 10.14104/j.cnki.1006-2076.2011.01.009  757. Chen W. Multi-sensory activity room for the elderly. Disability in China. 2013;(9):56.  758. Chen Y, Zhang Y. Research Progress on Treatment and Prognosis of PCDH19-Related Epilepsy. Chinese Journal of Practical Pediatrics. 2022;37(7):541-5. doi: 10.19538/j.ek2022070615  759. Chen Y, Gong A, Gao Y, Yu S, Yang Z. Application of electron microscope in experimental acupuncture. China Medical Herald. 2012;9(22):31-3. doi: 10.3969/j.issn.1673-7210.2012.22.012  760. Chen Y, Lv Y. Research progress on the correlation between sarcopenia and cognitive impairment. Practical Geriatrics. 2020;34(02):111-6.  761. Chen C, Liu H, Lin Y, Fan F, Wang Q. Association between sarcopenia and post-stroke cognitive impairment in elderly patients with first-time acute minor ischemic stroke. Chinese Journal of Geriatrics. 2021;40(4).  762. Chen C, Xun F, Meng X, Lv J. The Effects of Selenium on Human Health and Diseases and the Scientifically Supplementation of Selenium to Human Bodies. Journal of Liaoning University(Natural Science Edition). 2016;43(2):155-68. doi: 10.3969/j.issn.1000-5846.2016.02.011  763. Chen Z, Teng Z, Qiao T. Discussion on the use of statins for primary prevention of atherosclerosis. Chinese Journal of Vascular Surgery(Electronic Version). 2020;12(1):66-9. doi: 10.3969/j.issn.1674-7429.2020.01.019  764. Chen Z, Ma L, Lou X, Wang Y. Voxel-based morphometry and voxel-based diffusion tensor analysis in amyotrophic lateral sclerosis. Chinese Journal of Radiology. 2010;44(4):354-60. doi: 10.3760/cma.j.issn.1005-1201.2010.04.005  765. Chen Z, Wang F. A Case of Bilateral Anterior Cerebral Artery Infarction with Mutism and Left Hand Apraxia. The Journal of Practical Medicine. 2005;21(12):1361. doi: 10.3969/j.issn.1006-5725.2005.12.078  766. Chen Z, Huang G, Xu B. Clinical diagnosis and treatment of simple acute infarction of corpus callosum and literature review. Chinese Journal of Cerebrovascular Diseases. 2020;17(4):192-8. doi: 10.3969/j.issn.1672-5921.2020.04.005  767. Chen Z, Huang G, Xu B. Clinical characteristics of acute simple corpus callosum infarction. Journal of Brain and Nervous Diseases. 2020;28(7):414-8.  768. Cheng Y, Gan D, Ye Ya. Research progress of correlation between blood ammonia level and hepatic encephalopathy. Chinese Hepatology. 2019;24(10):1202-4. doi: 10.14000/j.cnki.issn.1008-1704.2019.10.039  769. Yu H, Qiu J. Facing on the Challenges of Aging Society Research Review of Senior Citizen's Physical Activity. Journal of Beijing Sport University. 2013;36(8).  770. Chu Y, Li W. A Case Report of Amyotrophic Lateral Sclerosis-Dementia Complex. Journal of Brain and Nervous Diseases. 2001;9(3):180. doi: 10.3969/j.issn.1006-351X.2001.03.022  771. Cui C, Zhu Q, Gao X. Clinical effect of acupuncture combined with rehabilitation on motor aphasia after stroke. China's Naturopathy. 2016;24(9):28-9.  772. Cui W, Wei Y, Liang B, Chen G, Pei L. Effects of chronic disease comorbidity on the risk of possible sarcopenia among Chinese middle-aged and older adults. Chinese Journal of Osteoporosis and Bone Mineral Research. 2022;15(6).  773. Cui Y, Zhang Y, Li Q, Zhang L, Gu Y, Wang Z, et al. Correlation Analysis on AD Symptoms of Traditional Chinese Medicine with the Theory of the Kidney Storing Essence. Chinese Archives of Traditional Chinese Medicine. 2015;33(6):1356-9. doi: 10.13193/j.issn.1673-7717.2015.06.024  774. Dai L, Ding C, Fang F. Progress in diagnosis and treatment of dopa responsive dystonia. Chinese Journal of Evidence-Based Pediatrics. 2019;14(5):395-400. doi: 10.3969/j.issn.1673-5501.2019.05.016  775. Dai Y, Huo Y. Observation on the effect of sarcopenia training combined intervention in elderly patients with mild acute ischemic stroke. Chinese Journal of Convalescent Medicine. 2022;31(02):182-4. doi: 10.13517/j.cnki.ccm.2022.02.021  776. Dai X, Wu J, Dong Q. Clinical analysis on 11 cases of Creutzfeidt-Jakob disease. Journal of Apoplexy and Nervous Diseases. 2008;25(2):208-10. doi: 10.3969/j.issn.1003-2754.2008.02.024  777. Dai Y, Pang X, Liu Y, Zhang W. Creutzfeldt-jakob disease and sleep disorders. Journal of Apoplexy and Nervous Diseases. 2018;35(6):574-6.  778. Dai Z, Yang Z, Wang L, Ma W, Huang Y, Lu F, et al. Cardiovascular complications of Kearns-Sayre syndrome (report of 1 case). Journal of Nanjing Medical University(Natural Sciences). 2008;28(7):953-5.  779. Shan Y, Lu X, Lv S, Zhao S. Antagonistic effect of Glonium bromide combined with Neostigmine on neurocognitive impairment caused by muscle relaxations after laparoscopic rectal cancer radical surgery in elderly patients. Shandong Medical Journal. 2023;63(2):67-70. doi: 10.3969/j.issn.1002-266X.2023.02.017  780. Deng H. Osteocalcin and age-related diseases. Practical Geriatrics. 2020;34(1).  781. Deng X, Zhang P, Song L. A case of electrical storm after acute myocardial infarction. Chinese Journal of Cardiovascular Medicine. 2012;17(6):464-5. doi: 10.3969/j.issn.1007-5410.2012.06.020  782. Deng Y, Lou Y, Zhong M, Deng L. Clinical study of memantine combined with antidepressants in the treatment of AD with depression and anxiety and agitation. China Journal of Pharmaceutical Economics. 2018;13(8):48-51. doi: 10.12010/j.issn.1673-5846.2018.08.013  783. Ding G. Prevention of Alzheimer's Disease (Part 2). Health for the Elderly and Middle-Aged. 2022;(2):24-5.  784. Ding J. Gastrodia planting need to pay attention to several problems. Beijing Agriculture. 2004;(12):14. doi: 10.3969/j.issn.1000-6966.2004.12.020  785. Ding L, Zhu M. Application of oral nutritional supplement therapy in elderly patients. Food and Nutrition in China. 2017;23(09):69-73.  786. Ding M, Zhang Y, Wang X, Kong Q. Family analysis and literature review of generation aggravated spinocerebellar ataxia type 2. Journal of Apoplexy and Nervous Diseases. 2020;37(2):158-60.  787. Ding S. Care of patients with Parkinson's disease. World Health Digest. 2011;8(4):287-8. doi: 10.3969/j.issn.1672-5085.2011.04.291  788. Ding S, Wu S, Cui Y, Zhang Y, Bi X. The safety and efficacy of aricept in treatment of patients with vascular dementia. Chinese Journal of Geriatric Heart Brain and Vessel Diseases. 2003;5(6):395-6. doi: 10.3969/j.issn.1009-0126.2003.06.011  789. Ding S, Wu S, Zhang Y, Bi X, Cui Y. Clinical Observations on efficacy and Safety of Aricept in Treatment of Vascular Dementia. Journal of Neurology and Neurorehabilitation. 2004;1(1):1-3. doi: 10.3969/j.issn.1672-7061.2004.01.003  790. Ding X, Su H. Nursing for patients with Parkinson's disease during convalescence. Chinese Journal of Convalescent Medicine. 2009;18(11):992-. doi: 10.3969/j.issn.1005-619X.2009.11.028  791. Liu X, Yue J, Qiao R, Hou L, Dong B. The role of intestinal flora in the pathogenesis of sarcopenia. Practical Geriatrics. 2019;33(9).  792. Dong C, Lu A, Cao B, Hu H. One case of early adult-onset dentatorubral-pallidoluysian atrophy with an onset of epilepsy. Chinese Journal of Neurology. 2021;54(4):384-7. doi: 10.3760/cma.j.cn113694-20201223-00996  793. Dong H, Zhu H, Ma C, Rong J, Hu G, Li H. Progress in the comparative study of intervention effect and mechanism of centripetal and centrifugal exercise in functional rehabilitation of patients with hemiplegia. Chinese Journal of Rehabilitation Medicine. 2023;38(4):556-61. doi: 10.3969/j.issn.1001-1242.2023.04.023  794. Dong H, Yi W, Zhang B, Yang H. Effect of anti-anxiety intervention in coronary heart disease patients with anxiety disorder after interventional therapy. Chinese Journal of Rehabilitation. 2005;9(32):23-5. doi: 10.3321/j.issn:1673-8225.2005.32.011  795. Dong Z, Shi H, Zhang S, Feng L. Potential effects of dietary structure on health of elderly population. Chinese Journal of Health Education. 2020;36(12):1099-102. doi: 10.16168/j.cnki.issn.1002-9982.2020.12.008  796. Dong Z, Zhang H. Risk factors associated with sarcopenia among patients on maintenance hemodialysis. Journal of Nursing Science. 2018;33(09):20-4.  797. Du P, Wang J, Wang C, Wang Y. A case of borderline encephalitis. Clinical Focus. 2014;(11):1313-4. doi: 10.3969/j.issn.1004-583X.2014.11.035  798. Du T, Miao Y, Liu X, Zhang Q. Relationship between obstructive sleep apnea and sarcopenia in the elderly. Chinese Journal of Geriatrics. 2019;38(3).  799. Du X, Zhang H, Guo G. Research progress of sarcopenia in maintenance hemodialysis patients. Chinese Nursing Research. 2021;35(07):1194-8.  800. Duan H, Huo J, Chen F, Li L, Pan X, Cheng X, et al. Application of pathway nursing combined with multimodal exercise intervention in patients with tumor-associated sarcopenia under multi-disciplinary team. Chinese Journal of Modern Nursing. 2023;29(17).  801. Fan W, Xu Y, Huang Y. Is liquor good or bad for your health? Liquor-Making Science & Technology. 2014;(11):1-5. doi: 10.13746/j.njkj.2014.0245  802. Fang W, Wang G, Ma J, Cui L, Liu L, Ma J. Cognitive frailty and its influencing factors in elderly patients with persistent atrial fibrillation. Journal of Navy Medicine. 2023;44(9):907-10. doi: 10.3969/j.issn.1009-0754.2023.09.008  803. Feng F, Zhang H, Wang L. Progressive subcortical gliosis: neurodegenerative dementia should not be ignored. Chinese Journal of Contemporary Neurology and Neurosurgery. 2017;17(8):626-8. doi: 10.3969/j.issn.1672-6731.2017.08.012  804. Feng S, Shi M. Progress of clinical research on non-lipid-lowering effects of statins. China Pharmaceuticals. 2001;10(12):1-3. doi: 10.3969/j.issn.1006-4931.2001.12.001  805. Fu G, Dong N, Yang D. A family with creatine deficiency syndrome due to SLC6A8 gene novel mutation. Chinese Journal of Applied Clinical Pediatrics. 2020;35(20):1591-3. doi: 10.3760/cma.j.cn101070-20190928-00929  806. Fu Y, Ma J, Chen S. Progress of the relationship between serum uric acid and neurodegenerative diseases. Chinese Journal of Contemporary Neurology and Neurosurgery. 2018;18(3):204-12. doi: 10.3969/j.issn.1672-6731.2018.03.010  807. Fu Y, Du L, Zhu X. Anesthesia and perioperative management of a patient with multiple system atrophy undergoing artificial femoral head replacement: a case report. Chinese Journal of Anesthesiology. 2022;42(2):249-50. doi: 10.3760/cma.j.cn131073.20211130.00230  808. Fu L, Zhao H, Zhang X. Standardized construction of geriatric disease sample bank. Chinese Journal of Gerontology. 2016;36(6).  809. Gao A, Liu X, Lv J, Zhang Y, Guo L, He J, et al. Effect Evaluation of Diet and Exercises Mode Adjustment in Elderly Patients with Sarcopenia. Chinese Medical Record. 2019;(6).  810. Gao B, Bian Y. Effects of aerobic exercise on muscle decay, balance ability and cognitive function in elderly women. Medical Journal of National Defending Forces in Southwest China. 2017;27(12):1337-9.  811. Gao C, Yu P. The importance of the prevention and management of sarcopenia in the elderly. Chinese Journal of Clinical Healthcare. 2021;24(4).  812. Gao H, Guan Y, Zhang C, Liu B, Chen Y, Zhou F. Expression of Synaptophysin in the hippocampus of ALS transgenic mice. Chinese Journal of Histochemistry and Cytochemistry. 2013;22(4):286-9. doi: 10.3870/zgzzhx.2013.04.003  813. Gao H, Song M, Zhao S, Gao Y. Research status on cognition and assessment of senile sarcopenia. Chinese Journal of Modern Nursing. 2021;27(7).  814. Gao J. Tiapride versus risperidone in treatment of Alzheimer disease. Journal of Modern Clinical Medicine. 2017;43(1):42-4. doi: 10.11851/j.issn.1673-1557.2017.01.013  815. Gao J, Zhang J, Liang F, Chen Y, Xing L. Evaluation of intervention measures based on CGA in the treatment of senile sarcoenia. Journal of Modern Medicine & Health. 2023;39(12):2020-3+7.  816. Gao J, Wang L, Xue Y. Parkinson's disease is accompanied by dementia. Academic Journal of Chinese PLA Medical. 1999;20(4):310-2. doi: 10.3969/j.issn.1005-1139.1999.04.026  817. Gao S, Dong Y. Advances in research of frailty in elderly surgical patients. Chinese Journal of Clinicians(Electronic Edition). 2023;17(3):343-8. doi: 10.3877/cma.j.issn.1674-0785.2023.03.019  818. Gao W, Guo Y. Progress of cystatin C and cerebral small vessel disease. Journal of Neuroscience and Mental Health. 2017;17(8):596-9. doi: 10.3969/j.issn.1009-6574.2017.08.017  819. Gao Y, Liao L, Ying X. A resting-state fMRI comparative study on bladder storage function between normal volunteers and neurogenic overactive bladder. Chinese Journal of Urology. 2021;42(6):430-5. doi: 10.3760/cma.j.cn112330-20210414-00194  820. Geng Y, Li C, Li H, Wang M, Kang H. Clinical characteristics analysis of autoimmune encephalitis with epileptic seizure. Chinese Journal of Contemporary Neurology and Neurosurgery. 2023;23(3):205-13. doi: 10.3969/j.issn.1672-6731.2023.03.009  821. Gong W, Fan J, Tong F, Ye Z. Paired observation of long-term indwelling catheter and arterio-venous fistula for quality of life assessment in hemodialysis patients. Chinese Journal of Postgraduates of Medicine. 2010;33(18):11-4. doi: 10.3760/cma.j.issn.1673-4904.2010.18.005  822. Gong N. Influence of rehabilitation nursing on motor function recovery of stroke patients with hemiplegia. Clinical Journal of Diabetes World. 2020;17(10):269.  823. Gong Y, Sun J, Xiao S. Observation of systematic health education alleviating depression in patients with acute myocardial infarction. Practical Pharmacy and Clinical Remedies. 2003;6(4):191-. doi: 10.3969/j.issn.1673-0070.2003.04.017  824. Gong Y, Jiang N, Zhu H, Fu X, Li T, Deng W. Analysis of the Characteristics of Outpatients in Neurological Department in Different Periods. West China Medical Journal. 2015;30(9):1605-8. doi: 10.7507/1002-0179.20150461  825. Baha G, Luo R, Du X. Analysis of causes of misdiagnosis and misdiagnosis in 80 cases of acute myocardial infarction in the aged. Shandong Medical Journal. 2010;50(32):109. doi: 10.3969/j.issn.1002-266X.2010.32.062  826. Chen W, Zhang L, Gu Z. Research advancement in aging characteristics and related effects of regular physical activity. Journal of Shandong Sport University. 2011;27(1).  827. Gu Y, Miao J, Hu X, Shao R, Wang L, Yin Q. Application of comprehensive geriatric assessment in screening therisk factors of sarcopenia in octogenarians. Journal of Xuzhou Medical University. 2023;43(01):7-13.  828. Guo J, Huang L, Yang Y. Research progress of cognitive decline. Chinese Journal of Health Education. 2019;35(11):1025-8. doi: 10.16168/j.cnki.issn.1002-9982.2019.11.014  829. Guo P, Wang X, Wang D. Advances in the role of peroxisome proliferator-activated receptor gamma in neurodegenerative diseases. Progress in Physiological Sciences. 2015;(5):379-82.  830. Guo S, Li C. Progressive supranuclear ophthalmoplegia (report of 1 case). Stroke and Nervous Diseases. 2017;24(3):246-8. doi: 10.3969/j.issn.1007-0478.2017.03.020  831. Guo T, Yang J, Wu M, Guo Z. Impact of e-sports activity on adolescents' health, and rehabilitation interventions:a systematic review. Chinese Journal of Rehabilitation Theory and Practice. 2022;28(8):879-88. doi: 10.3969/j.issn.1006⁃9771.2022.08.002  832. Guo X, Lei K, Liang Y. Clinical effect of microsurgery in the treatment of glioma. China Clinical Practical Medicine. 2020;11(6):36-9. doi: 10.3760/cma.j.cn115570-20200508.00618  833. Han Q, Huang C. Analysis of misdiagnosis and imaging characteristics of elderly patients with stroke-like reaction hypoglycemia. Contemporary Medicine. 2022;28(3):148-50. doi: 10.3969/j.issn.1009-4393.2022.03.053  834. Han Z, Zhang Y. Dementia syndrome is common in old age. Shandong Medical Journal. 2003;43(25):50-1. doi: 10.3969/j.issn.1002-266X.2003.25.048  835. Hao D, Li F. Research progress on the correlation between frailty and cognitive impairment. Journal of Capital Medical University. 2017;38(03):406-10.  836. He C. The third part is about neurotrophic factor's neural repair function and its mechanism. Neuroscience Bulletin. 2002;18(1):466-71.  837. He J, Li X, Zhong M, Zou W, Zhao Q, Zou Q, et al. Voxel-based morphological comparison of gray matter between primary Parkinson's disease and healthy elderly patients. Journal of Practical Medical Imaging. 2015;16(3):260-2. doi: 10.16106/j.cnki.cn14-1281/r.2015.03.028  838. He W, Wang Y. Pharmacological research progress of dexmedetomidine in perioperative organ protection. Tianjin Pharmacy. 2020;32(3):64-7. doi: 10.3969/j.issn.1006-5687.2020.03.022  839. He X, Du M, Li L. Efficacy and safety of statins in primary prevention of cardiovascular events in elderly patients. Chinese Journal of Geriatric Heart Brain and Vessel Diseases. 2018;20(7):681-5. doi: 10.3969/j.issn.1009-0126.2018.07.003  840. He X, Jin Z. Advances in the application and safety of androgens in androgenic diseases. National Medical Journal of China. 2012;92(42):3020-2. doi: 10.3760/cma.j.issn.0376-2491.2012.42.020  841. He F, Ye J, Xu W. Clinical characteristics of leucine-rich glioma-inactivated protein 1 antibody-associated encephalitis. Chinese Journal of Neurology. 2016;49(1):26-9. doi: 10.3760/cma.j.issn.1006-7876.2016.01.006  842. He Y, Wang R, Ma Q, Pang Y, Qiang J, Chi A. Study on the recovery before and after exercise intervention of middle school students with chronic fatigue Syndrome based on Bodyguard. Youth Sport. 2020;(10):47-8,2. doi: 10.3969/j.issn.2095-4581.2020.10.012  843. Hu G, Jin L, Yuan N, Wang Z, Chen Z, Li J, et al. Clinical and electrophysiological features of patients with coexistence of epilepsy and narcolepsy. Chinese Journal of Neurology. 2021;54(6):560-6. doi: 10.3760/cma.j.cn113694-20201231-01023  844. Hu J, Feng S. Research Progress in Genetic Mechanisms of Fibronectin Type Ⅲ Domain-containing Protein 5/Irisin. Medical Recapitulate. 2018;24(9).  845. Hu L, Wu C, Yan W, Zhou H. Application of ultrasound-guided obturator nerve block combined with laryngeal mask general anesthesia in elderly patients undergoing TURBt. Chongqing Medical Journal. 2021;50(23):4071-5. doi: 10.3969/j.issn.1671-8348.2021.23.024  846. Hu T, Cao H. Progress in the pathogenesis of non-skeletal muscle system dysfunction in myasthenia gravis patients. Shandong Medical Journal. 2022;62(18):104-7. doi: 10.3969/j.issn.1002-266X.2022.18.026  847. Hu X. Nursing experience of senile constipation. Health Guide. 2017;(39):95. doi: 10.3969/j.issn.1006-6845.2017.39.090  848. Hu X, Li Y, Gao T, Li X, Wang X, Hou L. Research Progress of the Influence of Caloric Restriction on Senility and Geriatric Diseases. International Journal of Geriatrics. 2023;44(4).  849. Hu X, Dong J, Liu Y, Zheng H, Liu B, Jin L. Analysis of clinical and imaging features of frontotemporal dementia. Journal of Clinical Neurology. 2018;31(6):436-40. doi: 10.3969/j.issn.1004-1648.2018.06.011  850. Hu Z, Sun Y, Li P, Yu S, Zhang N. Case of lethargy, coldness and edema. Journal of Traditional Chinese Medicine. 2001;42(12):739-40. doi: 10.3321/j.issn:1001-1668.2001.12.020  851. Hu Z, Wu M, Wang P, Liu S. Effect of erector spinae plane block combined with general anesthesia on early postoperative cognitive function in elderly patients undergoing thoracoscopic radical resection of lung cancer. Chinese Journal of Anesthesiology. 2021;41(3):266-9. doi: 10.3760/cma.j.cn131073.20200422.00303  852. Huang J, Luo Q, Li M, Li J. The prevalence of cognitive impairment in patients with sarcopenia: a meta-analysis. Chinese Journal of Evidence-Based Medicine. 2023;23(09):1039-45.  853. Huang T, Zhang D, Qin L, Chen S, Mao Y, Bao H, et al. Age-related change in mitochondrial DNA copy number and its correlation with intrinsic capacity and body composition. Chinese Journal of Geriatrics. 2023;42(1).  854. Huang X, Liu C. Research progress in delayed hypogonadism. The World Clinical Medicine. 2016;10(15):121-3.  855. Huang Y. Human aging and aging diseases. Family Knowledge. 2009;(3):24-5.  856. Huang Y, Xu L, Zhuang P, Lin L, Zhang X. Meta-analysis of risk factors for sarcopenia in maintenance hemodialysis patients. Journal of Nursing Science. 2022;37(13):17-21.  857. Huo L, Su B. An analysis on risk factors for nosocomial infection in Alzheimer's patients. International Medicine and Health Guidance News. 2011;17(23):2949-52. doi: 10.3760/cma.j.issn.1007-1245.2011.23.041  858. Dai Y, Huo Y. Observation on the effect of sarcopenia training combined intervention in elderly patients with mild acute ischemic stroke. Chinese Journal of Convalescent Medicine. 2022;31(2).  859. Jiang F, Du X, Zhang J. Investigation of Frailty and Traditional Chinese Medicine Characteristics in Elderly  Patients with Chronic Obstructive Pulmonary Disease. Shandong Journal of Traditional Chinese Medicine. 2022;41(05):517-22+44. doi: 10.16295/j.cnki.0257-358x.2022.05.010  860. Jiang F, Li X, Zhang J, Wang Y. Correlation Between Traditional Chinese Medicine Constitution and the Comorbidities in Elderly Patients with Chronic Obstructive Pulmonary Disease：An Analysis of 3 408 Cases. Journal of Guangzhou University of Traditional Chinese Medicine. 2022;39(01):1-7. doi: 10.13359/j.cnki.gzxbtcm.2022.01.001  861. Jiang T, Wang P, Liang X, Fan S, Wei W, Wang Y. Correlation between sarcopenia and postoperative cognitive function decline in elderly patients undergoing hip surgery. Journal of Clinical Anesthesiology. 2022;38(08):795-800.  862. Jiang W, Chi Z, Shang W, Ma L, Wu W. Clinical features and changes of cerebral blood perfusion in children with alternating hemiplegia. Chinese Journal of Neurology. 2005;38(12):746-9. doi: 10.3760/j.issn:1006-7876.2005.12.005  863. Jiang X, Zhan B. Application effect of non-drug cluster nursing scheme on delirium patients in ICU. Guide of China Medicine. 2019;17(25):291-2.  864. Jiang J, Wang Y, Zhang J, Wang X, Liang Y, Shang S. Cortical basal ganglia degeneration: a case report. Journal of Apoplexy and Nervous Diseases. 2019;36(12):1123-6.  865. Jiang Z, Huang F, Zheng X, Lao Y. Perioperative risk assessment and control in elderly patients with intertrochanteric fracture of femur. The Journal of Traditional Chinese Orthopedics and Traumatology. 2015;(8):33-5.  866. Liang X, Fang M, Yang Y, Zhu T, Jiang H. Effect of Functional Electrical Stimulation Combined with Circulatory System on Limb Function,Balance Ability and Cognitive Function in Elderly Patients with Cerebral-stroke Sarcopenia. Progress in Modern Biomedicine. 2022;22(9).  867. Jiang Q, Tang H. Case report of a sporadic alzheimer's disease caused by psen2 gene mutation and literature review. Chinese Journal of Alzheimer's Disease and Related Disorders. 2021;4(4):302-5. doi: 10.3969/j.issn.2096-5516.2021.04.009  868. Jiang Y, Jiang W. The Development of Clinical Trial and Safety of Selegiline. Chinese Journal of Clinical Neurosciences. 2012;20(4):424-33. doi: 10.3969/j.issn.1008-0678.2012.04.013  869. Xie X, Chen Y, Li J. Study on the intervention of therapy for warming yang and supplementing qi on cardiotoxicity of anthracycline chemotherapy drugs. Modern Journal of Integrated Traditional Chinese and Western Medicine. 2023;32(6):786-91. doi: 10.3969/j.issn.1008-8849.2023.06.010  870. Jin J, Min S, Chen Q. Effect of low dose of ketamine combined with propofol on efficacy of electroconvulsive therapy in patients with depression. Journal of Clinical Anesthesiology. 2016;32(10):953-6.  871. Jin Q, Tian Q, Liu Y, Cui A, Gao Y, Rong Y, et al. The effects of ultrasound-guided local anesthesia onamyloid β-protein and Tau protein in patients. Journal of Guangxi Medical University. 2018;35(12):1663-6. doi: 10.16190/j.cnki.45-1211/r.2018.12.013  872. Jin Y, Wang Y, Zhang X, Zhou C, Guo Z. Observe and analyse the clinical curative effect of using GM-1 on treating premature white matter damage. Chinese Journal of Practical Pediatrics. 2006;21(9):666-8. doi: 10.3969/j.issn.1005-2224.2006.09.009  873. Kang J, Liu Y, Xiang W, Wu Z, Wang X, Zhang B, et al. Bilateral paramedian thalamic infarction induced by occlusion of artery of percheron:Clinical and imaging characteristics of 11 cases. Academic Journal of Chinese PLA Medical School. 2023;44(8):845-50,67. doi: 10.12435/j.issn.2095-5227.2023.073  874. Kang L, Zhu M, Gao Q, Zeng P, Liu X. Effect of continuing medical education on geriatrics on knowledge, attitude, belief and practice in community medical staffs. Chinese Journal of Multiple Organ Diseases in the Elderly. 2016;15(8).  875. Kang L, Zheng H. Analysis of 5 cases of mitochondrial encephalomyopathy with hyperlactacemia and stroke like seizure syndrome. Chinese Journal of Integrative Medicine on Cardio-Cerebrovascular Disease. 2015;13(11):1358-60. doi: 10.3969/j.issn.1672-1349.2015.11.051  876. Kang Q, Liao H, Yang S, Chen B, Yang L. ASAH1 Gene Mutation Causing Spinal Muscular Atrophy: 2 Cases Report and Literature Review. Neural Injury and Functional Reconstruction. 2020;15(10):575-8. doi: 10.16780/j.cnki.sjssgncj.20191666  877. Kang Y, Gao Y. Management of hip instability after total hip arthroplasty. China Journal of Orthopaedics and Traumatology. 2016;29(2):99-101. doi: 10.3969/j.issn.1003-0034.2016.02.001  878. Kong W, Xu G, Jia J, Cui X. Cerebrotendinous xanthomatosis:a case report and literature review. Journal of Shandong University(Health Sciences). 2021;59(11):72-5. doi: 10.6040/j.issn.1671-7554.0.2021.0849  879. Kuang C, Xia J, Wu S, Xu B. Research Progress of Exosomes-mediated Exercise Intervention in Alzheimer's Disease. China Sport Science and Technology. 2020;56(11):38-47. doi: 10.16470/j.csst.2020131  880. Lai B. How to get on the Healthy aging train. Health Care. 2017;(05):26-7.  881. Lan Z. Good muscle is the foundation of a long and healthy life. China Health Food. 2020;(7):54-7.  882. Lei P, Li Y. Research progress on the influence of psychosomatic therapy on perimenopausal symptoms. Chinese Journal of Gerontology. 2016;36(21):5467-70. doi: 10.3969/j.issn.1005-9202.2016.21.114  883. Lengh H. Research progress of nondrug analgesia for neonatal pain. Chongqing Medical Journal. 2013;42(4):461-3. doi: 10.3969/j.issn.1671-8348.2013.04.042  884. Li C, Cui Y, Luo G, Zhang C, Gao C, Zhang J. The relation between diaphragm function and sarcopenia and the application value of ultrasound diaphragmatic assessment in sarcopenia. Geriatrics & Health Care. 2023;29(4).  885. Li Z, Dai C, Gao X, Ji H, Lian S. Effect of transverse abdominal muscle plane block combined with dexmedetomidine on postoperative delirium in elderly patients undergoing gynecological surgery. China Practical Medicine. 2020;15(5):141-3. doi: 10.14163/j.cnki.11-5547/r.2020.05.063  886. Li C, Yu D, Xiao W, Zhang HP, Zhang Y, Shi J. Parkinson syndrome caused by acute carbon monoxide poisoning: a report of 15 cases. Chinese Journal of Neurology. 2006;39(10):650. doi: 10.3760/j.issn:1006-7876.2006.10.012  887. Li M, Xu M. Research update on frailty combination in elderly patients with heart failure. Chinese Journal of Multiple Organ Diseases in the Elderly. 2023;22(6).  888. Li C, Zhang H. A case of renal encephalopathy with periodic three-phase EEG manifestations and literature review. Chinese Journal of Rural Medicine and Pharmacy. 2021;28(8):38-9. doi: 10.3969/j.issn.1006-5180.2021.08.023  889. Li D, Tang G. Research Progress of Early Rehabilitation Treatment of ICU. Medicine & Philosophy. 2015;36(24):54-7.  890. Li D, Liu Y, Tian F, Jing W, Guo C. Effect of ultrasound-guided stellate ganglion + erector spinal block on POD and immune stress response in elderly patients with lung cancer. The Journal of Medical Theory and Practice. 2022;35(2):264-6. doi: 10.19381/j.issn.1001-7585.2022.02.035  891. Li D, Wang D, Yang W, Xiong Z, Song H, Liu J, et al. Research progresses of MR quantitative magnetic susceptibility imaging in neurodegenerative diseases. Chinese Journal of Medical Imaging Technology. 2019;35(8):1248-51. doi: 10.13929/j.1003-3289.201901195  892. Li F, Yang H, Wang J, Liu C. Deacetylase 1 mediates the pathogenesis of age-related diseases and its relationship with exercise. Chinese Journal of Sports Medicine. 2012;31(11).  893. Li G, Zhao J, Wang H, Sun R, Zhang H, Shi Y, et al. The research progress of CARASIL. Chinese Journal of Practical Nervous Diseases. 2020;23(15):1354-8. doi: 10.12083/sysj.2020.15.022  894. Li G. Nursing of a patient with Alzheimer's disease combined with Parkinson's disease. Journal of Frontiers of Medicine. 2013;(34):299-300. doi: 10.3969/j.issn.2095-1752.2013.34.334  895. Li H, Li Y, Cheng Y, Gu Y, Luo Y. Progress in role of Toll-like receptor 4 in Parkinson disease. Chinese Journal of Pathophysiology. 2021;37(4):744-51. doi: 10.3969/j.issn.1000-4718.2021.04.022  896. Li H, Jin C, Cui C. Neuroacanthocytosis with epilepsy as the first symptom: a case report. Shandong Medical Journal. 2015;(19):107-8. doi: 10.3969/j.issn.1002-266X.2015.19.042  897. Li H, Yang Z, Qian P, Xue J. ASAH1 gene mutation causing spinal muscular atrophy with progressive myoclonic epilepsy in a Chinese child and literature review. Chinese Journal of Applied Clinical Pediatrics. 2018;33(6):470-4. doi: 10.3760/cma.j.issn.2095-428X.2018.06.018  898. Li J, Zheng X, Li R, Liu X, Tian J, Chao Y. Clinical study of coronary artery bypass graft under fast tracking cardiac anesthesia management. Chinese Journal of Modern Clincal Medicine. 2003;1(7):586-7.  899. Li J, Li Y. Functional surgical treatment of dystonia. Chinese Journal of Stereotactic and Functional Neurosurgery. 2005;18(2):113-6. doi: 10.3969/j.issn.1008-2425.2005.02.017  900. Li J. Advances in diagnosis and treatment of PADAM. Basic and Clinical Medicine. 2006;26(3):231-7. doi: 10.3969/j.issn.1001-6325.2006.03.002  901. Huang J, Luo Q, Li M, Li J. The prevalence of cognitive impairment in patients with sarcopenia:a meta-analysis. Chinese Journal of Evidence-based Medicine. 2023;23(9).  902. Li J, Liu K. Dementia with lewy bodies: a case report. Journal of Clinical Psychiatry. 2001;11(3):186. doi: 10.3969/j.issn.1005-3220.2001.03.032  903. Li K, Li H. Advances in gene research of epileptic encephalopathy. Journal of Epilepsy. 2018;4(2):117-20. doi: 10.7507/2096-0247.20180024  904. Li L, Zhang Z, Fan Z. Advances in the treatment of neurodegenerative diseases by targeting Nrf2. Journal of Brain and Nervous Diseases. 2020;28(1):48-53.  905. Li N. Research progress of progressive supranuclear palsy. Journal of Chinese Practical Diagnosis and Therapy. 2016;30(5):429-31. doi: 10.13507/j.issn.1674-3474.2016.05.005  906. Li Q. Short-term Efficacy and Prognosis of Video-assisted Thoracoscopy in Patients with Thymoma and Muscle Weakness. The Practical Journal of Cancer. 2020;35(6):955-8. doi: 10.3969/j.issn.1001-5930.2020.06.024  907. Li Q, Tang J, Wang Y, Wang C. The effect of leukoosteoporosis on Parkinson's disease. Stroke and Nervous Diseases. 2001;8(5):269-. doi: 10.3969/j.issn.1007-0478.2001.05.021  908. Li S. Translational neurogenomics: Interpreting complex brain diseases. World Science. 2018;(8):15. doi: 10.3969/j.issn.1000-0968.2018.08.007  909. Li S, Li S. Brief Introduction to the Origin, Shape, Production Area, Sort and Differentiating of Wild Ginseng. Asia-Pacific Traditional Medicine. 2008;4(1):37-9.  910. Li S. Clinical analysis of the efficacy and safety of Donepezil hydrochloride in the treatment of vascular dementia. Gems of Health. 2018;(36):105,7.  911. Li S, Gong X, Xu W, Zhang F. Research progress of pulmonary function rehabilitation after stroke. Chinese Journal of Convalescent Medicine. 2020;29(7):687-91. doi: 10.13517/j.cnki.ccm.2020.07.006  912. Li S, Zhou S, Zhang L. The clinical features and nenrologic outcome of acute disseminated encephalomyelitis in children. Chinese Journal of Practical Pediatrics. 2009;24(4):281-5.  913. Li S, Wu X, Xie Y, Li X, Li R, Yan YS, et al. The clinical application of magnetic resonance DWI-ADC values in diagnosis of sarcopenia on perimenopausal womenx. Chinese Journal of Magnetic Resonance Imaging. 2020;11(5).  914. Li S, Li J, Song Q, Yu L, Wang W, Wang Y. Mechanism of alkaloids from wine steamed Coptidis Rhizoma-volatile oil from Acori Tatarinowii Rhizoma on prevention and treatment of diabetic cognitive impairment based on interactive network of "key components-potential targets-core pathway". Chinese Traditional and Herbal Drugs. 2021;52(19):5910-21. doi: 10.7501/j.issn.0253-2670.2021.19.014  915. Li W, Zeng C. Research progress on regulation effect of traditional Chinese medicine on epilepsy. Global Traditional Chinese Medicine. 2022;15(9):1742-8. doi: 10.3969/j.issn.1674-1749.2022.09.048  916. Li W, Li L, Zhao G, Hou J, Chen Y. Effects of dexmedetomidine on hemodynamics, renal function and brain protection in patients with heart valve replacement. Chinese Journal of Integrative Medicine on Cardio-Cerebrovascular Disease. 2022;20(11):2041-6. doi: 10.12102/j.issn.1672-1349.2022.11.026  917. Li X, An J, Ren Y. Early warning factors of vascular cognitive impairment. Journal of International Neurology and Neurosurgery. 2017;44(4):423-7. doi: 10.16636/j.cnki.jinn.2017.04.019  918. Li X, Xu Z, Wang X. Research progress on the role of gut microbes in the pathogenesis of Alzheimer's disease. Chinese Journal of Gerontology. 2022;42(18):4644-7. doi: 10.3969/j.issn.1005-9202.2022.18.068  919. Li X. Medical German reading. German medicine. 2001;18(1):58.  920. Li X, Ma J, Yang X. A systematic review of the nutritional status of the elderly in home care institutions in recent 5 years. Chinese Journal of Geriatric Care. 2021;19(4).  921. Li X, Liu X, Wang G. Clinical and polysomnographic features of rapid eye movement sleep behavior disorder. National Medical Journal of China. 2002;82(13):891-3. doi: 10.3760/j:issn:0376-2491.2002.13.008  922. Li X, Qiu L, Zhao L, Xu H, Lang Z, Zhang J, et al. Progress in clinical application of renal oxygen saturation monitoring. Journal of Medical Forum. 2020;41(3):166-9.  923. Li Y, Li C. Research Progress of PI4P Metabolic Enzymes and Their Inhibitors. China Pharmaceuticals. 2022;31(19):117-23. doi: 10.3969/j.issn.1006-4931.2022.19.027  924. Li Y, Shi Q, Lin Y, Liu M. Clinical analysis of skeletal muscle and small fiber involvement associated with anti-contactin-associated protein-like 2 antibodies positive Morvan syndrome. Chinese Journal of Neurology. 2022;55(8):861-7. doi: 10.3760/cma.j.cn113694-20211224-00923  925. Li Y, Zhang K. Advances in Parkinson's disease sleep disorder. Journal of Neurology and Neurorehabilitation. 2022;18(1):22-8. doi: 10.12022/jnnr.2021-0105  926. Li H, Chen X. Imaging features of idiopathic normal pressure hydrocephalus. Chinese Journal of Nervous and Mental Diseases. 2012;38(11):699-702. doi: 10.3969/j.issn.1002-0152.2012.11.0016  927. Li Y, Tian H. Seventy cases of tic disorder in children were treated by differentiation. New Chinese Medicine. 2008;40(4):83-4. doi: 10.3969/j.issn.0256-7415.2008.04.047  928. Li Y, Shan D, Liu N, Kang C, Zhi H. Application effect of laparoscopic nephron-sparing surgery and radical nephrectomy in localized renal cancer. Oncology Progress. 2022;20(24):2585-8. doi: 10.11877/j.issn.1672-1535.2022.20.24.28  929. Li Y, Wang Q, Xu J, Ding D, Li H, Zhu L, et al. Research progress in the animal models of Parkinson's disease depression. Journal of Clinical Medicine in Practice. 2019;23(10):122-6. doi: 10.7619/jcmp.201910036  930. Li Y. The effect of hyperbaric oxygen therapy on cognitive dysfunction in patients after traumatic brain injury. Modern Diagnosis and Treatment. 2015;26(21):4958-9.  931. Li Y, Song G, Lin L, Xin S, Cao Y. Inflammatory response after intranasal inoculation with an adenovirus vaccine encoding multivalent Aβ3-10 in Alzheimer's disease transgenic mice. Journal of Apoplexy and Nervous Diseases. 2017;34(5):393-7.  932. Tang W, Yan X, Fan P, Xu D, Li Y. Analysis of the Clincal Effect of Dietary Nutrition Intervention Combined with Resistance Training on the Cognitive Function of Elderly Male Patients with Sarcopenia. Progress in Modern Biomedicine. 2020;20(14).  933. Li C, Qi X, Liu J, Xiong B, Qiu F. Clinical characteristics of Baló's concentric sclerosis: analysis of 12 cases. Chinese Journal of Neurology. 2011;44(2):113-6. doi: 10.3760/cma.j.issn.1006-7876.2011.02.010  934. Li X. Clinical analysis of 42 cases with vasogenic Parkinson's syndrome. Medical Innovation of China. 2010;7(4):74-5. doi: 10.3969/j.issn.1674-4985.2010.04.049  935. Li X, Wang Z, Feng T, Li X. Clinical features and head magnetic resonance characteristics of progressive supranuclear palsy. Shanxi Medical Journal. 2013;42(16):910-2.  936. Liang B, Shang X, Pei L. Association between chronic diseases and possible sarcopenia in middle-aged and older Chinese men:A prospective cohort study. National Journal of Andrology. 2023;29(1).  937. Liang H, Mao W, Mu R, Yang Q, Zhang W. Tongdu Tiaoshen acupuncture combined with Chinese medicine in the treatment of progressive myoclonic epilepsy 7 type test cases 1. China's Naturopathy. 2023;31(3):82-5. doi: 10.19621/j.cnki.11-3555/r.2023.0325  938. Liang S. Influence of multilevel health management on the level of knowledge and practice of safe and rational drug use in elderly patients with chronic disease comorbidities. Medical Diet and Health. 2020;18(6).  939. Liang X, Fang M, Yang Y, Zhu T, Jiang H. Effect of Functional Electrical Stimulation Combined with Circulatory System on Limb Function, Balance Ability and Cognitive Function in Elderly Patients with Cerebral-stroke Sarcopenia. Progress in Modern Biomedicine. 2022;22(09):1713-7. doi: 10.13241/j.cnki.pmb.2022.09.023  940. Liang X, Chen Y, Wen H, He J, Tang NN, Zhai W. Risk factors for sarcopenia in elderly patients with maintenance hemodialysis: a multivariate logistic regression analysis. Internal Medicine. 2021;16(03):335-8. doi: 10.16121/j.cnki.cn45-1347/r.2021.03.15  941. Liang Y, Xue L, Gao Y, Hou Y, Wang X. Analysis of sarcopenia and influencing factors in hospitalized elderly patients. Chinese Journal of Geriatrics. 2020;39(9).  942. Liao J. Clinical protocol of ketogenic diet therapy. Chinese Journal of Applied Clinical Pediatrics. 2013;28(12):881-3. doi: 10.3760/cma.j.issn.2095-428X.2013.12.001  943. Lin J, Zhang Y, Han Y, Han Y, Mao X, Yin F, et al. Progressive supranuclear palsy: a case report and literature review. Journal of Apoplexy and Nervous Diseases. 2015;32(7):638-40.  944. Lin J, Zhao J. Effects of different fluid treatments on postoperative cognitive dysfunction in patients undergoing major laparoscopic surgery. China Modern Doctor. 2023;61(6):30-4. doi: 10.3969/j.issn.1673-9701.2023.06.008  945. Lin L, Lin X, Lin C, Huang Q. Application of Ultrasound-guided Transverses Abdominis Plane Block Assisted with Dexmedetomidine in the Elderly Patients Undergoing Inguinal Hernia Repair. Journal of Fujian Medical University. 2016;50(5):325-9.  946. Lin S, Huang L, Lin X, Zhang H, Li J. Comparison of application effects of different approaches of laparoscopic nephron sparing nephrectomy in the treatment of renal tumors. Journal of North Sichuan Medical College. 2023;38(6):818-21. doi: 10.3969/j.issn.1005-3697.2023.06.022  947. Lin T, Yue J. To explore the pathogenesis of sarcopenia from serum inflammatory markers. Practical Geriatrics. 2019;33(9).  948. Liu B, Wang Y, Cheng N. Autoimmune encephalitis with anti-LGI1 antibody: a case report and literature review. Journal of Changzhi Medical College. 2023;37(1):55-7. doi: 10.3969/j.issn.1006-0588.2023.01.013  949. Liu B, Li S, Zhao Q. Research progress of ultrasound-guided drug injection in the treatment of post-stroke pain. Chinese Journal of Gerontology. 2021;41(18):4124-30. doi: 10.3969/j.issn.1005-9202.2021.18.066  950. Liu D, Zhang H, Bao Y, Sun J, Zhang H. Syngnathus pharmacological activity research progress. Jilin Journal of Chinese Medicine. 2015;(10):1040-2. doi: 10.13463/j.cnki.jlzyy.2015.10.021  951. Liu H, Zhang N, Zhao Y, Zhang Q, Zhang K. Advances in the pathogenesis of Parkinson's disease. Chinese Journal of Geriatric Heart Brain and Vessel Diseases. 2015;(11):1230-2. doi: 10.3969/j.issn.1009-0126.2015.11.032  952. Liu H, Zou L. The causes of death of inpatients with mental disorders were analyzed. Health Guide. 2020;(52):281.  953. Liu H, Yang J, Cao H, Jia S. Physical activity and exercise rehabilitation for children with intellectual disabilitiy: a systematic review of sys-tematic reviews. Chinese Journal of Rehabilitation Theory and Practice. 2022;28(9):993-1002. doi: 10.3969/j.issn.1006⁃9771.2022.09.001  954. Liu J, Hong Y. The Treatment of Vascular Dementia Combined XingNao-YiZhiTang with Western Medicine-A Clinical Observation on 60 Patients. Journal of Longdong University. 2017;28(1):78-80. doi: 10.3969/j.issn.1674-1730.2017.01.017  955. Liu J, Xu H. Research progress in clinical diagnosis and treatment of frailty. Electronic Journal of Metabolism and Nutrition of Cancer. 2019;6(1).  956. Peng N, Cao M, Liu J. Several problems worth paying attention to in senile sarcopenia. Geriatrics & Health Care. 2020;26(1).  957. Liu J, Mou C. Research progress on the relationship between uric acid and non-motor symptoms of Parkinson's disease. Laboratory Medicine and Clinic. 2016;13(15):2219-21. doi: 10.3969/j.issn.1672-9455.2016.15.060  958. Liu J, Ding Q, Zhou B. Strengthen the prevention and standard treatment of sarcopenia. China Medical News. 2021;36(18).  959. Liu J, Ding Q, Zhou B, Liu X, Liu J, Liu Y, et al. Chinese expert consensus on diagnosis and treatment for elderly with sarcopenia(2021). Chinese Journal of Geriatrics. 2021;40(8).  960. Liu M, Fu C, Kang X. Cell therapy for Parkinson's disease. Neijiang Technology. 2020;41(3):95-6.  961. Liu P, Zhang J. Analysis of 10 misdiagnosed cases of primary hypothyroidism. Chinese Journal of Misdiagnostics. 2009;9(25):6152-.  962. Liu Q, Ding S, Shang Y. Research progress in the treatment of neurodegenerative diseases by traditional Chinese medicine. Journal of Chengde Medical University. 2021;38(6):518-21.  963. Liu Q, Liu M, Zhang Y. Hospital full cycle rehabilitation model for patients with Parkinson's disease. Geriatrics & Health Care. 2020;26(3):340-2. doi: 10.3969/j.issn.1008-8296.2020.03.002  964. Liu R. Health guidance in convalescent period of children with JE. Chinese Clinical New Medicine. 2004;4(12):1148-.  965. Liu S, Li M, Zhu J, Zhang Y, Wu Y, Liu C, et al. Correlation of muscle strength with cognitive function and medial temporal lobe atrophy in patients with mild to moderate Alzheimer's disease. National Medical Journal of China. 2022;102(35).  966. Liu X. Effect of Jiuwei Zhenxin Granule combined with aripiprazole on schizophrenia. Modern Journal of Integrated Traditional Chinese and Western Medicine. 2018;27(30):3347-50. doi: 10.3969/j.issn.1008-8849.2018.30.012  967. Liu X, Shan W, Ren J, Ren G, Wang Q. Clinical progress of leucine-rich glioma inactivated-1 antibody-mediated encephalitis. Chinese Journal of Neuromedicine. 2019;18(3):306-11. doi: 10.3760/cma.j.issn.1671-8925.2019.03.019  968. Liu X. What is the diet nursing notice of the sequela of encephalitis. Health Guide. 2020;(41):47.  969. Liu X, Xiang D, Ding Q. Proceedings of the 2011 American Geriatric Society Scientific Annual Meeting. Chinese Journal of Geriatrics. 2011;30(8):700-2. doi: 10.3760/cma.j.issn.0254-9026.2011.08.026  970. Liu X, Zhang Y, Song Y. Clinical analysis of three cases of mitochondrial encephalomyopathy in children with lacticemia and stroke-like attack. Chinese Journal of Contemporary Neurology and Neurosurgery. 2016;16(5):291-6. doi: 10.3969/j.issn.1672-6731.2016.05.009  971. Liu X, Yue J, Qiao R, Hou L, Dong B. The role of intestinal flora in the pathogenesis of sarcopenia. Practical Geriatrics. 2019;33(9).  972. Liu X, Zhang L, Cao S. Progress of clinical research on senile diabetes mellitus. Electronic Journal of Clinical Medical Literature. 2019;6(81):193-4. doi: 10.16281/j.cnki.jocml.2019.81.155  973. Liu X, Li F, Ma J, Liu J. A systematic evaluation of the treatment of insomnia by soothing the liver. Hebei Journal of Traditional Chinese Medicine. 2013;(11):1705-8. doi: 10.3969/j.issn.1002-2619.2013.11.062  974. Liu Y, Chen W, Huang X. Overview of traditional Chinese medicine treatment and syndrome differentiation for epilepsy. Beijing Journal of Traditional Chinese Medicine. 2011;30(8):638-40.  975. Liu Y, Dai R. A case report of Creutzfeldt-Jakob disease misdiagnosed as Alzheimer's disease. Journal of Epileptology and Electroneurophysiology. 2018;27(5):319-20.  976. Liu J, Ding Y, Zhang J. A case of Creutzfeldt-Jakob disease was diagnosed by M.RI. Journal of Medical Imaging. 2014;(10):1748-,61.  977. Long N, Wu Y. Understanding and research progress of sarcopenia. Chinese Journal of Health Care and Medicine. 2018;20(5).  978. Lou F, Li J, Luo X, Chang Y, Qu Y, Ren Y. Research advances in sleep disorders in Parkinson's disease. Chinese Journal of Geriatrics. 2010;29(11):964-6. doi: 10.3760/cma.j.issn.0254-9026.2010.11.030  979. Lu W, Song K, Wang L, Cai Y, Xie H, Wang W, et al. Analysis on two cases of adolescent type of Hallervorden─ Spatz disease. Chinese Journal of Tissue Engineering Research. 2001;5(15):54-5. doi: 10.3321/j.issn:1673-8225.2001.15.022  980. Lu H, Zhang T. The risk factors of lower urinary tract dysfunction and urodynamics study in stroke patients in the convalescent stage. Chinese Journal of Neurology. 2010;43(1):56-9. doi: 10.3760/cma.j.issn.1006-7876.2010.01.014  981. Lu J, Feng L, Chen J, Yang R. Nursing care of 1 patient with anti-LGI1 encephalitis. Modern Clinical Nursing. 2017;16(11):83-5. doi: 10.3969/j.issn.1671-8283.2017.11.019  982. Lu S, Sun C. Research progress on the effects of immune aging on bone metabolism and bone-derived hormone secretion. Journal of Xinxiang Medical University. 2019;36(9).  983. Lu B, Liu Y, Chen J, Gu L, Fan Y, Ouyang X. Correlation between sarcopenia in elderly inpatients. Chinese Journal of General Practice. 2021;19(11):1819-22. doi: 10.16766/j.cnki.issn.1674-4152.002175  984. Lu H, Wang Q. Lewy body dementia: a case report. Journal of Guangxi Medical University. 2000;17(6):981. doi: 10.3969/j.issn.1005-930X.2000.06.112  985. Lu L. A preliminary study on Professor Shan Zhaowei's experience in the diagnosis and treatment of chronic constipation. Modern Interventional Diagnosis and Treatment in Gastroenterology. 2019;24(10):1209-11. doi: 10.3969/j.issn.1672-2159.2019.10.034  986. Lu X, Mei B, Chen S, Liu X, Yu X, Gu E. The clinical observation of ultra-sound guided lumbar plexus and sciatic plexus nerve block combined with general anesthesia on elderly patients undergoing hip replacement surgery. Journal of Clinical Anesthesiology. 2016;32(3):237-40.  987. Lu X, Ye J, Zhou S, Lu B, Chen X, Liu L. A case of hemineglect and visual wholeness and facial agnosia after stroke. National Medical Journal of China. 2005;85(21):1509. doi: 10.3760/j:issn:0376-2491.2005.21.023  988. Lu Y, Li D, Sun S, Song X, Wu H, Zhang H, et al. Myotonic dystrophy with leukoencephalopathy. Chinese Journal of Nervous and Mental Diseases. 2019;45(3):166-70. doi: 10.3969/j.issn.1002-0152.2019.03.008  989. Lv J. Research progress on the relationship between uric acid and nervous system diseases. Journal of Apoplexy and Nervous Diseases. 2013;30(7):666-7.  990. Lv P, Zhang Y, Zhang L, Dong M, Tian Z, Wang X, et al. A case of Kennedy disease with hemilimb weakness was reported. Journal of China-Japan Friendship Hospital. 2021;35(1):52,封2. doi: 10.3969/j.issn.1001-0025.2021.01.015  991. Lv X. Dizziness involves many problems, and it is important to understand the cause. Jiangsu Journal of Health Care. 2022;(12).  992. Luan Z, Xu P, Chi Q. Clinical application of atypical antipsychotic drugs. Guide of China Medicine. 2010;8(34):46-8. doi: 10.3969/j.issn.1671-8194.2010.34.026  993. Ma A, Guo X, Li D, Zhang B, Pan X. A study on the cerebral glucose metabolism in progressive supranuclear palsy. Chinese Journal of Internal Medicine. 2012;51(11):885-8. doi: 10.3760/cma.j.issn.0578-1426.2012.11.013  994. Ma H, Yang H, Guo X, Geng X, Qiu K. Research Progress in the Prevention and Treatment of Diseases with Acupuncture and Moxibustion by Regulating VEGF. Chinese Journal of Library and Information Science for Traditional Chinese Medicine. 2023;47(5):228-32. doi: 10.3969/j.issn.2095-5707.202209092  995. Ma J, Cui M, Jia M, Cui L. Report of 2 cases of serotonin syndrome. Journal of Neuroscience and Mental Health. 2004;4(5):410-. doi: 10.3969/j.issn.1009-6574.2004.05.038  996. Ma L, Cai Y, Hu Z, Chen Y, Fang F. Application of demonstration education based on behavior change theory in pelvic floor muscle function exercise of patients with prostate cancer after operation. China Modern Doctor. 2023;61(19):84-7,96. doi: 10.3969/j.issn.1673-9701.2023.19.020  997. Ma L, Su Q, Ma Y, Lv L, Liu H, Han L. Validity and reliability of the Chinese Version of Rapid Geriatric Assessment. Journal of Nursing Science. 2019;34(18).  998. Ma Q. Discussion on nursing safety of elderly patients in Department of Cardiology. Chinese Community Doctors. 2012;14(21):337-8. doi: 10.3969/j.issn.1007-614x.2012.21.314  999. Ma Y, Xu K, Chen G, Zou Y. Clinical observation of piracetam in the treatment of intractable myoclonic epilepsy in children. Chinese Journal of Practical Nervous Diseases. 2016;19(7):117-8. doi: 10.3969/j.issn.1673-5110.2016.07.075  1000. Ma Y, Yang L. Research progress of rehabilitation therapy for cerebral infarction patients. Diet Health. 2018;5(24):296. doi: 10.3969/j.issn.2095-8439.2018.24.380  1001. Meng W, Liu C, Xia X, Li Y, Li Z, Zhu X, et al. Effect of TSG on tau phosphorylation via GSK-3βpathway. Journal of Hainan Medical University. 2022;28(14):1059-67. doi: 10.13210/j.cnki.jhmu.20220418.001  1002. Meng J, Zhu F. Clinical Research on the Effect of Living Quality for Treating Patients of Obstetrics and Gynecology Postoperative Urinary Retention with Integrated Nursing of TCM. Liaoning Journal of Traditional Chinese Medicine. 2015;42(4):856-8. doi: 10.13192/j.issn.1000-1719.2015.04.074  1003. Meng Q, Chai X, Hu G. Recent advances in depression after Parkinson's disease. Chinese Journal of Gerontology. 2013;33(2):477-80. doi: 10.3969/j.issn.1005-9202.2013.02.119  1004. Meng Y, Li J, Li F. Effect of sarcopenia on the prognosis and quality of life of elderly patients with non-ST-segment elevation acute coronary syndrome. Beijing Medical Journal. 2022;44(11).  1005. Mi L, Fu S, Yuan Z, Jiang G, Peng K, Tan Z, et al. Treatment of fresh femoral neck fracture with Alzheimer's disease by hip arthroplasty via minimally invasive direct anterior approach (an observation of short-term outcomes). Chinese Journal of Bone and Joint Injury. 2017;32(10):1012-4. doi: 10.7531/j.issn.1672-9935.2017.10.002  1006. Miao D, Liao L. Effectiveness and safety of solifenacin succinate in treatment of overactive bladder. Adverse Drug Reactions Journal. 2015;(2):134-7. doi: 10.3760/cma.j.issn.1008-5734.2015.02.018  1007. Miao Q. Research in Epworth scale on preliminary screening of patients with obstructive sleep apnea syndrome. Chinese Journal of Practical Nursing. 2009;25(16):20-1. doi: 10.3760/cma.j.issn.1672-7088.2009.06.008  1008. Min D, Chen X, Shen D. A case report of the dysinhibitory amyotrophic lateral sclerosis (ALS) -Parkinson-dementia complex. Chinese Journal of Neurology. 2001;34(1):51. doi: 10.3760/j.issn:1006-7876.2001.01.025  1009. Mou Y, Zhu B. The progress of the relationship of geriatric syndrome and human microbiome. Practical Journal of Clinical Medicine. 2020;17(5).  1010. Miao Z, Lu F, Ma B, Liu X. Advances in human mesenchymal stem cell exosomes. Chinese Medicinal Biotechnology. 2019;14(4):361-5. doi: 10.3969/j.issn.1673-713X.2019.04.013  1011. Na K. Whether statins can be used in elderly patients with cognitive impairment. Chinese Journal for Clinicians. 2019;47(9):1009-10. doi: 10.3969/j.issn.2095-8552.2019.09.001  1012. Ni Z. 30 cases of hydrocephalus after decompressive craniectomy. Chinese Journal of Practical Medicine. 2012;39(10):103-4. doi: 10.3760/cma.j.issn.1674-4756.2012.10.048  1013. Nie Y. Analysis of 10 cases of adult hypothyroidism misdiagnosed as nervous system disease. China Practical Medicine. 2015;(32):106-7. doi: 10.14163/j.cnki.11-5547/r.2015.32.073  1014. Nie Y. Analysis of 10 cases of adult hypothyroidism misdiagnosed as nervous system disease. Chinese Journal of Trauma and Disability Medicine. 2016;24(11):32-3. doi: 10.13214/j.cnki.cjotadm.2016.11.020  1015. Ning W. "Soft and fat" old people are prone to dementia. Health & Life. 2023;(5).  1016. Ning X, Zhang A. Advances in Research on the Effects of Iron Deficiency on the Human. China Modern Doctor. 2008;46(16):50-2. doi: 10.3969/j.issn.1673-9701.2008.16.023  1017. Niu Q, Ding X. The Cyanobacteria/β-N-methylamino-L-alanine Hypothesis of the Pathogenesis of Amyotrophic Lateral Sclerosis and Other Neurodegenerative Diseases. Chinese Journal of Clinical Neurosciences. 2012;20(3):307-11. doi: 10.3969/j.issn.1008-0678.2012.03.013  1018. Niu Z, Rao X. Traditional Chinese medicine understanding and intervention strategy of health-related quality of life in chronic kidney disease. Beijing Journal of Traditional Chinese Medicine. 2020;39(09):901-5. doi: 10.16025/j.1674-1307.2020.09.001  1019. Ou M, Li S, Liu G, Jia J, Yu C. A System Review of the Critical Role of Vitamin D in the Clinical Implications of Vitamin D Deficiency. Progress in Modern Biomedicine. 2014;14(19):3798-800. doi: 10.13241/j.cnki.pmb.2014.19.051  1020. Ou M, Hu Z. Non-alcoholic subacute primary dege-neration of corpus callosum:A case report and literature review. Journal of International Neurology and Neurosurgery. 2023;50(1):59-63. doi: 10.16636/j.cnki.jinn.1673-2642.2023.01.012  1021. Ou W, Kong Q, Liu H, Ouyang L. Analysis of therapeutic effect of multi-channel antibiotic therapy on intracranial infection. The Journal of Medical Theory and Practice. 2022;35(16):2742-5. doi: 10.19381/j.issn.1001-7585.2022.16.014  1022. Xia S, Guo S, Pan H. Assessment of sarcopenia and its correlation with sleep Research Assessment of sarcopenia and its correlation with sleep research. Practical Geriatrics. 2020;34(12).  1023. Pan Q, Dai F, Pan W, Liu J, Chen R. Recent Developments in Oral Frailty in the Elderly. Chinese General Practice. 2022;25(36).  1024. Pan S. Discussion on TCM syndrome and treatment of chronic fatigue syndrome. 浙江中医杂志  Zhejiang Journal of Traditional Chinese Medicine. 2008;43(1):29. doi: 10.3969/j.issn.0411-8421.2008.01.014  1025. 庞博, 张子华, 胡鑫, 张洋, 纪仲秋, 贺子荣, et al. 老年人轻度认知功能损害与跌倒风险关系研究进展. 中国老年学杂志. 2021;41(5):1115-20. doi: 10.3969/j.issn.1005-9202.2021.05.061  1026. Pang M, Song J, Fu J, Li G, Ma M. A family study of autosomal recessive primary familial brain calcification caused by JAM2 gene mutation and literature review. Chinese Journal of Neurology. 2022;55(2):140-5. doi: 10.3760/cma.j.cn113694-20210527-00370  1027. Pei S, Wen C, Li Y. Clinical effect of cerebral glycoside carnosine in the adjuvant treatment of cognitive dysfunction after cerebral infarction. Practical Clinical Journal of Integrated Traditional Chinese and Western Medicine. 2021;21(2):50-2. doi: 10.13638/j.issn.1671-4040.2021.02.023  1028. Peng G, Wu Y, Chen Y, Zhang Z, Wang G. Survey about quality of life in 116 uremia patients after renal transplantation. Chinese Journal of Tissue Engineering Research. 2011;15(5):909-12. doi: 10.3969/j.issn.1673-8225.2011.05.036  1029. Peng L. Clinical symptoms and treatment of patients with drug-induced Parkinson's syndrome. Journal of Medical Information. 2014;(22):576-. doi: 10.3969/j.issn.1006-1959.2014.22.703  1030. Peng L, Liu Y, Li J, Wang M, Lu H. Clinical analysis of 14 patients with voltage-gated potassium channels complex antibody encephalitis. Chinese Journal of Neuroimmunology and Neurology. 2018;25(2):97-101,28. doi: 10.3969/j.issn.1006-2963.2018.02.005  1031. Peng N, Cao M, Liu J. Several Issues for Concerns Regarding Sarcopenia in the Elderly. Geriatrics & Health Care. 2020;26(1).  1032. Pu L, Liang Z. Adavance of Pariknson’s disease-cognitive impairment based on 18 F-FDG PET. Journal of Practical Radiology. 2014;(6):1046-9. doi: 10.3969/j.issn.1002-1671.2014.06.039  1033. Qi X, Gao X, Xu H, Zhao L, Yu Z, An Y. Study on anesthesia in patients with lower extremity fracture complicated by calf intermuscular venous thrombosis. Modern Journal of Integrated Traditional Chinese and Western Medicine. 2015;24(2):193-5. doi: 10.3969/j.issn.1008-8849.2015.02.031  1034. Qi M, Tao Y, Wang L, Wang L. Research on translation and psychometric evaluation and test of chinese version of Stroke Impact Scale 3.0 for Proxy. Chinese Journal of Nervous and Mental Diseases. 2006;32(3):199-205. doi: 10.3969/j.issn.1002-0152.2006.03.002  1035. Qian H, Chen L, Liu J, Li J, He Y, Xian W, et al. The bilateral subthalamic nucleus deep brain stimulation can improve the symptoms in patients with moderate or advanced Parkinson's Disease. Chinese Journal of Nervous and Mental Diseases. 2009;35(4):203-7. doi: 10.3969/j.issn.1002-0152.2009.04.002  1036. Qin X, Chen W, Liu T, Cao J, Gao Y. Advances in biomarkers related to chronic heart failure complicated with frailty syndrome. Chinese Journal of Geriatric Heart Brain and Vessel Diseases. 2022;24(12):1330-2. doi: 10.3969/j.issn.1009-0126.2022.12.026  1037. Qin X, Xu Z, Chen Y, Shang T, Xiong J. Clinical analysis of neurological involvement in acquired immunodeficiency syndrome. Chinese Journal of Contemporary Neurology and Neurosurgery. 2011;11(5):548-53. doi: 10.3969/j.issn.1672-6731.2011.05.015  1038. Qu J. Treatment of Parkinson's disease. Progress in Japanese Medicine. 2005;26(10):470-3.  1039. Quan Z, Zeng H, Fang Z, Yang X. Hyperbaric oxygen treatment of severe carbon monoxide poisoning complicated with hematomas of psoas major and iliopsoas muscle: a case report. Chinese Journal of Nautical Medicine and Hyperbaric Medicine. 2023;30(1):134-5. doi: 10.3760/cma.j.cn311847-20210112-00014  1040. Niu Z, Rao X. Discussion on TCM cognition and intervention strategy of health-related quality of life in chronic kidney disease. Beijing Journal of Traditional Chinese Medicine. 2020;(9).  1041. Rao Z, Zheng L, Chang Y, Yu T, Shi R. Research status and prospect of soluble Klotho in motion regulation. Chinese Journal of Sports Medicine. 2019;(12).  1042. Ren B, Qiu T, Xu S, Guo B, Hui Q, Chang P, et al. The diagnosis, treatment and research progress of Madelung's disease. Chinese Journal of Aesthetic and Plastic Surgery. 2016;27(10):626-8. doi: 10.3969/j.issn.1673-7040.2016.10.016  1043. Ren G, Zhao J. Clinical analysis of 11 AIDS patients with neurological symptoms. Modern Practical Medicine. 2014;26(1):76-7. doi: 10.3969/j.issn.1671-0800.2014.01.046  1044. Ren M, Chen S. Pathological crying and laughing acupuncture treatment case. The Journal of Medical Theory and Practice. 2021;34(21):3860. doi: 10.19381/j.issn.1001-7585.2021.21.080  1045. Ren Z, Zhou Y, Yao X. Understanding Elderly Frailty in the Community:Exploring Influencing Factors and Analyzing the Situation. International Journal of Geriatrics. 2023;44(4).  1046. Ru L, Guli EAS, Wang Y, La AM, Yan M. Analysis of Gene Mutation and Clinical Features in Children with Duchenne Muscular Dystrophy. Progress in Modern Biomedicine. 2022;22(14):2784-90. doi: 10.13241/j.cnki.pmb.2022.14.037  1047. Ru M. Six cases of cerebral cysticercosis. Chinese Journal of Zoonoses. 2000;16(4):23. doi: 10.3969/j.issn.1002-2694.2000.04.049  1048. Sang D, Zheng S. Vitamin D and Age-related Diseases. International Journal of Geriatrics. 2023;44(01):82-6.  1049. Shang D, Tian J, Ji Y, Shi C, Wu J, Yang J, et al. Mutational analysis of amyloid precursor protein gene in a family with Alzheimer disease. Journal of Zhengzhou University(Medical Sciences). 2015;50(3):416-9. doi: 10.13705/j.issn.1671-6825.2015.03.029  1050. Shang X, Su Q, Zhou Z. Spinocerebellar ataxia type 3:Clinical pictures and mutation in 4 cases. Journal of Wannan Medical College. 2016;35(2):133-5. doi: 10.3969/j.issn.1002-0217.2016.02.008  1051. Shao J, Zhang X, Yang Y, Wen R, Li Z, Ma D, et al. Research progress of flavonoids for prevention and treatment of Alzheimer's disease. China Pharmacy. 2020;31(24):3066-72. doi: 10.6039/j.issn.1001-0408.2020.24.21  1052. Shao Z, Zhang D, Lei Y, Liu S, Liu W, Wang M, et al. Voxel-based morphological study of cerebral gray matter volume in type 2 diabetic patients with microvascular complications. Journal of Shanxi Medical University. 2021;52(12):1615-20. doi: 10.13753/j.issn.1007-6611.2021.12.017  1053. Shao Z, Wang R, Xue S. Clinical Analysis of Parkinson's Disease Complications in 56 Patients. Chinese General Practice. 2010;13(6):649-51. doi: 10.3969/j.issn.1007-9572.2010.06.035  1054. Shen Y, Zou Z. Smoking can also cause intestinal gas. Home Medicine. 2018;(9):49.  1055. Shi X, Bao X, Liu X, Hou S, Qin J, Wu X. Clinical analysis of the variants of benign childhood epilepsy with centro-temporal spikes. Chinese Journal of Practical Pediatrics. 2014;29(7):528-33. doi: 10.7504/ek2014070611  1056. Shi K, Shang X. The clinical analysis of LGi1-antibody-related limbic encephalitis. Journal of Apoplexy and Nervous Diseases. 2017;34(6):523-7.  1057. Shi X, Wan Q, Ji W, Liu C. Physical activity scale and application progress in elderly population. Chinese Journal of Gerontology. 2020;40(13):2896-900. doi: 10.3969/j.issn.1005-9202.2020.13.065  1058. Shi X. Nursing care of a patient with lateral ventricular drainage in perioperative period of chronic subdural hematoma. Modern Nurse. 2017;(7):163-4.  1059. Shi W, Li H, Li X, Su Y, Zhao L, Lu K, et al. Reliability and validity of the Chinese version of North Star Ambulatory Assessment for children with Duchenne muscular dystrophy. Chinese Journal of Evidence-Based Pediatrics. 2017;12(4):246-50. doi: 10.3969/j.issn.1673-5501.2017.04.002  1060. Shi X. Analysis of intervention effect of Zhixinxing health education model on patients with unstable angina pectoris. Shanxi Medical Journal. 2022;51(16):1915-7. doi: 10.3969/j.issn.0253-9926.2022.16.040  1061. Song Q, Chao A. Discussion on the pathogenesis of sarcopenia in elderly patients with non-operative hip fracture. Jilin Medical Journal. 2021;42(05):1241-4.  1062. Song T, Li H, Li X. Pure autonomic failure: a case report. Chinese Journal of Neurology. 2021;54(9):949-51. doi: 10.3760/cma.j.cn113694-20201129-00920  1063. Song W, Wang J, Liu J. Research on senile asthenic syndromex. Journal of Brain and Nervous Diseases. 2018;26(9).  1064. Song Y, Ge L, Kong J, Guo P, Liu L. Relationship between NDUFV1 and NDUFS1 gene changes and childhood hereditary leukoencephalopathy. Experimental and Laboratory Medicine. 2019;37(6):1006-8,18. doi: 10.3969/j.issn.1674-1129.2019.06.006  1065. Song Z, Shi J, Xu J, Chen J, Jiang W, Wang L, et al. The Clinical and Follow-up Analysis of 8 Cases with Creutzfeldt-Jakob. Clinical Misdiagnosis & Mistherapy. 2009;22(12):44-5. doi: 10.3969/j.issn.1002-3429.2009.12.028  1066. Sun C, Li J, Lin L, Liu X. Research progress on nervous system related diseases complicated with pneumonia. Chinese Journal of Geriatrics. 2023;42(3).  1067. Sun J, Li M, Liu Y. Advances in the role of antisense oligonucleotides in degenerative diseases of the central nervous system. Journal of Apoplexy and Nervous Diseases. 2020;37(12):1137-40. doi: 10.19845/j.cnki.zfysjjbzz.2020.0541  1068. Sun P, Wang H. Detection significance of serum myocardial enzymes in patients with schizophrenia. Chinese Journal of Laboratory Diagnosis. 2017;21(10):1788-9. doi: 10.3969/j.issn.1007-4287.2017.10.040  1069. Sun X, Wang P, Cui Y. Efficacy of superficial temporal artery-middle cerebral artery branch anastomosis combined with encepho-du-ro-myo-synangiosis versus encephalo-duro-arterio-myo-synangiosis in the treatment of patients with moyamo-ya disease. Hainan Medical Journal. 2023;34(17):2493-8. doi: 10.3969/j.issn.1003-6350.2023.17.013  1070. Sun X, Wei G, Cai K, Yang X, Liu C. Effect of laparoscopic nephron sparing nephrectomy on renal function and quality of life in patients with renal cell carcinoma. Oncology Progress. 2022;20(12):1289-92. doi: 10.11877/j.issn.1672-1535.2022.20.12.27  1071. Sun X, Dong B. The importance and characteristics of nutritional therapy for chronic diseases. Journal of Modern Clinical Medicine. 2017;43(4).  1072. Sun Y, Liu Y. Research progress of liver diseases and psychoneurotic disorders. Practical Journal of Organ Transplantation(Electronic Version). 2018;6(1):76-82. doi: 10.3969/j.issn.2095-5332.2018.01.020  1073. Sun Z, Zhao Z. Clinical analysis of 60 cases of delayed encephalopathy caused by acute carbon monoxide poisoning. China Health Care & Nutrition. 2014;(7):4733-4.  1074. Suo L, Wang J, Sun L, Liu J, Li J. Benzodiazepines:benefits, risks and rational drug use. Chinese Journal of Drug Dependence. 2019;28(3):163-71. doi: 10.13936/j.cnki.cjdd1992.2019.03.001  1075. Tan C, Qiu Y, Chen M, Lin X. The effect of early rehabilitation strategy on the long-term prognosis of elderly critically ill mechanicaly ventilated patients. Chinese Journal of Geriatric Care. 2023;21(4):142-6. doi: 10.3969/j.issn.1672-2671.2023.04.033  1076. Tan H, Liang J, Guo Q, Hua G, Chen J, Tang X, et al. Total corpus callosum incision for drug refractory epilepsy. Chinese Journal of Minimally Invasive Neurosurgery. 2020;25(8):341-4. doi: 10.11850/j.issn.1009-122X.2020.08.002  1077. Tan Y, Liu Y, Kuang J, Ding W. Effects of unilateral and bilateral non-convulsive electric shock on the cognitive function in patients with schizophrenia. Journal of Modern Electrophysiology. 2021;28(3):154-8. doi: 10.3969/j.issn.1672-0458.2021.03.006  1078. Tan Z, Guo R, Tan Z, Liu D, Guo S. Clinical application and research progress of statins in the treatment of acute myocardial infarction. China Medical Herald. 2018;15(20):21-4.  1079. Tang Q, Cao L. Intestinal flora and neurological disorders. Chinese Journal of Biotechnology. 2021;37(11):3757-80. doi: 10.13345/j.cjb.210253  1080. Tang Y, Chu X, Wang T, Chen Y, Xie C, Wang YY, et al. Application of paeoniflorin in the treatment of autoimmune diseases. Chinese Journal of Cellular and Molecular Immunology. 2022;38(12):1143-50.  1081. Tang F, Li H, Liang H, Jin Z, Wei X, Geng D. A case of manic episode caused by paralytic dementia and literature review. Journal of Clinical Psychiatry. 2019;29(3):148. doi: 10.3969/j.issn.1005-3220.2019.03.003  1082. Tang W, Yan X, Fan P, Xu D, Li Y. Analysis of the Clincal Effect of Dietary Nutrition Intervention Combined with Resistance Training on the Cognitive Function of Elderly Male Patients with Sarcopenia. Progress in Modern Biomedicine. 2020;20(14):2698-701+727. doi: 10.13241/j.cnki.pmb.2020.14.021  1083. Tang Y, Jiao L, Cao L, Yuan B, Li Q. Perioperative nursing of chorea-acanthocytosis 1 routine deep brain stimulation. Fujian Medical Journal. 2023;45(4):149-51. doi: 10.3969/j.issn.1002-2600.2023.04.051  1084. Tao S, Huang H. Effect of modified electroconvulsive therapy on EEG and cognitive function in patients with mental illness. Zhejiang Clinical Medicine Journal. 2012;14(1):49-51. doi: 10.3969/j.issn.1008-7664.2012.01.022  1085. Tian W, Zhang J, Huang S, Song Q. Analysis of Risk Factors of Occurrence Condition of Bladder Dysfunction in Cerebral Palsy and Clinical Application of Urodynamic Parameters. Xinjiang Medical Journal. 2017;47(10):1129-33.  1086. Tong S. Analysis and countermeasures of fall related factors in neurology patients. Health Guide. 2017;(33):190,7. doi: 10.3969/j.issn.1006-6845.2017.33.182  1087. Tu Q, Tuo X. Sarcopenia,frailty and nutrition. Chinese Journal of Clinical Healthcare. 2021;24(3).  1088. Wan X, Zhu F. Application of Bedside Cranial Soft Channel Drainage in Intracranial Hematoma. Modern Hospital Journal. 2016;16(10):1437-8. doi: 10.3969/j.issn.1671-332X.2016.10.010  1089. Wang H, Wang L. Analysis of related factors and cognitive function in the elderly with oligomyosis. China Medical Herald. 2021;(8).  1090. Wang W, Cui L, Zhuang Y, Wu L, Xu X. Research progress of anxiety/depression in patients with chronic obstructive pulmonary disease. Journal of Clinical Medicine in Practice. 2014;(9):191-4. doi: 10.7619/jcmp.201409072  1091. Wang B, Wang C, Sun H, Guo S. Analysis of clinical features, serologic and cerebrospinal fluid tests in patients with neurosyphilis at different stages. Chinese Journal of Contemporary Neurology and Neurosurgery. 2016;16(7):404-10. doi: 10.3969/j.issn.1672-6731.2016.07.005  1092. Wang C. Effect of sevoflurane-remifentanil combined anesthesia on central vascular stress response in patients with giant uterine fibroids. Heilongjiang Medical Journal. 2022;46(9):1066-7. doi: 10.3969/j.issn.1004-5775.2022.09.014  1093. Wang D. Relationship between neurotransmitter networks and acetylcholine metabolism in patients with Parkinson's disease. Laboratory Medicine and Clinic. 2020;17(2):269-72. doi: 10.3969/j.issn.1672-9455.2020.02.041  1094. Wang F, Dong Y, Ma D, Jia Q, Ma Y. Clinical progress of Hegu needling. International Journal of Traditional Chinese Medicine. 2021;43(6):613-7. doi: 10.3760/cma.j.cn115398-20200228-00239  1095. Wang F, Lv S, Dong Z. Clinical study of Yizyitongluo capsule treating vascular dementia. Modern Journal of Integrated Traditional Chinese and Western Medicine. 2002;11(4):295-7. doi: 10.3969/j.issn.1008-8849.2002.04.010  1096. Wang H, Wu X, Yuan J, Han Y, Zhang Y, Meng H, et al. Research progress in physiology and pathology of Wnt5a/Frizzled-2/Ca2+and Wnt3a/Frizzled pathways. Journal of Jilin University(Medicine Edition). 2021;47(3):811-8. doi: 10.13481/j.1671-587Ⅹ.20210335  1097. Wang H, Hai S, Liu Y, Liu Y, Zhou J, Yang Y, et al. Prevalence of Sarcopenia and Associated Factors in Community-dwelling Elderly Populations in Chengdu China. Journal of Sichuan University (Medical Sciences). 2019;50(02):224-8. doi: 10.13464/j.scuxbyxb.2019.02.016  1098. Wang H. Research progress on pathogenic genes and clinical phenotypes of myoclonic atonic epilepsy. Chinese Journal of Practical Pediatrics. 2020;35(1):55-60. doi: 10.19538/j.ek2020010614  1099. Wang J. Research progress of chronic diseases complicated with sarcopenia in the elderly. Practical Geriatrics. 2020;34(02):105-6.  1100. Wang J. The relationship between common senile syndrome and depression. Practical Geriatrics. 2023;37(3).  1101. Wang J, Gong X. Review of sports medicine in 2019. Science & Technology Review. 2020;38(3).  1102. Wang J, Ji X, Cui Y, Li J, Du L, Zhang Y, et al. Physical and Cognitive Frailty in Hospitalized Elderly and the Influencing Factors. Geriatrics & Health Care. 2019;25(04):451-5+9.  1103. Wang J, Li H. Comparison of efficacy of uterine artery embolization combined with hysteroscopic clearance and methotrexate combined with hysteroscopic clearance in the treatment of caesarean section scar pregnancy. Chinese Journal for Clinicians. 2017;45(6):105-6. doi: 10.3969/j.issn.2095-8552.2017.06.038  1104. Wang J, Zhang M, He J. Effects of lumbar quadratus block combined with general anesthesia in elderly patients with percutaneous nephrolithotomy. Journal of Clinical Medicine in Practice. 2023;27(6):72-5,80. doi: 10.7619/jcmp.20223324  1105. Wang J, Xiu S, Wang L. Relationship between grip strength and cognitive impairment in middle aged and elderly patients with type 2 diabetes mellitus. Journal of Shanxi Medical University. 2018;49(05):518-22. doi: 10.13753/j.issn.1007-6611.2018.05.014  1106. Wang J, Cui L, Wang H, Miao J, Zhang X. Diagnosis and Treatment of Neuronal Intranuclear Inclusion Disease:a Case Report with Blepharospasm. Practical Journal of Cardiac Cerebral Pneumal and Vascular Disease. 2021;29(2):117-25. doi: 10.12114/j.issn.1008-5971.2021.00.026  1107. Wang J. Analysis of causes of stroke in young and middle-aged patients. 中国社区医师. 2010;12(5):18. doi: 10.3969/j.issn.1007-614x.2010.05.015  1108. Wang K. A comprehensive understanding of Parkinson's disease. Health for the Elderly and Middle-Aged. 2018;(4):14-6.  1109. Wang L, Cheng Z, Li H. Memory Impairment of Patients with Parkinson's Disease. Chinese Mental Health Journal. 2004;18(7):499-500. doi: 10.3321/j.issn:1000-6729.2004.07.017  1110. Wang J, Xiu S, Wang L. Relationship between grip strength and cognitive impairment in middle aged and elderly patients with type 2 diabetes mellitus. Journal of Shanxi Medical University. 2018;49(5).  1111. Wang L, Qing X, Ling L, Xu H. Influence of ultrasound-guided transversus abdominisplane combined with diclofenac sodium suppository for anesthesia dur-ing laparoscopic hysterectomy of the patients on their cognition level,and plasma interleukin-6,C-reactive protein,corti-sol levels. Chinese Journal of Family Planning. 2021;29(11):2332-5,40. doi: 10.3969/j.issn.1004-8189.2021.11.018  1112. Wang L, Shen Y. Correlation between thyroid function and senile frailty. Chinese Journal of Geriatric Care. 2020;18(04):120-2.  1113. Wang Q, Guo J, Li B, Dong L, Zhao X, Yang Y, et al. Analysis of clinical characteristics and risk factors of sarcopenia in elderly patients with chronic kidney disease stage 3-4. Chinese Journal of Nephrology. 2023;39(7).  1114. Wang Q, Guo J, Li B, Dong L, Zhao X, Yang Y, et al. Analysis of clinical characteristics and risk factors of sarcopenia in elderly patients with chronic kidney disease stage 3-4. Chinese Journal of Nephrology. 2023;39(7):485-90. doi: 10.3760/cma.j.cn441217-20221115-01128  1115. Wang Q. Research status on hypermethioninemia. International Journal of Pediatrics. 2018;45(11):855-9. doi: 10.3760/cma.j.issn.1673-4408.2018.11.010  1116. Wang R. Drug Rehabilitation of Spasticity (review). Chinese Journal of Rehabilitation Theory and Practice. 2007;13(1):23-5. doi: 10.3969/j.issn.1006-9771.2007.01.006  1117. Wang S. Clinical effects of single Sialic four hexose sodium injection ganglioside in treating the periventricular leukomniacia. Clinical Medicine of China. 2009;25(6):596-7. doi: 10.3760/cma.j.issn.1008-6315.2009.06.013  1118. Wang T. Therapeutic effect and application value of acupuncture and Shaofuzhuyu decoction in the treatment of uterine fibroids. Special Health. 2023;(4):85-7.  1119. Wang W, Wang H. Multiple sclerosis induced vertigo. Chinese Journal of Contemporary Neurology and Neurosurgery. 2005;5(5):318. doi: 10.3969/j.issn.1672-6731.2005.05.013  1120. Wang W. Old age disease you didn't know about. Health for Everyone. 2016;(7):43.  1121. Wang W. Introduction to the 3rd International Symposium on Hypertension and Related Diseases. Chinese
[truncated: 1,146,597 more chars]
